# 4′-*O*-substitutions determine selectivity of aminoglycoside antibiotics

**DOI:** 10.1038/ncomms4112

**Published:** 2014-01-28

**Authors:** Déborah Perez-Fernandez, Dmitri Shcherbakov, Tanja Matt, Ng Chyan Leong, Iwona Kudyba, Stefan Duscha, Heithem Boukari, Rashmi Patak, Srinivas Reddy Dubbaka, Kathrin Lang, Martin Meyer, Rashid Akbergenov, Pietro Freihofer, Swapna Vaddi, Pia Thommes, V. Ramakrishnan, Andrea Vasella, Erik C. Böttger

**Affiliations:** 1 Laboratorium für Organische Chemie, ETH Zürich, Wolfgang-Pauli-Strasse 10, 8093 Zürich, Switzerland; 2 Institut für Medizinische Mikrobiologie, Universität Zürich, Gloriastrasse 30/32, 8006 Zürich, Switzerland; 3 MRC Laboratory of Molecular Biology, Francis Crick Avenue, Cambridge Biomedical Campus, Cambridge CB2 0QH, UK; 4 Institute of Systems Biology, Universiti Kebangsaan Malaysia, 43600, Bangi, Selangor, Malaysia; 5 Euprotec Limited, Unit 12 Williams House, Manchester Science Park, Lloyd Street North, Manchester M15 6SE, UK; 6 These authors contributed equally to this work

## Abstract

Clinical use of 2-deoxystreptamine aminoglycoside antibiotics, which target the bacterial ribosome, is compromised by adverse effects related to limited drug selectivity. Here we present a series of 4′,6′-*O*-acetal and 4′-*O*-ether modifications on glucopyranosyl ring I of aminoglycosides. Chemical modifications were guided by measuring interactions between the compounds synthesized and ribosomes harbouring single point mutations in the drug-binding site, resulting in aminoglycosides that interact poorly with the drug-binding pocket of eukaryotic mitochondrial or cytosolic ribosomes. Yet, these compounds largely retain their inhibitory activity for bacterial ribosomes and show antibacterial activity. Our data indicate that 4′-*O*-substituted aminoglycosides possess increased selectivity towards bacterial ribosomes and little activity for any of the human drug-binding pockets.

## Abstract (web summary)

Aminoglycoside antibiotics target the ribosome but their limited selectivity for the bacterial ribosome can cause side effects in humans. Here, the authors synthesize 4′-*O*-ether or 4′,6′-*O*-acetal modifications and show that these compounds possess increased selectivity against bacterial ribosomes.

## Graphical abstract

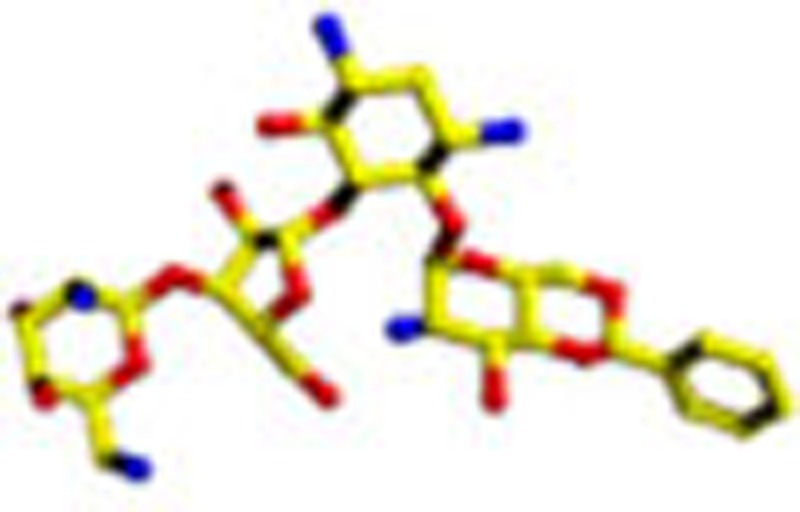

Selectivity is of particular concern for ribosomal antibiotics^1^,^2^ as the ribosome is present in all three domains of life and is a relatively conserved structure. Among this class, aminoglycosides are listed by the WHO as critically important antimicrobials for human therapy^3^. Their high efficacy, broad-spectrum antibacterial potency and lack of drug-related allergy are well-known features and make aminoglycosides a common choice for the treatment of serious infections including multidrug-resistant tuberculosis^4^. A major drawback of aminoglycosides relates to their adverse effects. Aminoglycoside-induced ototoxicity, that is, the compounds’ ability to cause irreversible hearing loss due to destruction of inner ear sensory hair cells^5^, occurs in a sporadic, dose-dependent manner. In addition, inherited forms of hypersensitivity to aminoglycoside ototoxicity exist, which are linked to point mutations in mitochondrial rRNA, such as A1555G and C1494U^6^,^7^. Recent evidence converges on mitochondrial function as a key element in aminoglycoside-induced ototoxicity, as experimental evidence was provided for both aminoglycoside-induced dysfunction of the mitochondrial ribosome and A1555G/C1494U-linked mitochondrial hypersusceptibility to aminoglycoside antibiotics^8^,^9^. These findings suggest that aminoglycoside ototoxicity is directly related to the drugs’ mechanism of action on the eukaryotic ribosome.

The 2-deoxystreptamine aminoglycoside antibiotics bind to helix 44 (h44) of 16S rRNA, which is part of the decoding site of the bacterial 30S ribosomal subunit^10^,^11^, thereby decreasing translational fidelity and inhibiting translocation^12^,^13^,^14^,^15^. The interaction core formed by rings I and II is mainly responsible for drug binding. Ring I intercalates into the internal loop formed by A1408, A1492, A1493 and the base pair C1409–G1491. Here ring I becomes properly positioned by stacking interaction with G1491 and the formation of hydrogen bonds with A1408 (Fig. 1). Despite variations in chemical composition, ring I always binds in the same orientation and forms a pseudo base-pair interaction with the Watson–Crick edge of adenine 1408. The ring oxygen of ring I accepts a hydrogen bond from the N6 of adenine, and the amino- or hydroxyl-group at position 6′ donates a hydrogen bond to the N1 of adenine^10^,^11^. Additional hydrogen bonds link the hydroxyl groups at positions 3′ and 4′ of ring I to the phosphate groups of the two bulged adenine bases 1492 and 1493, further stabilizing the position of ring I.

The use of 2-deoxystreptamine aminoglycosides as antimicrobial agents builds upon the compounds’ preferential activity for the prokaryotic versus the eukaryotic ribosome^1^,^2^. At the structural level, the selectivity of these compounds rests upon two residues in the drug-binding pocket—residues 1408 and 1491 (refs 16, 17, 18, 19). Residue 1408 is an adenine in bacterial and mitochondrial ribosomes as compared with a guanine in cytosolic ribosomes. Residue 1491 is a guanine in bacterial ribosomes, but a cytosine in mitochondrial and an adenine in cytosolic ribosomes. Both these changes disrupt the bacterial C1409–G1491 base-pair interaction (see Supplementary Fig. 1 for a comparison of bacterial and eukaryotic drug-binding pockets). A guanine at residue 1408 mainly affects 2-deoxystreptamines with a 6′NH_2_ as it would preclude the proper insertion of a corresponding ring I into the binding pocket, that is, the 6′ ammonium group cannot accept hydrogen bonds from the Watson–Crick sites of the guanine residue, and additionally its positive charge would create repulsion against the N1 and N2 amino groups^20^. In contrast, 2-deoxystreptamines with a 6′OH group are less affected by a 1408G as the 6′ hydroxyl group could still become an acceptor of a hydrogen bond from N1 or N2 (ref. 20; Fig. 1). A C1409–C1491 opposition mainly affects 2-deoxystreptamines with a 6′OH but much less so compounds with a 6′NH_2_. Possibly, the 6′OH–N1 A1408 interaction is less stable as compared with the 6′NH_2_–N1 A1408 interaction, making 6′OH 2-deoxystreptamines highly dependent on proper stacking interaction with residue 1491 (refs 21, 22).

Here we address the question of whether the specificity of the 2-deoxystreptamine aminoglycosides can be modified to increase selectivity at the drug-target level in spite of the constraints imposed by the high conservation of the drug-binding pocket. We focus on ring I, as it interacts with phylogenetically variable 16S rRNA residues 1408 and 1491. As we expected, structural modifications on ring I affect drug selectivity. In addition, our results reveal the unanticipated finding that well-known synthetic intermediates can show promising biological properties.

## Results

### Synthesis of compounds and assessment of specificity

**Figure 1:**
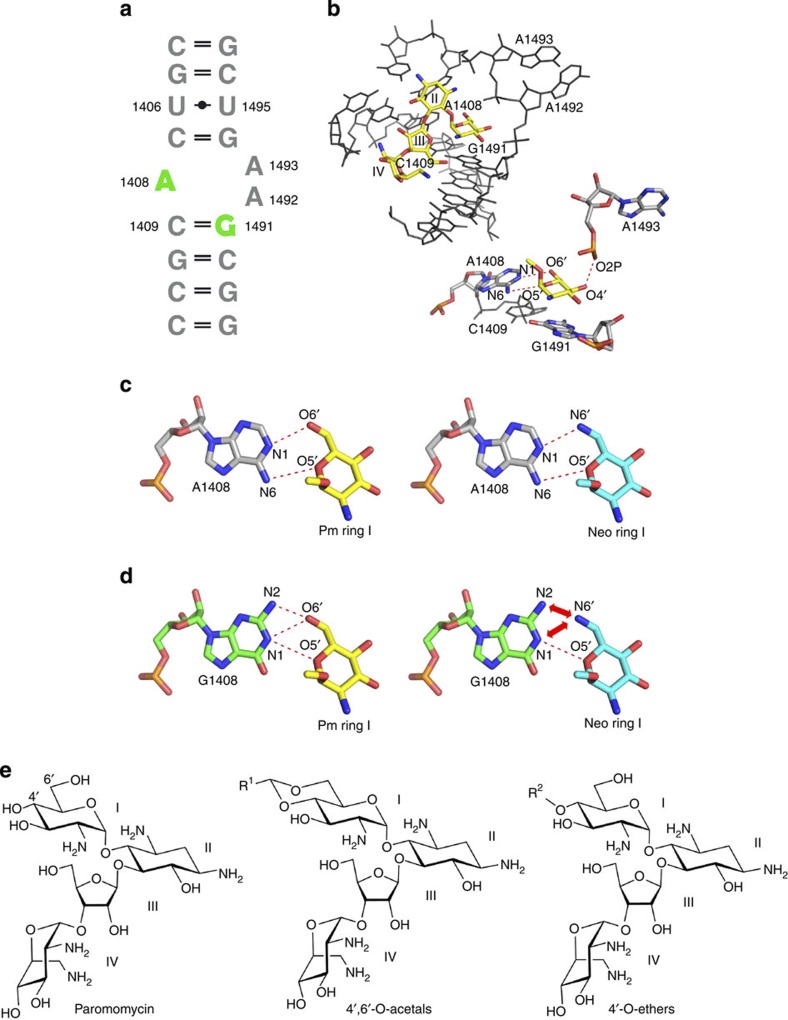
Interaction of ring I with residues in the A site loop of 16S rRNA. (**a**) Secondary structure of the aminoglycoside-binding pocket in helix 44 of 16S rRNA. Key polymorphic residues determining the selectivity of aminoglycosides are residues 1408 and 1491, highlighted in bold green. (**b**) Overview of paromomycin bound to the bacterial A site. Detailed view of the 6′OH paromomycin ring-I stacking interaction with G1491 and hydrogen bonding with A1408 and A1493 (ref. 10). Hydrogen bonds between aminoglycoside ring I and A1408 are shown as red dotted lines, as is hydrogen bonding between 4′OH and O2P of A1493. (**c**) Ring I interaction with A1408. Left paromomycin (Pm), right neomycin (Neo). Hydrogen bond interaction between the 6′-subsituent (6′OH, 6′NH_2_) and the N1 of A1408 is indicated by a red dotted line, as is hydrogen bond interaction between O5′ and N6 of A1408. (**d**) Ring I interaction with G1408 (model)^20^. Left paromomycin, right neomycin. Possible hydrogen bond interactions are indicated by red dotted lines. The positive charge of neomycin’s 6′-ammonium group would create repulsion against the N1 and N2 amino groups of G1408, indicated by red arrows. (**e**) General chemical structure of 4′,6′-*O*-acetals and 4′-*O*-ethers in comparison to paromomycin, the 6′ and 4′ positions that were target for substitution are indicated.

Paromomycin and neomycin are closely related 4,5-disubstituted aminoglycosides, their structure differing by the ring I 6′ substituent (6′OH versus 6′NH_2_, see Fig. 1c,d). Ring I of paromomycin was modified by substitutions of the 4′ hydroxyl position. We first synthesized 4′,6′-*O*-acetals, starting with benzylidene derivatives (Fig. 1e). The 4′,6′-*O*-benzylidene acetal 1 was synthesized and tested for growth inhibition activity (minimal inhibitory concentration, MIC) of bacterial cells using wild type and recombinant strains of *Mycobacterium smegmatis* with single point mutations in the drug-binding pocket. The point mutations were chosen to reflect the phylogenetically variable 16S rRNA residues, namely positions 1408 (A bacterial/mitochondrial, G cytosolic) and 1491 (G bacterial, A cytosolic, C mitochondrial). We subsequently synthesized acetals 2, 3, 4, 5, 6, 7, 8, 9, 10, 11, 12, 13, 14, 15, 16, 17, 18, 19, 20, 21, 22, 23, 24, 25, 26, 27, 28, 29, 30, 31, 32, 33, 34, 35, 36 (for a complete list of chemical structures synthesized see Supplementary Fig. 2) to investigate structure–activity relations (SAR) and compared their growth inhibition activity with those of neomycin and paromomycin. As previously noted^21^, the interaction of neomycin and paromomycin with the A site shows varying degrees of specificity for A1408 and G1491. Thus, the MIC activities (Table 1) demonstrate that interaction of the 6′ amino neomycin is dependent on an adenine at residue 1408 (MIC for A1408 is 0.8 μM, MIC for G1408 is >720 μM), while mutational alterations of G1491 have less of an effect (MIC G1491 0.8 μM versus MIC C1491 27 μM). In comparison, interaction of the 6′ hydroxyl paromomycin with the ribosome is less affected by a G1408 alteration (MIC A1408 1.6 μM versus MIC G1408 102 μM) as compared with mutational alteration of residue G1491 (MIC G1491 1.6 μM versus MIC C1491 >720 μM). Surprisingly, the interaction of the 4′,6′-*O*-acetals with the A site was observed to be largely dependent on both rRNA residues 1408 and 1491. Thus, high MIC values are associated with each single nucleotide alteration affecting rRNA residue A1408 or G1491 (MIC G1408 ≥720 μM, MIC C/A1491 ≥720 μM). MIC activities for selected acetals 1, 2, 3, 9 and 30 are summarized in Table 1.

SAR studies based on >30 acetals (see Supplementary Table 1 for a summary of MIC activities) revealed that the nature, position and number of substituents at the equatorial hydrophobic residue at C(2) of the acetal 1,3-dioxane ring have a modest effect on compound activity in general, for example, replacement of the phenyl group in 1 by a 3- or 4-chlorophenyl substituent (10, 2), a 4-fluorophenyl substituent (6), a 3,5-dichlorophenyl analogue (16), a 3- or 4-methoxyphenyl analogue (11, 3), a 2,5-dimethoxyphenyl analogue (18), a 4-dimethylaminophenyl (4), a 3- or 4-hydroxyphenyl (12, 5), a 4-trifluoromethylphenyl (9), a 2-, 3- or 4-nitrophenyl analogue (15, 13 and 7) all have relatively little effect. Together with the lower activity of the cyclohexyl analogue 25 of 1 this suggests that both hydrophobic and stacking interactions are relevant to the drug–ribosome interaction. This interpretation is in agreement with the activities of 23 and 24, where the phenyl substituent has been replaced by an electron-rich 2-furyl or 2-thiophenyl ring, and with the activities of 26 and 27, possessing larger aromatic moieties. Introducing a linker between the phenyl ring and C(2) of the 1,3-dioxane ring had a marked effect on the properties of the acetals: a two-carbon chain between the aromatic moiety and C(2) of the 1,3-dioxane ring being optimal (1, 29, 30, 31, 32, 33, 34). Disruption of ring I of the benzylidene acetals abolishes activity (35 and 36 versus 1).

We next synthesized the C(4′)*O*-substituted ethers 37 and 39, 40, 41, 42, the linear acetal 42 and the C(6′)*O*-substituted ether 38. The MIC data (Table 2) demonstrate a similar selectivity profile for the C(4′)*O-*substituted ethers 37 and 39, 40, 41, 42 as for the corresponding 4′,6′-*O*-acetal analogues 1, 2, 3, 9 and 30, respectively. The acetal 1 and the C(4′)-*O*-benzyl ether 37 show significantly higher antibacterial activity than the corresponding C(6′)-*O*-benzyl ether 38, and the linear acetal 43 is noticeably less active than the corresponding cyclic acetal 30 (see summarized MIC data in Supplementary Table 1). Altogether, this indicates that proper orientation and location of the aromatic residue rather than just hydrophobic effects are relevant for selectivity.

### Drug–target interaction in cell-free translation assays

To study drug–target interaction and the contribution of the polymorphic residues 1408 and 1491 to drug binding more directly, we assessed compound activity in cell-free ribosomal translation assays for selected pairs of 4′,6′-*O*-acetals and corresponding 4′-*O*-ethers, that is, compounds 1, 2, 30, 37, 39 and 42. Compound activity in the *in vitro* translation assay is defined as the drug concentration that inhibits the *in vitro* translation reaction by 50% (inhibitory concentration, IC_50_). Corroborating the MIC data, the ribosomal activity of the 4′,6′-*O*-acetals and 4′-*O*-ethers was found to be highly dependent on both specific nucleobases present at 16S rRNA positions 1408 and 1491, with little difference between corresponding acetals and ethers (Table 3). To address the question of how these compounds affect the eukaryotic A site we assessed drug susceptibility of recombinant bacterial hybrid ribosomes. The hybrid ribosomes were engineered to carry the cytoplasmic A site, the mitochondrial wild-type A site or the mitochondrial mutant deafness A site (A1555G, C1494U)^8^,^9^,^18^. Neomycin and paromomycin each showed a characteristic pattern of interaction with the eukaryotic drug-binding pockets. In addition, both aminoglycosides interact preferentially with the mitochondrial deafness A1555G, C1494U mutant A site (Table 4). In contrast, the series of 4′,6′-*O*-acetals and 4′-*O*-ethers did not show a preferential activity for any of the eukaryotic drug-binding pockets—these compounds are poor inhibitors of mitochondrial, mitochondrial mutant deafness and cytosolic hybrid ribosomes.

**Figure 2:**
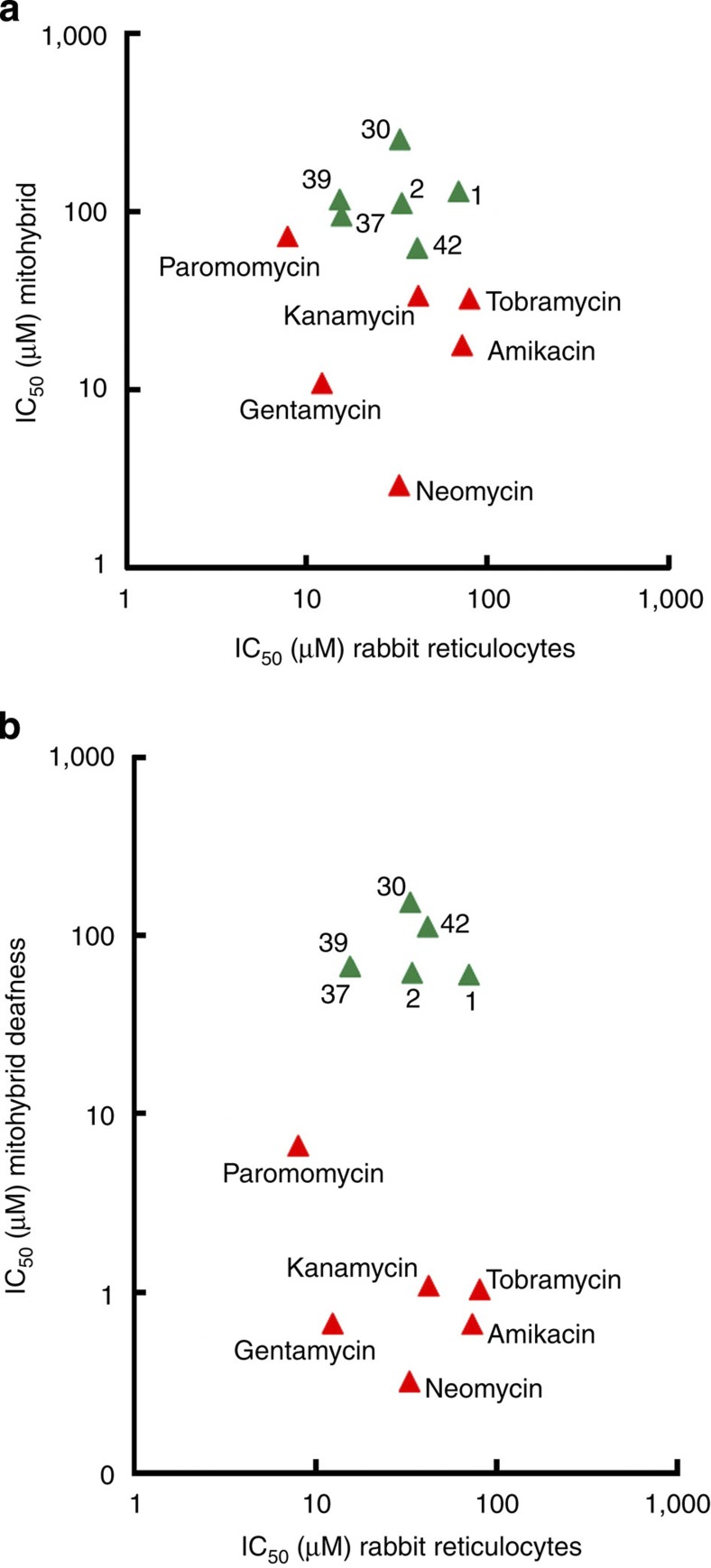
Drug-induced inhibition of protein synthesis in ribosomes. Inhibition of protein synthesis depicted as IC_50_ (μM); IC_50_ values represent the drug concentrations required to inhibit *in vitro* synthesis of firefly luciferase to 50%. (**a**) *y* axis: IC_50_ mitohybrid ribosomes, *x* axis: IC_50_ rabbit reticulocyte ribosomes; (**b**) *y* axis: IC_50_ mitohybrid deafness ribosomes, *x* axis: IC_50_ rabbit reticulocyte ribosomes.

We compared the target specificity and drug–target interaction of the present series of compounds with that of paromomycin, neomycin, gentamicin, tobramycin, amikacin and kanamycin. Aminoglycoside activity against 1491C and 1491A mutant bacterial ribosomes correlates well (*R*^2^=0.97), yet no such correlation exists between the activity against 1491C and 1408G mutant ribosomes (Supplementary Fig. 3). Compared with available 4,5 and 4,6 2-deoxystreptamines and as per interaction with the eukaryotic ribosome the present series of compounds have retained aminoglycoside selectivity for the cytosolic ribosome, but show decreased interaction with mitochondrial wild type and, in particular, mitochondrial mutant deafness hybrid ribosomes (Fig. 2).

Aminoglycoside-mediated dysfunction of the eukaryotic ribosome has been connected to drug cytotoxicity in mammalian cells^9^,^18^,^23^,^24^. To assess the potential cytotoxicity of the 4′,6′-*O*-acetals and the 4′-*O*-ethers we determined cell toxicity of compounds 1, 30, 37 and 39 in HEK293 cells. Cytotoxicity of the compounds was significantly lower than that of geneticin, an aminoglycoside known to be cytotoxic to mammalian cells (see Supplementary Fig. 4).

### Determination of antibacterial activity

To assess antimicrobial activity, we determined the MIC values of compounds 1, 2, 30, 37, 39 and 42 against clinical isolates of *Escherichia coli* and *Staphylococcus aureus* in comparison to those of paromomycin and aminoglycosides used in the clinic for the treatment of infectious diseases, that is, gentamicin, tobramycin, kanamycin and amikacin (Table 5). The acetals and ethers—in particular the arylalkyl ethers 39 and 42—showed antimicrobial activity that was comparable to that of the parental paromomycin.

**Figure 3:**
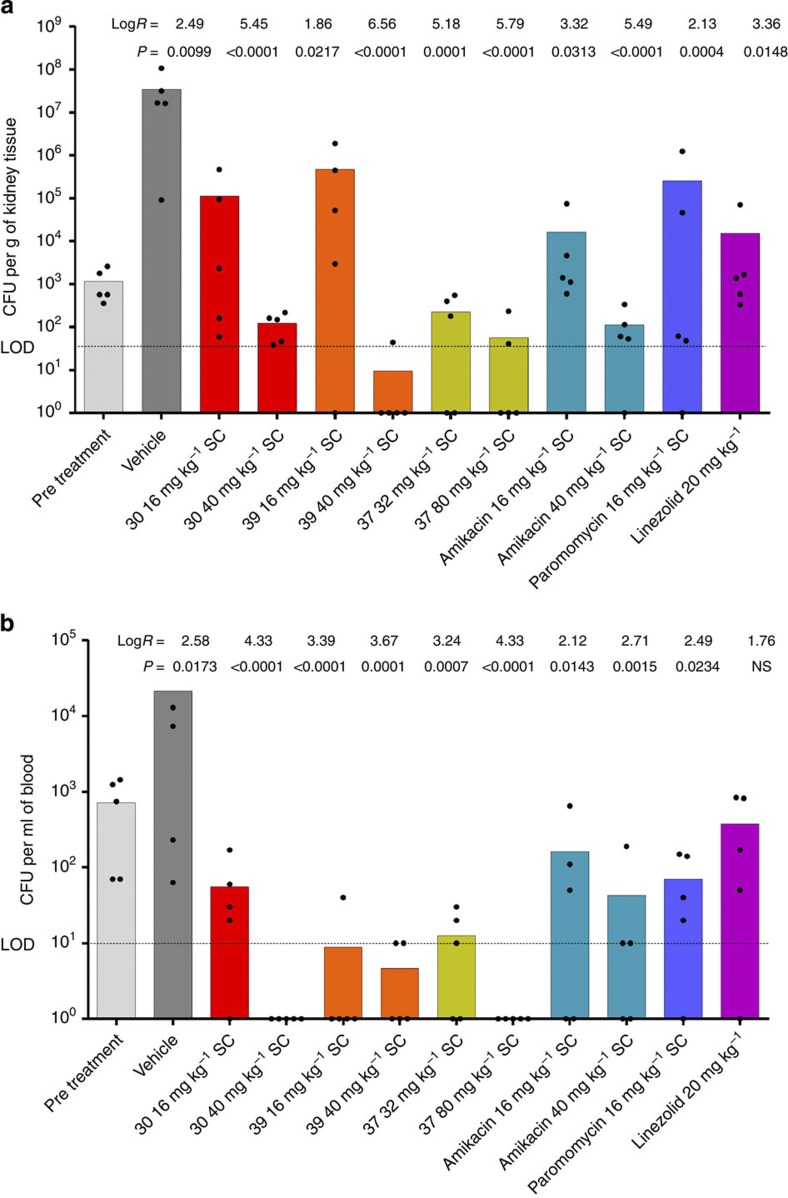
*In vivo* activity of aminoglycoside compounds and comparators in a murine septicaemia model. (**a**) Bacterial burden in kidney, CFU per g tissue. (**b**) Bacterial burden in blood, CFU ml^−1^. The CFU values for the individual animals are indicated by dots, the mean of the group (five mice per treatment group) by the bar. The log reduction compared with the vehicle control as well as the *P*-values (non-parametric Kruskal–Wallis using pairwise comparisons) are indicated as numbers above each group. Colour coding—pre-treatment: light grey; vehicle control: dark grey; 30: red; 39: orange; 37: green; amikacin: light blue; paromomycin: dark blue; linezolid: violet. LOD, limit of detection.

To investigate whether the *in vitro* activity of our compounds translates to activity *in vivo*, we determined the antibacterial *in vivo* activity in neutropenic murine models of septicaemia^25^,^26^. Efficacy of compounds 30, 37 and 39 was compared with amikacin and the parental paromomycin. In animals infected with methicillin-resistant *S. aureus*, treatment with 30, 37 and 39 reduced the bacterial burden both in blood and in kidney and was as effective as the comparator amikacin and the parental paromomycin. Compared with the vehicle-treated mice, drug treatment reduced bacterial burden in the kidney between 3 to 6 log_10_ and in blood between 2 to 3 log_10_ in a dose-dependent manner. Among all drugs studied, the greatest efficacy in reducing *S. aureus* burden in the blood was following treatment with 30 and 37, which both reduced the bacterial burden below detectable limits (Fig. 3).

### Crystal structure analysis

The distinct ribosomal activity profile of the acetal and ether derivatives differentiates them from available 4,5- and 4,6-disubstituted aminoglycosides. To study the structural basis of drug-target interaction we determined the three-dimensional (3D) structure of a number of the acetals and ethers in complex with the small ribosomal subunit of *Thermus thermophilus*. Two acetals, the parental phenyl derivative 1 and the most active phenethyl analogue 30, and two ethers, the 4′*-O***-**benzyl ether 37 and the closely related 4-(chlorophenyl) methyl ether 39 were chosen for analysis.

**Figure 4:**
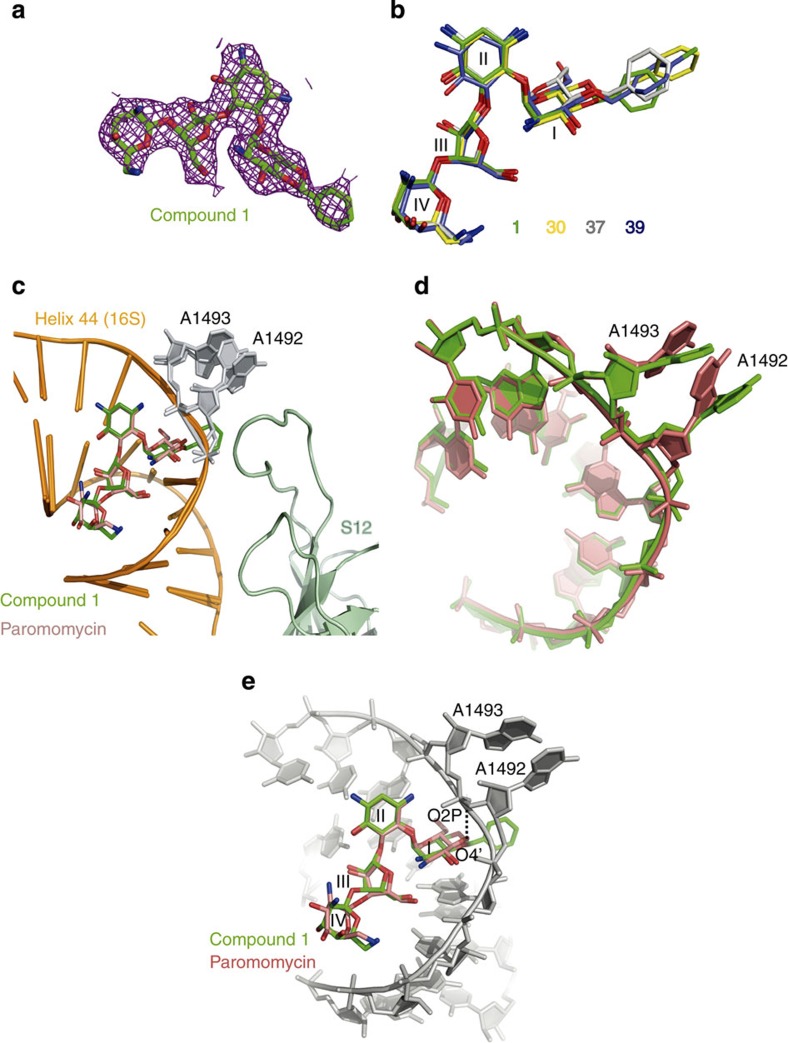
Structures of antibiotics bound to the decoding center of the 30S ribosomal subunit. (**a**) 3*F*_o_—2*F*_*c*_ difference Fourier density around compound 1 (green) contoured at 1.0σ. (**b**) Superposition of antibiotics in the decoding center, colour coding as indicated by the numbering. (**c**) Decoding center of the 30S subunit (helix 44 of 16S RNA, orange; protein S12, light green), showing compound 1 in green. The conformation of A1492 and A1493 (grey) is shown. The benzyl ring is in proximity to the nucleobase of the flipped-out A1492. Also shown is a superposition with paromomycin (Par, salmon). (**d**) Superposition of helix 44 of 16S RNA for structures of 30S-compound 1 (green) and 30S-paromomycin (salmon, Protein Data Bank ID code 1FJG); the orientations of A1492 and A1493 that are flipped-out from helix 44 are tilted relative to the paromomycin structure. (**e**) View of compound 1 and loss of a hydrogen bond with O2P of A1493 compared with paromomycin. The benzyl ring is in proximity to the nucleobase of the flipped-out A1492.

The overall structures of the 30S subunit in complex with all four compounds were globally similar to previous 30S ribosomal subunit structures (with RNA phosphate root mean-squared deviation of ~0.6 Å). Both acetals (1, 30) and ethers (37, 39) were found inserted in the major groove of helix 44 of 16S rRNA in a manner that resembles the structures of 30S–apramycin^27^ and 30S–paromomycin^10^ complexes (Fig. 4 and Supplementary Figs 5 and 6). Similar to paromomycin, rings II, III and IV of all four derivatives maintained their interactions with residues in helix 44, showing that neither the acetal nor the ether modifications alter the fundamental binding mode to the ribosome. However, the orientations of A1492 and A1493 that are extruded from helix 44 were tilted relative to the paromomycin structure (Fig. 4c,d). The synthetically added substituents of compounds 1 and 30, which result in a ring system mimicking apramycin’s bicyclic moiety, stacked perfectly with the nucleobase of G1491. Unlike all other aminoglycosides that form a pseudo basepair interaction between ring I and A1408 consisting of two hydrogen bonds, no pseudobase interaction was observed for the acetal series as a consequence of the absence of hydrogen bonding with N1 of A1408. The loss of this interaction constitutes the main structural difference between the benzylidene and benzyl compounds examined (37, 39). For both the benzyl and benzylidene compounds (1, 30, 37 and 39), the 4′**-***O***-**substituent prevents hydrogen bonding with O2P of A1493 (Fig. 4e). The aromatic substituent is positioned in the minor groove of the 1409–1491 basepair in proximity to the backbone and ribose of A1492. It mainly points out into the solvent and does not show stacking interaction to ribosomal moieties.

**Figure 5:**
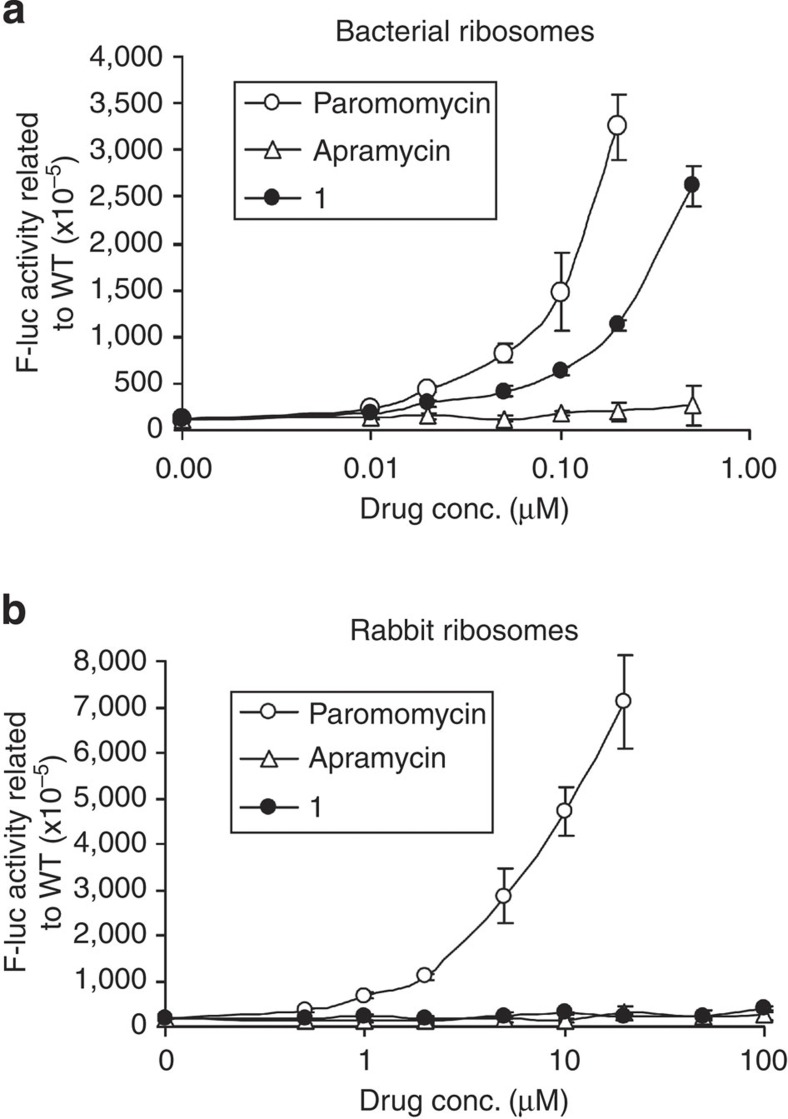
Aminoglycoside-induced misreading. Dose–response curves of aminoglycoside-induced misincorporation of amino acids using the H245R near-cognate mutant F-luc mRNA as template. Shown is luciferase activity upon translation of mutant template relative to wild-type F-luc mRNA (mean±s.d.; *n*=3). (**a**) Bacterial ribosomes; (**b**) rabbit reticulocyte ribosomes. 1 (filled circles), paromomycin (open circles) and apramycin (open triangles).

The 3D structure of the drug–ribosome interaction and the benzylidene acetal’s chemical structure resemble that of apramycin, while the IC_50_ values for mutant ribosomes indicate important differences. Compared with the 4′,6′-*O*-acetals and their susceptibility to mutational alteration of residue 1491, the antiribosomal activity (IC_50_) of apramycin is strongly affected by the A1408G alteration (see Supplementary Table 2 and compare to Table 3). To challenge these disparate findings we synthesized 44 by removing rings 3 and 4 from the 4′,6′-*O*-benzylidene acetal 1 (for a comparison of the structure of 44 and apramycin both isolated and in complex with the ribosome, see Supplementary Fig. 7). In contrast to apramycin, compound 44 had virtually no activity on any of the bacterial ribosomes tested (Supplementary Table 2). Further evidence for a distinct mode of interaction of the 4′,6′-*O*-benzylidene acetals with the ribosome as compared with apramycin was found in their ability to induce misreading. The effects were studied on bacterial ribosomes and on eukaryotic cytosolic ribosomes, using acetal 1 as representative in comparison to apramycin and paromomycin. Acetal 1 induced pronounced misreading on bacterial ribosomes but little if any misreading on eukaryotic ribosomes (Fig. 5). This unique pattern of misreading induction differentiates acetal 1 from both the parental paromomycin (misreading on both bacterial and eukaryotic ribosomes) and the comparator apramycin (no misreading on bacterial or eukaryotic ribosomes).

## Discussion

Here we addressed the question of whether it is possible to modify 4,5-disubstituted 2-deoxystreptamine aminoglycosides that show a preference for either the cytoplasmic ribosome (paromomycin) or the mitochondrial ribosome (neomycin), but which have comparable activities for the bacterial ribosome. With a view to develop aminoglycoside derivatives that are more selective at the ribosomal target level, chemical synthesis was guided by a collection of mutant bacterial ribosomes with single point mutations in h44. Previous work has established the important role of this mutant model system in understanding the SAR of aminoglycosides and mechanisms of antibiotic action^20^,^21^. A selective response of aminoglycosides to alterations of rRNA residues 1408 or 1491 (*E. coli* numbering) can potentially overcome drug ototoxicity^8^,^9^,^17^,^27^.

We succeeded in the synthesis of a series of compounds that exploit the polymorphic residues present in the drug-binding pocket. Phylogenetic selectivity in aminoglycoside binding involves rRNA residue 1491C for mitoribosomes and rRNA residues 1491A and 1408G for cytoribosomes^9^,^17^,^18^. Compared with the parental scaffolds paromomycin and neomycin the 4′,6′-*O*-acetals and 4′-*O*-ethers are highly susceptible to any alteration of residues 1408 or 1491 (Table 3). Hybrid ribosomes carrying the cytoplasmic A site, the mitochondrial wild-type A site or the mitochondrial deafness A site (A1555G, C1494U) were used to study compound interaction with the eukaryotic drug-binding pockets. These bacterial hybrid ribosomes have been shown to faithfully reflect drug susceptibility of eukaryotic ribosomes^8^,^9^,^18^,^27^. In contrast to paromomycin or neomycin, the series of 4′,6′-*O*-acetals and 4′-*O*-ethers have lost most, if not all of the preferential activity for any of the eukaryotic drug-binding pockets—these compounds bind poorly to mitochondrial wild-type and cytosolic hybrid ribosomes (Table 4) and have also lost the aminoglycosides’ characteristic activity for the mitochondrial mutant deafness A site^9^. However, these compounds retain to a large extent their activity for bacterial ribosomes and show good antibacterial activity *in vitro* and *in vivo* (Table 5, Fig. 3). These results identify C(4′) and its substituents as promising sites through which the drug–target interaction can be manipulated for increased selectivity.

By crystal structure analysis an additional aminoglycoside-binding site at helix 69 of the 23S rRNA has been postulated in bacterial ribosomes^28^,^29^. Although single-molecule measurements point to an involvement of H69 in aminoglycoside-mediated inhibition of translocation and ribosome recycling^30^, the implications of this suggested additional binding site for aminoglycoside action are as yet unclear, as mutations in H69 reportedly have not been associated with aminoglycoside resistance. In contrast, single point mutations in the A site at 16S rRNA positions 1408 and 1491 are both necessary and sufficient to confer non-responsiveness to aminoglycoside action in bacteria^16^,^20^,^21^.

The molecular basis for the compounds’ increased selectivity was revealed by the observation that it is possible to separate compound interaction with residue 1408 (main denominator of cytosolic selectivity) from compound interaction with residue 1491 (main denominator of mitochondrial selectivity), that is, aminoglycoside activity for A1408G mutant ribosomes does not go hand-in-hand with activity for G1491C mutant ribosomes. This separation of ribosomal activities has provided the basis for the synthesis of more selective aminoglycosides, that is, compounds with a selective decrease in activity for mitoribosomes (mitochondrial wild type, mitochondrial mutant deafness) but not at the expense of increased activity for the cytoribosome (Fig. 2).

The structural basis for the altered specificity of the series of modified aminoglycosides is unclear. Globally, the crystal structures of the bacterial 30S subunit in complex with compounds 1, 30, 37 and 39 resemble the structures of available 30S–aminoglycoside complexes^10^,^11^, in particular the 30S–apramycin complex^27^, even if subtle differences are present (see Results and Fig. 4). This work exemplifies the difficulty of predicting specificity at the typical resolutions of ~3 Å as for current ribosomal crystal structures. Such specificity may arise as the result of subtle energetics that cannot easily be deduced from the structures. A more thorough understanding of the structural basis for the compounds’ selectivity will require a careful comparison with structures of both higher eukaryotic cytosolic and mitochondrial ribosomes in complex with 2-deoxystreptamine aminoglycosides, preferably at higher resolutions than possible to date. Such structures are currently not feasible because the appropriate experimental conditions have not been established. At the functional level, misreading-inducing activity on bacterial ribosomes but not on eukaryotic ribosomes (Fig. 5) and the compounds’ unique structure–activity profiles of drug–mutant ribosome interaction (Table 3) differentiates them from available aminoglycosides, and establishes the series of 4′ modifications as a novel chemotype with a unique mode of interaction with h44.

Interest in aminoglycosides has recently seen a revival not least because structural details of drug–target interactions and aminoglycoside-modifying enzymes have provided a wealth of knowledge^31^,^32^,^33^, facilitating hypothesis-driven approaches to developing aminoglycosides recalcitrant to resistance determinants^34^,^35^,^36^. The description of aminoglycoside–ribosome complexes at atomic resolution^10^,^11^ has greatly stimulated efforts at structure-based design of new bioactive derivatives^37^,^38^,^39^,^40^,^41^,^42^. However, rational design of specificity using these structures is limited by the current resolution of crystallographic analysis and an incomplete understanding of the thermodynamic and kinetic factors involved in antibiotic binding^43^,^44^. These limitations in understanding small molecule–RNA interactions and the constraints imposed by the limited sequence polymorphism in the drug-binding pocket make it a formidable challenge to synthesize aminoglycosides that are more selective at the drug-target level. The combined chemical synthesis, structural analysis, genetics and functional studies employed here have enabled us to manipulate a complex biological system of drug–target interaction. By introducing defined substitutions we have identified a new chemotype of aminoglycosides with increased selectivity and little activity for any of the human drug-binding pockets, an important step towards the development of less toxic aminoglycosides.

## Methods

### Antibiotics

Paromomycin, neomycin, gentamicin, tobramycin, kanamycin, amikacin and apramycin were obtained from Sigma.

### Compound synthesis

Chemical synthesis procedures are detailed in the Supplementary Methods.

### Strains harbouring mutant ribosomes

The construction of these strains, which are derived from single rRNA allelic *M. smegmatis ΔrrnB*, has been described previously^8^,^18^. They consist of recombinant mutant strains with single point mutations in the small ribosomal subunit A site (16S rRNA positions 1408A→G, 1491G→A and 1491G→C) and mutant strains with hybrid ribosomes where the bacterial A site had been replaced by various eukaryotic homologues (cytoribosome, mitoribosome and deafness mitoribosome).

### Bacterial strains

Clinical isolates of *E. coli* and *S. aureus* were obtained from the Diagnostic Department, Institute of Medical Microbiology, University Zurich. MIC values were determined by broth microdilution assays. Microtitre plates were incubated overnight for *E. coli* and *S. aureus*, and 72 h for *M. smegmatis.*

### *In vivo* infection experiments

All animal experiments were performed under UK Home Office Licenses with clearance by the ethical review committee at the University of Manchester. Male mice aged 7–8 weeks were used in this study. Mice were supplied by Charles River UK and were specific pathogen-free. The strain of mouse used was Hsd:ICR (CD-1), which is a well-characterized outbred strain. Mice weights at the start of the experiment were 22–25 g. Mice were housed in sterile individual ventilated cages with free access to sterile food and water and were exposed to 12 h light/dark cycles with dawn/dusk phases. Five mice were used in each group.

Mice were rendered temporarily neutropenic by immunosuppression with cyclophosphamide at 200 mg kg^−1^ 4 days before infection and 150 mg kg^−1^ 1 day before infection by intraperitoneal injection. The immunosuppression regime leads to neutropenia starting 24 h post administration, which continues throughout the study. For *in vivo* infection a methicillin-resistant strain of *Staphylococcus aureus*, clinical isolate MRSA AG041, was used. Twenty four hours post the second round of immunosuppression mice were infected with *S. aureus* MRSA AG041 by intravenous injection into the lateral tail vein using ~1 × 10^7^ CFU per mouse. This strain had the following MIC values (μM given in brackets)—amikacin 4.0 mg l^−1^ (7.0), paromomycin 4.0 mg l^−1^ (6.5), 30 4.0 mg l^−1^ (5.6), 37 8.0 mg l^−1^ (11.2), 39 4.0 mg l^−1^ (5.6) and linezolid 1.0 mg l^−1^. Compounds 30, 37, 39 and comparator amikacin were administered at 4 times and at 10 times the MIC value in mg kg^−1^, the parental paromomycin at four times the MIC value in mg kg^−1^. Linezolid (20 mg kg^−1^) was used as positive control. Test articles and comparators were reconstituted and diluted in 0.9% saline. Dosing solutions were prepared immediately prior to administration of the first dose and stored at 4 °C between treatments. Antibacterial treatment was initiated 1 h post infection and delivered subcutaneously at 10 ml kg^−1^ (linezolid was given by intravenous bolus injection). All drugs were administered at 1, 9 and 17 h post infection.

At 1 h (pre-treatment group) or 24 h post infection blood samples were collected by cardiac puncture under isoflurane anaesthesia and mice were humanely killed using Pentobarbitone overdose. Both kidneys were removed and homogenized in 2 ml ice cold sterile phosphate-buffered saline. Kidney homogenates were quantitatively cultured onto mannitol salt agar (MSA) and incubated at 37 °C for 24 h before being counted. Individual blood samples were quantitatively cultured onto cysteine lactose electrolyte-deficient (CLED) agar and incubated at 37 °C for 24 h before being counted. Data were analysed, by StatsDirect software (version 2.7.8), using the non-parametric Kruskal–Wallis test (pairwise comparisons, Conover–Inman).

### Isolation and purification of ribosomes

Ribosomes were purified from bacterial cell pellets as described previously^45^. In brief, ribosome particles were isolated by successive centrifugation and fractionated by sucrose gradient (10–40%) centrifugation. The 70S ribosome-enriched fraction was pelleted, resuspended in association buffer, incubated for 30 min at 4 °C, dispensed into aliquots and stored at −80 °C following shock freezing in liquid nitrogen. Ribosome concentrations of 70S were determined by absorption measurements on the basis of 23 pmol ribosomes per A_260_ unit. Integrity of purified 70S ribosomes was determined by analytical ultracentrifugation.

### Cell-free luciferase translation assays

Purified 70S hybrid ribosomes were used in translation reactions of luciferase mRNA. Luciferase mRNA was produced *in vitro* using T7 RNA polymerase (Thermo Scientific) on templates of modified plasmids pGL4.14 (firefly luciferase) and pGL4.75 (renilla luciferase, both Promega), where the mammalian promoter driving transcription of luciferases was replaced by the T7 bacteriophage promoter. A typical translation reaction with a total volume of 30 μl contained 0.25 μM 70S ribosomes, 4 μg firefly (F-luc) mRNA, 0.4 μg renilla (R-luc) mRNA, 40% (vol/vol) *M. smegmatis* S100 extract, 200 μM amino acid mixture, 24 units of RiboLock (Thermo Scientific), 0.4 mg ml^−1^ tRNAs, and energy was supplied by addition of 12 μl of commercial S30 Premix without amino acids (Promega). In addition to ribosomes, rabbit reticulocyte lysate (Promega) was used for *in vitro* translation of F-luc mRNA. A standard 30 μl reaction contained 20 μl reticulocyte lysate, 4 μg F-luc mRNA, 0.4 μg R-luc mRNA, amino-acid mixture (200 μM each) and 24 units RiboLock. Following addition of serially diluted aminoglycosides, the reaction mixture was incubated at 37 °C for 35 min and stopped on ice. Thirty microliter samples of the reaction mixture were assayed for luciferase activities using the Dual Luciferase Reporter Assay System (Promega). Luminescence was measured using a luminometer FLx800 (Bio-Tek Instruments).

Misreading was assessed in a gain–of-function assay as described previously^27^. In brief, we introduced Arg245 (CGC, near-cognate codon) into the firefly luciferase protein to replace residue His245 (CAC codon). Arg245 F-luc mRNA and wt F-luc mRNA were used in *in vitro* translation reactions; in addition, R-luc mRNA was used as internal control. We quantified misreading by calculating mutant firefly/renilla luciferase activity as compared with wild-type firefly/renilla luciferase activity.

### Crystal structure analysis

Crystals obtained from purified *T. thermophilus* 30S ribosomes^46^ were soaked for 4 days in cryoprotectant solution (100 mM MES-KOH (pH 6.5), 200 mM KCl, 75 mM NH_4_Cl, 15 mM MgCl_2_, 26% MPD and 100μM antibiotic) before being flash-frozen. Crystals were pre-screened and data sets were collected at the European Synchrotron Radiation Facility (ESRF). Data were integrated and scaled using XDS^47^. A starting model consisting of the empty 30S ribosome (without anticodon-stem loop, mRNA and ions) was used for initial refinement and phase calculation using CNS^48^. The anticodon-stem loop and mRNA were then fitted into the unbiased difference map (*mF*_*o*_–*DF*_*c*_ map) and the model was subjected to another round of refinement. Finally, ligands were placed manually into unbiased difference maps (*mF*_*o*_–*DF*_*c*_ map) using COOT^49^, which were refined to resolutions between 2.9 and 3.5 Å. Data and refinement statistics are reported in Supplementary Table 3.

## Additional information

**How to cite this article:** Perez-Fernandez, D. *et al*. 4′-*O*-substitutions determine selectivity of aminoglycoside antibiotics. *Nat. Commun.* 5:3112 doi: 10.1038/ncomms4112 (2014).

**Accession codes:** Coordinates and structure factors for 30S-compound 1 complex, 30S-compound 30 complex, 30S-compound 37 complex and 30S-compound 39 complex have been deposited in the Protein Data Bank under accession codes 4b3m, 4b3r, 4b3s and 4b3t.

## Supplementary Material

Supplementary Information Supplementary Figures 1-7, Supplementary Tables 1-5, Supplementary Methods and Supplementary References

## Figures and Tables

**Table 1:** Minimal inhibitory concentrations (MIC, **μ**M) of 4**′**,6**′**-*O*-benzylidene acetals.

Bacterial A site	Neomycin Ring I	Paromomycin Ring I	1	2	3	9	30
	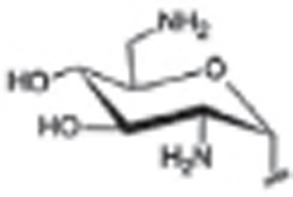	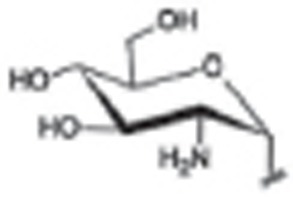	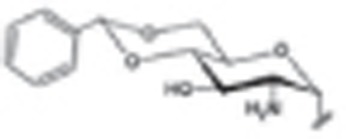	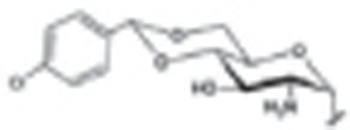	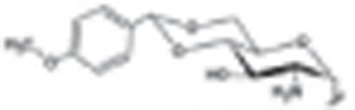	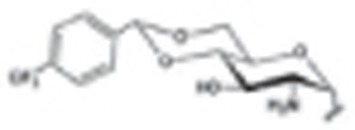	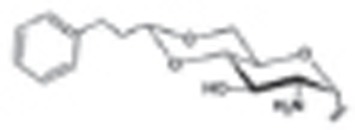
WT	0.8	1.6	5.6	5.6	5.6	2.8–5.6	2.8
1491C	27	>720	>720	>720	>720	720	≥720
1491A	3.2	51–102	720	>720	>720	720	≥720
1408G	>720	51–102	720	>720	>720	>720	720

**Table 2:** Minimal inhibitory concentrations (MIC, **μ**M) of C(4**′**)-*O*-ethers.

Bacterial A site	37	39	40	41	42
	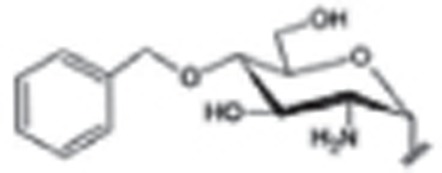	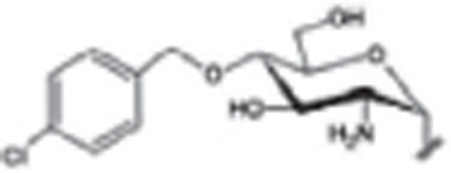	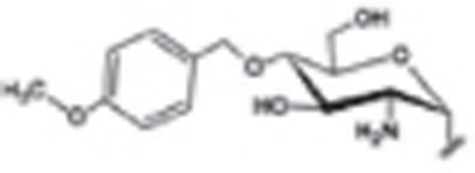	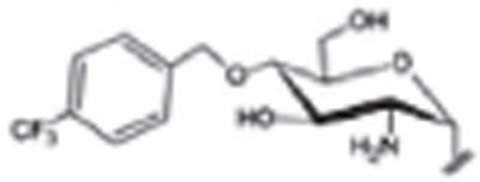	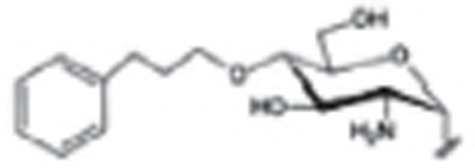
WT	5.6	2.8	11	11	5.6
1491C	>720	≥720	>720	≥720	≥720
1491A	≥720	≥720	>720	>720	≥720
1408G	>720	≥720	>720	>680	≥720

**Table 3:** Compound interaction with polymorphic residues in the drug-binding pocket (IC_50_, **μ**M).

**Bacterial A site**	** Paromomycin **	** Neomycin **	**37 4′-** ***O*** **-ether**	**1 4′,6′-** ***O*** **-acetal**	**39 4′-** ***O*** **-ether**	**2 4**′**,6′-** ***O*** **-acetal**	**42 4′-** ***O*** **-ether**	**30 4′,6′-** ***O*** **-acetal**
Wild type	0.03	0.03	0.14	0.28	0.14	0.14	0.28	0.14
G1491C	19.2	1.1	122.0	131.0	83.3	80.4	255.5	163.5
G1491A	1.1	0.08	38.4	79.3	29.0	43.0	87.4	66.4
A1408G	0.5	31.4	17.5	19.0	5.9	10.5	16.1	9.5

**Table 4:** Compound interaction with hybrid ribosomes—selectivity profile (IC_50_, **μ**M).

**Eukaryotic A site**	** Paromomycin **	** Neomycin **	**37 4′-** ***O*** **-ether**	**1 4′,6′-** ***O*** **-acetal**	**39 4′-** ***O*** **-ether**	**2 4′,6′-** ***O*** **-acetal**	**42 4′-** ***O*** **-ether**	**30 4′,6′-** ***O*** **-acetal**
Mitochondrial wild type	81.5	2.9	93.7	127.5	120.7	115.1	85.0	259.7
Mitochondrial mutant A1555G	6.7	0.3	67.9	61.3	71.5	65.8	118.6	161.8
Mitochondrial mutant C1494U	5.1	0.3	69.3	53.3	53.3	51.1	121.2	168.7
Cytosolic wild type	14.4	29.4	87.8	66.8	94.1	93.7	225.1	203.7

**Table 5:** Minimal inhibitory concentrations (MIC, **μ**M) of clinical isolates.

	** Gentamicin **	** Tobramycin **	** Kanamycin **	** Amikacin **	** Paromomycin **	**37 4′-** ***O*** **-ether**	**1 4′,6′-** ***O*** **-acetal**	**39 4′-** ***O*** **-ether**	**2 4′,6′-** ***O*** **-acetal**	**42 4′-** ***O*** **-ether**	**30 4′,6′-** ***O*** **-acetal**
*Staphylococcus aureus* (MRSA)											
AG 042	70–140	≥538	538	27	>415	22	22	5.6–11	5.6–11	5.6	5.6–11
AG 043	70–140	67	269–538	14–27	6.5–13	11–22	11–22	5.6	11	5.6–11	5.6
AG 044	35	17	134	6.8	6.5–13	22	22–45	11–22	22	11	22
AG 045	35	17	134	6.8	6.5	22	22–45	11	11–22	5.6–11	22
AG 053	70–140	34–67	538	14	6.5	11	22	5.6	11	5.6–11	5.6–11

*Escherichia coli*											
AG 001	8.8–18	8.4–17	34	n.d.	26–52	45–90	45–90	11–22	22–45	22	45–90
AG 002	4.4	4.2	8.4–17	n.d.	13–26	45–90	45–90	22	22–45	22	45–90
AG 003	560–1120	67	34–67	n.d.	13–26	45–90	45	11–22	22–45	11–22	45
AG 004	1120	134	67–134	n.d.	52	45–90	90	11–22	22–45	22	22–45
