# Supplementary material for: 4′-O-substitutions determine selectivity of aminoglycoside antibiotics
Source: Nat Commun. 2014 Jan 28;5:3112. doi: 10.1038/ncomms4112 (PMC3942853; doi:10.1038/ncomms4112)
Supplement: Supplementary Information — Supplementary Figures 1-7, Supplementary Tables 1-5, Supplementary Methods and Supplementary References [file ncomms4112-s1.pdf]

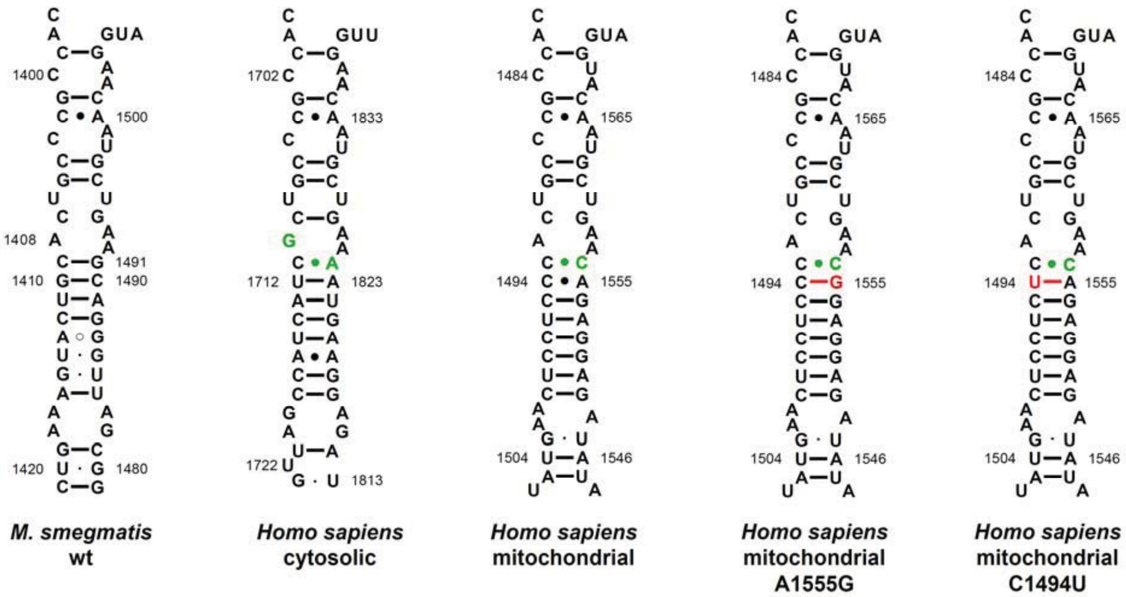

Supplementary Figure 1. Comparison of small-subunit rRNA secondary structures. Comparison of rRNA helices that correspond to the proximal part of helix 44 in bacterial 16S rRNA, representing the ribosomal small subunit's decoding site (A site). Homology modelling based on nucleotide sequence accession numbers: *M. smegmatis* WT, NC008596 (*E. coli* numbering); *Homo sapiens* cytosolic, K03432 (*H. sapiens* 18S rRNA numbering); *H. sapiens* mitochondrial, J01415 (*H. sapiens* mitoDNA numbering). The eukaryotic homologs of *E. coli* 16S rRNA residues 1408 and 1491 which are phylogenetically variable and determine drug selectivity are indicated in green, the deafness-associated mitochondrial positions A1555G and C1494U are given in red.

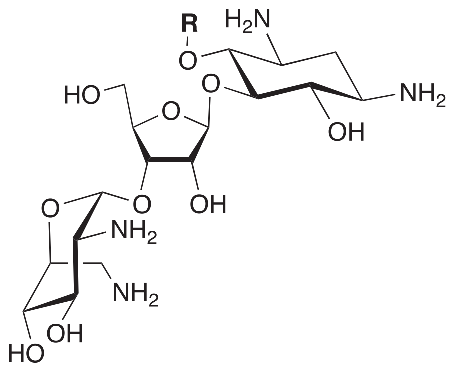

R=

1 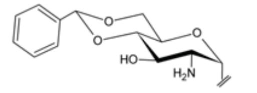 **2** 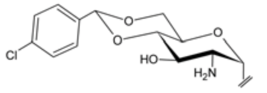 **3** 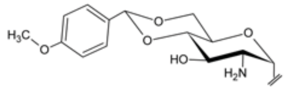

4 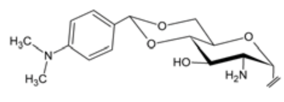 **5** 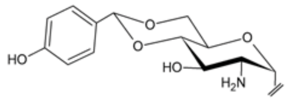 **6** 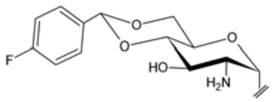

7 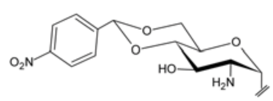 **8** 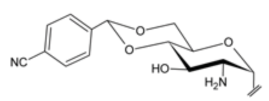 **9** 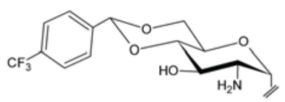

10 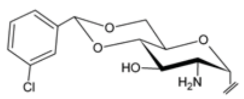 **11** 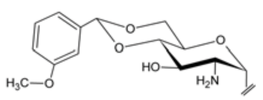 **12** 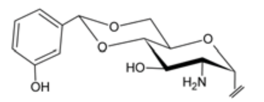

13 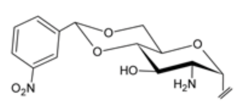 **14** 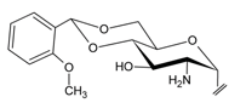 **15** 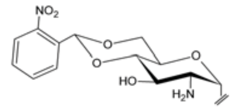

16 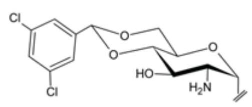 **17** 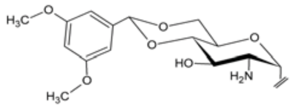 **18** 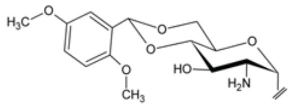

19 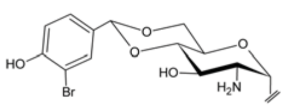 **20** 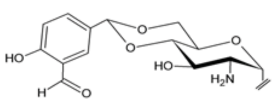 **21** 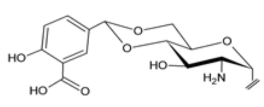

22 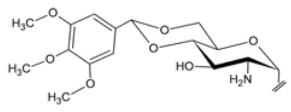 **23** 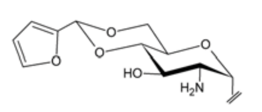 **24** 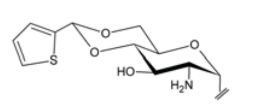

25 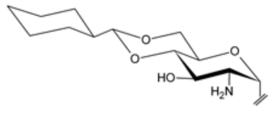 26 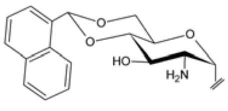 27 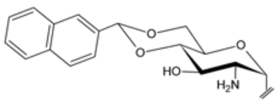

28 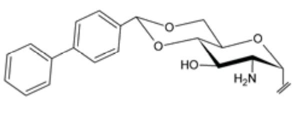 29 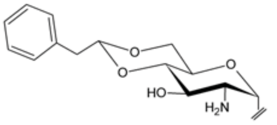 30 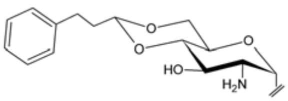

31 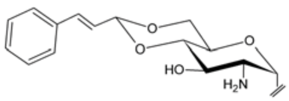 32 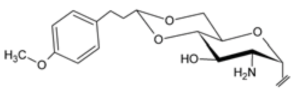 33 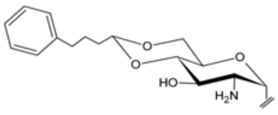

34 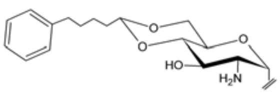 35 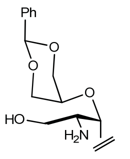 36 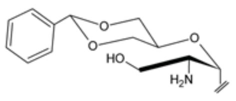

37 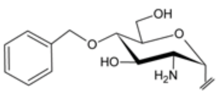 38 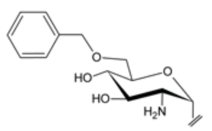 39 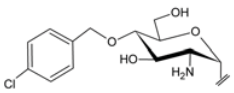

40 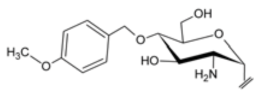 41 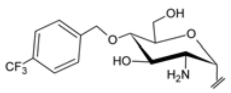 42 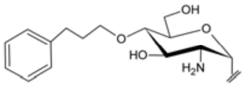

43 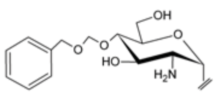

Supplementary Figure 2. Chemical structures of compounds synthesized.

a

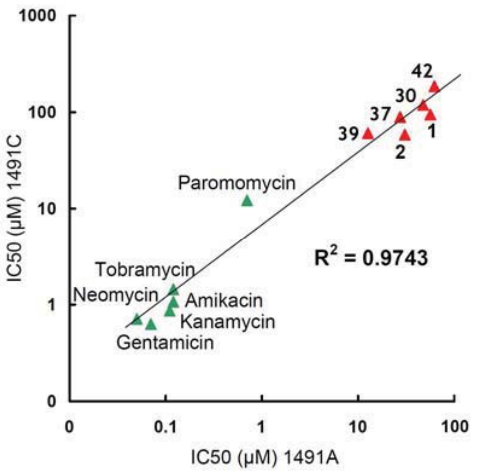

b

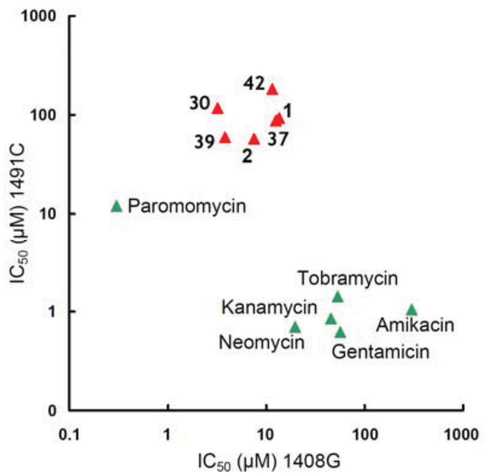

Supplementary Figure 3. Drug-induced inhibition of protein synthesis in ribosomes. Inhibition of protein synthesis depicted as IC_{50} (μM), IC_{50} values represent the drug concentrations required to inhibit *in vitro* synthesis of firefly luciferase to 50%. (**a**) y-axis: IC_{50} bacterial mutant G1491C ribosomes, x-axis: IC_{50} bacterial mutant G1491A ribosomes; (**b**) y-axis: IC_{50} bacterial mutant G1491C ribosomes, x-axis: IC_{50} bacterial mutant A1408G ribosomes.

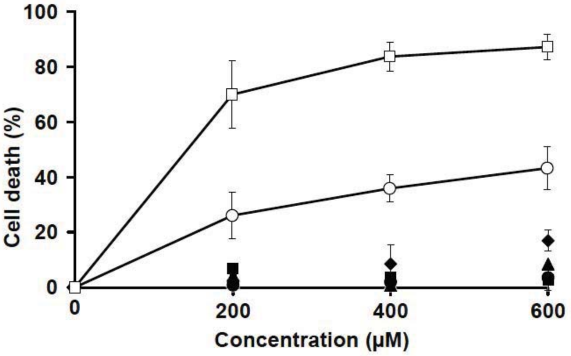

	LC_{25} (µM)
**Hygromycin**	78 ± 9.3
**Geneticin**	195 ± 16.6
**1**	> 600
**30**	> 600
**37**	> 600
**39**	> 600

Supplementary Figure 4. Compound cytotoxicity. Cytotoxicity was assessed in human embryonic kidney (HEK293) cells (mean ± SD; n = 3). Hygromycin (open boxes), geneticin (open circles), **1** (black boxes), **30** (black triangles), **37** (black circles), **39** (black diamonds). The concentration LC_{25} values, i.e., the drug concentration at which 25% of the cells in culture are non-viable, were calculated from fitting concentration-response curves to the data of at least three independent experiments using PRISM 5 software. For cytotoxicity assays, HEK-293 cells were grown in 96-well plates (4000 cells/well) in DMEM medium containing 10% FBS and 1% glutamine (100 µL/well) at 37°C and 5% CO_2. Following overnight incubation, serial dilutions of compound dissolved were added (20µL per well), and the cells were incubated for an additional 72h. Cell viability was measured using Alamar Blue fluorimetric assay (Life Technologies) according to the manufacturer's instructions. Fluorescence was measured using an FLx800 plate reader (Bio-Tek Instruments). Cell viability was calculated as the ratio between the numbers of living cells in cultures grown in the presence of the tested compounds and those in cultures grown under the identical conditions without the tested compound. Hygromycin and geneticin were used as positive controls.

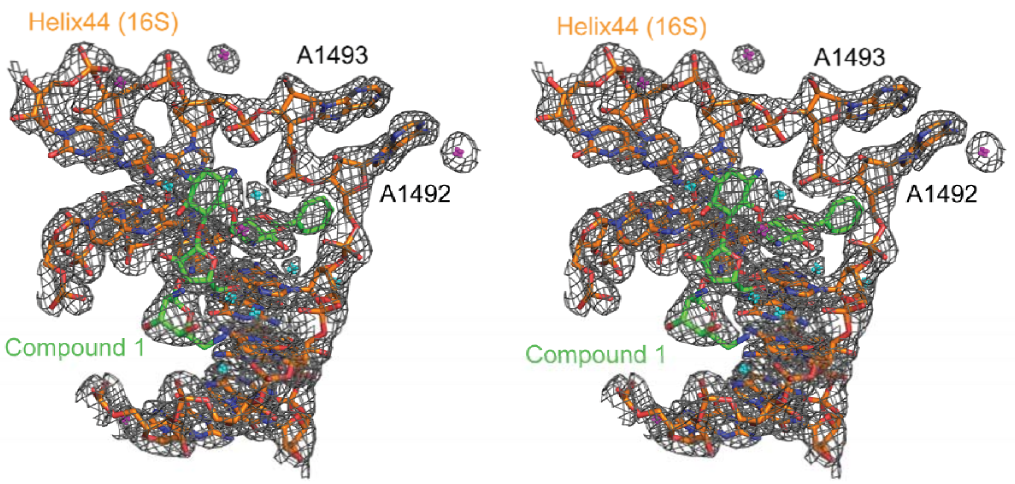

Supplementary Figure 5. Stereo view of electron density map. The stereo view of the representative electron density from the $2F_o - F_c$ map contoured at 1.5σ shows compound **1** (green) bound to the helix 44 of 16S rRNA. The magnesium ion and water molecule are shown in magenta and cyan, respectively.

37

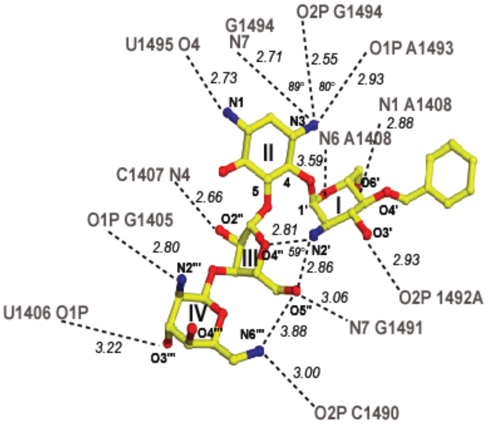

1

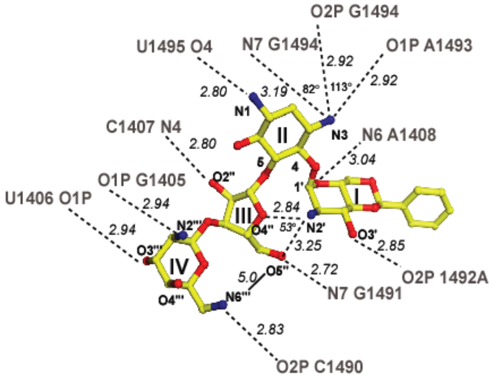

39

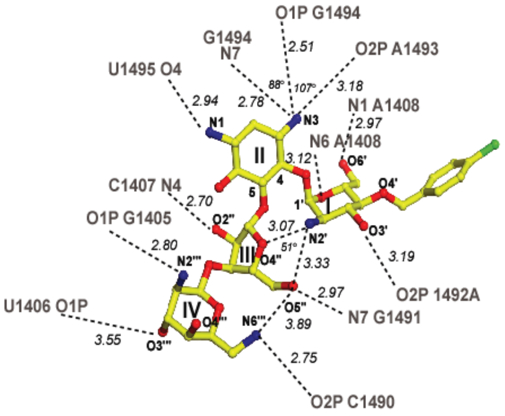

30

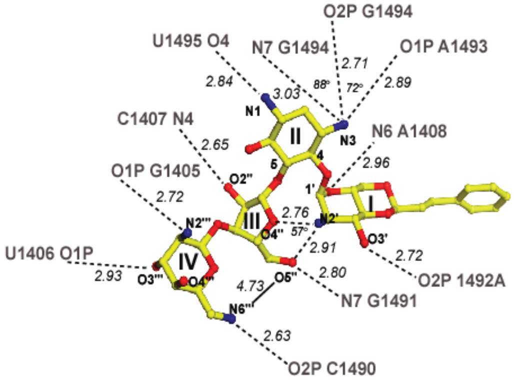

Supplementary Figure 6. Molecular interactions between 4',6'-*O*-acetals, 4'-*O*-ethers and h44 16S rRNA. Molecular interactions between 16S rRNA nucleotides and compounds **1**, **30**, **37** and **39** (distances are given in Angstrom). The hydrogen bonds are represented by grey dashed lines, with the distance length stated. Ring I, II, III and IV are indicated; ring II represents the 2-deoxystreptamine ring, ring I represents the mono- or bicyclic ring system of the ether and acetal compounds respectively.

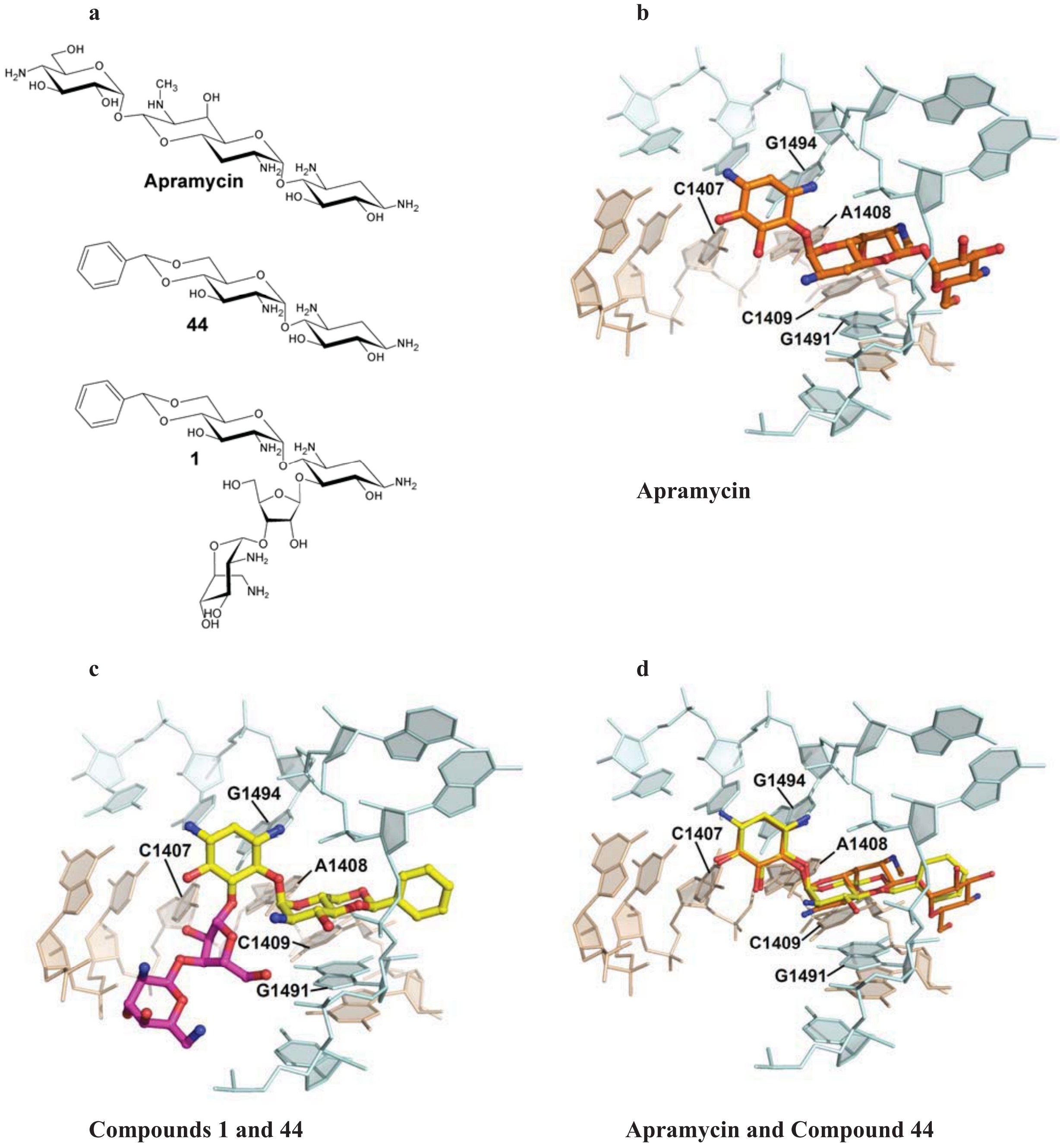

Supplementary Figure 7. Structural similarity between apramycin and 44. (**a**) Chemical structures of apramycin, **1** and **44**. (**b**) Decoding site of 30S *Thermus thermophilus* subunit in complex with apramycin[27]. Apramycin is shown in orange; *E. coli* numbering used throughout. (**c**) Modelling of the 3D structure of **44** (yellow) bound to the A-site of 30S *T. thermophilus*, based on structural similarity between **44** and **1**. Rings III and IV of **1** absent in **44** are shown in magenta. (**d**) Superimposition of modelled **44** (yellow) and apramycin (orange) bound to the A-site of 30S *T. thermophilus* in crystal structures.

Supplementary Table 1. MIC determinations (μM) of acetal and ether derivatives

	Bacterial A site			
	**WT A1408/G1491**	**G1491C Mutant A1408/G1491C**	**G1491A Mutant A1408/G1491A**	**A1408G Mutant A1408G/G1491**
**Neomycin**	0.8	27	3.2	>720
**Paromomycin**	1.6	>720	51-102	102
**1**	5.6	>720	720	720
**2**	5.6	>720	>720	>720
**3**	5.6	>720	>720	>720
**4**	2.6-5.2	>680	340	340-680
**5**	5.6	>720	360	720
**6**	2.8-5.6	>720	>720	>720
**7**	5.2	>680	>680	>680
**8**	22-45	>720	>720	>720
**9**	2.8-5.6	720	720	>720
**10**	2.8-5.6	720	720	360-720
**11**	5.6-11	>720	720	360-720
**12**	11	≥720	720	>720
**13**	2.6-5.2	>680	340	340-680
**14**	11-22	>720	>720	>720
**15**	5.2	>680	170-340	340-680
**16**	5.2	170	170-340	170-340
**17**	45	>720	>720	>720
**18**	5.2-10.5	>680	>680	≥680
**19**	2.6	680	84	170
**20**	10.5-21	84	84	84
**21**	2.6	680	84-170	170
**22**	42-84	>680	>680	>680
**23**	11-22	>720	>720	>720
**24**	11-22	>720	>720	>720
**25**	45-90	>720	>720	>720
**26**	5.2-10.5	340	340-680	680
**27**	2.6-5.2	680	680	>680

**28**	84	84	84-170	170
**29**	90	180-360	360	360
**30**	2.8	≥720	≥720	720
**31**	2.8-5.6	≥720	≥720	≥720
**32**	2.6-5.2	≥680	>680	>680
**33**	340-680	340-680	≥680	>680
**34**	≥680	>680	>680	>680
**35**	≥720	>720	>720	>720
**36**	≥720	>720	>720	>720
**37**	5.6	>720	≥720	>720
**38**	45-90	>720	>720	>720
**39**	2.8	≥720	≥720	≥720
**40**	11	>720	>720	>720
**41**	10.5	≥680	>680	>680
**42**	5.6	720	≥720	≥720
**43**	22	>720	>720	>720

Supplementary Table 2. Interaction of apramycin and compound 44 with polymorphic residues in the drug binding pocket (IC_{50}, µM)

	bacterial wt	G1491C	G1491A	A1408G
**Apramycin**	0.08	71	11	>200
**44**	>200	>200	>200	>200

Supplementary Table 3. Summary of crystallographic data and refinements for compound 1, 30, 37 and 39 complexed with the 30S ribosomal subunit

	**30S-compound 1 complex**	**30S-compound 30 complex**	**30S-compound 37 complex**	**30S-compound 39 complex**
**Data collection**				
Space Group	$P4_12_12$	$P4_12_12$	$P4_12_12$	$P4_12_12$
Cell dimensions (Å)				
a<br>*b*<br>*c*	401.7<br>401.7<br>175.3	402.5<br>402.5<br>175.0	401.2<br>401.2<br>173.6	401.7<br>401.7<br>173.9
$\alpha = \beta = \gamma$ (°)	90	90	90	90
Resolution (Å)	2.9	3.0	3.15	3.0
Rsym or Rmerge	11.9 (141.4)	12.9 (144.4)	17.2 (95.7)	12.1 (120.6)
$I / \sigma I$	14.3 (1.9)	12.3 (2.1)	8.2 (2.1)	14.8 (2.2)
Completeness (%)	99.5 (98.6)	99.2 (97.6)	99.2 (99.5)	99.8 (99.8)
Redundancy	7.4	6.6	4.0	7.4
**Refinement**				
Resolution (Å)	40.0-2.9	40.0-3.0	40.0-3.15	40.0-3.0
No. reflections	311372	281438	240081	280162
R_{work}/R_{free}	21.8/25.1	21.4/24.8	21.0/24.7	21.0/24.3
No. atoms				
Protein/RNA	52180	52123	53083	52122
Ligand/Ion/Water	325	281	230	289
B-factors				
RNA	83.8	79.0	77.6	80.0
Protein	94.3	89.4	88.1	90.6
Ion	73.7	66.7	59.7	69.0
Ligand	67.6	61.2	60.5	70.3
R.m.s deviations				
Bond lengths (Å)	0.007	0.006	0.007	0.007
Bond angles (°)	1.218	1.226	1.239	1.222

Values in parentheses are for the highest resolution shell.

Supplementary Table 4. Formation of the azido acetals 47-57

Entry	Product	R	Temperature	Time	Yield
1	**47**	4-Cl	26–70°	36 h	31%[a])
2	**48**	4-MeO	26°	1 h 20	69%
3	**49**	4-F	65°	1 h 10	69%
4	**50**	4-NO_2	70°	2 h	35%
5	**51**	4-CN	70°	19 h	38%
6	**52**	3-MeO	24°	4 h	40%
7	**53**	3-NO_2	60°	23 h	26%
8	**54**	2-MeO	24°	2 h 30	66%
9	**55**	3,5-Cl_2	60°	22 h	23%
10	**56**	2,5-$(MeO)_2$	60°	2 h 15	73%
11	**57**	4-Ph	50°	1 h 30	68%

[a]) The starting material was completely consumed and the low yield was due to the formation of the paromamine analogue of **47** (30%) resulting from cleavage of the glycosidic bond to ring III.

Supplementary Table 5. Preparation of the protected acetals 61–80

Entry	Compound	R	Method	Time	Yield
1[e,f])	**61**	4- Me_2N-phenyl	B	8 h	46%
2[l])	**62**	4-HO-phenyl	B	2 h	62–70%
3	**63**	3-CF_3-phenyl	B	19 h	73%
4	**64**	3-Cl-phenyl	A	20 min	61%
5	**65**	3-HO-phenyl	B	3 h 30 min	55%
6[c,d])	**66**	2-NO2-phenyl	B	28 h	29%
7[a])	**67**	3,5-$(MeO)_2$-C_6H_3	B	22 h	72%
8	**68**	3-Br,4-OH-C_6H_3	B	3 h 30 min	60%
9[b])	**69**	3,4,5-$(MeO)_3$-phenyl	B	21 h	70%
10[h])	**70**	2-furanyl	A	40 min	58%
11[i,j])	**71**	2-thiophenyl	A	22 h	42%
12	**72**	cyclohexyl	A	5 h	49%
13[g])	**73**	1-naphthyl	A	20 min	71%
14	**74**	2-naphthyl	B	23 h	66%
15[k])	**75**	$PhCH_2$	A	2 h	76%
16	**76**	$Ph(CH_2)_2$	A	30 min	96%
17	**77**	PhCH=CH	A	2 h	57%
18	**78**	4-MeO-$C_6H_4(CH_2)_2$	B	5h 40 min	78%
19	**79**	$Ph(CH_2)_3$	B	26 h	91%
20	**80**	$Ph(CH_2)_4$	B	2 h 15	94%

[a]) 0.2 equiv. $TsOH.H_2O$ instead of 0.5 equiv. for 1 h: 39%. [b]) Average yield of two experiments. [c]) With 1.2 equiv. CSA instead of $TsOH.H_2O$. [d]) 0.5 equiv. $TsOH.H_2O$: 21% yield after 48h. [e]) With 0.8 equiv. $TsOH.H_2O$. [f]) 1 equiv. CSA: 42% after 2 h 30. [g]) CSA, 23 h: 66%. [h]) 64% conversion. [i]) 1.5 equiv. of $FeCl_3$, 24 h: 45% yield and 44% conversion. [j]) 2 equiv. of $FeCl_3$, 24 h at 55°: 43% yield. [k]) No reaction at 0°, starting material recovered. [l]) 1 equiv. CSA, 2 h 30: 35% yield.

Supplementary Methods

Synthesis – 4’,6’-*O*-Benzylideneparomomycin (**1**) was obtained in a yield of 64% by benzylidenating pentaazido paromomycin **45** to **46**[50] followed by a Staudinger reaction and hydrolysis.

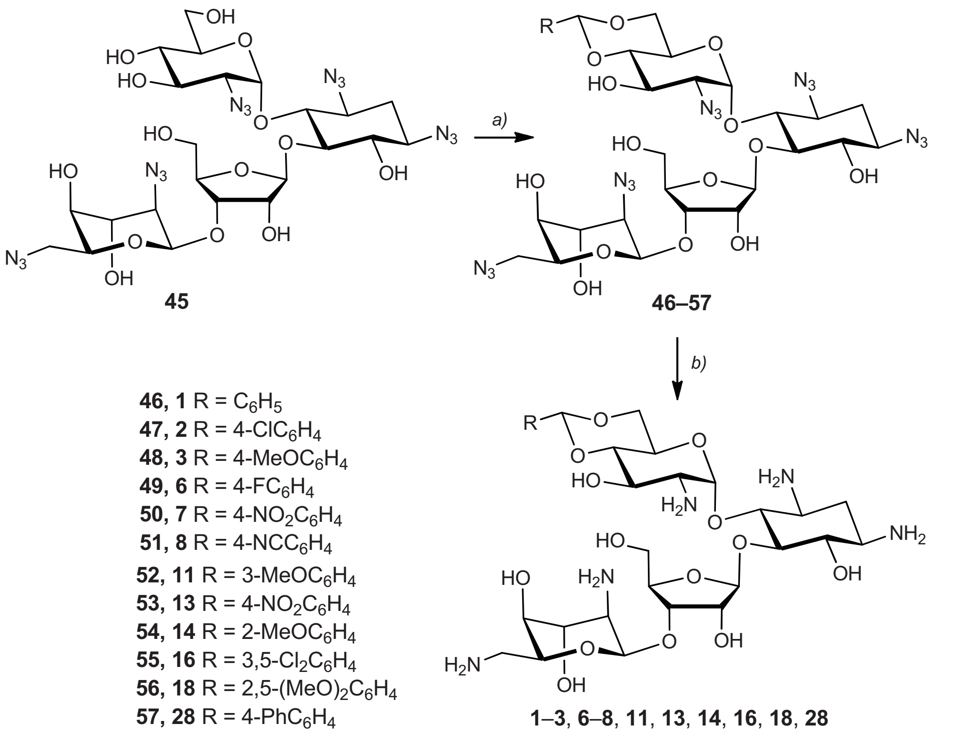

a) ArylCH$(OMe)_2$, TsOH-H_2O, DMF, 25–60°; *b)* PMe_3, NaOH, aq. THF, 60°; *or* HS$(CH_2)_3$SH, Et_3N, MeOH.

Acetals **47**–**57** were synthesized by acid-catalyzed transacetalation of **2** in DMF with the dimethyl acetals of the corresponding substituted benzyaldehydes. The dimethyl acetals possessing an electron-donating substituent were prepared as described according to Clerici and coworkers.[51] Dimethyl acetals possessing electron-withdrawing substituents were synthesized according to Hassner and coworkers.[52] Reaction conditions for the synthesis of benzylidene acetals **47**–**57** and yields are given in Supplementary Table 4. Transacetalation leading to acetals possessing electron-donating phenyl substituents (entries 1–4 and 10) proceeded in yields of 40–73% at room temperature, or upon heating to 50–60°. Harsher conditions were required for the preparation of 4’,6’-O-benzylidene acetals possessing electron-withdrawing phenyl substituents (entries 5–9 and 11), and the yields were lower.

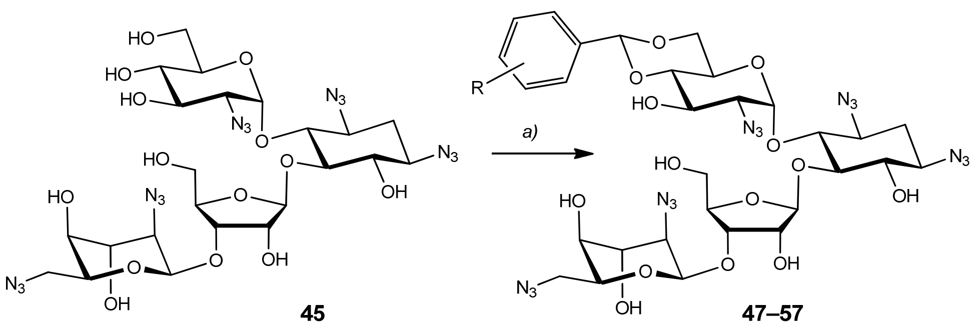

a) 5 equiv. $ArCH(OMe)_2$, 0.5 equiv. $TsOH.H_2O$, DMF.

Treatment of **45** with benzaldehyde dimethyl acetal in DMF and in the presence of $TsOH.H_2O$ at ambient temperature proceeded very slowly. The mixture was heated to 65° for 1 h. No change was observed. Additional benzaldehyde dimethyl acetal and $TsOH.H_2O$ were added. After 24 h, the starting material was consumed. FC yielded **46** (13%) and the paromamine derived benzylidene acetal **58** (58%) resulting from glycoside cleavage.

Staudinger reaction/hydrolysis of **46**–**57** afforded the deprotected derivatives **1–3** and **6–8, 11, 13, 14, 16, 18,** and **28** in good yields (61–>98%), with the exception of the 4-cyanobenzylidene derivative **51** that was treated with propane-1,3-dithiol in MeOH in the presence of Et_3N[52] to give the deprotected derivative **8** (78%). Similary, **58** provided the paromamine derived acetal **44**.

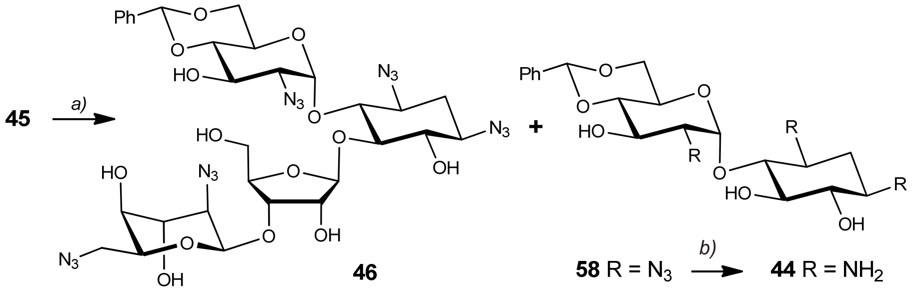

a) $PhCH(OMe)_2$, $TsOH\text{-}H_2O$, DMF, 65°, 25 h; (58% **58**; 13% **44**); *b)* PMe_3, NaOH, aq. THF, 60°(89%).

To avoid glycoside cleavage during the preparation of acetals derived from less reactive substituted benzaldehydes, we used the selectively *O*-acetylated derivative **60**. The solubility of diol **60** allowed performing the acetalation in toluene, and the increased resistance to acid-promoted glycoside cleavage allowed acetalation at a higher temperature than the one tolerated by **45**. Diol **60**

was obtained (77%)[1]) by selective hydrolysis of **59**[27] with one equivalent of TsOH.H_2O in MeOH. For the formation of acetals of liquid aldehydes, $FeCl_3$ was added to a solution of **60** in the aldehyde (Supplementary Table 5, entries 3, 7, 9–14, 17, 18). The acetals were obtained in yields between 42 and 96%. The lowest yield resulted from using 2-thiophene carboxaldehyde (42%; entry 10); it was neither increased by adding more $FeCl_3$ (1.5 instead of 0.5 equiv.), nor by heating to 55°, as determined by recovering the starting diol. All other acetals were obtained by treating diol **60** and the corresponding aldehyde[2]) in toluene at reflux, in the presence of 5 Å molecular sieves and cat. amounts of TsOH.H_2O. The acetals were obtained in good overall yields, with the exception of the 2-nitrobenzylidene derivative **66** (29%, entry 4).

[1]) Prolonged reaction times resulted in lower yields due to partial deacetylation. Reaction with 0.5 equivalents of TsOH.H_2O gave somewhat lower yields (61%) after 5 h.

[2]) Aldehydes (known) that were not commercially available were prepared by Dess Martin oxidation of the alcohol.

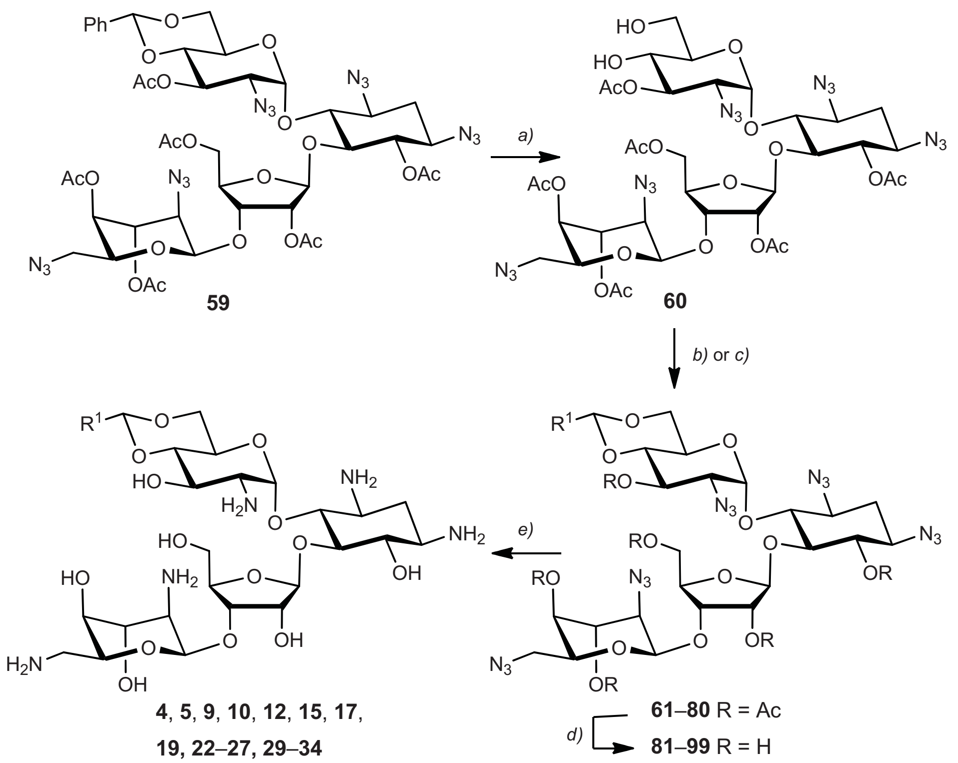

61, 81, **4** R^1 = 4-$Me_2NC_6H_4$
62, 82, **5** R^1 = 4-HOC_6H_4
63, 83, **9** R^1= 4-$CF_3C_6H_4$
64, 84, **10** R^1= 3-ClC_6H_4
65, 85, **12** R^1= 3-HOC_6H_4
66, 86, **15** R^1= 2-$NO_2C_6H_4$

67, 87, **17** R^1= 3,5-$(MeO)_2C_6H_4$
68, 88, **19** R^1= 3-Br,4-OHC_6H_3
69, 89, **22** R^1= 3,4,5-$(MeO)_3C_6H_4$
70, 90, **23** R^1= 2-furyl
71, 91, **24** R^1= 2-thiophenyl
72, 92, **25** R^1= cyclohexyl

73, 93, **26** R^1= 1-naphthyl
74, 94, **27** R^1= 2-naphthyl
75, 95, **29** R^1= benzyl
76, 96, **30** R^1= $(CH_2)_2C_6H_5$
77, 97, **31** R^1= (*E*)-$(CH)_2C_6H_5$
78, 98, **32** R^1= $(CH_2)_2$(4-$OMeC_6H_4$)
79, 99, **33** R^1= $(CH_2)_3C_6H_5$
80, **100**, **34** R^1= $(CH_2)_4C_6H_5$

a) MeOH, TsOH-H_2O, 25°, 2h (77%); *b)* R^1CHO, 0.3–0.5 equiv. $FeCl_3$, 23°; 0.3–24h, *c)* R^1CHO, 0.5 equiv. TsOH-H_2O, toluene, reflux, 2–26h; *d)* NaOMe, CH_2Cl_2/MeOH 1:4, 25°; *or* (**65**) $MeNH_2$, aq. MeOH/EtOH, 0°, 1h *then* 6h, 26° (63%), *or* (**68**) NaOMe, MeOH, 26°, 15h (80 %); *e)* PMe_3, NaOH, aq. THF, 60°; *or* $HS(CH_2)_3SH$, Et_3N, MeOH.

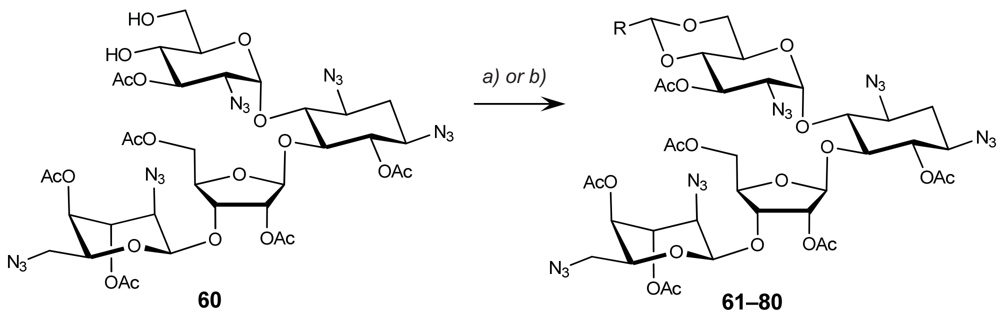

60 **61–80**

a) RCHO (neat), 0.3–0.5 equiv. $FeCl_3$; *b)* 5 equiv. RCHO, 0.5 equiv $TsOH.H_2O$, 5Å MS, toluene, reflux.

Standard deacetylation of the acetals **61–80**, followed by Staudinger reaction/hydrolysis led to the alcohols **81–100** in good yields. Staudinger reaction/hydrolysis of the 4-hydroxybenzylidene acetal **81** did not lead to the desired amine. The 2-aryldioxane ring was cleaved under the reaction conditions. The azido groups were therefore reduced by treating **81** with propane-1,3-dithiol to provide **5**.

The synthesis of the acetals **20**–**21** followed the same protocols, acid catalyzed reaction of **60** with 3-formyl-4-hydroxybenzaldehyde proceeding with the expected regioselectivity on account of the intramolecular hydrogen bond, yielding mostly the salicylaldehyde derived **101** besides small amounts of the isomeric **102**. The salicyliate analog **103** was obtained similarly. Standard deacetylation to **104** and **105** followed by a Staudinger reaction provided **20** and **21**, respectively.

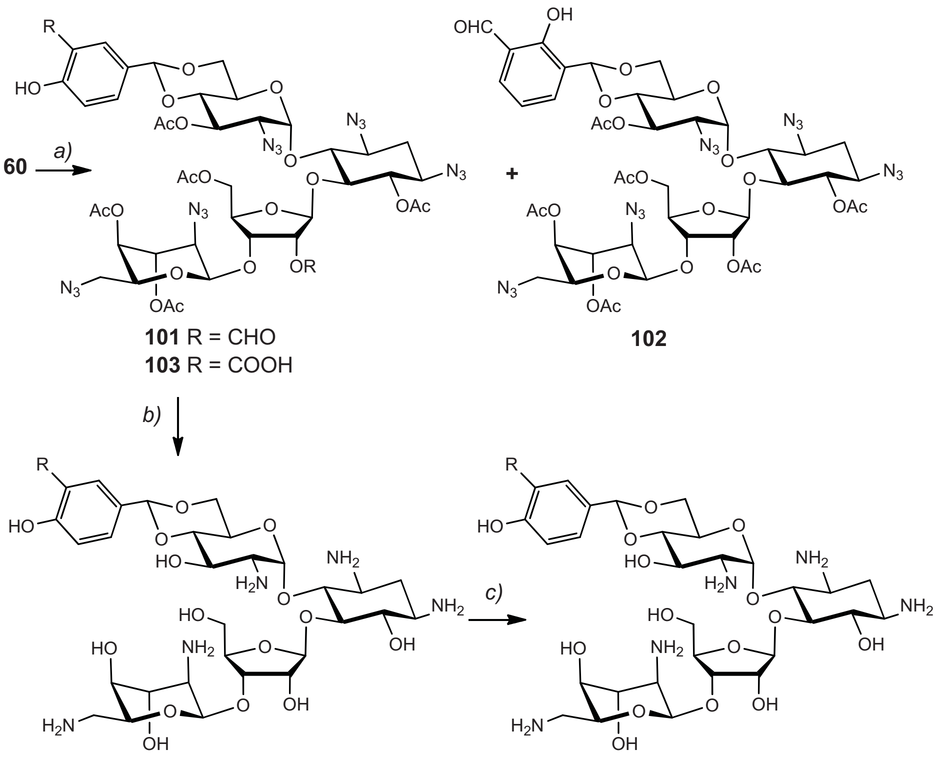

a) 4-OH-3-RC_6H_3CHO, TsOH-H_2O, DMF, 65°, 25 h (55% **101**, 5% **102,** for **103** 60%) b) **101**, K_2CO_3, aq. MeOH, 26°,12h, (73%) or **103**, $MeNH_2$, aq. MeOH/EtOH, 0°, 1h *then* 6h, 26°, (63%), c) HS$(CH_2)_3$SH, Et_3N, MeOH (71% **20**, 68% **21**).

We used the known regioselective reductive opening of 2-phenyl-1,3-dioxane rings with borane-dimethyl sulfide in the presence of dibutyl borinic acid triflate to transform **59** into the 4'-*O*-benzyl ether **106**. Standard deacetylation to **107** and Staudinger reaction afforded the desired benzyl ether **37**.

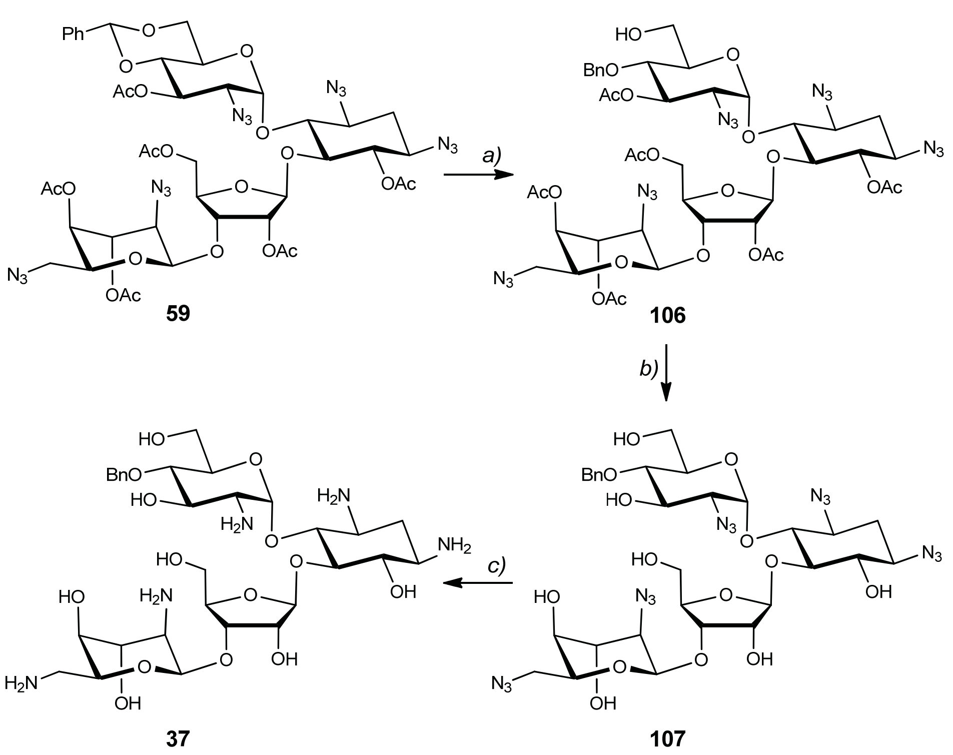

a) 2M $BH_3 \cdot Me_2S$ in THF, 1M Bu_2BOTf in CH_2Cl_2, CH_2Cl_2, –5° (61%). *b*) 0.02N MeONa, MeOH, 26° (86%). *c*) 0.1M aq. NaOH, 1M PMe_3 in THF, THF, 50° (79%).

The isomeric 6’-*O*-benzyl ether **38** was prepared from **46** *via* the 4-methoxybenzyl protected acetal **108**. The known regioselective reductive ring opening of 2-phenyl-1,3-dioxane rings with cyanoborohydride in the presence of hydrochloric acid gave the protected 6’-O-benzyl ether **109** in modest yields. Standard deprotection with dichlorodicyano benzoquinone (DDQ), *in situ* hydrolysis, and Staudinger reaction provided **38** via **110** in good yields.

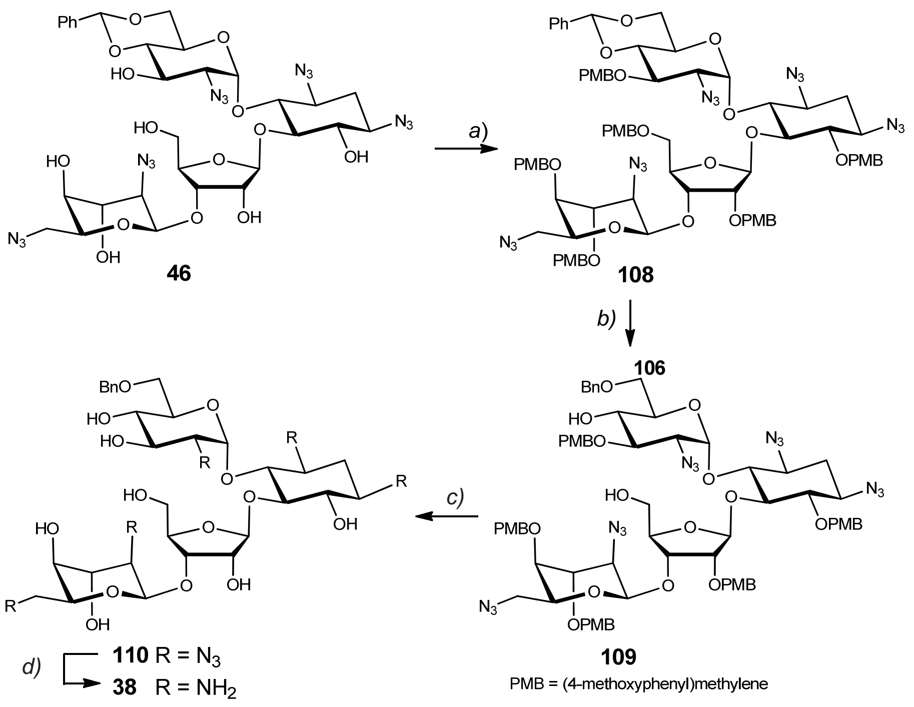

a) NaH, *p*-MeOBnCl, Bu_4NI, THF, 0 to 26° (48%). *b*) $NaCNBH_3$, 0.7M HCl in Et_2O, 4 Å MS, THF, 0° (33%). *c*) DDQ, CH_2Cl_2/H_2O/i-PrOH 20:1:1, 0 to 26° (59%). *d*) 0.1M aq. NaOH, 1M PMe_3 in THF, THF, 50° (85%).

In view of the advantage of the relatively robust methoxybenzyl (as compared to the acetyl) group we synthesized the substituted 4'-*O*-benzyl ethers **39** and **41**–**43** from the methoxybenzyl protected benzylidene acetal **108** by debenzylidenation to **111**, methoxytritylation to **112**, benzylation to **113**–**116**, standard deprotection, and Staudinger reaction, using standard procedures. Synthesis of methoxybenzyl ether **40** required a different approach. We obtained **40** by regioselective reductive ring opening of the 4-methoxybenzylidene acetal **48** with borane-tetrahydrofuran in the presence of dibutyl borinic acid triflate followed by a Staudinger reaction.

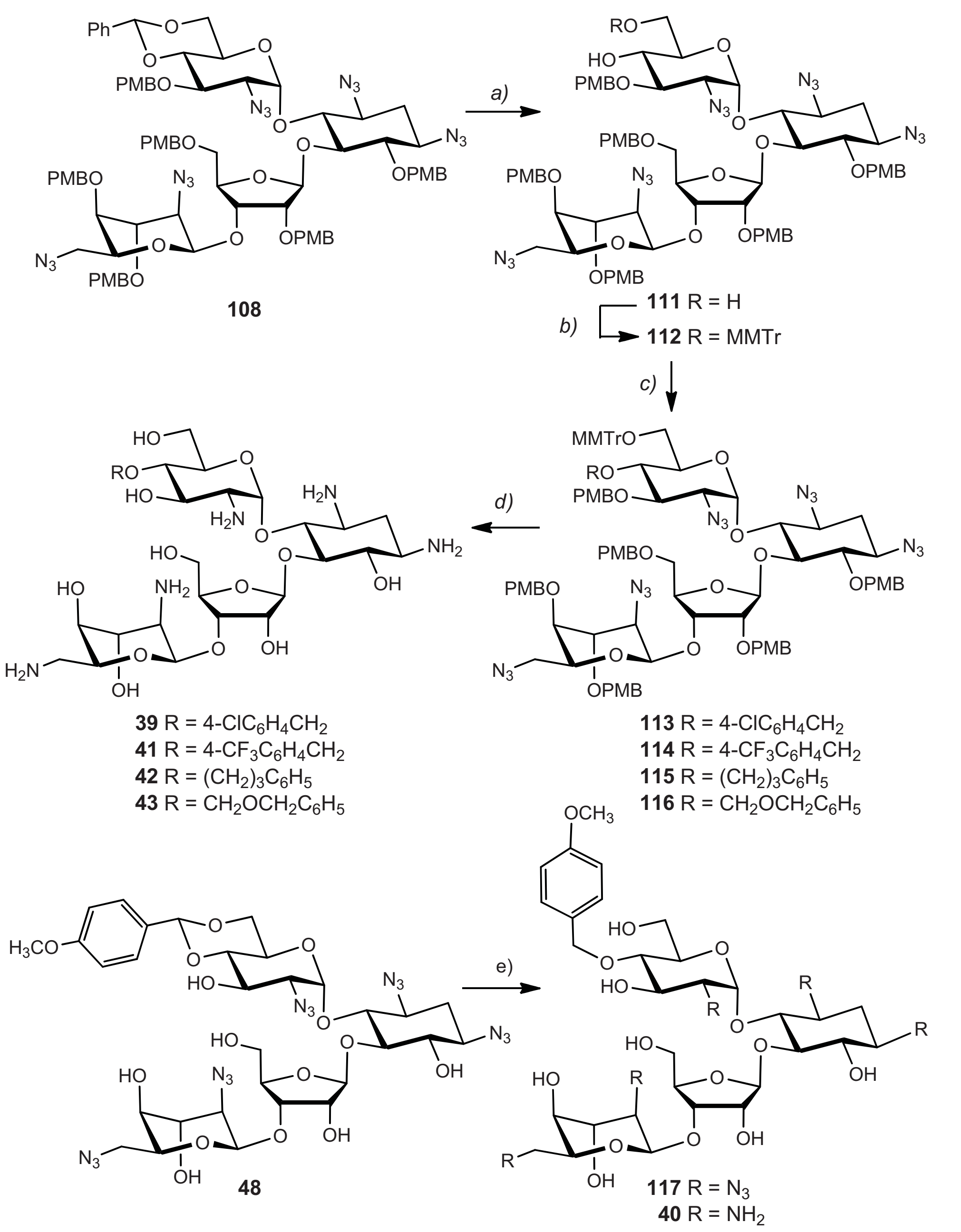

a) TsOH·H_2O, MeOH/CH_2Cl_2 10:1, 25°, 3h (79%); *b)* NaH, DMF, RHal, Bu_4NI (0.1 equiv.), 25°, 4–24h (65% **112**; 96% **113**; 85% **114**; 86% **115**; 83% **116**); *c)* 1. DDQ (1.1 equiv. per PMB and MMTr group), CH_2Cl_2/MeOH/H_2O 20:1:0.4, 25°, 12–24h; 2. 1M PMe_3 in THF (1.2 equiv. per azido group), THF/0.1N NaOH 4:1, 50°, 4–8h (86% **39**, 87% **41·5AcOH**, 92% **42**, 72% **43**), *d)* 1M BH_3·THF in THF and 1M Bu_2BOTf in CH_2Cl_2, -5° to 0°, 50 min. (59%), *e)* 1M PMe_3 in THF (1.2 equiv. per azido group), THF/0.1N NaOH 4:1, 50°, then 10% aq. AcOH (74%).

To obtain the 3’,4’-*seco* acetals **35** and **36** we submitted pentaazide **45** to a regioselective periodate 1,2-diol cleavage, followed by borohydride reduction of the intermediate dialdehyde, acetalation with benzyldehyde and chromatographic separation of the main products to yield acetals **119** (45%) and **120** (30%). Reduction of the azido groups with base catalyzed propane-1,3-dithiol transformed these pentaazides into the diastereoisomeric *seco*-paromomycin acetals **36** and **37**, respectively.

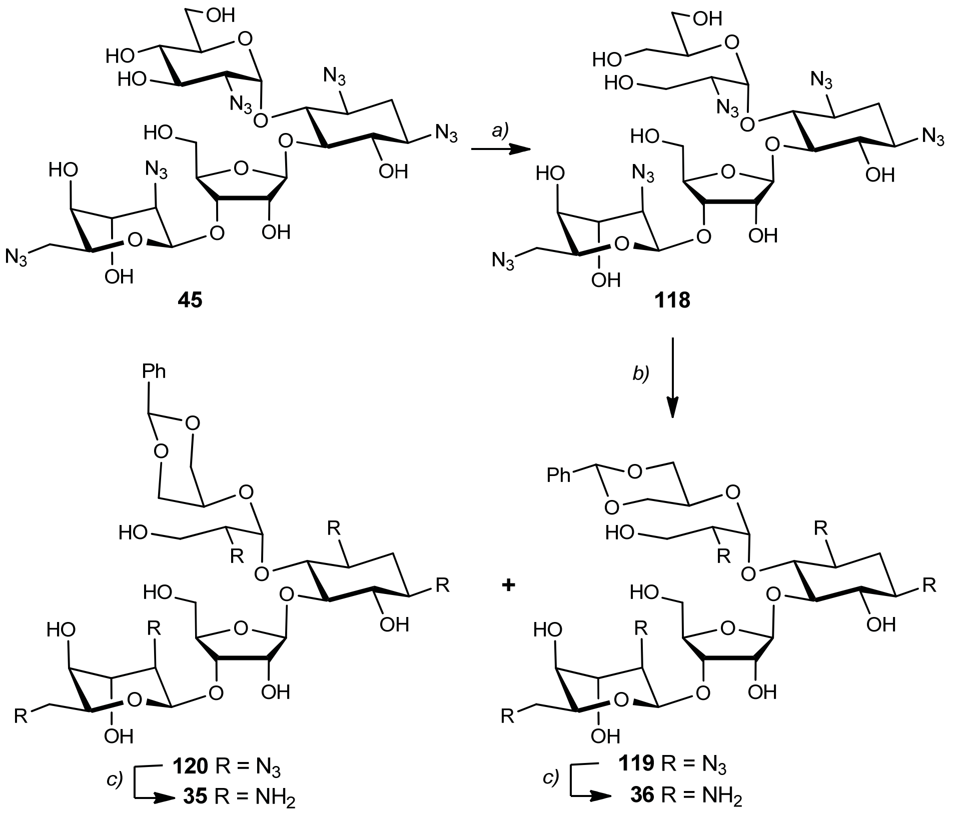

a) 1. 2 equiv. $NaIO_4$, 50% aq. EtOH, 0°, 20h, then $(HOCH_2)_2$; 2. $NaBH_4$, 50% aq. EtOH, 0°, 2h (50%); *b)* PhCHO, HCOOH, 0°, 16h (45% **119**; 30% **120**); *c)* $HS(CH_2)_3SH$, Et_3N, MeOH (60%).

Experimental part

General. THF was distilled from Na and benzophenone, DMF, MeOH, pyridine, Et_3N from CaH_2. Qual. TLC: precoated silica-gel glass plates (*Merck* silica gel *60 F_{254}*); detection by heating with 'mostain' (400 ml of 10% H_2SO_4 soln., 20 g of $(NH_4)_6Mo_7O_{24}.6H_2O$, 0.4 g of $Ce(SO_4)_2$). Flash chromatography (FC): silica gel *Merck*. M.p.: uncorrected. Optical rotations: 1-dm cell at 25°, 589 nm. FT-IR spectra: neat (ATR), absorption in cm^{-1}. ^{1}H- and ^{13}C-NMR spectra: chemical shifts δ in ppm rel. to TMS as external standard, and coupling constants *J* in Hz. ^{1}H-NMR spectral assignments are based on DQFCOSY and HSQC spectra. ^{13}C-NMR spectral assignments are based on HSQC spectra. HR-MALDI-MS: in gentisic acid (= 2,5-dihydroxybenzoic acid, DHB) matrix.

1. Synthesis of the 4',6'-O-Alkylidene-protected Paromomycin Derivatives

1.1. General Transacetalization Procedure for the Formation of Acetals 47-58.

A soln. in DMF of **47**[50] the substituted benzyaldehyde dimethyl acetal (5 equiv.) corresponding to the dioxane moiety of **47–57**, and of $TsOH.H_2O$ (0.5 equiv.) was stirred at 25 or at 65°, diluted with AcOEt, and washed with 0.1M aq. NaOH. The aq. layer was extracted twice with AcOEt. The combined org. layers were washed with brine, dried ($MgSO_4$), filtered, and evaporated. FC (hexane/AcOEt 4:1, $CHCl_3$/AcOEt 1:1 → $CHCl_3$/AcOEt/MeOH 6:6:0.25) gave **47–57**.

*1,3,2',2''',6'''-Pentadeamino-1,3,2',2''',6'''-pentaazido-4',6'-*O*-(4 chlorobenzylidene)paromomycin* (**47**). Reaction of 28 mg of **45** at 26–70° for 36 h and FC gave 10 mg **47** (31%). White solid. R_f ($CHCl_3$/AcOEt/MeOH 3:3:0.5) 0.48. ^{1}H-NMR (500 MHz, CD_3OD): δ 7.48 (*d*, *J* = 8.4, 2 arom. H); 7.35 (*d*, *J* = 8.6, 2 arom. H); 5.83 (d, *J* = 3.9, H–C(1')); 5.59 (*s*, ArC*H*); 5.38 (d, *J* = 2.0 , H–C(1'')); 5.13 (d, *J* = 1.8, H–C(1''')); 4.42 (dd, *J* = 6.6, 4.6, H–C(3'')); 4.31 (dd, *J* = 4.6, 2.0, H–C(2'')); 4.22 (dd, *J* = 10.1, 5.0, H_a–C(6')); 4.17–4.08 (m, H–C(5'), H–C(4'')); 4.10 (t, *J* = 10.0, H–C(3')); 4.02 (ddd, *J* = 8.5, 4.5, 2.0, H–C(5''')); 3.94 (t, *J* = 3.4, H–C(3''')); 3.84 (dd. *J* = 11.9, 2.8, H_a–C(5'')); 3.76 (t, *J* = 10.2,H_b–C(6')); 3.72–3.63 (m, H–C(4), H–C(5), H_b–C(5''), H–C(2'''), H_a–C(6''')); 3.56–3.50 (m, H–C(3)); 3.54 (t, *J* = 9.4, H–C(4')); 3.48–3,36 (m, H-C(1), H–C(6), H–C(4''')); 3.38 (dd, *J* = 12.9, 4.5, H_b–C(6''')); 3.24 (dd, *J* = 10.2, 3.9, H–C(2')); 2.21 (dt, *J* = 12.9, 4.2,

H_{eq}–C(2)); 1.41 (q, J =12.1, H_{ax}–C(2)). ^{13}C-NMR (126 MHz, CD_3OD): δ 137.97 (*s*); 135.80 (*s*); 129.26 (4*d*); 109.78 (d, C)1'')); 102.21 (*d*, Ar*C*H); 99.80 (d, C(1''')); 99.15 (d, C(1')); 85.27 (d, C(5)); 83.47 (d, C(4'')); 82.96 (d, C(4')); 77.79 (d, C(4)); 77.30, 77.28 (2d, C(6), C(3'')); 75.68 (d, C(5''')); 75.13 (d, C(2'')); 71.19 (d, C(3''')) 69.84 (t, C(6')); 69.64 (d, C(4''')); 69.57 (d, C(3')); 65.21 (d, C(2')); 64.52 (d, C(5')); 63.71 (t, C(5'')); 61.91 (d C(2''')); 61.83 (d, C(1)); 61.30 (d, C(3)); 52.52 (t, C(6''')); 33.02 (t, (C(2)). Anal. calc. for $C_{31}H_{38}ClN_{15}O_{14}$.MeOH (900.21): C 41.36, H 4.70, N 23.34; found: C 41.16, H 4.66, N 22.99.

*1,3,2',2''',6'''-Pentadeamino-1,3,2',2''',6'''-pentaazido-4',6'-*O*-(4-methoxybenzylidene)paromomycin* (**48**). Reaction of 71 mg of **45** at 26° for 1.5 h and FC (hexane/AcOEt 9:1) gave 57 mg of **48** (69%). White solid. R_f ($CHCl_3$/AcOEt/MeOH 3:3:0.5) 0.46. $[\alpha]_D^{29}$ = +112.5 (c = 0.29, MeOH). IR (ATR): 3403*w*, 2923*w*, 2099*s*, 1614*w*, 1518*w*, 1453*w*, 1373*w*, 1334*w*, 1302*w*, 1249*m*, 1166*w*, 1142*m*, 1123*m*, 1083*s*, 1023*s*, 992*s*, 925*m*. ^{1}H-NMR (500 MHz, CD_3OD): δ 7.42–7.39 (m, 2 arom. H); 6.90–6.87 (m, 2 arom. H); 5.82 (d, J = 3.9, H–C(1')); 5.54 (s, Ar*CH*); 5.39 (d, J = 1.9, H–C(1'')); 5.13 (d, J = 1.8, H–C(1''')); 4.43 (dd, J = 6.6, 4.6, H–C(3'')); 4.32 (dd, J = 4.9, 1.9, H–C(2'')); 4.20 (dd, J = 10.0, 4.9, H_a–C(6')); 4.16–4.08 (m, H–C(5'), H–C(4'')); 4.10 (t, J = 9.7, H–C(3')); 4.02 (ddd, J = 8.5, 4.6, 2.0, H–C(5''')); 3.93 (t, J = 3.4, H–C(3''')); 3.84 (dd, J = 11.9, 2.8, H_a–C(5'')); 3.78 (s, MeO); 3.74 (t, J = 10.1, H_b–C(6')); 3.72–3.63 (m, H–C(4), H–C(5), H_b–C(5''), H–C(2'''), H_a–C(6''')); 3.56–3.50 (m, H–C(3)); 3.52 (t, J = 9.3, H–C(4')); 3.48–3.41 (m, H–C(1), H–C(6), H–C(4''')); 3.38 (dd, J = 13.0, 4.6, H_b–C(6''')); 3.24 (dd, J = 10.2, 3.9, H–C(2')); 2.22 (dt, J = 12.8, 4.2, H_{eq}–C(2)); 1.41 (q, J = 12.4, H_{ax}–C(2)).^{13}C-NMR (126 MHz, CD_3OD δ 161.69 (s); 131.51 (s); 128.90 (2d); 114.41 (2d); 109.77 (d, C(1'')); 103.16 (d, Ar*C*H); 99.81 (d, C(1''')); 99.17 (d, C(1')); 85.27 (d, C(5)); 83.48 (d, C(4'')); 82.95 (d, C(4')); 77.77 (d, C(4)); 77.29 (2d, C(3''), C(6)); 75.67 (d, C(5''')); 75.13 (d, C(2'')); 71.19 (d, C(3''')); 69.82 (t, C(6')); 69.64 (d, C(4''')); 69.61 (d, C(3')); 65.22 (d, C(2')); 64.60 (d, C(5')); 63.72 (t, C(5'')); 61.92 (d, C(1)); 61.83 (d, C(2''')); 61.32 (d, C(3)); 55.73 (q, MeO); 52.51 (t, C(6''')); 33.03 (t, C(2)). HR-MALDI-MS (*m*/*z*): 902.2569 (22, $[M + K]^+$, $C_{31}H_{41}KN_{15}O_{15}^+$; calc. 902.2544); 887.2817 (39); 886.2783 (100, $[M + Na]^+$, $C_{31}H_{41}N_{15}NaO_{15}^+$; calc. 886.2799). Anal. calc. for $C_{31}H_{41}N_{15}O_{15}$.MeOH (895.79): C 42.91, H 5.06, N 23.45; found: C 43.31, H 5.00, N 23.14.

*1,3,2',2''',6'''-Pentadeamino-1,3,2',2''',6'''-pentaazido-4',6'-*O-*(4-fluorobenzylidene)paromomycin* (**49**). Reaction of 175 mg of **45** at 66° for 1 h 10 and FC gave 139 mg of **49** (69%).White solid. R_f ($CHCl_3$/AcOEt/MeOH 3:3:0.5) 0.34. ^{1}H-NMR (300 MHz, CD_3OD): δ 7.54–7.49 (m, 2 arom. H); 7.10–7.04 (m, 2 arom. H); 5.83 (d, *J* = 3.9, H–C(1')); 5.59 (s, ArC*H*); 5.39 (br. s, H–C(1'')); 5.13 (br. s, H–C(1''')); 4.45–4.41 (m, H–C(3'')); 4.32 (br. d, *J* = 4.7, H–C(2'')); 4.24–4.07 (m, H_a–C(6'), H–C(4''), H–C(5'), H–C(3')); 4.04–3.99 (m, H–C(5''')); 3.93 (t, *J*= 3.3, H–C(3''')); 3.84 (dd, *J* = 11.8, 2.8, H_a–C(5'')); 3.79–3.63 (m, H_b–C(6'), H–C(2'''), H_b–C(5''), H–C(4), H–C(5), H_a–C(6''')); 3.57–3.50 (m, H–C(3)); 3.54 (t, *J* = 9.5, H–C(4')); 3.46–3.35 (m, H–C(4'''), H–C(1), H–C(6)); 3.37 (dd, *J* = 12.9, 4.7, H_b–C(6''')); 3.24 (dd, *J* = 10.2, 3.9, H–C(2')); 2.23–2.16 (m, H_{eq}–C(2)); 1.41 (q, *J* = 12.1, H_{ax}–C(2)). ^{13}C-NMR (75 MHz, CD_3OD):δ 164.49 (d, ^1J(C,F) = 245.6); 135.41 (d, ^4J(C,F) = 3.2); 129.66 (2d, ^3J(C,F) = 7.9); 115.81 (2d, ^2J(C,F) = 22.0); 109.74 (d, C(1'')); 102.37 (d, Ar*C*H); 99.80 (d, C(1''')); 99.15 (d, C(1')); 85.25 (d, C(5)); 83.46 (d, C(4'')); 82.93 (d, C(4')); 77.78 (d, C(4)); 77.28 (2d, C(3''), C(6)); 75.67 (d, C(5''')); 75.13 (d, C(2'')); 71.17 (d, C(3''')); 69.82 (t, C(6')); 69.63, 69.57 (2*d*, C(4'''), C(3')); 65.19 (*d*, C(2')); 64.54 (*d*, C(5')); 63.70 (*t*, C(5'')); 61.90 (*d*, C(1)); 61.82 (d, C(2''')); 61.30 (d, C(3)); 52.51 (t, C(6''')); 33.02 (t, C(2)). ^{19}F-NMR (256 MHz, CD_3OD): δ –113.03 (tt, *J* = 14.9, 9.6, arom. F). HR-MALDI-MS (*m*/*z*): 890.2335 (28, $[M + K]^+$, $C_{30}H_{38}FKN_{15}O_{14}^+$; calc. 890.2344); 875.2620 (38); 874.2582 (100, $[M + Na]^+$, $C_{30}H_{38}FN_{15}NaO_{14}^+$; calc. 874.2599). Anal. calc. for $C_{30}H_{38}FN_{15}O_{14}$.MeOH (883.76): C 42.13, H 4.79, N 23.77; found: C 42.39, H 4.79, N 23.77.

*1,3,2',2''',6'''-Pentadeamino-1,3,2',2''',6'''-pentaazido-4',6'-*O-*(4-cyanobenzylidene)paromomycin* (**51**). Reaction of 115 mg of **45** at 70° for 19 h and FC gave 50 mg of **51** (38%). White solid. R_f ($CHCl_3$/AcOEt/MeOH 3:3:0.5) 0.31. ^{1}H-NMR (300 MHz, CD_3OD): δ 7.76–7.67 (m, 4 arom. H); 5.84 (d, *J* = 3.7, H–C(1')); 5.67 (s, ArC*H*); 5.39 (d, *J* = 1.6, H–C(1'')); 5.13 (d, *J* = 1.6, H–C(1''')); 4.43 (dd, *J* = 6.5, 4.4, H–C(3'')); 4.32 (dd, *J* = 4.4, 1.9, H–C(2'')); 4.25 (dd, *J* = 10.0, 4.9, H_a–C(6')); 4.18–4.08 (m, H–C(4''), H–C(5'), H–C(3')); 4.02 (ddd, *J* = 8.4, 4.4, 1.9, H–C(5''')); 3.94 (t, *J*= 3.1, H–C(3''')); 3.85 (dd, *J* = 12.1, 2.8, H_a–C(5'')); 3.79 (t, *J* = 10.3, H_b–C(6')); 3.73–3.63 (m, H–C(2'''), H_b–C(5''), H–C(4), H–C(5), H_a–C(6''')); 3.58 (t, *J* = 9.3, H–C(4')); 3.58–3.48 (m H–C(3)); 3.47–3.33 (m, H–C(4'''), H–C(1), H–C(6), H_b–C(6''')); 3.26 (dd, *J* = 10.6, 4.0, H–C(2')); 2.21

(dt, J = 12.8, 4.4, H_{eq}–C(2)); 1.41 (q, J = 12.5, H_{ax}–C(2)). ^{13}C-NMR (75 MHz, CD_3OD): δ 144.07 (s); 133.18 (2d); 128.61 (2d); 119.56 (s); 113.79 (s, CN); 113.79 (s); 109.82 (d, C(1”)); 101.58 (d, Ar*C*H)); 99.81 (d, C(1”’)); 99.12 (d, C(1’)); 85.28 (d, C(5)); 83.48 (d, C(4”)); 83.00 (d, C(4’)); 77.78 (d, C(4)); 77.31 (2d, C(3”), C(6)); 75.68 (d, C(5”’)); 75.13 (d, C(2”)); 71.20 (d, C(3”’)); 69.89 (t, C(6’)); 69.65, 69.57 (2d, C(4”’), C(3’)); 65.22 (d, C(2’)); 64.48 (d, C(5’)); 63.72 (t, C(5”)); 61.92 (d, C(1)); 61.83 (d, C(2”’)); 61.30 (d, C(3)); 52.53 (t, C(6”’)); 33.02 (, C(2)). HR-MALDI-MS (*m*/*z*): 858.2722 (39); 857.2675 (100, $[M-H]^-$, $C_{31}H_{37}N_{16}O_{14}^-$; calc. 857.2681). Anal. calc. for $C_{31}H_{38}N_{16}O_{14}$.2 MeOH (922.82): C 42.95, H 5.02, N 24.29; found: C 42.90, H 4.81, N 23.86.

*1,3,2’,2”’,6”’-Pentadeamino-1,3,2’,2”’,6”’-pentaazido-4’,6’-*O*(-3-methoxybenzylidene)-paromomycin* (**52**). Reaction of 101 mg of **45** at 24° for 4 h and FC gave 47 mg of **52** (40%). White solid. R_f ($CHCl_3$/AcOEt/MeOH 3:3:0.5) 0.45. $[\alpha]_D^{29}$ = +98.2 (*c* = 0.17, MeOH). IR (ATR): 3400*w*, 2923*w*, 2872*w*, 2099*s*, 1590*w*, 1492*w*, 1456*w*, 1435*w*, 1372*w*, 1326*w*, 1258*m*, 1140*w*, 1121*w*, 1079*s*, 1032*s*, 994*s*. ^{1}H-NMR (500 MHz, CD_3OD): δ 7.27–7.23 (m, 1 arom. H); 7.07–7.05 (m, 2 arom. H); 6.91–6.88 (m, 1 arom. H); 5.83 (d, J = 3.9, H–C(1’)); 5.56 (s, ArC*H*); 5.39 (d, J = 1.9, H–C(1”)); 5.14 (d, J = 1.8, H–C(1”’)); 4.43 (dd, J = 6.6, 4.6, H–C(3”)); 4.32 (dd, J = 4.6, 1.9, H–C(2”)); 4.22 (dd, J = 9.9, 4.9, H_a–C(6’)); 4.17–4.11 (m, H–C(5’), H–C(4”)); 4.11 (t, J = 9.6, H–C(3’)); 4.02 (ddd, J = 8.4, 4.5, 2.0, H–C(5”’)); 3.94 (t, J= 3.4, H–C(3”’)); 3.84 (dd, J = 12.1, 2.8, H_a–C(5”)); 3.81–3.64 (m, H_b–C(6’), H–C(2”’), H–C(4), H–C(5), H_b–C(5”), H_a–C(6”’)); 3.78 (s, MeO); 3.56–3.51 (m, H–C(3)); 3.54 (t, J = 9.5, H–C(4’)); 3.47–3.42 (m, H–C(1), H–C(6), H–C(4”’)); 3.39 (dd, J = 12.9, 4.5, H_b–C(6”’)); 3.25 (dd, J = 10.2, 3.9, H–C(2’)); 2.22 (dt, J = 12.9, 4.2, H_{eq}–C(2)); 1.41 (q, J = 12.4, H_{ax}–C(2)). ^{13}C-NMR (126 MHz, CD_3OD): δ 161.04 (s); 140.59 (s); 130.17 (d); 119.93 (d); 115.80 (d); 112.92 (d); 109.80 (d, C(1”)); 102.92 (d, Ar*C*H); 99.84 (d, C(1”’)); 99.19 (d, C(1’)); 85.30 (d, C(5)); 83.51 (d, C(4”)); 83.00 (d, C(4’)); 77.81 (d, C(4)); 77.35, 77.31 (2d, C(6), C(3”)); 75.68 (d, C(5”’)); 75.19 (d, C(2”)); 71.20 (d, C(3”’)); 69.85 (t, C(6’)); 69.68 (d, C(4”’)); 69.62 (d, C(3’)); 65.24 (d, C(2’)); 64.60 (d, C(5’)); 63.73 (t, C(5”)); 61.93 (d, C(1)); 61.88 (d, C(2”’)); 61.36 (d, C(3)); 55.74 (q, MeO); 52.53 (t, C(6”’)); 33.06 (t, C(2)). HR-MALDI-MS (*m*/*z*): 863.2932 (33); 862.2849 (88, $[M-H]^-$, $C_{31}H_{40}N_{15}O_{15}^-$; calc. 862.2834). Anal. calc. for $C_{31}H_{41}N_{15}O_{15}.CH_3CO_2CH_2CH_3$ (951.85): C 44.16, H 5.19, N 22.07; found: C 43.83, H 5.32, N 21.71.

*1,3,2',2''',6'''-Pentadeamino-1,3,2',2''',6'''-pentaazido-4',6'-*O*-(2-methoxybenzylidene)paromomycin* (**54**). Reaction of 99 mg of **45** at 24° for 2.5 h and FC gave 76 mg of **54** (66%). White solid. R_f ($CHCl_3$/AcOEt/MeOH 3:3:0.5) 0.40. $[\alpha]_D^{29}$ = +120.6 (*c* = 0.28, MeOH). IR (ATR): 3396*w*, 2936*w*, 2098*s*, 1686*w*, 1606*w*, 1590*w*, 1497*w*, 1464*w*, 1441*w*, 1376*w*, 1330*w*, 1250*m*, 1140*m*, 1117*w*, 1081*s*, 1022*s*, 988*s*. ^{1}H-NMR (500 MHz, CD_3OD): δ 7.55–7.53 (m, arom. H); 7.34–7.30 m, 1 arom. H); 6.98–6.92 (m, 2 arom. H); 5.93 (s, ArC*H*); 5.83 (d, *J* = 3.9, H–C(1')); 5.39 (d, *J* = 1.9, H–C(1'')); 5.13 (d, *J* = 1.8, H–C(1''')); 4.43 (dd, *J* = 6.6, 4.6, H–C(3'')); 4.32 (dd, *J* = 4.5, 2.0, H–C(2'')); 4.18 (dd, *J* = 9.7, 5.0, H_a–C(6')); 4.18–4.13 (m, H–C(5'), H–C(4'')); 4.10 (t, *J* = 9.7, H–C(3')); 4.02 (ddd, *J* = 8.4, 4.5, 1.9, H–C(5''')); 3.93 (t, *J* = 3.4, H–C(3''')); 3.84 (dd, *J* = 12.3, 2.8, H_a–C(5'')); 3.83 (s, MeO); 3.73–3.64 (m, H_b–C(6'), H–C(2'''), H_b–C(5''), H–C(4), H–C(5), H_a–C(6''')); 3.57–3.52 (m, H–C(3)); 3.54 (t, *J* = 9.5, H–C(4')); 3.47–3.42 (m, H–C(1), H–C(6), H–C(4''')); 3.40 (dd, *J* = 12.9, 4.6, H_b–C(6''')); 3.24 (dd, *J* = 10.2, 3.9, H–C(2')); 2.22 (dt, *J* = 12.8, 4.2, H_{eq}–C(2)); 1.41 (q, *J* = 12.5, H_{ax}–C(2)). ^{13}C-NMR (126 MHz, CD_3OD): δ 158.18 (s); 131.45 (d); 128.53 (d); 127.15 (s); 121.42 (d); 111.92 (d); 109.77 (d, C(1'')); 99.82 (d, C(1''')); 99.17 (d, C(1')); 98.46 (d, Ar*C*H); 85.28 (d, C(5)); 83.49 (d, C(4'')); 83.24 (d, C(4')); 77.79 (d, C(4)); 77.30 (2d, C(3''), C(6)); 75.67 (d, C(5''')); 75.14 (d, C(2'')); 71.19 (d, C(3''')); 70.02 (t, C(6')); 69.65 (2d, C(4'''), C(3')); 65.20 (d, C(2')); 64.69 (d, C(5')); 63.72 (t, C(5'')); 61.93 (d, C(1)); 61.84 (d, C(2''')); 61.39 (d, C(3)); 56.18 (q, MeO); 52.51 (t, C(6''')); 33.08 (t, C(2)). HR-MALDI-MS (*m*/*z*): 887.2812 (33); 886.2784 (100, $[M + Na]^+$, $C_{31}H_{41}N_{15}NaO_{15}^+$; calc. 886.2799). Anal. calc. for $C_{31}H_{41}N_{15}O_{15}$.MeOH (895.79): C 42.91, H 5.06, N 23.45; found: C 43.01, H 5.23, N 23.37.

*1,3,2',2''',6'''-Pentadeamino-1,3,2',2''',6'''-pentaazido-4',6'-*O*-(2,5-dimethoxybenzylidene)paromomycin* (**56**). Reaction of 119 mg of **45** at 60° for 2 h 15 and FC gave 104 mg of **56** (73%). White solid. R_f ($CHCl_3$/AcOEt/MeOH 3:3:0.5) 0.54. $[\alpha]_D^{29}$ = +105.0 (*c* = 0.295, MeOH). IR (ATR): 3403*w*, 2935*w*, 2099*s*, 1720*w*, 1655*w*, 1504*m*, 1464*w*, 1421*w*, 1375*w*, 1328*w*, 1258*m*, 1222*m*, 1160*w*, 1140*w*, 1123*w*, 1079*m*, 1023*s*, 990*s*. ^{1}H-NMR (500 MHz, CD_3OD): δ 7.12–7.11 (m, arom. H); 6.92–6.87 (m, 2 arom. H); 5.89 (s, ArCH); 5.83 (d, *J* = 3.9, H–C(1')); 5.39 (d, *J* = 2.0, H–C(1'')); 5.14 (d, *J* = 1.8, H–C(1''')); 4.43 (dd, *J* = 6.5, 4.6, H–C(3'')); 4.32 (dd, *J* = 4.6, 2.0, H–

C(2”)); 4.20–4.13 (m, H_a–C(6’), H–C(4”), H–C(5’)); 4.11 (t, *J* = 9.7, H–C(3’)); 4.02 (ddd, *J* = 8.4, 4.5, 2.0, H–C(5’”)); 3.94 (t, J= 3.4, H–C(3’”)); 3.84 (dd, J = 11.9, 2.7, H_a–C(5”)); 3.79 (s, o-MeO); 3.75–3.64 (m, H_b–C(6’), H–C(2’”), H_b–C(5”), H–C(4), H–C(5), H_a–C(6’”)); 3.73 (s, m-MeO); 3.59–3.51 (m, H–C(3)); 3.53 (t, *J* = 9.3, H–C(4’)); 3.48–3.40 (m, H–C(4’”), H–C(1), H–C(6)); 3.39 (dd, *J* = 12.9, 4.6, H_b–C(6’”)); 3.24 (dd, *J* = 10.2, 3.9, H–C(2’)); 2.22 (dt, *J* = 12.9, 4.3, H_{eq}–C(2)); 1.41 (q, *J* = 12.4, H_{ax}–C(2))^{13}C-NMR (126 MHz, CD_3OD): δ 155.11 (s); 152.42 (s); 128.12 (d); 116.59 (s); 113.96 (d); 113.61 (d); 109.81 (d, C(1”)); 99.85 (d, C(1’”)); 99.19 (d, C(1’)); 98.31 (d, ArCH); 85.31 (d, C(5)); 83.52 (d, C(4”)); 83.20 (d, C(4’)); 77.83 (d, C(4)); 77.36, 77.30 (2d, C(3”), C(6)); 75.67 (d, C(5’”)); 75.20 (d, C(2”)); 71.20 (d, C(3’”)); 70.00 (t, C(6’)); 69.68, 69.66 (2d, C(4’”), C(3’)); 65.20 (d, C(2’)); 64.69 (d, C(5’)); 63.73 (t, C(5”)); 61.92 (d, C(1)); 61.88 (d, C(2’”)); 61.47 (d, C(3)); 57.02, 56.16 (2q, 2 MeO); 52.53 (t, C(6’”)); 33.13 (t, C(2)).HR-MALDI-MS (*m*/*z*): 917.2917 (40); 916.2887 (100, [*M* + Na]+, $C_{32}H_{43}N_{15}NaO_{16}^+$; calc. 916.2904). Anal. calc. for $C_{32}H_{43}N_{15}O_{16}$.MeOH (925.82): C 42.81, H 5.12, N 22.69; found: C 42.93, H 5.34, N 22.34.

*1,3,2’,2’”,6’”-Pentadeamino-1,3,2’,2’”,6’”-pentaazido-4’,6’-*O*-(4-phenylbenzylidene)paromomycin* (**57**). Reaction of 80 mg of **45** at 50° for 1.5 h and FC gave 66 mg of **57** (68%). White solid. R_f ($CHCl_3$/AcOEt/MeOH 3:3:0.5) 0.42. M.p. 98° (softening)–123° (dec.). $[\alpha]_D^{29}$ = +102.6 (*c* = 0.29, MeOH). IR (ATR): 3400*w*, 2924*w*, 2872*w*, 2098*s*, 1655*w*, 1615*w*, 1599*w*, 1488*w*, 1449*w*, 1373*w*, 1330*w*, 1257*m*, 1140*m*, 1121*m*, 1084*m*, 1025*s*, 925*m*. ^{1}H-NMR (500 MHz, CD_3OD): δ 7.62–7.57 (m, 6 arom. H); 7.44–7.41 (m, 2 arom. H); 7.35–7.31 (m, 1 arom. H); 5.84 (d, *J* = 3.9, H–C(1’)); 5.65 (s, ArCH); 5.40 (d, *J* = 1.9, H–C(1”)); 5.14 (d, *J* = 1.9, H–C(1’”)); 4.44 (dd, *J* = 6.6, 4.6, H–C(3”)); 4.33 (dd, *J* = 4.5, 1.9, H–C(2”)); 4.24 (dd, *J* = 10.0, 5.0, H_a–C(6’)); 4.19–4.11 (m, H–C(4”), H–C(5’), H–C(3’)); 4.03 (ddd, *J* = 8.4, 4.5, 1.9, H–C(5’”)); 3.94 (t, *J* = 3.4, H–C(3’”)); 3.85 (dd, *J* = 12.0, 2.8, H_a–C(5”)); 3.79 (t, *J* = 10.1, H_b–C(6’)); 3.73–3.65 (m, H–C(2’”), H_b–C(5”), H–C(4), H–C(5), H_a–C(6’”)); 3.57 (t, *J* = 9.4, H–C(4’)); 3.57–3.52 (m, H–C(3)); 3.49–3.42 (m, H–C(4’”), H–C(1), H–C(6)); 3.39 (dd, *J* = 12.9, 4.5, H_b–C(6’”)); 3.26 (dd, *J* = 10.2, 3.9, H–C(2’)); 2.22 (dt, *J* = 12.8, 4.3, H_{eq}–C(2)); 1.41 (q, *J* = 12.5, H_{ax}–C(2)). ^{13}C-NMR (126 MHz, CD_3OD): δ 143.17 (s); 142.04 (s); 138.18 (s); 129.92, 128.56, 128.12, 128.07, 127.70 (9d); 109.78 (d, C(1”)); 102.97 (d, ArCH)); 99.81 (d, C(1’”)), 99.18 (d, C(1’)); 85.28 (d, C(5)); 83.48 (d, C(4”)); 83.03 (d, C(4’)); 77.79 (d, C(4));

77.30 (2d, C(3”), C(6)); 75.68 (d, C(5”’)); 75.14 (d, C(2”)); 71.19 (d, C(3”’)); 70.02 (t, C(6’)); 69.65, 69.63 (2d, C(4”’), C(3’)); 65.25 (d, C(2’)); 64.63 (d, C(5’)); 63.72 (t, C(5”)); 61.93 (d, C(1)); 61.84 (d, C(2”’)); 61.32 (d, C(3)); 52.52 (t, C(6”’)); 33.04 (t, C(2)). HR-MALDI-MS (*m*/*z*): 909.3147 (40); 908.3058 (100, $[M-H]^-$, $C_{36}H_{42}N_{15}O_{14}^-$; calc. 908.3041). Anal. calc. for $C_{36}H_{43}N_{15}O_{14}$.MeOH (941.34): C 47.18, H 5.03, N 22.31; found: C 47.26, H 5.11, N 22.39.

1.2. Synthesis of Acetals 61–80 and 101-103.

General Acetalization Procedure A. Under N_2, a soln. of **60** in the aldehyde was treated with $FeCl_3$ (0.3–0.5 equiv.). The mixture was stirred for 20 min–24 h at 23°, diluted with AcOEt, filtered over Celite, and washed with 0.1M aq. NaOH. The aq. layer was extracted twice with AcOEt. The combined org. layers were washed with brine, dried ($MgSO_4$), filtered, and evaporated, followed by isolation of the acetal by FC (hexane/AcOEt or cyclohexane/AcOEt 8:2 → 6:4).

General Acetalization Procedure B. Under N_2, a soln. of **60** in toluene (~ 0.01M) was treated with 5 Å molecular sieves, TsOH.H_2O (0.5 equiv.), and the aldehyde (5 equiv.). The mixture was stirred for 2–26 h under reflux, diluted with AcOEt, filtered through *Celite*, and washed with 0.1M aq. NaOH. The aq. layer was extracted twice with AcOEt. The combined org. layers were washed with brine, dried ($MgSO_4$), filtered, and evaporated, followed by FC (hexane/AcOEt or cyclohexane/AcOEt 8:2 6:4).

6,3’,2”,5”,3”’,4”’-Hexa-O-*acetyl-1,3,2’,2”’,6”’-pentadeamino-1,3,2’,2”’,6”’-pentaazido paromomycin* (**60**). A soln. of **59**[50] (747 mg, 0.688 mmol) in MeOH (15 ml) was treated with TsOH.H_2O (131 mg, 0.688 mmol) and stirred at 25° for 2 h. The mixture was diluted with AcOEt (50 ml) and $NaHCO_3$ (35 ml). The aq. layer was extracted twice with AcOEt (2 x 50 ml). The combined org. layers were washed with brine (70 ml), dried ($MgSO_4$), filtered, and evaporated. FC ($CHCl_3$/AcOEt 3:2 → $CHCl_3$/AcOEt 1:1) gave 61 (530 mg, 77%). White solid. R_f ($CHCl_3$/AcOEt/MeOH 3:3:0.5) 0.57. ^{1}H-NMR (500 MHz, $CDCl_3$): δ 5.90 (d, *J* = 3.7, H–C(1’)); 5.34 (d, *J* = 2.2, H–C(1”)); 5.32 (dd, *J* = 10.5, 9.2, H–C(3’)); 5.03 (t, *J* = 2.8, H–C(3”’)); 4.95–4.91 (m, H–C(6), H–C(2”)); 4.88 (d, *J* = 1.8, H–C(1”’)); 4.71–4.70 (m, H–C(4”’)); 4.45–4.40 (m, H_a–C(5”), H–C(3”)); 4.33–4.30 (m, H–C(4”)); 4.22 (dd, *J* = 12.1, 5.6, H_b–C(5”)); 4.10 (ddd, *J* = 8.2, 4.1, 1.7, H–

C(5’’’)); 4.02 (dt, *J* = 9.8, 3.4, H–C(5’)); 3.91–3.83 (m, H–C(5), H_2–C(6’)); 3.74–3.68 (m, H–C(4), H–C(4’)); 3.60 (dd, *J* = 13.0, 8.2, H_a–C(6’’’); 3.53–3.48 (m, H–C(3)); 3.46–3.41 (m, H–C(1)); 3.32 (t, *J* = 2.0, H–C(2’’’)); 3.25 (dd, *J* = 13.1, 4.2 , H_b–C(6’’’)); 3.11 (dd, *J* = 10.6, 3.7, H–C(2’)); 2.63 (d, *J* = 5.4, HO–C(4’)); 2.34 (dt, *J* = 13.3, 4.7, H_{eq}–C(2)); 2.18, 2.17, 2.163, 2.157, 2.11, 2.10 (6s, 6 AcO); 2.15–2.05 (m, HO–C(6’)); 1.58 (q, *J* = 12.6, H_{ax}–C(2)). ^{13}C-NMR (126 MHz, $CDCl_3$): δ 171.69, 170.92, 170.13, 169.78, 169.68, 168.54 (6*s*, 6 CH_3*C*O); 106.95 (d, C(1’’)); 99.14 (d, C(1’’’)); 96.05 (d, C(1’)); 81.83 (d, C(5)); 79.30 (d, C(4’’)); 75.98, 75.95 (2d, C(4), C(3’’)); 75.29 (d, C(2’’)); 74.63 (d, C(6)); 73.63 (d, C(5’’’)); 73.28 (d, C(3’)); 72.32 (d, C(5’)); 69.35 (d, C(4’)); 68.70 (d, C(3’’’)); 65.71 (d, C(4’’’)); 64.01 (t, C(5’’)); 61.76 (t, C(6’)); 60.73 (d, C(2’)); 59.37 (d, C(3)); 58.01 (d, C(1)); 56.47 (d, C(2’’’)); 50.66 (t, C(6’’’)); 31.41 (t, C(2)); 20.94, 20.89, 20.86, 20.79, 20.69, 20.56 (6q, 6 Me). HR-MALDI-MS (*m/z*): 1036.2737 (38, $[M + K]^+$, $C_{35}H_{47}KN_{15}O_{20}^+$; calc. 1036.2759); 1021.3050 (45); 1020.2995 (32, $[M + Na]^+$, $C_{35}H_{47}N_{15}NaO_{20}^+$; calc. 1020.3019).

*6,3’,2’’,5’’,3’’’,4’’’-Hexa-*O*-acetyl-1,3,2’,2’’’,6’’’-pentadeamino-1,3,2’,2’’’,6’’’-pentaazido-4’,6’-(4-hydroxybenzylidene)paromomycin* (**62**). According to **B**, reaction of 154 mg of **60** for 2.5 h and FC (hexane/AcOEt 4:1 → 55:45) gave 106 mg of **62** (62%). White solid. R_f (hexane/AcOEt 1:1) 0.25. ^{1}H-NMR (300 MHz, $CDCl_3$):): δ 7.30 (d, *J* = 8.4, 2 arom. H); 6.78 (d, *J* = 8.6, 2 arom. H); 5.84 (d, *J* = 3.8, H–C(1’)), 5.56 (t, *J* = 10.0, H–C(3’)); 5.50 (s); 5.43 (s); 5.35 (s, H–C(1’’)); 5.03–4.88 (m, H–C(6), H–C(2’’), H–C(1’’’), H–C(3’’’)); 4.70 (br. s, H–C(4’’’)); 4.46–4.19 (m, H–C(5’), H_a–C(6’), H–C(3’’), H–C(4’’), H_a–C(5’’), H_b–C(5’’)); 4.12–4.08 (m, H–C(5’’’)); 3.89 (t, *J* = 8.7, H–C(5)); 3.72–3.32 (m, H–C(1), H–C(3), H–C(4), H–C(4’), H_b–C(6’), H_a–C(6’’’)); 3.32 (br. s, H–C(2’’’)); 3.25 (dd, *J* = 13.1, 4.2, H_b–C(6’’’)); 3.07 (dd, *J* = 10.4, 3.8, H–C(2’)); 2.37 (dt, *J* = 13.2, 4.6, H_{eq}–C(2)); 2.170, 2.165, 2.15, 2.11, 2.10 (5s, 6 AcO); 1.58 (q, *J* = 12.7, H_{ax}–C(2)). ^{13}C-NMR (75 MHz, $CDCl_3$): δ 170.88, 170.20, 169.82, 169.72, 168.56 (5s, 6 C=O); 156.45 (s); 129.47 (s); 127.86 (d, 2 C); 115.073 (d, 2 C); 107.14 (d, C(1’’)); 101.64 (d, ArCH); 99.10 (d, C(1’’’)); 97.61 (d, C(1’)); 82.22 (d, C(5)); 79.23 (d, C(4’’)); 78.93 (d, C(4’)); 77.23 (d, C(4)); 75.78 (d, C(3’’)); 75.38 (d, C(6)); 74.77 (d, C(2’’)); 73.59 (d, C(5’’’)); 69.10 (d, C(3’)); 68.73, 68.64 (d and t, C(3’’’), C(6’)); 65.74 (d, C(4’’’)); 63.67 (t, C(5’’)); 63.31 (d, C(5’)); 61.57 (d, C(2’)); 58.98 (d, C(3)); 58.03 (d, C(1)); 56.48 (d, C(2’’’)); 50.66 (t, C(6’’’)); 31.38 (t, C(2)); 30.89, 20.80, 20.68, 20.56 (4q, 6 Me). HR-MALDI-MS (*m/z*): 1140.3030 (41,

$[M + K]^+$, $C_{42}H_{51}KN_{15}O_{21}^+$; calc. 1140.3021), 1124.3278 (100, $[M + Na]^+$, $C_{42}H_{51}N_{15}NaO_{21}^+$; calc. 1124.3282).

*6,3',2",5",3"',4"'-Hexa-*O*-acetyl-1,3,2',2"',6"'-pentadeamino-1,3,2',2"',6"'-pentaazido-4',6'-*O*-[(4-trifluoromethyl)benzylidene]paromomycin* (**63**). According to **B**, reaction of 89 mg of **62** for 19 h and FC gave 75 mg of **63** (73%). White solid. R_f (hexane/AcOEt 1:1) 0.58. M.p. 71° (softening)–115°. $[\alpha]_D^{25}$ = +98.2 (*c* = 0.15, $CHCl_3$). IR (ATR): 2949*w*, 2876*w*, 2101*s*, 1743*s*, 1431*w*, 1371*m*, 1324*m*, 1217*s*, 1165*m*, 1123*m*, 1093*m*, 1063*s*, 1030*s*. ^{1}H-NMR (300 MHz, $CDCl_3$): δ 7.64–7.54 (m, 4 arom. H); 5.86 (d, *J* = 3.9, H–C(1')); 5.57 (t, *J* = 10.2, H–C(3')); 5.53 (s, ArCH); 5.35 (d, *J* = 1.9, H–C(1")); 5.03 (t, *J* = 2.5, H–C(3"')); 4.93 (t, *J* = 9.6, H–C(6)); 4.92–4.89 (m, H–C(2"), H–C(1"')); 4.70 (br. s, H–C(4"')); 4.46–4.40 (m, H_a–C(5"), H–C(3")); 4.32–4.19 (m, H–C(5'), H–C(4"), H_a–C(6'), H_b–C(5")); 4.13–4.10 (m, H–C(5"')); 3.90 (t, *J* = 8.8, H–C(5)); 3.73 (t, *J* = 11.8, H_b–C(6')); 3.70 (m, H–C(4), H–C(4'), H_a–C(6"'); 3.54–3.39 (m, H–C(3), H–C(1)); 3.32 (br. s, H–C(2"')); 3.25 (dd, *J* = 13.2, 4.1 , H_b–C(6"')); 3.10 (dd, *J* = 10.7, 3.8, H–C(2')); 2.39 (dt, *J* = 13.2, 4.7, H_{eq}–C(2)); 2.17, 2.16. 2.14, 2.12, 2.10 (5s, 6 AcO); 1.60 (q, *J* = 12.9, Hax–C(2)) . ^{13}C-NMR (75 MHz, $CDCl_3$): δ 170.75, 170.16, 169.97, 169.79, 169.68, 168.52 (6s, 6 C=O); 140.51 (s); 126.78 (2d), 125.28, 126.23 (2d and q, ^{2}J(C,F) = 5.1); 107.19 (d, C(1")); 100.61 (d, ArCH); 99.12 (d, C(1"')); 97.61 (d, C(1')); 82.26 (d, C(5)); 79.28 (d, C(4")); 79.05 (d, C(4')); 75.80 (d, C(3")); 75.37 (d, C(6)); 74.76 (d, C(2")); 73.65 (d, C(5"')); 68.96 (d, C(3')); 68.75 (d, C(3"'), t, C(6')); 65.75 (d, C(4"')); 63.62 (t, C(5")); 63.16 (d, C(5')); 61.52 (d, C(2')); 58.99 (d, C(3)); 58.06 (d, C(1)); 56.49 (d, C(2"')); 50.70 (t, C(6"')); 31.42 (t, C(2)); 20.91, 20.84, 20.70, 20.58 (4q, 6 Me). d of C(4) and q of CF_3 hidden by noise. ^{19}F-NMR (256 MHz, $CDCl_3$): -62.70 (s, CF_3). HR-MALDI-MS (*m/z*): 1192.2894 (50, $[M + K]^+$, $C_{43}H_{50}F_3KN_{15}O_{20}^+$; calc. 1192.2946); 1177.3200 (52); 1176.3182 (100, $[M + Na]^+$, $C_{43}H_{50}F_3N_{15}NaO_{20}^+$; calc. 1176.3201). Anal. calc. for $C_{43}H_{50}F_3N_{15}O_{20}$.0.5 AcOEt (1197.99): C 45.12, H 4.54, N 17.54; found: C 44.90, H 4.63, N 17.29.

*6,3',2",5",3"',4"'-Hexa-*O*-acetyl-1,3,2',2"',6"'-pentadeamino-1,3,2',2"',6"'-pentaazido-4',6'-*O*-(3-chlorobenzylidene)paromomycin* (**64**). According to **A**, reaction of 153 mg of **60** for 20 min and FC gave 104 mg of **64** (61%). White solid. R_f (hexane/AcOEt 1:1) 0.60. M.p. 76 (softening)–95°.

$[\alpha]_D^{25}$ = +99.4 (*c* = 0.20, $CHCl_3$). IR (ATR): 2920*w*, 2851*w*, 2100*s*, 1741*s*, 1643*w*, 1577*w*, 1538*w*, 1434*w*, 1370*m*, 1332*w*, 1213*s*, 1174*w*, 1123*m*, 1087*m*, 1030*s*, 937*w*. ^{1}H-NMR (300 MHz, $CDCl_3$): δ 7.43–7.28 (m, 4 arom. H); 5.84 (d, *J* = 3.8, H–C(1')); 5.60 (t, *J* = 10.2, H–C(3')); 5.46 (s, ArCH); 5.34 (d, *J* = 2.2, H–C(1")); 5.03 (t, *J* = 3.0, H–C(3"')); 4.93 (t, *J* = 9.9, H–C(6)); 4.93–4.89 (m, H–C(2")); 4.88 (d, *J* = 1.9, H–C(1"')); 4.70 (t, *J* = 1.9, H–C(4"')); 4.46–4.38 (m, H_a–C(5"), H–C(3")); 4.32–4.19 (m, H–C(5'), H–C(4"), H_a–C(6'), H_b–C(5")); 4.11 (ddd, *J* = 8.5, 4.1, 1.7, H–C(5"')); 3.90 (t, *J* = 8.8, H–C(5)); 3.73–3.55 (m, H_b–C(6'), H–C(4), H–C(4'), H_a–C(6"'); 3.54–3.38 (m, H–C(3), H–C(1)); 3.32 (t, *J* = 2.2, H–C(2"')); 3.25 (dd, *J* = 12.9, 3.9, H_b–C(6"')); 3.08 (dd, *J* = 10.4, 3.9, H–C(2')); 2.37 (dt, *J* = 13.2, 4.7, H_{eq}–C(2)); 2.173, 2.169, 2.158, 2.14, 2.12, 2.10 (6s, 6 AcO); 1.61 (q, *J* = 12.6, H_{ax}–C(2)). ^{13}C-NMR (75 MHz, $CDCl_3$): δ 170.77, 170.16, 170.02, 169.80, 169.70, 168.54 (6s, 6 C=O); 138.75 (s); 134.17 (s); 129.59 (d); 129.29 (d); 126.66 (d); 124.48 (d); 107.15 (d, C(1")); 100.71 (d, ArCH); 99.10 (d, C(1"')); 97.65 (d, C(1')); 82.23 (d, C(5)); 79.23 (d, C(4")); 78.97 (d, C(4')); 77.23 (d, C(4)); 75.77 (d, C(3")); 75.35 (d, C(6)); 74.74 (d, C(2")); 73.62 (d, C(5"')); 68.91 (d, C(3')); 68.71 (d, C(3"'), t, C(6')); 65.72 (d, C(4"')); 63.63 (t, C(5")); 63.14 (d, C(5')); 61.52 (d, C(2')); 58.92 (d, C(3)); 58.02 (d, C(1)); 56.47 (d, C(2"')); 50.66 (t, C(6"')); 31.36 (t, C(2)); 20.81, 20.70, 20.57 (3q, 6 Me). HR-MALDI-MS (*m*/*z*): 1158.2693 (35); 1144.2897 (44); 1143.2983 (46); 1142.2934 (100, $[M + Na]^+$, $C_{42}H_{50}ClN_{15}NaO_{20}^+$; calc. 1142.2943).

*6,3',2",5",3"',4"'-Hexa-*O*-acetyl-1,3,2',2"',6"'-pentadeamino-1,3,2',2"',6"'-pentaazido-4',6'-*O*-(3-hydroxybenzylidene)paromomycin* (**65**). According to **B**, reaction of **5** (500 mg, 0.5 mmol) for 3.5 h and FC (hexane/AcOEt 4:1 to 1:1) gave **65** (304 mg, 55%). White solid. *R*f (hexane/AcOEt 1:1) 0.23. IR (ATR): 3442*w* (br.), 2944*w* (br.), 2101*s*, 1742*s*, 1371*m*, 1220*s*, 1032*s*, 985*m*, 911*m*, 762*m*, 733*m*. ^{1}H-NMR (400 MHz, $CDCl_3$): 7.25–7.16 (m, 2 arom. H); 6.90–6.84 (m, 2 arom. H); 5.87 (d, *J* = 4.0, H–C(1')); 5.64 (s, Ar*CH*); 5.54 (dd, *J* = 10.0, 10.0, H–C(3')); 5.34 (d, *J* = 2.4, H–C(1")); 5.02 (dd, *J* = 2.8, 2.8, H–C(3"')); 4.94–4.88 (m, H–C(6), H–C(2''), H–C(1"')); 4.46–4.39 (m, H–C(3"), H_a–C(5'')); 4.39–4.28 (m, H–C(5'), H_a–C(6'), H–C(4'')); 4.70 (m, H–C(4''')); 4.10 (m, H–C(5"')); 3.77–3.39 (m, H–C(4'), (H_b–C(6'), H–(1), H–C(3), H–C(4), H_a–C(6''')); 3.25 (dd, J = 13.2, 4.0, H_b–C(6''')); 4.10 (dd, *J* = 12.1, 5.1, H_b–C(5'')); 3.89 (dd, *J* = 8.8, 8.8, H–C(5)); 3.31 (m, H–C(2''')); 3.15 (dd, *J* = 10.0, 4.0, H–C(2')); 2.37 (m, H_{eq}–C(2)); 1.58 (ddd, *J* = 13.2, 12.4, 12.4, H_{ax}–

C(2)) 2.162, 2.159, 2.144, 2.131, 2.110, 2.091 (6 s, 6 AcO). ^{13}C-NMR (100 MHz, CDCl3): δ 170.80, 170.23, 170.09, 169.84, 169.74, 168.56 (6 s, 6 C=O); 155.02 (s); 120.74 (2 s); 130.89, 128.04, 119.91, 117.61 (4 d); 107.28 (C(1'')); 102.93 (d, ArCH); 99.22 (C(1''')); 97.59 (C(1')); 82.33 (C(5)); 79.54 (C(4'')); 79.43 (C(4')); 76.11 (C(4)); 75.91 (C(3'')); 75.46 (C(6)); 74.85 (C(2'')); 74.72 (C(5''')); 69.00 (C(3')); 68.86, 68.84 (C(6'), C(3''')); 65.86 (C(4''')); 63.69 (C(5'')); 63.12 (C(5')); 61.54 (C(2')); 59.11 (C(3)); 58.15 (C(1)); 56.61 (C(2''')); 50.81 (C(6''')); 31.51 (C(2)); 20.99, 20.91, 20.86, 20.76, 20.64 (5q, 6 Me). HR-MALDI-MS (*m/z*): 11140.3030 (41, $[M + K]^+$, $C_{42}H_{51}KN_{15}O_{21}^+$; calc. 1140.3021), 1124.3276 (100, $[M + Na]^+$, $C_{42}H_{51}N_{15}NaO_{21}^+$; calc. 1124.3282).

*6,3',2",5",3"',4"'-Hexa-*O*-acetyl-1,3,2',2"',6"'-pentadeamino-1,3,2',2"',6"'-pentaazido-4',6'-(3-bromo-4-hydroxybenzylidene) paromomycin* (**68**). According to **B**, reaction of **60** (500 mg, 0.5 mmol) for 3.5 h and FC (hexane/AcOEt 9:1 → 5:5) gave **68** (355 mg, 60%). Light brown solid. *R*f (hexane/AcOEt 1:1) 0.27. IR (ATR): 3340*w* (br.), 2944*w*, 2932*w*, 2102*m*, 1746*m*, 1372*m*, 1224*s*, 1021*s*. ^{1}H-NMR (400 MHz, CDCl3): δ 7.54 (d, *J* = 2.0, 1 arom. H); 7.27 (dd, *J* = 8.4, 2.0, 1 arom. H); 7.12 (dd, *J* = 8.4, 1 arom. H); 5.83 (d, *J* = 4.0, H–C(1')); 5.53 (dd, *J* = 10.0, 10.0, H–C(3')); 5.39 (s, Ar*CH*); 5.33 (d, *J* = 2.0; H–C(1'')); 5.02 (dd, *J* = 2.8, 2.8, H–C(3''')); 4.92–4.87 (d, *J* = 2.0, H–C(1''')); 4.92–4.87 (m, H–C(2''), H–C(6)); 4.69 (dd, *J* = 2.8, 2.=, H–C(4''')); 4.44–4.39 (m, H–C(3''), H_a–C(5')); 4.30–4.08 (m, H–C(5'), H_a–C(6'), H–C(4''), H_b–C(5''), H–C(5''')); 3.88 (dd, *J* = 8.6, 8.6, H–C(5)); 3.72–3.54 (m, H–C(4'), H_b–C(6'), H–C(4), H_a–C(6''')); 3.51–3.37 (m, H–C(1), H–C(3)); 3.31 (m, H–C2''')); 3.2.4 (dd, *J* = 13.2, 4.0, H_b–C(6''')); 3.08 (dd, *J* = 10.0, 4.0, H–C(2')); 2.37 (ddd, *J* = 13.2, 4.4, 4.4, H_{eq}–C(2)); 2.156, 2.155, 2.138, 2.114, 2.103, 2.084 (6 s, 6 AcO); 1.60 (ddd, *J* = 13.2, 12.8, 12.8, H_{ax}–C(2)). ^{13}C-NMR (100 MHz, $CDCl_3$): δ 170.87, 170.24, 170.14, 169.87, 169.77, 168.61 (6s, 6 C=O), 153.12, 134.24 (2 s), 130.38, 127.41 (2 d), 115.89 (d), 111.19 (*s*), 109.97 (d, Ar*C*H); 107.24 (C(1'')); 100.72 (C(1')); 99.18 (C(1''')); 82.23 (C(5)); 79.35 (C(4'')); 79.01 (C(4')); 76.79 (C(4)); 75.80 (C(3'')); 74.85 (C(2'')); 75.47 (C(6)); 73.68 (C(5''')); 69.11 (C(3')); 68.83 (C(3''')); 68.74 (C(6')); 65.85 (C(4''')); 63.71 (C(5'')); 63.27 (C(5')); 61.68 (C(2')); 59.05 (C(3)); 58.14 (C(1)); 56.59 (C(2''')); 50.77 (C(6''')); 31.45 (C(2)); 20.98, 20.93, 20.88, 20.84, 20.75, 20.64 (6q, 6 Me). HR-MALDI-MS (*m/z*): 1220.2105 (44, $[M + K]^+$, $C_{42}H_{50}{}^{81}BrKN_{15}O_{21}^+$; calc. 1220.2126), 1218.2135 (33, $[M + K]^+$, $C_{42}H_{50}{}^{79}BrKN_{15}O_{21}^+$; calc. 1218.2126), 1204.2371 (100, [*M* +

Na]$^+$, $C_{42}H_{50}{}^{81}BrN_{15}NaO_{21}{}^+$; calc. 1204.2387), 1202.2381 (88, [*M* + Na]$^+$, $C_{42}H_{50}{}^{79}BrN_{15}NaO_{21}{}^+$; calc. 1202.2387).

*6,3',2'',5'',3''',4'''-Hexa-*O*-acetyl-1,3,2',2''',6'''-pentadeamino-1,3,2',2''',6'''-pentaazido-4',6'-*O*-(3,4,5-trimethoxybenzylidene)paromomycin* (**69**). According to **B**, reaction of 61 mg of **60** for 21 h and FC gave 50 mg of **69** (70%). White solid. R_f (hexane/AcOEt 1:1) 0.34. M.p. 95 (softening)–110°. $[\alpha]_D^{25}$ = +92.5 (*c* = 0.23, $CHCl_3$). IR (ATR): 2941*w*, 2876*w*, 2839*w*, 2101*s*, 1743*s*, 1594*w*, 1508*w*, 1462*w*, 1421*w*, 1371*m*, 1330*w*, 1219*s*, 1156*w*, 1125*m*, 1096*m*, 1030*s*. ^{1}H-NMR (300 MHz, $CDCl_3$): δ 6.68 (s, 2 arom. H); 5.84 (d, *J* = 4.0, H–C(1')); 5.56 (t, *J* = 10.3, H–C(3')); 5.43 (s, ArCH); 5.34 (d, *J* = 2.5, H–C(1'')); 5.02 (t, *J* = 2.8, H–C(3''')); 4.93 (t, *J* = 10.0, H–C(6)); 4.91–4.88 (m, H–C(2'')); 4.88 (d, *J* = 1.9, H–C(1''')); 4.70 (t, *J* = 1.9, H–C(4''')); 4.45–4.39 (m, H_a–C(5''), H–C(3'')); 4.32–4.19 (m, H–C(5'), H–C(4''), H_a–C(6'), H_b–C(5'')); 4.13–4.09 (m, H–C(5''')); 3.90 (t, *J* = 9.0, H–C(5)); 3.88–3.85 (m, H_b–C(6')); 3.85 (2s, 2 MeO); 3.82 (s, MeO); 3.74–3.39 (m, H–C(4), H–C(4'), H_a–C(6'''), H–C(3), H–C(1)); 3.31 (t, *J* = 1.9, H–C(2''')); 3.24 (dd, *J* = 13.1, 4.1 , H_b–C(6''')); 3.08 (dd, *J* = 10.6, 3.7, H–C(2')); 2.39 (dt, *J* = 13.4, 4.4, H_{eq}–C(2)); 2.169, 2.165, 2.15, 2.12, 2.11, 2.10 (6s, 6 AcO); 1.60 (q, *J* = 12.8, H_{ax}–C(2)). ^{13}C-NMR (75 MHz, $CDCl_3$): δ 170.72, 170.12, 169.94, 169.75, 169.65, 168.49 (6s, 6 C=O); 153.13 (2s); 138.55 (s), 132.46 (s); 107.14 (d, C(1'')); 103.37 (2d); 101.48 (d, ArCH); 99.08 (d, C(1''')); 97.64 (d, C(1')); 82.22 (d, C(5)); 79.24 (d, C(4'')); 78.94 (d, C(4')); 77.24 (d, C(4)); 75.77 (d, C(3'')); 75.35 (d, C(6)); 74.74 (d, C(2'')); 73.60 (d, C(5''')); 69.01 (d, C(3')); 68.70 (d, C(3'''), t, C(6')); 65.72 (d, C(4''')); 63.60 (t, C(5'')); 63.24 (d, C(5')); 61.46 (d, C(2')); 60.78 (q, MeO); 59.00 (d, C(3)); 58.00 (d, C(1)); 56.46 (d, C(2''')); 56.06 (2q, 2 MeO); 50.66 (t, C(6''')); 31.43 (t, C(2)); 20.86, 20.78, 20.66, 20.55 (q, 6 Me). HR-MALDI-MS (*m*/*z*): 1215.3367 (31); 1214.3355 (57, [*M* + K]$^+$, $C_{45}H_{57}KN_{15}O_{23}{}^+$; calc. 1214.3389); 1199.3654 (56); 1198.3634 (100, [*M* + Na]$^+$, $C_{45}H_{57}N_{15}NaO_{23}{}^+$; calc. 1198.3649). Anal. calc. for $C_{45}H_{57}N_{15}O_{23}$ (1176.03): C 45.96, H 4.89, N 17.87; found: C 45.87, H 5.03, N 17.83.

*6,3',2'',5'',3''',4'''-Hexa-*O*-acetyl-1,3,2',2''',6'''-pentadeamino-1,3,2',2''',6'''-pentaazido-4',6'-*O*-furfurylideneparomomycin* (**70**). According to **A**, reaction of 146 mg of **60** for 40 min and FC gave 91 mg of **70** (58%). Pale orange solid. R_f (hexane/AcOEt 1:1) 0.60. M.p. 98 (softening)–105°.

$[\alpha]_D^{25}$ = +117.2 (*c* = 0.11, $CHCl_3$). IR (ATR): 2945*w*, 2872*w*, 2102*s*, 1744*s*, 1431*w*, 1370*m*, 1332*w*, 1221*s*, 1174*w*, 1125*w*, 1093*m*, 1032*s*, 994*m*, 972*m*, 925*m*. ^{1}H-NMR (300 MHz, $CDCl_3$): δ 7.39 (br. s, 1 arom. H); 6.46–6.43 (m, 1 arom. H); 6.38–6.35 (m, 1 arom. H); 5.81 (d, *J* = 3.9, H–C(1')); 5.55 (s, ArCH); 5.54 (t, *J* = 10.0, H–C(3')); 5.34 (d, *J* = 1.7, H–C(1")); 5.02 (t, *J* = 2.5, H–C(3"')); 4.93 (t, *J* = 9.6, H–C(6)); 4.93–4.86 (m, H–C(2"), H–C(1"')); 4.70 (br. s, H–C(4"')); 4.46–4.37 (m, H_a–C(5"), H–C(3")); 4.34–4.22 (m, H–C(5'), H–C(4"), H_a–C(6'), H_b–C(5")); 4.14–4.08 (m, H–C(5"')); 3.90 (t, *J* = 8.8, H–C(5)); 3.73–3.38 (m, H_b–C(6'), H–C(4), H–C(4'), H_a–C(6"'), H–C(3), H–C(1)); 3.31 (br. s, H–C(2"')); 3.24 (dd, *J* = 13.2, 4.1 , H_b–C(6"')); 3.06 (dd, *J* = 10.4, 3.8, H–C(2')); 2.39 (dt, *J* = 13.5, 4.1, H_{eq}–C(2)); 2.167, 2.155, 2.13, 2.11, 2.09 (5s, 6 AcO); 1.61 (q, *J* = 12.9, H_{ax}–C(2)). ^{13}C-NMR (75 MHz, $CDCl_3$): δ 170.71, 170.14, 169.97, 169.78, 169.67, 168.52 (6s, 6 C=O); 149.55 (s); 142. 67 (d); 110.28 (d); 108.14 (d); 107.19 (d, C(1")); 99.08 (d, C(1"')); 97.69 (d, C(1')); 96.27 (d, ArCH); 82.18 (d, C(5)); 79.22 (d, C(4")); 78.99 (d, C(4')); 75.76 (d, C(3")); 75.35 (d, C(6)); 74.72 (d, C(2")); 73.61 (d, C(5"')); 68.87 (d, C(3')); 68.73 (d, C(3"')); 68.61 (t, C(6')); 65.73 (d, C(4"')); 63.61 (t, C(5")); 63.06 (d, C(5')); 61.57 (d, C(2')); 58.83 (d, C(3)); 58.02 (d, C(1)); 56.46 (d, C(2"')); 50.66 (t, C(6"')); 31.27 (t, C(2)); 20.86, 20.79, 20.68, 20.56 (6q, 6 Me); d of C(4) hidden by solvent peaks. HR-MALDI-MS (*m*/*z*): 1115.2929 (40); 1114.2916 (75, $[M + K]^+$, $C_{40}H_{49}KN_{15}O_{21}^+$; calc. 1114.2865); 1099.3154 (50); 1098.3129 (100, $[M + Na]^+$, $C_{40}H_{49}N_{15}NaO_{21}^+$; calc. 1098.3125). Anal. calc. for $C_{40}H_{49}N_{15}O_{21}$ (1075.91): C 44.65, H 4.59, N 19.53; found: C 44.74, H 4.59, N 19.05.

6,3',2",5",3"',4"'-Hexa-O-*acetyl-1,3,2',2"',6"'-pentadeamino-1,3,2',2"',6"'-pentaazido-4',6'*-O-*[(1-naphthyl)methylidene]paromomycin* (**73**). According to **A**, reaction of 195 mg of **60** for 20 min and FC gave 157 mg of **73** (71%). White solid. R_f (hexane/AcOEt 1:1) 0.55. M.p. 77 (softening)–115°. $[\alpha]_D^{25}$ = 0.21, CHCl3). IR (ATR): 2941*w*, 2876*w*, 2100*s*, 1742*s*, 1510*w*, 1431*w*, 1370*m*, 1215*s*, 1172*w*, 1123*w*, 1104*m*, 1029*s*, 988*m*. ^{1}H-NMR (300 MHz, $CDCl_3$): δ 8.15–8.12 (m, 1 arom H); 7.86–7.84 (m, 2 arom. H); 7.74–7.71 (m, 1 arom. H); 7.53–7.43 (m, 3 arom. H); 6.04 (s, ArCH); 5.90 (d, *J* = 3.9, H–C(1')); 5.62 (t, *J* = 9.9, H–C(3')); 5.35 (d, *J* = 1.9, H–C(1")); 5.03 (br. s, H–C(3"')); 4.98–4.86 (m, H–C(6), H–C(2"), H–C(1"')); 4.71 (br. s, H–C(4"')); 4.50–4.28 (m, H_a–C(5"), H–C(3"), H–C(5'), H–C(4"), H_a–C(6')); 4.24 (dd, *J* = 12.1, 5.2, H_b–C(5")); 4.16–4.08 (m, H–C(5"')); 3.95–3.37 (m, H–

C(5), H_b–C(6'), H–C(4), H–C(4'), H_a–C(6'''), H–C(3), H–C(1)); 3.33 (br. s, H–C(2''')); 3.25 (dd, *J* = 13.2, 4.1 , H_b–C(6''')); 3.13 (dd, *J* = 10.2, 3.6, H–C(2')); 2.40 (dt, *J* = 13.2, 4.4, H_{eq}–C(2)); 2.17, 2.16. 2.08 (3s, 6 AcO; 1.61 (q, *J* = 12.4, H_{ax}–C(2)). ^{13}C-NMR (75 MHz, $CDCl_3$): δ 170.75, 170.15, 169.96, 169.78, 169.67, 168.52 (6s, 6 C=O); 133.78; 132.05, 130.45; 129.85; 128.58; 126.18; 125.67; 125.03; 124.62; 124.10; 107.16 (d, C(1'')); 101.08 (d, ArCH); 99.14 (d, C(1''')); 97.67 (d, C(1')); 82.27 (d, C(5)); 79.45 (d, C(4'')); 79.31 (d, C(4')); 77.23 (d, C(4)); 75.84 (d, C(3'')); 75.39 (d, C(6)); 74.80 (d, C(2'')); 73.60 (d, C(5''')); 68.99, 68.74 (d, C(3'), d, C(3'''), t, C(6')); 65.74 (d, C(4''')); 63.64 (t, C(5'')); 63.34 (d, C(5')); 61.56 (d, C(2')); 59.08 (d, C(3)); 58.05 (d, C(1)); 56.49 (d, C(2''')); 50.68 (t, C(6''')); 31.46 (t, C(2)); 20.90, 20.82, 20.69, 20.57 (4q, 6 Me). HR-MALDI-MS (*m*/*z*): 1175.3224 (46); 1174.3203 (82, $[M + K]^+$, $C_{46}H_{53}KN_{15}O_{20}^+$; calc. 1174.3228); 1159.3523 (56); 1158.3489 (100, $[M + Na]^+$, $C_{46}H_{53}N_{15}NaO_{20}^+$; calc. 1158.3489). Anal. calc. for $C_{46}H_{53}N_{15}O_{20}$.0.2 AcOEt (1153.62): C 48.72, H 4.77, N 18.21; found: C 48.76, H 4.89, N 17.77.

*6,3',2'',5'',3''',4'''-Hexa-*O*-acetyl-1,3,2',2''',6'''-pentadeamino-1,3,2',2''',6'''-pentaazido-4',6'-*O*-(2-phenylethylidene)paromomycin* (**75**). According to **A**, reaction of 116 mg of **60** for 2 h and FC gave 97 mg of **75** (76%). White solid. R_f (hexane/AcOEt 1:1) 0.46. M.p. 90 (softening)–96°. $[\alpha]_D^{25}$ = +90.0 (*c* = 0.105, $CHCl_3$). IR (ATR): 2941*w*, 2876*w*, 2101*s*, 1743*s*, 1496*w*, 1455*w*, 1433*w*, 1371*m*, 1336*w*, 1219*s* , 1170*w*, 1125*m*, 1029*s*, 891*w*. ^{1}H-NMR (300 MHz, $CDCl_3$ δ 7.26–7.20 (m, 5 arom. H); 5.79 (d, *J* = 4.1, H–C(1')); 5.49 (t, *J* = 10.0, H–C(3')); 5.33 (d, *J* = 2.2, H–C(1'')); 5.02 (t, *J* = 2.5, H–C(3''')); 4.92 (t, *J* = 9.3, H–C(6)); 4.90–4.86 (m, H–C(2'')); 4.87 (d, *J* = 1.6, H–C(1''')); 4.69 (br. s, H–C(4''')); 4.63 (t, *J* = 5.0, $PhCH_2CH$); 4.44–4-.36 (m, 2 H, H_a–C(5''), H–C(3'')); 4.29 (td, *J* = 5.0, 1.9, H–C(4'')); 4.15–4.06 (m, H_b–C(5''), H–C(5'), H_a–C(6'), H–C(5''')); 3.88 (t, *J* = 8.8, H–C(5)); 3.69–3.55 (m, H–C(4), Hb–C(6')); 3.53–3.38 (m, H–C(4'), H_a–C(6'''), H–C(3), H–C(1)); 3.32–3.28 (m, H–C(2''')); 3.24 (dd, *J* = 13.1, 4.0, H_b–C(6''')); 3.05 (dd, *J* = 10.6, 3.7, H–C(2')); 3.00–2.85 (m, $PhCH_2$); 2.39 (dt, *J* = 13.4, 4.4, H_{eq}–C(2)); 2.17, 2.15, 2.11, 2.09, 2.07, 2.06 (6s, 6 AcO); 1.60 (q, *J* = 12.8, H_{ax}–C(2)). ^{13}C-NMR (75 MHz, $CDCl_3$): δ 170.68, 170.16, 169.97, 169.79, 169.68, 168.52 (6s, 6 C=O); 136.14 (s); 129.85 (2d); 128.14 (2d); 126.57 (d); 107.10 (d, C(1'')); 102.77 (d, $PhCH_2CH$); 99.11 (d, C(1''')); 97.50 (d, C(1')); 82.15 (d, C(5)); 79.28 (d, C(4'')); 78.71 (d, C(4')); 77.24 (d, C(4)); 75.82 (d, C(3'')); 75.36 (d, C(6)); 74.73 (d, C(2'')); 73.59 (d, C(5''')); 69.01 (d, C(3')); 68.75 (d, C(3''')); 68.22,

(t, C(6')); 65.73 (d, C(4''')); 63.59 (t, C(5'')); 63.28 (d, C(5')); 61.38 (d, C(2')); 58.95 (d, C(3)); 58.06 (d, C(1)); 56.49 (d, C(2''')); 50.66 (t, C(6''')); 40.73 (t, $PhCH_2$); 31.36 (t, C(2)); 20.90, 20.78, 20.70, 20.58 (4q, 6 Me). HR-MALDI-MS (*m/z*): 1138.3234 (39, $[M + K]^+$, $C_{43}H_{53}KN_{15}O_{20}^+$; calc. 1138.3228); 1123.3511 (53); 1122.3475 (100, $[M + Na]^+$, $C_{43}H_{53}N_{15}NaO_{20}^+$; calc. 1122.3489).

6,3',2'',5'',3''',4'''-Hexa-O-*acetyl-1,3,2',2''',6'''-pentadeamino-1,3,2',2''',6'''-pentaazido-4',6'*-O-*(3-phenylpropylidene)paromomycin* (**76**). According to **A**, reaction of 177 mg of **60** for 30 min and FC gave 189 mg of **76** (96%). White solid. R_f (hexane/AcOEt 1:1) 0.50. M.p. 66 (softening)–86°. $[\alpha]_D^{25}$ = +108.7 (*c* = 0.24, $CHCl_3$). IR (ATR): 2949*w*, 2876*w*, 2100*s*, 1741*s*, 1494*w*, 1451*w*, 1429*w*, 1370*m*, 1217*s*, 1170*w*, 1120*m*, 1028*s*. ^{1}H-NMR (300 MHz, $CDCl_3$): δ 7.32–7.14 (m, 5 arom. H); 5.81 (d, *J* = 4.1, H–C(1')); 5.48 (t, *J* = 10.4, H–C(3')); 5.33 (d, *J* = 2.5, H–C(1'')); 5.02 (t, *J* = 2.8, H–C(3''')); 4.92 (t, *J* = 10.2, H–C(6)); 4.90–4.83 (m, H–C(2'')); 4.87 (d, *J* = 1.9, H–C(1''')); 4.70–4.69 (br. s, H–C(4''')); 4.47–4.36 (m, $Ph(CH_2)_2CH$, H_a–C(5''), H–C(3'')); 4.30 (td, *J* = 4.7, 1.9, H–C(4'')); 4.20 (dd, *J* = 12.1, 5.2, H_b–C(5'')); 4.15–4.06 (m, H–C(5'), H_a–C(6'), H–C(5''')); 3.88 (t, *J* = 8.8, H–C(4)); 3.65 (t, *J* = 9.6, H–C(5)); 3.56 (dd, *J* = 13.2, 8.2, H_b–C(6')); 3.52–3.28 (m, H–C(4'), H_a–C(6'''), H–C(3), H–C(1), H–C(2''')); 3.24 (dd, *J* = 12.9, 4.1, H_b–C(6''')); 3.02 (dd, *J* = 10.4, 4.1, H–C(2')); 2.69 (t, *J* = 7.4, $PhCH_2$); 2.38 (dt, *J* = 13.2, 4.7, H_{eq}–C(2)); 2.17, 2.16, 2.15, 2.11, 2.07 (5s, 6 AcO); 2.06–1.92 (m, $PhCH_2CH_2CH$); 1.60 (q, *J* = 12.6, H_{ax}–C(2)). ^{13}C-NMR (75 MHz, $CDCl_3$): δ 170.70, 170.13, 170.01, 169.77, 169.66, 168.51 (6s, 6 C=O); 141.35 (s); 128.47 (d, 2C); 128.40 (d, 2 C); 125.93 (d); 107.10 (d, C(1'')); 101.55 (d, $Ph(CH_2)_2CH$); 99.11 (d, C(1''')); 97.56 (d, C(1')); 82.20 (d, C(5)); 79.27 (d, C(4'')); 78.59 (d, C(4')); 76.47 (d, C(4)); 75.81 (d, C(3'')); 75.36 (d, C(6)); 74.76 (d, C(2'')); 73.57 (d, C(5''')); 69.08 (d, C(3')); 68.72, 68.22 (d, C(3'''), t, C(6')); 65.72 (d, C(4''')); 63.57 (t, C(5'')); 63.40 (d, C(5')); 61.50 (d, C(2')); 58.99 (d, C(3)); 58.04 (d, C(1)); 56.48 (d, C(2''')); 50.65 (t, C(6''')); 35.32 (t, $PhCH_2$); 31.39 (t, C(2)); 30.13 (t, $PhCH_2CH_2$); 20.88, 20.84, 20.78, 20.76, 20.67, 20.56 (6q, 6 Me). HR-MALDI-MS (*m/z*): 1153.3457 (27); 1152.3403 (49, $[M + K]^+$, $C_{44}H_{55}KN_{15}O_{20}^+$; calc. 1152.3385); 1137.3650 (54); 1136.3619 (100, $[M + Na]^+$, $C_{44}H_{55}N_{15}NaO_{20}^+$; calc. 1136.3640).

6,3',2'',5'',3''',4'''-Hexa-O-*acetyl-1,3,2',2''',6'''-pentadeamino-1,3,2',2''',6'''-pentaazido-4',6'*-O-*(4-phenylbutylidene)paromomycin* (**79**). According to **B**, reaction of 108 mg of **60** for 26 h

and FC gave 111 mg of **79** (91%). White solid. R_f (Hexane/AcOEt 1:1) 0.58. M.p. 76 (softening)–81°. $[\alpha]_D^{25}$ = +105.4 (c = 0.19, $CHCl_3$). IR (ATR): 2937*w*, 2864*w*, 2100*s*, 1742*s*, 1496*w*, 1451*w*, 1433*w*, 1370*m*, 1336*w*, 1217*s*, 1168*w*, 1122*m*, 1029*s*, 980*m*, 952*m*. ^{1}H-NMR (300 MHz, $CDCl_3$): δ 7.29–7.14 (m, 5 arom. H); 5.80 (d, J = 3.9, H–C(1')); 5.46 (t, J = 10.2, H–C(3')); 5.33 (d, J = 2.2, H–C(1")); 5.02 (t, J = 2.8, H–C(3"')); 4.92 (t, J = 9.9, H–C(6)); 4.90–4.84 (m, H–C(2"), H–C(1"')); 4.69 (br. s, H–C(4"')); 4.52–4.46 (m, Ph(CH_2)$_3$CH); 4.44–4.34 (m, H_a–C(5"), H–C(3")); 4.29 (td, J = 4.9, 1.0, H–C(4")); 4.20 (dd, J = 11.8, 5.2, H_b–C(5")); 4.16–4.06 (m, 3 H, H–C(5'), H_a–C(6'), H–C(5"')); 3.87 (t, J = 8.8, H–C(5)); 3.67–3.30 (m, H_b–C(6'), H–C(4), H–C(4'), H_a–C(6"'), H–C(3), H–C(1), H–C(2"')); 3.24 (dd, J = 13.2, 4.1, H_b–C(6"')); 3.01 (dd, J = 10.4, 3.9, H–C(2')); 2.63–2.58 (m, $PhCH_2$); 2.38 (dt, J = 13.2, 4.4, H_{eq}–C(2)); 2.167, 2.159, 2.151, 2.12, 2.11, 2.07 (6s, 6 AcO); 1.76–1.60 (m, $PhCH_2CH_2CH_2$); 1.58 (q, J = 12.9, H_{ax}–C(2)). ^{13}C-NMR (75 MHz, $CDCl_3$): δ 170.73, 170.14, 170.04, 169.79, 169.67, 168.53 (6s, 6 C=O); 142.14 (s); 128.43 (2d); 128.30 (2d); 125.75 (d); 107.08 (d, C(1")); 102.54 (d, Ph(CH_2)$_3$CH); 99.10 (d, C(1"')); 97.53 (d, C(1')); 82.18 (d, C(5)); 79.22 (d, C(4")); 78.59 (d, C(4')); 76.43 (d, C(4)); 75.79 (d, C(3")); 75.34 (d, C(6)); 74.74 (d, C(2")); 73.56 (d, C(5"')); 69.03 (d, C(3')); 68.68 (d, C(3"')); 68.22 (t, C(6')); 65.69 (d, C(4"')); 63.60 (t, C(5")); 63.36 (d, C(5')); 61.46 (d, C(2')); 58.94 (d, C(3)); 58.01 (d, C(1)); 56.45 (d, C(2"')); 50.62 (t, C(6"')); 35.48 (t, $PhCH_2$); 33.50 (t, $PhCH_2CH_2CH_2$); 31.36 (t, C(2)); 25.77 (t, $PhCH_2CH_2CH_2$); 20.79, 20.69, 20.56 (3q, 6 Me). HR-MALDI-MS (m/z): 1167.3547 (38); 1166.3522 (69, $[M + K]^+$, $C_{45}H_{57}KN_{15}O_{20}^+$; calc. 1166.3541); 1151.3795 (56); 1150.3778 (100, $[M + Na]^+$, $C_{45}H_{57}N_{15}NaO_{20}^+$; calc. 1150.3797). Anal. calc. for $C_{45}H_{57}N_{15}O_{20}$ (1128.02): C 47.91, H 5.09, N 18.63; found: C 47.99, H 5.13, N 18.20.

*6,3',2",5",3"',4"'-Hexa-*O*-acetyl-1,3,2',2"',6"'-pentadeamino-1,3,2',2"',6"'-pentaazido-4',6'-*O*-(5-phenylpentylidene)paromomycin* (**80**). According to **B**, reaction of 99 mg of **60** for 2 h 15 and FC gave 107 mg of **80** (94%). White solid. R_f (hexane/AcOEt 1:1) 0.66. ^{1}H-NMR (300 MHz, $CDCl_3$): δ 7.29–7.14 (m, 5 arom. H); 5.80 (d, J = 3.7, H–C(1')); 5.46 (t, J = 9.8, H–C(3')); 5.34 (br. s, H–C(1")); 5.02 (t, J = 2.7, H–C(3"')); 4.92 (t, J = 9.4, H–C(6)); 4.92–4.87 (m, H–C(2"), H–C(1"')); 4.70 (br. s, H–C(4"')); 4.48 (t, J = 5.1, Ph(CH_2)$_4$CH); 4.44–4.37 (m, H_a–C(5"), H–C(3")); 4.31–4.27 (m, H–C(4")); 4.20 (dd, J = 12.0, 4.8, H_b–C(5")); 4.14–4.08 (m, H–C(5'), H_a–C(6'), H–C(5"')); 3.88 (t, J = 8.5, H–C(5)); 3.67–3.30 (m, H_b–C(6'), H–C(4), H–C(4'), H_a–C(6"'), H–C(3), H–C(1), H–

C(2''')); 3.25 (dd, *J* = 13.0, 4.2, H_b–C(6''')); 3.02 (dd, *J* = 10.5, 3.8, H–C(2')); 2.61–2.56 (m, 2 H, $PhCH_2$); 2.40–2.34 (m, H_{eq}–C(2)); 2.17, 2.16, 2.15, 2.11, 2.09, 2.08 (6s, 6 AcO); 1.69–1.53 (m, H_{ax}–C(2), 2 CH_2); 1.45–1.40 (m, CH_2). ^{13}C-NMR (75 MHz, $CDCl_3$): δ 170.72, 170.15, 170.03, 169.79, 169.68, 168.53 (6s, 6 C=O); 142.41 (s); 128.41 (d, 2 C); 128.29 (d, 2 C); 125.68 (d); 107.11 (d, C(1'')); 102.67 (d, $Ph(CH_2)_4CH$); 99.13 (d, C(1''')); 97.55 (d, C(1')); 82.20 (d, C(5)); 79.27 (d, C(4'')); 78.62 (d, C(4')); 75.82 (d, C(3'')); 75.357 (d, C(6)); 74.77 (d, C(2'')); 73.59 (d, C(5''')); 69.08 (d, C(3')); 68.74, 68.26 (d, C(3'''), t, C(6')); 65.73 (d, C(4''')); 63.62 (t, C(5'')); 63.41 (d, C(5')); 61.50 (d, C(2')); 59.00 (d, C(3)); 58.06 (d, C(1)); 56.49 (d, C(2''')); 50.69 (t, C(6''')); 35.87 (t, $PhCH_2$); 33.89 (t, $Ph(CH_2)_3CH_2$); 31.39 (t, C(2)); 31.19 (t, $PhCH_2CH_2$); 23.84 (t, $PhCH_2CH_2CH_2$); 21.90, 20.79, 20.69, 20.57 (4q, 6 Me).A (d, C(4)) hidden by solvent peaks. HR-MALDI-MS (*m*/*z*): 1180.3698 (42, $[M + K]^+$, $C_{46}H_{59}KN_{15}O_{20}^+$; 1180.3698); 1165.4008 (60); 1164.3974 (100, $[M + Na]^+$, $C_{46}H_{59}N_{15}NaO_{20}^+$; calc. 1164.3958). Anal. calc. for $C_{46}H_{59}N_{15}O_{20}$.0.5 AcOEt (1186.10): C 48.70, H 5.29, N 17.57; found: C 48.61, H 5.35, N 17.71.

6,3',2'',5'',3''',4'''-Hexa-O-*acetyl-1,3,2',2''',6'''-pentadeamino-1,3,2',2''',6'''-pentaazido-4',6'*-O-*(3-formyl-4-hydroxybenzylidene)paromomycin* (**101**) *and 6,3',2'',5'',3''',4'''-Hexa*-O-*acetyl-1,3,2',2''',6'''-pentadeamino-1,3,2',2''',6'''-pentaazido-4',6'*-O-*(3-formyl-2-hydroxybenzylidene)paromomycin* (**102**). According to **B**, reaction of **60** (500 mg, 0.5 mmol) for 3.5 h gave crude **101**/**102** ≥ 10:1. FC (hexane/AcOEt 7:3 gave **102** (31 mg, 5%) and FC (hexane/AcOEt 1:1) gave **101** (310 mg, 55%). *Data of* **101**: Light brown solid. *R*f (hexane/AcOEt 5:5) 0.24. IR (ATR): 2981*s*, 2973*s*, 2939*s*, 2866*m*, 2844*m*, 2101*s*, 1742*s*, 1659*m*, 1371*m*, 1213*s*, 1054*s*, 1016*s*, 907*m*, 727*s*. ^{1}H-NMR (400 MHz, CDCl3): δ 9.87 (s, Ar*CHO)* 7.65 (d, *J* = 2.4, 1 arom. H); 7.55 (dd, *J* = 8.8, 2.4, 1 arom. H); 6.95 (d, *J* = 8.8, 1 arom. H); 5.85 (d, *J* = 4.0, H–C(1')); 5.54 (dd, *J* = 9.8, 9.8, H–C(3')); 5.46 (s, Ar*CH*); 5.34 (d, *J* = 2.4; H–C(1'')); 5.01 (dd, *J* = 2.8, 2.8, H–C(3''')); 4.91–4.87 (d, *J* = 2.0, H–C(1''')); 4.91–4.87 (m, H–C(2''), H–C(6)); 4.69 (dd, *J* = 2.8, 2.0, H–C(4''')); 4.44–4.38 (m, H–C(3''), H_a–C(5'')); 4.31–4.23 (m, H–C(5'), H_a–C(6'), H–C(4'')); 4.21 (dd J = 12.0, 4.8, H_b–C(5''), H–C(5''')); 4.06–3.97 (m, H–C(5''')); 3.88 (dd, *J* = 8.8, 8.8, H–C(5)); 3.73–3.39 (m, H–C(4'), H_b–C(6'), H–C(1), H–C(3), H–C(4), H_a–C(6''')); 3.31 (m, H–C(2''')); 3.24 (dd, *J* = 13.2, 4.4, H_b–C(6''')); 3.02 (dd, *J* = 10.0, 4.0, H–C(2')); 2.37 (ddd, *J* = 13.2, 4.4, 4.4, H_{eq}–C(2)); 2.152, 2.149, 2.134, 2.110, 2.099,

2.081 (6 s, 6 AcO); 1.59 (ddd, *J* = 13.2, 13.2, 13.2, H_{ax}–C(2)). ^{13}C-NMR (100 MHz, CDCl3): δ 196.288 (s, CHO), 170.77, 170.19, 170.04, 169.82, 168.72 (5s, 6 C=O); 162.18 (s); 135.13, 131.79 (2 d); 129.17, 120.13 (2 s); 117.65, 107.24 (2 d); 100.63 *(d,* Ar*C*H); 107.24 (C(1'')); 99.18 (C(1''')); 97.67 (C(1')); 82.30 (C(5)); 79.36 (C(4'')); 79.08 (C(4')); 76.75 (C(4)); 75.88 (C(3'')); 74.83 (C(2'')); 75.44 (C(6)); 73.67 (C(5''')); 69.08 (C(3')); 68.81 (C(3''')); 68.74 (C(6')); 65.82 (C(4''')); 63.68 (C(5'')); 63.23 (C(5')); 61.62 (C(2')); 59.00 (C(3)); 58.12 (C(1)); 56.58 (C(2''')); 50.77 (C(6''')); 31.47 (C(2)); 20.95, 20.91, 20.86, 20.82, 20.72, 20.61 (6 q, 6 Me). HR-MALDI-MS (*m*/*z*): 1152.3225 (100, $[M + Na]^+$, $C_{43}H_{51}N_{15}NaO_{22}^+$; calc. 1152.3231).

Data of **102**: Light brown solid. *R*f (hexane/AcOEt 1:1) 0.38. IR (ATR): 3333*w*, 2941*w*, 2857*w*, 2102*s*, 1744*s*, 1659*s*, 1371*m*, 1221*s*, 1091*m*, 1030*s*, 768*w*. ^{1}H-NMR (400 MHz, $CDCl_3$): δ 9.88 (s, ArCHO); 7.66 (d, *J* = 2.0, 1 arom. H); 7.57 (dd, *J* = 8.8, 2.4, 1 arom. H); 6.97 (*d*, *J* = 8.8, 1 arom. H); 5.85 (d, *J* = 3.6, H–C(1')); 5.56 (dd, *J* = 10.0, 10.0, H–C(3')); 5.48 (s, Ar*CH*); 5.42–5.35 (m, H–C(1'')); 5.42–5.35 (m, H–C(3''')); 5.03–4.88 (m, H–C(6), H–C(2''), H–C(1'''), H–C(4''')); 4.44–4.23 (H–C(5'), H_a–C(6'), H–C(3''), H–C(4'')); H_a–C(5''), H_b–C(5'')); 3.88 (dd, *J* = 9.0, 9.0, H–C(5)); 3.70–3.59 (m, H–C(4'), H_b–C(6'), H–C(4)); 3.53–3.38 (m, H–C(1), H–C(3), H–C(2'''), H_a–C(6''')); 3.22 (dd, *J* = 13.2, 5.6, H_b–C(6''')); 3.16 (dd, *J* = 10.0, 3.6, H–C(2')); 2.37 (ddd, *J* = 13.2, 4.4, 4.4, H_{eq}–C(2)); 2.223, 2.171, 2.131, 2.123, 2.081, 2.044 (6 s, 6 AcO), 1.59 (ddd, *J* = 13.2, 13.2, 13.2, H_{ax}–C(2)). ^{13}C-NMR (100 MHz, CDCl3):δ 196.29 (s, CHO), 170.77, 170.19, 170.04, 169.82, 168.72 (5s, 6 C=O); 162.26 (s); 135.17 (d, 1 C); 131.83 (d); 129.1, 120.19 (2 s); 117.58 (d); 100.70 (d, ArCH); 106.74 (C(1'')); 97.72 (C(1''')); 97.51 (C(1')); 81.38 (C(5)); 80.06 (C(4'')); 79.14 (C(4')); 76.94 (C(4)); 75.14 (C(3'')); 74.10 (C(2'')); 74.95 (C(6)); 70.10 (C(5''')); 69.85 (C(3')); 69.46 (C(3''')); 68.79 (C(6')); 69.16 (C(4''')); 63.38 (C(5'')); 62.29 (C(5')); 61.87 (C(2')); 61.10 (C(3)); 59.16 (C(1)); 58.25 (C(2''')); 51.03 (C(6''')); 31.70 (t, C(2)); 20.97, 20.89, 20.75, 20.69, 20.61 (5q, 6 Me). HR-MALDI-MS (*m*/*z*) (negative mode): 1128.3260 (100, $[M - H]^-$, $C_{43}H_{50}N_{15}O_{22}^-$; calc. 1128.3255).

6,3',2'',5'',3''',4'''-Hexa-O-*acetyl-1,3,2',2''',6'''-pentadeamino-1,3,2',2''',6'''-pentaazido-4',6'*-O-*(3-carboxy-4-hydroxybenzylidene)paromomycin* (**103**). According to **B**, reaction of **60** (500

mg, 0.5 mmol) for 3.5 h and FC ($CHCl_3$/AcOEt/MeOH 4:4:0.2 → 4:4:1) gave **103** (345 mg, 60%). Light brown solid. *R*f ($CHCl_3$/AcOEt/MeOH 4:4:1) 0.14. IR (ATR): 3345*w*, 2942*w*, 2101*s*, 1742*s*, 1674*w* (sh.), 1371*m*, 1221*s*, 1094*m*, 1039*s*. $[\alpha]_D^{25}$ = +106.6 (*c* = 0.5, MeOH/35 µl DMSO): ^{1}H-NMR (400 MHz, D_6-DMSO): δ 7.75 (d, *J* = 2.0, 1 arom. H); 7.23 (dd, *J* = 8.4, 2.0, 1 arom. H); 6.69 (dd, *J* = 8.4, 1 arom. H); 5.89 (d, *J* = 3.6, H–C(1')); 5.43-5.36 (m, H–C(3'), H–C(1'')); 5.55 (s, Ar*CH*); 5.07–4.90 (dd, *J* = 4.8, 4.8, H–C(3''')); 5.07–4.90 (m, H–C(6), H–C(1'''), H–C(2'')); 4.69 (m, H–C(4''')); 4.43 (dd, *J* = 4.8, 4.8, H–C(3'')); 4.31–3.95 (m, H–C(5'), H_a–C(6'), H–C(4), H–C(5), H–C(4''), H_a–C(5''), H_a–C(5''), H–C(5'''), 3.82–3.66 (m, H–C(3)); 3.54–3.38 (m, H_a–C(6''')); 3.34 (dd, *J* = 12.8, 3.6, H_b–C(6''')); 2.55–2.53 (m, H_{eq}–C(2)); 2.13, 2.10, 2.09, 2.07, 2.04, 1.99 (6 s, 6 AcO); 1.76 (ddd, *J* = 12.4, 12.4, 12.4, H_{ax}–C(2)). ^{13}C-NMR (100 MHz, D_6-DMSO): δ 171.56, (s, CO_2H); 170.25, 169.64, 169.51, 169.38, 169.28 (5s, 6 C=O); 162.93 (s); 130.27, 128.33 (2 d); 125.84 (s); 118.18 (s); 115.78 (d); 101.02 (d, ArCH); 105.40 (C(1'')); 98.67 (C(1''')); 96.64 (C(1')); 80.75 (C(5)); 79.54 (C(4'')); 77.63 (C(4')); 75.95 (C(3'')); 74.81 (C(4)); 74.18 (C(2'')); 74.63 (C(6)); 72.63 (C(5''')); 68.70(C(3')); 68.03 (C(3''')); 67.58 (C(6')); 65.36 (C(4''')); 63.58 (C(5'')); 62.99 (C(5')); 60.92 (C(2')); 58.96 (C(3)); 57.37 (C(1)); 56.2 (C(2''')); 50.10 (C(6''')); 30.36 (C(2)); 20.64, 20.57, 20.50, 20.42, 20.39, 20.17 (6q, 6 Me). HR-MALDI-MS (*m*/*z*): 1168.3174 (100, $[M + Na]^+$, $C_{43}H_{51}N_{15}NaO_{23}^+$; calc. 1168.3180).

1.3. Synthesis of Acetals 119 and 120.

*(2S,3S,4R,5R,6R)-5-azido-2-(azidomethyl)-6-((2R,3S,4R,5S)-5-((1R,2R,3S,5R,6S)-3,5-diazido-2-((1R,2S)-2-azido-3-hydroxy-1-((2R,5R)-2-phenyl-1,3-dioxan-5-yloxy)propoxy)-6-hydroxycyclohexyloxy)-4-hydroxy-2-(hydroxymethyl)tetrahydrofuran-3-yloxy)tetrahydro-2H-pyran-3,4-diol (**119**)* and *(2S,3S,4R,5R,6R)-5-azido-2-(azidomethyl)-6-((2R,3S,4R,5S)-5-((1R,2R,3S,5R,6S)-3,5-diazido-2-((1R,2S)-2-azido-3-hydroxy-1-(2-phenyl-1,3-dioxan-5-yloxy)propoxy)-6-hydroxycyclohexyloxy)-4-hydroxy-2-(hydroxymethyl)tetrahydrofuran-3-yloxy)tetrahydro-2H-pyran-3,4-diol (**120**).* A soln. of **45** (1g, 1.34 mmol) in a minimum amount of 50% aq. EtOH was treated with $NaIO_4$ (0.52 g, 2 mmol) at 0° C. The mixture was stirred for 20 h, treated with ethylene glycol (0.5 ml), filtered, taken to dryness and dissolved in 50% aq. EtOH. A soln. of $NaBH_4$ (0.11g, 2.94

mmol) in 50% aq. EtOH was treated with the soln. of the crude at 0° C, stirred for 2h at 0° C, neutralized with 10% aq. AcOH, and taken to dryness. FC ($CHCl_3$/EtOAc/MeOH 3:3:1) R_f 0.33 gave 500 mg **118** (50%). Without further characterization, at 0° C and under Ar, a soln. of **118** (500 mg, 0.67 mmol) in freshly distilled benzaldehyde (5 ml) was treated dropwise with formic acid (0.9 ml) and stirred at 0° C for 16h. The pH was adjusted to 7 at 0° C with 1M aq. NaOH. The mixture was extracted with EtOAc (3× 20 ml). The combined organic layers were washed with brine (50 ml), dried ($NaSO_4$), filtered, and evaporated. FC (hexane to hexane/EtOAc 9:1 to remove benzaldehyde, then $CHCl_3$/EtOAc 1:1 to $CHCl_3$/EtOAc/MeOH 3:3:0.4) gave a 3:2 mixture of **119/120** (0.45g, 80 %), white solids. R_f ($CHCl_3$/EtOAc/MeOH 3:3:0.5) 0.52. The acetals were separated by FC (EtOAc/hexane 3:1) to give **119** (252 mg 45%) and **120** (169 mg, 30%.

Data of **119**: ^{1}H-NMR (400 MHz, CD_3OD): δ 7.43–7.40 (m, H–C(3)/H–C(5) of Ph); 7.33–7.27 (m, H–C(2)/H–C(4)/H–C(6) of Ph); 5.42 (s, PhCH); 5.34 (d, *J* = 4.7, H–C(1’)); 5.14 (d. *J* = 3.1, H–C(1’’)); 5.11 (d, *J* = 1.8, H–C(1’’’)); 4.50 (br. s, HO–C(3’)); 4.48 (m, H_a–C(4’) or H_a–C(6’)); 4.41 (m, H_a–C(6’) or H_a–C(4’)); 4.22 (dd, *J* = 4.7, 3.1, H–C(2’’)); 4.11–4.02 (m, H–C(5’), H–C(4’’)); 3.97 (ddd, *J* = 8.7, 4.4, 1.9, H–C(5’’’)); 3.87 (dd, *J* = 2.8, 2.8, H–C(3’’’)); 3.80 (dd, *J* = 12-0, 2.7, H_a–C(5’’)); 3.37 (dd, *J* = 3.4, 1.9 H–C(4’’’)); 3.73 (m, H_a–C(3’)); 3.72–3.44 (m, H–C(2’), H_b–C(3’), H_b–C(4’), H_b–C(6’), H–C(3), H–C(4), H–C(5), H_b–C(5’’), H–C(2’’’), H_a–C(6’’’)); 3.42–3.30 (dd, *J* = 12.6, 4.2, H–C(1)); 3.40 (dd, *J* = 9.7, 9.7, H–C(6)); 3.29 (dd, *J* = 13.0, 4.4, H_b–C(6’’’)); 2.03 (ddd, *J* = 13.0, 4.2, 4.2, H_{eq}–C(2)); 1.22 (ddd, *J* = 13.0, 12.6, 12.6, H_{ax}–C(2)). ^{13}C-NMR (100 MHz, CD_3OD): δ 139.30 (s, C(1) of Ph); 129.91 (d, C(4) of Ph); 129.12 (d, C(3)/C(5) of Ph); 127.40 (d, C(2)/C(6) of Ph); 110.69 (C(1’’)); 103.76 (C(1’)); 102.48 (d, PhCH); 100.04 (C(1’’’)); 86.02 (C(5)); 83.76 (C(4’’)); 80.48 (C(4)); 77.47 (C(6)); 77.43 (C(3’’)); 75.41 (C(2’’)); 75.66 (C(5’’’)); 71.45, 70.98 (C(4’), C(6’)); 71.11 (C(3’’’)); 70.35 (C(5’)); 69.58 (C(4’’’)); 68.28 (C(2’)); 63.57 (C(5’’)); 61.96 (C(3’)); 61.87 (C(1)); 61.73 (C(2’’’)); 61.06 (C(3)); 52.47 (C(6’’’)); 33.20 (C(2)); This compound taken forward to the preparation of **36** without further characterization.

Data of **120**: ^{1}H-NMR (400 MHz, CD_3OD): 5.40 (d, *J* = 5.1, H–C(1’)); 5.14 (d *J* = 2-7, H–C(1’’)); 4.55–4.45 (br. s, HO–C(3’)); 4.28 (m, H_a–C(4’), H_a–C(6’)); 4.25 (dd, *J* = 4.8, 2.7, H–C(2’’));

4.28 (m, H_a–C(6’)); 4.11 (m, H_b–C(4’) or H_b–C(6’)); 4.04 (H_b–C(6’) or H_b–C(4’)); 4.10–4.05 (m, H–C(4’’)); 4-01 (dd, *J* = 9.5, 9.5, H–C(4)); 3.98 (ddd, *J* = 8.6, 4.3, 20., H–C(5’’’)); 3.89 (dd, *J* = 3.4, 3.4, H–C(3’’’)); 3.86 (dd, *J* = 11.7, 8.0, H_b–C(3’)); 3.79 (dd, *J* = 12.0, 2.7, H_a–C(5’’)); 3.78 (m, H–C(5’)); 3.73 (dd, *J* = 11.7, 4.0, H_a–C(3’)); 3.67–3.59 (m, H–C(2’), H–C(2’), H_b–C(5’’), H–C(2’’’), H_a–C(6’’’)); 3.48 (dd, *J* = 9.5, 9.1, H–C(5), 3.47 (ddd, *J* = 12.5, 9.5, 4.4, H–C(3)); 3.39 (dd, *J* = 3.4, 2.0, H–C(4’’’)); 3.35 (dd, *J* = 13.0, 3.2, H_b–C(6’’’), 3.33–3.25 (H–C(1), 1.98 (ddd, *J* = 13.0, 4.4, 4.24, H_{eq}–C(2)); 1.22 (ddd, *J* = 13.0, 12.5, 12.5, H_{ax}–C(2)). ^{13}C-NMR (100 MHz, CD_3OD): δ 140.05 (s, C(1) of Ph); 130.04 (d, C(4) of Ph); 129.10 (d, C(3)/C(5) of Ph); 127.76 (d, C(2)/C(6) of Ph); 111.59 (C(1’’)); 102.88 (d, PhCH);101.05 (C(1’)); 99.91 (C(1’’’)); 86.47 (C(5)); 83.60 (C(4’’)); 78.82 (C(4)); 77.46 (C(6)); 77.20 (C(3’’)); 75.56 (C(5’’’)); 75.24 (C(2’’)); 71.09 (C(3’’’)); 70.91, 69.45 (C(4’), C(6’)); 71.54 (C(5’)); 69.61 (C(4’’’)); 68.92 (C(2’)); 63.36 (C(5’’)); 62.19 (C(3’)); 61.93 (C(1)); 61.76 (C(2’’’)); 61.96 (C(3)); 52.53 (C(6’’’)); 33.24 (C(2)). HR-MALDI-MS (*m*/*z*): [*M*+H]$^+$, 858.2824 [*M*+Na]$^+$.

1.4. General Procedure for the Deacetylation of the Hexaacetates 61–64, **66**, **67**, **and 69–80**.

A soln. of the hexaacetates in CH_2Cl_2/MeOH 1:4 was treated with NaOMe (12 equiv.), and stirred at 25°. The mixture was treated with *Amberlite IR-120 (H^+)*, filtered, and evaporated. FC ($CHCl_3$/AcOEt/MeOH 1:1:0 → 3:3:0.3) gave **81–84**, **86**, **87**, and **89–100**, respectively.

1.5. Deacetylation of the Hexaacetates 65, **68**, **101**, and **103.**

1,3,2’,2”’,6”’-Pentadeamino-1,3,2’,2”’,6”’-pentaazido-4’,6’-O-(3-hydroxybenzylidene)paromomycin (**85**). A soln. of **65** (300 mg, 0.27 mmol) in MeOH (2 ml) at 0° was treated with a soln. of $MeNH_2$ (33% in EtOH, 30 ml), and stirred for 1 h at 0° and for 6 h at 26°. Evaporation and FC ($CHCl_3$/AcOEt/MeOH 6:6:0.2 to 6:6:1) gave **85** (141 mg, 63%). White solid. *R*f ($CHCl_3$/AcOEt/MeOH 6:6:1) 0.30. IR (ATR): 3426*w* (br.), 2924*w*, 2105*s*, 1458*w*, 1371*w*, 1261*w*, 1082*w*, 1033*w*, 765*w*. $[\alpha]_D^{25}$ = +110.0 (*c* = 0.4, MeOH). ^{1}H-NMR (400 MHz, CD_3OD): δ 7.44 (dd, *J* = 7.6, 1.6, 1 arom. H); 7.17 (ddd, *J* = 8.2, 7.4, 1.8, 1 arom. H); 6.85-6.78 (*m*, 2 arom. H); 5.90 (br. s, ArC*H*); 5.83 (d, *J* = 3.6, H–C(1’)); 5.40 (*d*, *J* = 2.0, H–C(1”)); 5.14 (d, *J* = 1.8, H–C(1’’’)); 4,43 (dd, *J*

= 6.4, 4.8, H–C(3'')); 4.32 (dd, J = 4.8, 2.0, H–C(2'')); 4.21 (dd, J = 9.6, 4.8, H_a–C(6')); 4.19–4.09 (m, H–C(3'), H–C(5'), H–C(4'')); 4.02 (ddd, J = 8.4, 4.5, 2.0, H–C(5''')); 3.94 (dd, J = 3.6, 3.6, H–C(3''')); 3.85 (dd, J = 12.0, 2.8, H–C(5'')); 3.76 (dd, J = 10.0, 9.9, H_b–C(6')); 3.73–3.64 (m, H–C(4), H–C(5), H_b–C(5''), H–C(2'''), H_a–C(6''')); 3.58–3.42 (m, H–C(45'), H–C(1), H–C(3), H–C(6), H–C(4''')); 3.40 (dd, J = 12.8, 4.5, H_b–C(6''')); 3.25 (dd, J = 10.0, 3.6. H–C(2')); 2.22 (ddd, J = 12.8, 4.2, 4.2, H_{eq}–C(2)); 1.41 (ddd, J = 12.8, 12.4, 12.4, H_{ax}–C(2)). ^{13}C-NMR (100 MHz, CD_3OD): δ 155.19 (*s*), 131.13, 128.63 (2 *d*); 124.92 (*s*), 120.34, 116.57 (2 *d*); 109.66 (C(1'')); 99.79 (*d*, Ar*C*H); 99.71 (C(1''')); 99.10 (C(1')); 85.21 (C(5)); 83.45 (C(4'')); 83.14 (C(4')); 77.52 (C(4)); 77.31 (C(3'')); 77.21 (C(6)); 75.59 (C(5''')); 75.16 (C(2'')); 71.11 (C(3''')); 69.62 (C(4''')); 69.56 (C(3')); 69.93 (C(6')); 65.14 (C(2')); 64.58 (C(5')); 63.62 (C(5'')); 61.82 (C(2''')); 61.82 (C(1)); 61.29 (C(3)); 52.46 (C(6''')); 32.98 (C(2)). HR-MALDI-MS (*m*/*z*) (negative mode): 848.2677 (100, $[M-H]^-$, $C_{30}H_{38}N_{15}O_{15}^-$; calc. 848.2672).

*1,3,2',2''',6'''-Pentadeamino-1,3,2',2''',6'''-pentaazido-4',6'-*O-(*3-bromo-4-hydroxybenzylidene)paromomycin* (**88**). Under N_2, a soln. of **68** (300 mg, 0.25 mmol) in 0.02N MeONa/MeOH was stirred for 15 h at 26°, treated with *Amberlite IR-120 (*H+ form*)*, filtered, and evaporated. FC ($CHCl_3$/AcOEt/MeOH 4:4:0.1 → 6:6:1) gave **88** (189 mg, 80%). White solid. *R*f (CHCl3/AcOEt/MeOH 8:8:1) 0.30. IR (ATR): 3386*w* (br.), 2934*w*, 2098*s*, 1610*w*, 1503*w*, 1368*w*, 1259*w*, 1084*w*, 1025*w*, 822*w*, 820*w*. ^{1}H-NMR (400 MHz, CD_3OD): δ 7.60 (dd J = 2.0, 1 arom. H); 7.28 (d, J = 8.5, 2.0, 1 arom. H); 6.87 (d, J = 8.5, 1 arom. H); 5.81 (d, J = 3.6, H–C(1')); 5.50 (br. s, ArC*H*); 5.39 (d, J = 2.0; H–C(1'')); 5.14 (d, J = 2.0, H–C(1''')); 4.43 (dd J = 6.8, 4.8, H–C(3'')); 4.32 (dd, J = 4.8, 2.0, H–C(2'')); 4.20 (dd, J = 10.0, 5.0, H_a–C(6')); 4.19–4.08 (m, H–C(3'), H–C(4''), H–(5'),); 4.03 (ddd, J = 8.4, 4.5, 2.0, H–C(5''')); 3.95 (dd, J = 3.4, 3.4, H–C(3''')); 3.85 (m, H_a–C(5'')); 3.75–3.63 (m, H_b–C(6') H–C(4), H–C(5), H–C(5''), H–C(2'''), H_a–C(6''')); 3.55–3.42 (m, H–C(4'), H–C(1), H–C(3), H–C(6), H–C(4''')); 3.40 (dd, J = 12.8, 4.5, H_b–C(6''')); 3.24 (dd, J = 10.0, 3.6, H–C(2')); 2.22 (ddd, J = 12.8, 4.4, 4.4, H_{eq}–C(2)); 1.41 (ddd, J = 12.8, 12.4, 12.3, H_{ax}–C(2)). ^{13}C-NMR (100 MHz, CD_3OD. δ 155.87 (s); 132.27 (d); 131.72 (s); 127.82, 116.59 (2 d); 110.22 109.55 (s); 102.03 (d, Ar*C*H); 110.22 (C(1'')); 99.69 (C(1''')); 99.04 (C(1')); 85.10 (C(5)); 83.35 (C(4'')); 82.74 (C(4')); 77.68 (C(4)); 77.22 (C(3'')); 77.11 (C(6)); 75.51 (C(5''')); 75.07 (C(2'')); 71.00 (C(3'''));

69.64 (C(6')); 69.54 (C(4''')); 69.43 (C(3')); 65.05 (C(2')); 64.39 (C(5')); 63.54 (C(5'')); 61.73 (2 C, C(1), C(2''')); 61.13 (C(3)); 52.37 (C(6''')); 32.85 (C(2)). HR-MALDI-MS (*m*/*z*): 968.1467 (40, $[M + K]^+$, $C_{30}H_{38}{}^{81}BrN_{15}KO_{15}{}^+$; calc. 968.1492), 966.1461 (33, $[M + K]^+$, $C_{30}H_{38}{}^{79}BrKN_{15}O_{15}{}^+$; calc. 966.1492), 952.1732 (100, $[M + Na]^+$, $C_{30}H_{38}{}^{81}BrN_{15}NaO_{15}{}^+$; calc. 952.1753), 950.1748 (94, $[M + Na]^+$, $C_{30}H_{38}{}^{79}BrN_{15}NaO_{15}{}^+$; calc. 950.1753).

1,3,2',2''',6'''-Pentadeamino-1,3,2',2''',6'''-pentaazido-4',6'-O-(3-formyl-4-hydroxy-benzylidene)paromomycin (**104**). A soln. of **101** (180 mg, 0.16 mmol) in MeOH/H_2O 5:1 (6 ml) was treated with K_2CO_3 (396 mg, 2.87 mmol), stirred for 12 h at 26°, filtered, and evaporated. A suspension of the residue in AcOEt was filtered, and the filtrate was washed with brine, dried (Na_2SO_4), filtered, and evaporated. FC ($CHCl_3$/AcOEt/MeOH 10:10:0.25 → 10:10:1) gave **104** (102 mg, 73%). White solid. *R*f (CHCl3/AcOEt/MeOH 6:6:1) 0.32. IR (ATR): 3392*w* (br.), 2928*w*, 2103*s*, 1490*w*, 1371*w*, 1277*w*, 1087*w*, 1030*m*, 771*w*. ^{1}H-NMR (400 MHz, CD_3OD): δ 10.03 (s, (CHO)); 7.84 (d, *J* = 2.0, (1 arom. H)); 7.67 (dd, *J* = 8.8, 2.4, (1 arom. H)); 6.95 (d, *J* = 8.8, (1 arom. H)); 5.83 (d, *J* = 3.6, H–C(1')); 5.60 (br. s, (ArC*H*)); 5.40 (d, *J* = 1.8; H–C(1'')); 5.14 (d, *J* = 1.8, H–C(1''')); 4.44 (dd *J* = 6.4, 4.4, H–C(3'')); 4.32 (dd, *J* = 4.4, 1.8, H–C(2'')); 4.23 (dd, *J* = 10.0, 5.0, H_a–C(6')); 4.18–4.09 (m, H–C(3'), H–C(4''), H–(5')); 4.03 (ddd, *J* = 8.4, 4.5, 2.0, H–C(5''')); 3.94 (dd, *J* = 3.6, 3.6, H–C(3''')); 3.84 (dd, *J* = 12.0, 2.8, H_a–C(5'')); 3.77 (dd, *J* = 10.0, 10.0, H_b–C(6')); 3.73–3.64 (m, H_b–C(6') H–C(4), H–C(5), H–C(5''), H–C(2'''), H_a–C(6''')); 3.58–3.42 (m, H–C(4'), H–C(1), H–C(3), H–C(6), H–C(4''')); 3.40 (dd, J = 12.8, 4.5, H_b–C(6''')); 3.25 (dd, *J* = 10.5, 3.6, H–C(2')); 2.22 (ddd, *J* = 12.8, 4.4, 4.4, H_{eq}–C(2)); 1.42 (ddd, *J* = 12.8, 12.4, 12.3, H_{ax}–C(2)). ^{13}C-NMR (100 MHz, CD_3OD):δ 196.93 (s, CHO), 162.94 (s), 135.98, 131.92 (2 d), 131.16, 122.06 (2 s); 117.97 (d); 101.98 (d, Ar*C*H). 109.70 (C(1'')); 99.78 (C(1''')); 99.10 (C(1')); 85.22 (C(5)); 83.45 (C(4'')); 82.89 (C(4')); 77.76 (C(4)); 77.29 (C(3'')); 77.23 (C(6)); 75.61 (C(5''')); 75.15 (C(2'')); 71.13 (C(3''')); 69.77 (C(6')); 69.63 (C(4''')); 69.55 (C(4''')); 65.18 (C(2')); 64.49 (C(5')); 63.64 (C(5'')); 61.84 (2 C, C(1), C(2''')); 61.22 (C(3)); 52.48 (C(6''')); 32.95 (C(2)). HR-MALDI-MS (*m*/*z*) (negative mode): 876.2615 (100, $[M - H]^-$, $C_{31}H_{38}N_{15}O_{16}{}^-$; calc. 876.2621).

1,3,2',2''',6'''-Pentadeamino-1,3,2',2''',6'''-pentaazido-4',6'-O-(3-carboxy-4-hydroxy-

benzylidene)paromomycin (**105**). A soln. of **103** (40 mg, 35 μmol) in MeOH (0.4 ml) was cooled to 0°, treated with a soln. of $MeNH_2$ (33% in EtOH, 3 ml) and stirred for 1 h at 0° and for 6 h at 26°. Evaporation and FC (AcOEt/MeOH 85:15 to 3:1) gave **105** (20 mg, 63%). White solid. R_f (AcOEt/MeOH 3:1) 0.46. IR (ATR): 3373*w* (br.), 2923*w*, 2102*s*, 1632*w*, 1575*w*, 1498*w*, 1452*w*, 1371*m*, 1257*m*, 1083*w*, 1029*w*, 832*w*. ^{1}H-NMR (400 MHz, $CDCl_3$): δ 8.00 (dd, *J* = 2.0, 1 arom. H); 7.43 (dd, *J* = 8.4, 2.4, 1 arom. H); 6.79 (d, *J* = 8.4, 1 arom. H); 5.52 (br. s, ArC*H*). 5.84 (*d*, *J* = 3.7, H–C(1')), 5.54 (dd, *J* = 10.7, 9.2, H–C(3')); 5.34 (d, *J* = 2.2; H–C(1'')); 5.03 (dd, *J* = 2.8, 2.4, H–C(3''')); 4.94 (dd, *J* = 4.8, 2.1, H–C(2'')); 4.93 (dd, *J* = 9.7, 8.7, H–C(6)); 4.89 (d, *J* = 1.9, H–C(1''')); 4.70 (dd, *J* = 2.8, 1.0, H–C(4''')); 4.44–4.42 (dd, *J* = 6.8, 54.8, H–C(3'')); 4.42 (dd, *J* = 11.9, 2.5, H_a–C(5'')); 4.30 (ddd, *J* = 6.8, 5.8, 2.5, H–C(4'')); 4.21 (dd, *J* = 11.9, 5.8, H_b–C(5'')); 4.11 (ddd, J = 8.3, 4.1, 1.0, H–C(5''')); 4.09 (ddd, *J* = 9.6, 2.6, 2.6, H–C(5')); 3.89 (dd, *J* = 8.7, 7.9, H–C(5)); 3.83 (m, H_a–C(6')); 3.78 (m, H_b–C(6')); 3.68 (dd, J = 9.6, 9.2, H–C(4')); 3.67 (dd, *J* = 9.3, 7.9, H–C(4)); 3.61 (dd, *J* = 13.0, 8.3; H_a–C(6''')); 3.49 (ddd, *J* = 12.3, 9.3, 4.7, H–C(3)); 3.45 (ddd, *J* = 12.4, 9.7, 4.6, H–C(1)); 3.32 (dd, *J* = 2.4, 1.9, H–C(2''')); 3.24 (dd, *J* = 13.0, 4.1, H_b–C(6''')); 2.98 (dd, *J* = 10.7, 3.7, H–C(2')); 2.35 (ddd, *J* = 13.3, 4.7, 4.6, H_{eq}–C(2)); 1.58 (ddd, *J* = 13.3, 12.4, 12.3, H_{ax}–C(2)). ^{13}C-NMR (100 MHz, $CDCl_3$):, δ 176.30 (s, CO_2H); 163.22, 136.08 (2 s); 132.06, 130.21 (2 d); 119.29 (s); 116.95 (d); 103.25 (d, Ar*C*H)); 106.96 (C(1'')); 99.04 (C(1''')); 96.45 (C(1')); 81.69 (C(5)); 79.12 (C(4'')); 76.16 (C(4)); 75.94 (C(3'')); 75.41 (C(4')); 74.28 (C(6)); 74.55 (C(2'')); 73.69 (C(5''')); 71.87 (C(5')); 71.81 (C(3')); 68.73 (C(3''')); 65.75 (C(4''')); 64.20 (C(5'')); 61.31 (C(2')); 61.17 (C(6')); 59.12 (C(3)); 57.99 (C(1)); 56.45 (C(2'')); 50.66 (C(6''')); 31.19 (C(2)).HR-MALDI-MS (*m*/*z*) (negative mode): 892.2573 (100, $[M – H]^–$, $C_{30}H_{39}N_{15}O_{17}^–$; calc. 892.2570).

1.6. Procedures for the Staudinger Reduction of the Azido Groups.

A. Under N_2, a soln. of the azide in THF was treated with 0.1M aq. NaOH (2 equiv.) and 1M aq. PMe_3 in THF (6 equiv.), stirred at 60°, and evaporated. FC (THF, THF/MeOH, MeOH, MeOH/25% aq. NH_3 49:1 → MeOH/25% aq. NH_3 4:1) gave the amine.

B. A 0.03 M soln. of the azide in MeOH was treated with Et_3N (25 equiv.) and propane-1,3-dithiol (25 equiv.), stirred at 25° for 1-4 days, and evaporated. A soln. of residue in H_2O was washed

with AcOEt. Evaporation of the aq. layer gave the crude amine.

C. A 0.02 M solution of the azide (1 equiv.) in THF was treated with 1M NaOH (0.5 ml) and 1 M aq. PMe_3 in THF (6 equiv.), stirred for 2–4 h at 50°, and evaporated. A soln. of the residue in water was washed with EtOAc, and evaporated to give the crude amine.

*4',6'-*O*-Benzylideneparomomycin* (**1**). According to Staudinger protocol **A**, reaction of 110 mg of **46** and FC gave 73 mg of **1** (79%). White solid. R_f (MeOH/25% aq. NH_3 4:1) 0.30. $[\alpha]_D^{25}$ = +43.2 (c = 0.25, H_2O). IR (ATR): 3355*m*, 3290*m*, 2909*m*, 1589*m*, 1455*m*, 1378*m*, 1334*m*, 1023*s*, 978*s*, 927*m*. ^{1}H-NMR (500 MHz, D_2O): δ 7.57–7.55 (m, 2 arom. H); 7.50–7.48 (m, 3 arom. H); 5.58 (s, ArCH); 5.55 (d, J = 3.8, H–C(1')); 5.40 (d, J = 2.3, H–C(1")); 5.04 (d, J = 1.7, H–C(1"')); 4.44 (dd, J = 6.6, 5.0, H–C(3")); 4.35–4.31 (m, H–C(2"), H_a–C(6')); 4.18–4.13 (m, H–C(4"), H–C(5"')); 4.10–4.04 (m, H–C(3"'), H–C(5')); 3.94–3.87 (m, H_a–C(5"), H–C(3')); 3.91 (t, J = 10.5, H_b–C(6')); 3.77–3.73 (m, H–C(4'), H–C(5)); 3.74 (dd, J = 10.0, 5.2, H_b–C(5")); 3.71–3.70 (m, H–C(4"')); 3.54 (t, J = 9.3, H–C(4)); 3.40 (t, J = 9.7, H–C(6); 3.28 (dd, J = 13.6, 7.9, H_a–C(6"')); 3.21 (dd, J = 13.5, 3.9, H_b–C(6"')); 3.13 (br. s, H–C(2"')); 3.04–3.00 (m, H–C(3)); 2.99 (dd, J = 10.0, 3.9, H–C(2')); 2.94–2.89 (m, H–C(1)); 2.07 (dt, J = 13.0, 4.1, H_{eq}–C(2)); 1.34 (q, J = 12.5, H_{ax}–C(2)). ^{13}C-NMR (126 MHz, D_2O): δ 138.79 (s); 132.61 (d); 131.43 (2d); 128.91 (2d); 111.35 (d, C(1")); 104.52 (d, ArCH); 101.83 (d, C(1')); 101.18 (d, C(1"')); 86.96 (d, C(5)); 84.46 (d, C(4)); 83.89 (d, C(4")); 83.52 (d, C(4')); 78.88 (d, C(6)); 78.06 (d, C(3")); 76.05 (d, C(2")); 74.61 (d, C(5"')); 72.73 (d, C(3"')); 72.56 (d, C(3')); 71.11 (d, C(4"')); 70.72 (t, C(6')); 66.32 (d, C(5')); 63.38 (t, C(5")); 58.55 (d, C(2')); 54.87 (d, C(2"')); 53.02 (d, C(1)); 52.24 (d, C(3)); 43.41 (t, C(6"')); 36.81 (t, C(2)). HR-MALDI-MS (m/z): 726.3117 (31, $[M + Na]^+$, $C_{30}H_{49}N_5NaO_{14}^+$; calc. 726.3174); 705.3347 (37); 704.3336 (100, $[M + H]^+$, $C_{30}H_{50}N_5O_{14}^+$; calc. 704.3349). Anal. calc. for $C_{30}H_{49}N_5O_{14}$ (703.74): C 51.20, H 7.02, N 9.95; found: C 51.30, H 7.15, N 9.98.

*4',6'-*O*-(4-Chlorobenzylidene)paromomycin* (**2**). According to Staudinger protocol **A**, reaction of 17 mg of **47** and FC gave 12 mg of **2** (83%). White solid. R_f (MeOH/25% aq. NH_3 4:1) 0.36. IR (ATR): 3349*m*, 3285*m*, 2921*m*, 1738*w*, 1598*w* 1495*w*, 1456*w*, 1373*m*, 1133*s*, 1104*s*, 1085*s*, 1014*s*, 996*s*. ^{1}H-NMR (500 MHz, D_2O): δ 7.50–7.44 (m, 4 arom. H); 5.73 (s, ArCH); 5.49 (d, J = 3.8, H–

C(1')); 5.35 (d, J = 2.3, H–C(1")); 4.99 (d, J = 1.8, H–C(1"')); 4.45 (dd, J = 6.6, 5.0, H–C(3")); 4.31–4.28 (m, H–C(2")); 4.28 (dd, J = 12.7, 4.8, H_a–C(6')); 4.14–4.10 (m, H–C(4")); 4.08 (m, H–C(5"')); 4.02 (t, J = 3.3, H–C(3"')); 4.03–3.98 (td, J = 9.9, 4.8, H–C(5')); 3.90–3.80 (m, H_b–C(6'), H_a–C(5")); 3.83 (t, J = 9.0, H–C(3')); 3.74–3.60 (m, H–C(5), H–C(4'), Hb–C(5"), H–C(4"')); 3.48 (t, J = 9.3, H–C(4)); 3.33 (t, J = 9.7, H–C(6)); 3.20 (dd, J = 13.5, 8.0, H_a–C(6"')); 3.12 (dd, J = 13.5, 3.9, H_b–C(6"')); 3.07–3.06 (m, H–C(2"')); 2.99–2.91 (m, H–C(3)); 2.92 (dd, J = 10.0, 3.8, H–C(2')); 2.85–2.79 (m, H–C(1)); 2.00 (dt, J = 12.9, 4.2, H_{eq}–C(2)); 1.26 (q, J = 12.6, H_{ax}–C(2)). ^{13}C-NMR (126 MHz, D_2O): δ 137.69 (s); 137.50 (s); 131.40 (2d); 130.51 (2d); 111.28 (d, C(1")); 103.69 (d, ArCH); 101.99 (d, C(1')); 101.38 (d, C(1")); 86.97 (d, C(5)); 84.71 (d, C(4)); 83.91 (d, C(4")); 83.53 (d, C(4')); 79.27 (d, C(6)); 78.14 (d, C(3")); 76.06 (d, C(2")); 75.16 (d, C(5"')); 72.87 (d, C(3')); 72.70 (d, C(3"')); 71.15 (d, C(4"')); 70.72 (t, C(6')); 66.25 (d, C(5')); 63.49 (t, C(5")); 58.62 (d, C(2')); 54.97 (d, C(2"')); 52.99 (d, C(1)); 52.31 (d, C(3)); 43.47 (t, C(6"')); 37.21 (t, C(2)). HR-MALDI-MS (*m*/*z*): 738.2974 (95, $[M + H]^+$, $C_{30}H_{49}ClN_5O_{14}^+$; calc. 738.2965).

*4',6'-*O*-(4-Methoxybenzylidene)paromomycin* (**3**). According to Staudinger protocol **A**, reaction of 48 mg of **48** and FC gave 38 mg of **3** (93%). White solid. R_f (MeOH/25 % aq. NH_3 4:1) 0.32. $[\alpha]_D^{25}$ = +43.2 (c = 0.25, H_2O). IR (ATR): 3355*w*, 2917*w*, 1614*w*, 1518*w*, 1461*w*, 1378*w*, 1302*w*, 1250*w*, 1101*s*, 1024*s*, 937*m*. ^{1}H-NMR (300 MHz, D_2O): δ 7.50 (*d*, J = 8.8, 2 arom. H); 7.04 (*d*, J = 8.8, 2 arom. H); 5.73 (*s*, ArC*H*); 5.65 (*d*, J = 4.0, H–C(1')); 5.40 (*d*, J = 2.2, H–C(1")); 5.17 (*d*, J = 1.6, H–C(1"')); 4.53 (*dd*, J = 6.5, 4.7, H–C(3")); 4.39 (*dd*, J = 4.7, 2.2, H–C(2")); 4.34–4.22 (*m*, 2 H); 4.22–4.14 (*m*, 2 H); 4.05–3.70 (*m*, 7 H); 3.85 (*s*, MeO); 3.61 (*t*, J = 9.6, H–C(4)); 3.56 (*t*, J = 10.3, H–C(6)); 3.43–3.20 (*m*, 3 H); 3.23–3.02 (*m*, 3 H); 2.19 (*dt*, J = 12.8, 4.0, H_{eq}–C(2)); 1.49 (*q*, J = 12.5, H_{ax}–C(2)). ^{13}C-NMR (75 MHz, D_2O): δ 159.62 (s); 128.65 (s); 127.60 (2d); 113.91(2d); 109.20 (d, C(1")); 101.53 (d, ArCH); 97.92, 96.71 (2d, H–C(1'), H–C(1"')); 84.39 (d, C(5)); 81.02, 80.81, 80.32 (3d, C(4), C(4"), C(4')); 74.96 (d, C(6)); 74.17 (d, C(3")); 73.19 (d, C(2")); 70.63 (d, C(5"')); 68.82, 68.56 (2d, C(3'), C(3"')); 67.75 (d, C(4"'), t, C(6')); 63.62 (d, C(5')); 60.29 (t, C(5")); 55.30 (d, C(2'), q, CH3O); 51.46 (d, C(2"')); 50.40, 49.11 (2d, C(1), C(3)); 40.49 (t, C(6"')); 32.24 (t, C(2)). HR-MALDI-MS (*m*/*z*): 735.3475 (36); 734.3442 (100, $[M + H]^+$, $C_{31}H_{52}N_5O_{15}^+$; calc. 734.3454).

*4',6'-*O*-[4-(Dimethylamino)benzylidene]paromomycin* (**4**). According to the general acetalization protocol **B**, reaction of 154 mg **60** for 8 h and FC gave 80 mg of **61** (46%). Yellowish solid. R_f (cyclohexane/AcOEt 1:1) 0.48. ^{1}H-NMR (300 MHz, $CDCl_3$): δ 7.34–7.26 (m, 2 arom. H); 6.73–6.64 (m, 2 arom. H); 5.84 (d, *J* = 3.7, H–C(1')); 5.55 (t, *J* = 10.0, H–C(3')); 5.42 (s, ArCH); 5.34 (d, *J* = 2.2, H–C(1")); 5.03 (t, *J* = 2.8, H–C(3"')); 4.93 (t, *J* = 10.0, H–C(6)); 4.94–4.86 (m, H–C(2"), H–C(1"')); 4.70 (br. s, H–C(4"')); 4.46–4.38 (m, H_a–C(5"), H–C(3")); 4.33–4.18 (m, H–C(4"), H–C(5'), H_a–C(6'), H_b–C(5")); 4.14–4.07 (m, H–C(5"')); 3.90 (t, *J* = 9.0, H–C(5)); 3.68–3.35 (m, H_b–C(6'), H–C(4), H–C(4'), H_a–C(6"'), H–C(3), H–C(1)); 3.32 (br. s, H–C(2"')); 3.25 (dd, *J* = 13.1, 4.1, H_b–C(6"')); 3.05 (dd, *J* = 10.6, 4.1, H–C(2')); 2.936, 2.932 (2s, NMe_2); 2.38 (dt, *J* = 13.1, 4.7, H_{eq}–C(2)); 2.172, 2.167, 2.158, 2.12, 2.102, 2.098 (6s, 6 AcO); 1.61 (q, *J* = 13.1, H_{ax}–C(2)). ^{13}C-NMR (75 MHz, $CDCl_3$): δ 170.73, 170.15, 170.01, 169.79, 169.68, 168.53 (6s, 6 C=O); 151.20 (s); 127.16 (2d); 125.06 (s); 111.98 (2d); 107.12 (d, C(1")); 102.27 (d, ArCH); 99.12 (d, C(1"')); 97.68 (d, C(1')); 82.22 (d, C(5)); 79.26 (d, C(4")); 78.92 (d, C(4')); 77.23 (d, C(4)); 75.82 (d, C(3")); 75.39 (d, C(6)); 74.78 (d, C(2")); 73.58 (d, C(5"')); 69.07 (d, C(3')); 68.74, 68.63 (d, C(3"'), t, C(6')); 65.73 (d, C(4"')); 63.63 (t, C(5")); 63.45 (d, C(5')); 61.63 (d, C(2')); 59.00 (d, C(3)); 58.05 (d, C(1)); 56.49 (d, C(2"')); 50.66 (t, C(6"')); 40.53 (q, NMe_2); 31.40 (t, C(2)); 20.90, 20.80, 20.70, 20.58 (4q, 6 Me). Without further characterization, application of the general deacetylation protocol to 39 mg of **61** and FC gave 22 mg of **81** (73%). White solid. R_f ($CHCl_3$/AcOEt/MeOH 3:3.0.5) 0.21. Finally, without further characterization, according to Staudinger protocol **A**, reaction of 16 mg of **81** and FC gave 13.5 mg **4** (99%). White solid. R_f (MeOH/25% aq. NH_3 4:1) 0.27. IR (ATR): 3172*w*, 2899*w*, 1615*w*, 1528*w*, 1454*w*, 1378*w*, 1360*w*, 1100*s*, 1048*s*, 996*s*. ^{1}H-NMR (500 MHz, D_2O): δ 7.44 (d, *J* = 8.9, 2 arom. H); 7.00 (d, *J* = 9.0, 2 arom. H); 5.71 (s, ArCH); 5.71–5.70 (m, H–C(1')); 5.41 (d, *J* = 2.3, H–C(1")); 5.22 (d, *J* = 1.7, H–C(1"')); 4.54 (dd, *J* = 4.9, 6.8, H–C(3")); 4.41 (dd, *J* = 2.3, 4.8, H–C(2")); 4.30 (dd, *J* = 4.9, 10.3, H_a–C(6')); 4.32–4.28 (m, H–C(5"')); 4.22–4.19 (m, H–C(4"')); 4.20 (t, *J* = 3.2, H–C(3"')); 4.07–4.01 (m, H–C(5')); 4.03 (t, *J* =9.9, H–C(3')); 3.92 (dd, *J* = 3.1, 12.4, H_a–C(5")); 3.92–3.87 (m, H_b–C(6')); 3.84 (t, *J* = 9.1, H–C(5)); 3.81–3.79 (br. s, H–C(4")); 3.80–3.73 (m, H_b–C(5"), H–C(4')); 3.65 (t, *J* = 9.4, H–C(4)); 3.61 (t, *J* = 10.3, H–C(6)); 3.44–3.43 (br. s, H–C(2"')); 3.41 (dd, *J* = 7.2, 13.6, H_a–C(6"')); 3.35 (dd, *J* = 3.9, 13.6, H_b–C(6"')); 3.28 (dd, *J* = 4.0, 10.2, H–

C(2’)); 3.20 (ddd, $J = 4.1, 10.4, 12.4$, H–C(3)); 3.13 (ddd, $J = 4.1, 9.7, 12.2$, H–C(1)); 2.90 (s, Me_2N); 2.23 (dt, $J = 12.8, 4.2$, H_{eq}–C(2)); 1.55 (q, $J = 12.6$, H_{ax}–C(2)). ^{13}C-NMR (126 MHz, D_2O): δ 129.98 (2d); 117.33 (2d); 112.25 (d, C(1”)); 104.74 (d, ArCH); 100.32 (d, C(1’)); 99.07 (d, C(1”’)); 87.34 (d, C(5)); 83.85 (d, C(4”)); 83.03 (d, C(4), d, C(4’)); 77.77 (d, C(3”)); 76.59 (d, C(6)); 75.99 (d, C(2”)); 73.29 (d, C(5”’)); 71.24 (d, C(3’), d, C(3”’)); 70.34 (t, C(6’), d, C(4”’)); 66.44 (d, C(5’)); 63.04 (t, C(5”)); 57.84 (d, C(2’)); 54.02 (d, C(2”’)); 53.17 (d, C(3)); 51.75 (d, C(1)); 43.37 (2q, Me2N, t, C(6”’)); 34.53 (t, C(2)). HR-MALDI-MS (*m/z*): 748.3811 (38); 747.3774 (100, $[M + H]^+$, $C_{32}H_{55}N_6O_{14}^+$; calc. 747.3771).

*4’,6’-*O*-(4-Hydroxybenzylidene)paromomycin* (**5**). According to the general deacetylation protocol reaction of 69 mg of **62** and FC gave 41 mg of **82** (91%). White solid. R_f ($CHCl_3$/AcOEt/MeOH 3:3.0.5) 0.25. Without characterization, according to Staudinger protocol **B**, reaction of **82** (0.034 mmol) for 4 d, followed by FC (MeOH/25% aq. NH_3 1:0 → 9:1) and lyophilisation gave 17 mg of **5** (69%). White solid. R_f (MeOH/25% aq. NH_3 4:1) 0.33. IR (ATR): 3290*w* (br.), 2905*w*, 1594*w*, 1518*w*, 1464*w*, 1376*w*, 1255*w*, 1136*s*, 1022*s*. ^{1}H-NMR (500 MHz, D_2O):, δ 7.39 (d, $J = 8.7$, 2 arom. H); 6.88 (d, $J = 8.7$, 2 arom. H); 5.68 (s, ArCH); 5.51 (d, $J = 3.9$, H–C(1’)); 5.39 (d, $J = 2.4$, H–C(1”)); 5.01 (d, $J = 1.9$, H–C(1”’)); 4.48 (dd, $J = 6.6, 4.9$, H–C(3”)); 4.32 (dd, $J = 4.9, 2.4$, H–C(2”)); 4.28 (dd, $J = 10.3, 5.0$, H_a–C(6’)); 4.17–4.14 (m, H–C(4”)); 4.09–4.05 (m, H–C(5”’)); 4.05 (t, $J = 3.2$, H–C(3”’)); 4.03 (td, $J = 10.0, 4.8$, H–C(5’)); 3.93–3.84 (m, H_b–C(6’), H_a–C(5”)); 3.85 (t, $J = 9.8$, H–C(3’)); 3.81–3.64 (m, H–C(5), H_b–C(5”), H–C(4”’)); 3.66 (t, $J = 9.6$, H–C(4’)); 3.52 (t, $J = 9.3$, H–C(4)); 3.34 (t, $J = 9.6$, H–C(6)); 3.22 (dd, $J = 13.5, 8.1$, H_a–C(6”’)); 3.13 (dd, $J = 13.5, 4.0$, H_b–C(6”’)); 3.09–3.08 (m, H–C(2”’)); 2.98 (ddd, $J = 12.3, 9.5, 4.3$, H–C(3)); 2.94 (dd, $J = 10.0, 3.9$, H–C(2’)); 2.87–2.81 (m, H–C(1)); 2.03 (dt, $J = 13.0, 4.1$, H_{eq}–C(2)); 1.29 (q, $J = 12.6$, H_{ax}–C(2)). ^{13}C-NMR (125 MHz, D_2O): δ 130.66 (2d); 118.63 (2d); 111.20 (d, C(1”)); 104.64 (d, ArCH)); 101.49 (d, C(1’), C(1”’)); 86.96 (d, C(5)); 84.66 (d, C(4)); 83.91 (d, C(4”)); 83.56 (d, C(4’)); 79.41 (d, C(6)); 78.20 (d, C(3”)); 76.10 (d, C(2”)); 75.25 (d, C(5”’)); 72.94 (d, C(3’), C(3”’)); 71.20 (t, C(6’), d, C(4”’)); 66.34 (d, C(5’)); 63.51 (t, C(5”)); 58.63 (d, C(2’)); 55.02 (d, C(2”’)); 53.00 (d, C(1)); 52.30 (d, C(3)); 43.50 (t, C(6”’)); 37.33 (t, C(2)). HR-MALDI-MS (*m/z*): 720.3293 (100, $[M + H]^+$, $C_{30}H_{50}N_5O_{15}^+$; calc. 720.3303).

4',6'-O-*(4-Fluorobenzylidene)paromomycin* (**6**). According to Staudinger protocol **A**, reaction of 60 mg of **49** and FC gave 51 mg of **6** (quant.). White solid. R_f (MeOH/25% aq. NH_3 4:1) 0.33. IR (ATR): 3285*w*, 2875*w*, 1606*w*, 1514*w*, 1457*w*, 1376*w*, 1300*w*, 1224*w*, 1047*s*, 1014*s*, 935*m*. ^{1}H-NMR (500 MHz, D_2O): δ 7.54–7.50 (m, 2 arom. H); 7.19–7.13 (m, 2 arom. H); 5.71 (s, ArCH); 5.51 (d, *J* = 3.9, H–C(1')); 5.35 (d, *J* = 2.3, H–C(1")); 5.05 (d, *J* = 1.8, H–C(1"')); 4.46 (dd, *J* = 6.6, 4.9, H–C(3")); 4.32 (dd, *J* = 4.9, 2.3, H–C(2")); 4.18 (br. s, Ha–C(6')); 4.27 (dd, *J* = 10.2, 4.8, H_a–C(6')); 4.18–4.11 (m, H–C(4"), H–C(5"')); 4.07 (t, *J* = 3.2, H–C(3"')); 4.03–3.96 (m, H–C(5')); 3.88–3.82 (m, 3 H, H_a–C(5"), H–C(3'), H_b–C(6')); 3.73–3.63 (m, H_b–C(5"), H–C(5), H–C(4"'), H–C(4')); 3.51 (t, *J* = 9.4, H–C(4)); 3.44 (t, *J* = 10.0, H–C(6)); 3.32–3.21 (m, H_a–C(6"'), H_b–C(6"')); 3.18–3.17 (m, H–C(2"')); 3.01 (dd, *J* = 9.8, 3.9, H–C(2')); 3.00–2.93 (m, 2 H, H–C(3), H–C(1)); 2.08 (dt, *J* = 12.9, 4.1, H_{eq}–C(2)); 1.37 (q, *J* = 12.5, H_{ax}–C(2)). ^{13}C-NMR (126 MHz, D_2O): δ 165.87 (d, ^1J(C,F) = 245.7); 135.04 (d, ^4J(C,F) = 2.9); 131.09 (d, ^3J(C,F) = 8.7, 2 C); 118.18 (d, ^2J(C,F) = 21.9, 2C); 111.64 (d, C(1")); 103.83 (d, ArCH); 101.61 (d, C(1')); 100.55 (d, C(1"')); 86.95 (d, C(5)); 84.34 (d, C(4)); 83.91 (d, C(4")); 83.38 (d, C(4')); 78.06 (d, C(3")); 77.90 (d, C(6)); 76.02 (d, C(2")); 73.97 (d, C(5"')); 72.30 (d, C(3"')); 72.20 (d, C(3')); 70.90 (d, C(4"')); 70.67 (t, C(6')); 66.33 (d, C(5')); 63.37 (t, C(5")); 58.42 (d, C(2')); 54.61 (d, C(2"')); 53.12 (d, C(1)); 52.15 (d, C(3)); 43.35 (t, C(6"')); 35.95 (t, C(2)). HR-MALDI-MS (*m*/*z*): 744.3074 (32, $[M + Na]^+$, $C_{30}H_{48}FN_5NaO_{14}^+$; calc. 744.3079); 723.3262 (34); 722.3242 (100, $[M + H]^+$, $C_{30}H_{49}FN_5O_{14}^+$; calc. 722.3255).

4',6'-O-*(4-Nitrobenzylidene)paromomycin* (**7**). Reaction of 49 mg of **45** at 70° for 2 h according to the general procedure for tranacetalization and FC gave 104 mg **50** (35%). White solid. R_f ($CHCl_3$/AcOEt/MeOH 3:3:0.5) 0.36. ^{1}H-NMR (300 MHz, CD_3OD): 8.24 (d, *J* = 8.9, 2 arom. H); 7.76 (d, *J* = 8.8, 2 arom. H); 5.84 (d, *J* = 3.8, H–C(1')); 5.72 (s, ArCH); 5.39 (d, *J* = 1.8, H–C(1")); 5.13 (d, *J* = 1.7, H–C(1"')); 4.43 (dd, *J* = 6.6, 4.5, H–C(3")); 4.32 (dd, *J* = 4.5, 1.8, H–C(2")); 4.26 (dd, *J* = 9.9, 4.8, H_a–C(6')); 4.19–4.06 (m, H–C(3'), H–C(4"), H–C(5')); 4.62 (ddd, *J* = 8.4, 4.4, 1.9, H–C(5"')); 3.94 (t, *J* = 3.3, H–C(3"')); 3.84 (dd, *J* = 11.3, 2.8, H_a–C(5")); 3.80 (t, *J* = 10.2, 1 H); 3.74–3.34 (m, 11 H); 3.26 (dd, *J* = 10.1, 3.9, H–C(2')); 2.21 (dt, *J* = 12.8, 4.0, H_{eq}–C(2)); 1.42 (q, *J* = 12.1, H_{ax}–C(2)). Without further characterization, according to Staudinger protocol **A**, reaction of 19 mg of **50** gave 12 mg of **7** (74%). White solid. R_f (MeOH/25% aq. NH_3 4:1) 0.24. IR (ATR): 3172*w*, 2900*w*, 1611*w*,

1521*w*, 1349*w*, 1130*s*, 1099*s*, 995*s*. ^{1}H-NMR (500 MHz, D_2O): δ 8.31 (d, *J* = 9.0, 2 arom. H); 7.77 (d, *J* = 8.9, 2 arom. H); 5.88 (s, ArCH); 5.52 (d, *J* = 3.8, H–C(1')); 5.39 (d, *J* = 2.4, H–C(1")); 5.01 (d, *J* = 1.9, H–C(1"')); 4.47 (dd, *J* = 6.5, 5.0, H–C(3")); 4.35 (dd, *J* = 10.2, 4.8, H_a–C(6')); 4.32 (dd, *J* = 4.9, 2.5, H–C(2")); 4.17–4.14 (m, H–C(4")); 4.09–4.03 (m, H–C(5'), H–C(5"')); 4.05 (t, *J* = 3.4, H–C(3"')); 3.93 (t, *J* = 10.4, H_b–C(6')); 3.89 (dd, *J* = 12.5, 3.3, H_a–C(5")); 3.88 (t, *J* = 9.9, H–C(3')); 3.76–3.71 (m, H–C(5), H–C(4'), H_b–C(5")); 3.69–3.67 (m, H–C(4"')); 3.52 (t, *J* = 9.3, H–C(4)); 3.34 (t, *J* = 9.6, H–C(6)); 3.20 (dd, *J* = 13.5, 8.0, H_a–C(6"')); 3.11 (dd, *J* = 13.5, 4.0, H_b–C(6"')); 3.08–3.07 (m, H–C(2"')); 3.00–2.94 (m, H–C(3)); 2.96 (dd, *J* = 10.0, 3.8, H–C(2')); 2.83 (ddd, *J* = 12.1, 10.0, 4.0, H–C(1)); 2.02 (dt, *J* = 13.0, 4.0, H_{eq}–C(2)); 1.27 (q, *J* = 12.8, H_{ax}–C(2)). ^{13}C-NMR (126 MHz, D_2O): δ 130.17 (2d); 126.53 (2d); 111.25 (d, C(1")); 102.89 (d, ArCH)); 102.10 (d, C(1')); 101.53 (d, C(1"')); 87.00 (d, C(5)); 84.85 (d, C(4)); 83.98 (d, C(4'), C(4")); 79.55 (d, C(6)); 78.26 (d, C(3")); 76.10 (d, C(2")); 75.62 (d, C(5"')); 72.99 (d, C(3'), C(3"')); 71.20 (t, C(6'), d, C(4"')); 66.22 (d, C(5')); 63.61 (t, C(5")); 58.69 (d, C(2')); 55.06 (d, C(2"')); 53.00 (d, C(1)); 52.39 (d, C(3)); 43.53 (t, C(6"')); 37.50 (t, C(2)) HR-MALDI-MS (*m*/*z*): 750.2943 (38); 749.3196 (100, $[M + H]^+$, $C_{30}H_{49}N_6O_{16}^+$; calc. 749.3200).

*4',6'-*O*-(4-Cyanobenzylidene)paromomycin* (**8**). According to Staudinger protocol **B**, reaction of **51** (0.028 mmol, 24 mg) for 24 h, followed by ion-exchange chromatography (*Amberlite* CG-50 (NH_4^+), dioxane/H_2O/25% aq. NH_3 2:1:0 → 2:1:0.06) and lyophilisation gave 16 mg of **8** (78%). White solid. R_f (MeOH/25% aq. NH_3 4:1) 0.29. IR (ATR): 3354*w*, 3392*w*, 2883*w*, 2229*w*, 1581*w*, 1463*w*, 1373*w*, 1119*s*, 1016*s*. ^{1}H-NMR (300 MHz, D_2O): δ 7.83 (d, *J* = 8.0, 2 arom. H); 7.70 (d, *J* = 8.0, 2 arom. H); 5.82 (s, ArCH); 5.51 (br. s, H–C(1')); 5.37 (br. s, H–C(1")); 4.97 (br. s, H–C(1"')); 4.50–2.70 (m, 22 H); 2.00–1.95 (m, H_{eq}–C(2)); 1.22 (q, *J* = 12.6, H_{ax}–C(2)). ^{13}C-NMR (175 MHz, D_2O): δ 140.99, 132.70, 126.95, 119.13, 112.20, 108.47, 100.35, 99.32, 98.75, 84.23, 82.05, 81.19, 80.84, 76.71, 75.44, 73.33, 72.61, 70.18, 70.00, 68.44, 68.04, 63.44, 60.76, 55.90, 52.28, 50.21, 49.63, 40.74, 34.65. HR-MALDI-MS (*m*/*z*): 730.3321 (35); 729.3307 (100, $[M + H]^+$, $C_{31}H_{49}N_6O_{14}^+$; calc. 729.3307).

*4',6'-*O*[4-(Trifluoromethyl)benzylidene]paromomycin* (**9**). According to the general deacetylation

protocol, reaction of 57 mg of **63** and FC gave 42 mg of **83** (94%). White solid. R_f ($CHCl_3$/AcOEt/MeOH 3:3.0.5) 0.44. ^{1}H-NMR (300 MHz, CD_3OD): δ 7.72–7.65 (m, 4 arom. H); 5.85 (d, *J* = 3.9, H–C(1’)); 5.68 (s, ArCH); 5.40 (d, *J* = 1.8, H–C(1”)); 5.14 (d, *J* = 1.7, H–C(1”’)); 4.44 (dd, *J* = 6.7, 4.6, H–C(3”)); 4.33 (dd, *J* = 4.5, 1.8, H–C(2”)); 4.26 (dd, *J* = 9.9, 4.9, H_a–C(6’)); 4.19–4.11 (m, H–C(4”), H–C(5’), H–C(3’)); 4.03 (ddd, *J* = 8.4, 4.5, 1.9, H–C(5”’)); 3.94 (t, *J* = 3.3, H–C(3”’)); 3.85 (dd, *J* = 12.0, 2.7, H_a–C(5”)); 3.81 (t, *J* = 10.0, H_b–C(6’)); 3.72–3.64 (m, H–C(2”’), H_b–C(5”), H–C(4), H–C(5), H_a–C(6”’)); 3.58–3.48 (m, H–C(3)); 3.58 (t, *J* = 9.4, H–C(4’)); 3.47–3.40 (m, H–C(4”’), H–C(1), H–C(6)); 3.39 (dd, *J* = 12.9, 4.5, H_b–C(6”’)); 3.27 (dd, *J* = 10.2, 3.9, H–C(2’)); 2.22 (dt, *J* = 12.8, 4.2, H_{eq}–C(2)); 1.42 (q, *J* = 12.4, H_{ax}–C(2)). HR-MALDI-MS (*m*/*z*): 924.2549 (100, $[M + Na]^+$, $C_{31}H_{38}F_3N_{15}NaO_{14}^+$; calc. 924.2567). Without further characterization, according to Staudinger protocol **A**, reaction of 31 mg of **83** and FC gave 26 mg of **9** (98%). White solid. R_f (MeOH/25% aq. NH_3 4:1) 0.32. IR (ATR): 3354*w*, 2921*w*, 1583*w*, 1444*w*, 1378*w*, 1325*s*, 1122*s*, 1083*s*, 1066*s*, 1017*s*, 927*m*. ^{1}H-NMR (500 MHz, D_2O): δ 7.83 (d, *J* = 8.5, H–C(3) and H–C(5 of $C_6H_4CF_3$); 7.75 (d, *J* = 8.2, H–C(2) and H–C(6) of $C_6H_4CF_3$); 5.87 (s, C_6H_4CH); 5.56 (d, *J* = 3.7, H–C(1’); 5.42 (d, *J* = 2.4, H–C(1’’)); 5.04 (d, *J* = 1.8, H–C(1’’’)); 4.51 (dd, *J* = 6.6, 5.0, H–C(3’’)); 4.375 (dd, *J* = 10.0, 4.8, H_{eq}–C(6’)); 4.36 (dd, *J* = 4.9, 2.3, H–C(2’’)); 4.19 (ddd, *J* = 6.8, 4.9, 3.0, HC(4’’)); 4.12–4.07 (m, H–C(5’), H–C(3’’’), H–C(5’’’)); 3.99–3.88 (m, H–C(3’), H_{ax}–C(6’), H_a–C(5’’)); 3.80–3.74 (m, H–C(5), H–C(4’), H_b–C(5’’)); 3.71 (dd, *J* = 3.9, 1.6, H–C(4’’’)); 3.55 (t, *J* = 9.3, H–C(4))); 3.37 (t, *J* = 9.6, H–C(4)); 3.22 (dd, *J* = 13.5, 8.1, H_a–C(6’’’)); 3.12 (dd, *J* = 13.5, 3.7, H_b–C(6’’’)); 3.11 (dd, *J* = 3.1, 1.8, H–C(2’’’)); 3.02 (td, *J* = 11.0, 3.8, H–C(2’)); 2.87 (td, *J* = 10.0, 4.0, H–C(3)); 2.05 (dt, *J* = 13.0, 4.1, H_{eq}–C(2)); 1.30 (q, *J* = 12.5, H_{ax}–C(2)). ^{13}C-NMR (126 MHz, D_2O): δ 139.89, 126.78, 125.55, 108.45, 100.73, 99.43, 99.08, 84.34, 82.24, 81.27, 80.92, 77.36, 75.66, 74.50, 73.36, 70.45, 70.21, 68.42, 68.09, 63.48, 61.05, 56.00, 52.50, 50.21, 49.71, 40.92, 35.24 (4 Cs are either overlapping or obscured by noise). ^{19}F-NMR (256 MHz, D_2O): δ –60.85 (s, C*F₃*). HR-MALDI-MS (*m*/*z*): 773.3235 (36); 772.3210 (100, $[M + H]^+$, $C_{31}H_{49}F_3N_5O_{14}^+$; calc. 772.3223).

*4’,6’-*O*-(3-Chlorobenzylidene)paromomycin* (**10**). According to the general protocl for deacetylation, reaction of 70 mg of **64** and FC gave 54 mg of **84** (quant.). White solid. R_f ($CHCl_3$/AcOEt/MeOH 3:3.0.5) 0.45. Without characterization, according to Staudinger protocol **A**,

reaction of 52 mg of **84** and FC gave 46 mg of **10**. White solid. R_f (MeOH/25% aq. NH_3 4:1) 0.33. ^{1}H-NMR (500 MHz, D_2O):, δ 7.60–7.59 (m, arom. H); 7.51–7.43 (m, 3 arom. H); 5.75 (s, ArCH); 5.50 (d, *J* = 3.9, H–C(1')); 5.38 (d, *J* = 2.4, H–C(1")); 5.00 (d, *J* = 1.9, H–C(1"')); 4.47 (dd, *J* = 6.5, 4.9, H–C(3")); 4.32 (dd, *J* = 4.9, 2.3, H–C(2")); 4.31 (dd, *J* = 11.1, 4.9, H_a–C(6')); 4.17–4.14 (m, H–C(4")); 4.08–4.01 (m, H–C(5'), H–C(5"')); 4.04 (t, *J* = 3.3, H–C(3"')); 3.91–3.83 (m, H_a–C(5"), H_b–C(6')); 3.84 (t, *J* = 9.7, H–C(3')); 3.76–3.64 (m, H_b–C(5"), H–C(4"'), H–C(5), H–C(4')); 3.50 (t, *J* = 9.3, H–C(4)); 3.33 (t, *J* = 9.7, H–C(6)); 3.20 (dd, *J* = 13.5, 8.0, H_a–C(6"')); 3.11 (dd, *J* = 13.5, 4.0, H_b–C(6"')); 3.08 (ddd, *J* = 3.1, 1.8, 1.1, H–C(2"')); 2.96–2.91 (m, H–C(3), H–C(2')); 2.84–2.79 (m, H–C(1)); 2.01 (dt, *J* = 13.0, 4.1, H_{eq}–C(2)); 1.27 (q, *J* = 12.8, H_{ax}–C(2)). ^{13}C-NMR (126 MHz, D_2O): δ 132.95 (d); 132.47 (d); 128.98 (d); 127.42 (d); 111.20 (d, C(1")); 103.44 (d, ArCH); 102.10 (d, C(1')); 101.51 (d, C(1")); 86.96 (d, C(5)); 84.93 (d, C(4)); 83.58 (d, C(4"), d, C(4')); 79.50 (d, C(6)); 78.26 (d, C(3")); 76.10 (d, C(2")); 75.60 (d, C(5"')); 72.98 (d, C(4"')); 71.20 (t, C(6')); 66.25 (d, C(5')); 63.04 (t, C(5")); 58.68 (d, C(2')); 55.06 (d, C(2"')); 53.00 (d, C(1)); 52.36 (d, C(3)); 43.54. (t, C(6"')); 37.44 (t, C(2)). HR-MALDI-MS (*m/z*): 740.2943 (43); 739.2982 (38); 738.2945 (100, $[M + H]^+$, $C_{30}H_{49}ClN_5O_{14}^+$; calc. 738.2959).

*4',6'-*O*-(3-Methoxybenzylidene)paromomycin Triacetate* (**11**). According to Staudinger protocol **A**, reaction of 26 mg of **52** gave 18 mg of **11** (81%). The product was converted into the tetraacetate salt by stirring in 20% aq. AcOH and taking the soln. to dryness. White solid. R_f (MeOH/25% aq. NH_3 4:1) 0.34. IR (ATR): 3311*w*, 2884*w*, 1542*m*, 1402*m*, 1336*w*, 1285*w*, 1261*w*, 1096*s*, 1042*s*, 1014*s*. ^{1}H-NMR (500 MHz, D_2O):, δ 7.41 (t, *J* = 8.1, 1 arom. H); 7.16–7.07 (m, 2 arom. H); 7.07–7.06 (m, 1 arom. H); 5.85 (d, *J* = 4.1, H–C(1')); 5.75 (s, ArCH); 5.41 (d, *J* = 1.9, H–C(1")); 5.29 (s, H–C(1"')); 4.54 (dd, *J* = 6.7, 4.8, H–C(3")); 4.43 (dd, *J* = 4.6, 1.9, H–C(2")); 4.36–4.31 (m, H–C(5"')); 4.34 (dd, *J* = 10.6, 4.8, H_a–C(6')); 4.24–4.19 (m, H–C(4"), H–C(3"')); 4.16 (t, *J* = 10.0, H–C(3')); 4.01 (td, *J* = 10.0, 4.9, H–C(5')); 3.99–3.85 (m, H–C(5), H_b–C(6'), H_a–C(5")); 3.84 (s, MeO); 3.83–3.70 (m, H–C(4), H–C(4'), H_b–C(5"), H–C(4"')); 3.67 (t, *J* = 10.0, H–C(6)); 3.58 (s, H–C(2"')); 3.49 (dd, *J* = 10.4, 4.2, H–C(2')); 3.43 (dd, *J* = 13.6, 6.9, H_a–C(6"')); 3.37 (dd, *J* = 13.6, 3.8, H_b–C(6"')); 3.32–3.26 (m, H–C(1), H–C(3)); 2.34 (dt, *J* = 12.7, 3.9, H_{eq}–C(2)); 1.90 (s, 3 CH_3CO_2H) 1.70

(q, J = 12.6, H_{ax}–C(2)). . ^{13}C-NMR (126 MHz, D_2O): δ 132.83 (d); 121.65 (d); 118.23 (d); 114.41 (d); 112.72 (d, C(1'')); 104.09 (d, ArCH); 99.10 (d, C(1')); 98.01 (d, C(1''')); 87.53 (d, C(5)); 83.83 (d, C(4'')); 82.59, 81.39 (2d, C(4), C(4')); 77.62 (d, C(3'')); 75.92 (2d, C(6), C(2'')); 72.95 (d, C(5''')); 70.38 (d, C(3'''), t, C(6')); 69.76 (d, C(3'), d, C(4''')); 66.39 (d, C(5')); 62.81 (t, C(5'')); 58.04 (q, MeO, d, C(2')); 53.50 (d, C(2''')); 51.28, 53.50 (2d, C(1), C(3)); 42.96 (t, C(6''')); 32.83 (t, C(2)); 25.77 (d, CH_3CO_2H). HR-MALDI-MS (*m*/*z*): 757.3323 (28); 756.3287 (83, [*M* + Na]$^+$, $C_{31}H_{51}N_5NaO_{15}^+$; calc. 756.3279); 735.3468 (37); 734.3442 (100, [*M* + H]$^+$, $C_{31}H_{52}N_5O_{15}^+$; calc. 734.3454); 574.2595 (31, [*M* – ring IV + 2 H]$^+$, $C_{25}H_{40}N_3O_{12}^+$; calc. 574.2612).

4',6'-O-*(3-Hydroxybenzylidene)paromomycin* (**12**). According to Staudinger protocol **B**, reaction of **85** (140 mg, 0.16 mmol) for 3.5 d followed by FC (MeOH/25% aq. NH3 1:0 → 9:1) and lyophilisation gave **12** (71 mg, 60%). White fluffy solid. R_f (MeOH/25% aq. NH_3 4:1) 0.23. IR (ATR): 3156*w* (br.), 2884*w*, 1601*w*, 1525*w*, 1459*w*, 1376*w*, 1250*w*, 1103*s* (sh), 1043*s,* 762*m*,. ^{1}H-NMR (500 MHz, D_2O): δ 7.50 (*dd*, J = 7.8, 1.8, 1 arom. H); 7.35 (*ddd*, J = 8.2, 7.4, 1.7, 1 arom. H); 6.99 (*td*, J = 7.6, 1.1, 1 arom. H); 6.95 (*dd*, J = 8.2, 1.1, 1 arom. H); 5.97 (*s*, ArC*H*); 5.63 (d, J = 3.9, H–C(1')); 5.40 (d, J = 2.2, H–C(1'')); 5.15 (d, J = 1.8, H–C(1''')); 4.52 (dd, J = 7.0, 5.0, H–C(3'')); 4.39 (dd, J = 5.0, 2.2, H–C(2'')); 4.30 (dd, J = 10.3, 4.9, H_a–C(6')); 4.25–4.23 (m, H–C(4'')); 4.20–4.17 (m, H–C(5''')); 4.15 (t, J = 3.2, H–C(3''')); 4.10–4.05 (m, H–C(5')); 3.98 (dd, J = 10.3, 9.9, H_b–C(6')); 3.92–3.88 (m, H_a–C(5''), H–C(3')); 3.81–3.73 (m, H–C(4'), H–C(5), H_b–C(5''), H–C(4''')); 3.60 (t, J = 9.4, H–C(4)); 3.55 (t, J = 9.8, H–C(6)); 3.40–3.30 (m, H–C(2'''), H_a–C(6'''), H_b–C(6''')); 3.18-3.04 (m, H–C(2'), H–C(1), H–C(3)); 2.18 (dt, J = 12.9, 4.0, H_{eq}–C(2)); 1.29 (dt, J = 12.9, 12.6, H_{ax}–C(2)). ^{13}C-NMR (125 MHz, D_2O): δ 153.68 (s); 131.08, 127.02 (2 d); 122.25 (s); 120.06, 116.13 (2 d); 109.00 (C(1'')); 98.29 (d, Ar*C*H); 97.91 (C(1')); 96.75 (C(1''')); 84.19 (d, C(5)); 80.91 (C(4), C(4'')); 80.44 (C(4')); 74.85 (C(3'')); 74.16 (C(6)); 73.04 (C(2'')); 75.77 (C(5''')); 68.80 (C(3''')); 68.59 (C(3')) ; 67.79 (C(6')) ; 67.67 (C(4''')); 63.43 (C(5')); 60.21 (C(5'')); 55.21 (C(3)); 51.35 (C(2''')); 50.25 (C(2')); 48.93 (C(1)); 40.33 (t, C(6''')); 32.17 (C(2)). HR-MALDI-MS (*m*/*z*): 720.3300 (100, [*M* + H]$^+$, $C_{30}H_{50}N_5O_{15}^+$; calc. 720.3303).

4',6'-O-*(3-nitrobenzylidene)paromomycin-triammonium Triacetate* (**13-3AcOH**). Reaction of

100 mg of **45** at 60° for 23 h according to the general procedure for tranacetalization and FC gave 29 mg of **53** (26%). White solid. R_f ($CHCl_3$/AcOEt/MeOH 3:3:0.5) 0.36. ^{1}H-NMR (300 MHz, CD_3OD): δ 8.38 (s, 1 arom. H); 8.24 (d, J = 8.3, 1 arom. H); 7.92 (d, J = 7.7, 1 arom. H); 7.63 (t, J = 8.0, 1 arom. H); 5.84 (d, J = 3.7, H–C(1')); 5.74 (s, ArCH); 5.39 (s, H–C(1")); 5.13 (s, H–C(1"')); 4.43 (dd, J = 6.6, 4.8, H–C(3")); 4.34–4.32 (m, H–C(2")); 4.27 (dd, J = 9.9, 4.9, H_a–C(6')); 4.19–4.10 (m, 3 H); 4.05–3.99 (m, H–C(5"')); 3.94 (t, J = 3.1, H–C(3"')); 3.86 (dd, J = 11.9, 3.0, H_a–C(5")); 3.82 (t, J = 9.6, H_b–C(6')); 3.71–3.46 (m, 7 H); 3.48–3.25 (m, 5 H); 2.26–2.16 (m, H_{eq}–C(2)); 1.42 (q, J = 12.4, H_{ax}–C(2)). HR-MALDI-MS (m/z): 878.2604 (44); 877.2561 (100, $[M-H]^-$, $C_{30}H_{37}N_{16}O_{16}^-$; calc. 877.2573). Without further characterization, according to Staudinger protocol **A**, reaction of 16 mg of **53** and FC followed by stirring the crude in 10% aq. AcOH and evaporation gave 11 mg of **13-3AcOH** (61%). Yellowish solid. R_f (MeOH/25% aq. NH_3 4:1) 0.31. IR (ATR): 3095*m*, 1528*m*, 1406*m*, 1349*m*, 1100*s*, 1045*s*, 1014*s*, 937*m*. ^{1}H-NMR (500 MHz, D_2O): δ 8.43–8.42 (m, 1 arom. H); 8.34–8.32 (m, 1 arom. H); 7.96–7.94 (m, 1 arom. H); 7.72–7.69 (m, 1 arom. H); 5.91 (s, ArCH); 5.84 (d, J = 4.1, H–C(1')); 5.43 (d, J = 2.2, H–C(1")); 5.30 (d, J = 1.6, H–C(1"')); 4.56 (dd, J = 6.8, 4.8, H–C(3")); 4.45 (dd, J = 4.7, 2.2, H–C(2")); 4.40 (dd, J = 10.3, 4.8, H_a–C(6')); 4.35–4.33 (m, H–C(5"')); 4.25 (t, J = 3.2, H–C(3"')); 4.26–4.22 (m, H–C(4"));; 4.17 (t, J = 9.8, H–C(3')); 4.07 (td, J = 10.0, 4.8, H–C(5')); 3.97 (t, J = 10.4, H_b–C(6')); 3.95 (dd, J = 12.5, 3.1, H_a–C(5")); 3.90 (t, J = 9.1, H–C(5)); 3.85 (t, J = 9.5, H–C(4')); 3.84–3.83 (m, H–C(4"')); 3.80–3.76 (m, H–C(4), H_b–C(5")); 3.68 (t, J = 10.3, H–C(6)); 3.58–3.55 (m, H–C(2"')); 3.50–3.47 (m, H–C(2')); 3.44 (dd, J = 13.6, 7.0, H_a–C(6"')); 3.38 (dd, J = 13.6, 3.9, H_b–C(6"')); 3.32–3.23 (m, H–C(1), H–C(3)); 2.33 (dt, J = 12.7, 4.0, H_{eq}–C(2)); 1.92 (s, 3 CH_3CO_2H); 1.67 (q, J = 12.6, H_{ax}–C(2)). ^{13}C-NMR (126 MHz, D_2O): δ 135.66 (d); 132.72 (d); 127.34 (d); 124.06 (d); 112.67 (d, C(1")); 102.75 (s, ArCH); 99.41 (d, C(1')); 98.18 (d, C(1"')); 87.58 (d, C(5)); 83.88 (d, C(4")); 82.70 (2d, C(4), C(4')); 77.67 (d, C(3")); 75.96 (2d, C(6), C(2")); 73.06 (d, C(5"')); 70.00 (3d, C(3'), C(3"'), C(4"'), t, C(6')); 66.36 (d, C(5')); 62.92 (t, C(5")); 57.27 (d, C(2')); 53.64 (d, C(2"')); 53.64, 51.45 (2d, C(1), C(3)); 43.08 (t, C(6"')); signal of C(2) hidden. HR-MALDI-MS (m/z): 750.3211 (36); 749.3187 (100, $[M+H]^+$, $C_{30}H_{49}N_6O_{16}^+$; calc. 749.3200).

*4',6'-*O*-(2-Methoxybenzylidene)paromomycin* (**14**). According to Staudinger protocol **A**, reaction of 18 mg of **54** and FC gave 21 mg of **14**. White solid. R_f (MeOH/25% aq. NH_3 4:1) 0.31.

$[\alpha]_D^{25}$ = +51.3 (*c* = 0.15, H_2O). IR (ATR): 3148*w*, 1606*w*, 1526*w*, 1498*w*, 1463*w*, 1386*w*, 1287*w*, 1250*w*, 1095*s*, 1047*s*. ^{1}H-NMR (500 MHz, D_2O): δ 7.59–7.58 (m, 1 arom. H); 7.51–7.47 (m, 1 arom. H); 7.14–7.08 (m, 2 arom. H); 6.05 (s, ArCH); 5.94 (d, *J* = 4.2, H–C(1')); 5.44 (d, *J* = 2.1, H–C(1")); 5.32 (d, *J* = 1.6, H–C(1"')); 4.57 (dd, *J* = 6.8, 4.8, H–C(3")); 4.52 (dd, *J* = 4.6, 2.1, H–C(2")); 4.36–4.33 (m, H–C(5"')); 4.34 (dd, *J* = 10.3, 5.0, H_a–C(6')); 4.28–4.21 (m, H–C(3"'), H–C(4")); 4.23 (t, *J* = 9.8, H–C(3')); 4.02 (td, *J* = 10.0, 4.8, H–C(5')); 3.97 (m, H–C(4'), H_a–C(5"), H_b–C(6'), H–C(5)); 3.88 (s, MeO); 3.85–3.84 (m, H–C(4"')); 3.81 (t, *J* = 9.5, H–C(4)); 3.78–3.73 (m, H_b–C(5"), H–C(6)); 3.61 (br. s, H–C(2"')); 3.55 (dd, *J* = 10.5, 4.2, H–C(2')); 3.48–3.43 (m, H–C(3)); 3.45 (dd, *J* = 13.6, 7.0, H_a–C(6"')); 3.38 (dd, *J* = 13.5, 3.9, H_b–C(6"')); 3.40–3.34 (m, H–C(1)); 2.45 (dt, *J* = 12.7, 4.1, H_{eq}–C(2)); 1.82 (q, *J* = 12.6, H_{ax}–C(2)). ^{13}C-NMR (126 MHz, D_2O): δ 134.27 (d); 129.50 (d); 123.81 (d); 114.82 (d); 112.91 (d, C(1")); 100.30 (d, ArCH); 98.89 (d, C(1')); 97.90 (d, C(1"')); 87.52 (d, C(5)); 83.91 (d, C(4")); 82.73 (d, C(4)); 80.15 (d, C(4')); 77.57 (d, C(3")); 75.91 (d, C(6), C(2")); 73.01 (d, C(5"')); 70.44, 69.87 (3d, C(3'), C(3"'), C(4"'), t, C(6')); 66.61 (d, C(5')); 62.79 (t, C(5")); 58.56 (q, MeO); 57.61 (d, C(2')); 53.53 (d, C(2"')); 52.88 (d, C(1)); 51.30 (d, C(3)); 43.02 (t, C(6"')); 31.94 (t, C(2)). HR-MALDI-MS (*m/z*): 757.3328 (25); 756.3291 (67, $[M + Na]^+$, $C_{31}H_{51}N_5NaO_{15}^+$; calc. 756.3279); 735.3470 (36); 734.3442 (100, $[M + H]^+$, $C_{31}H_{52}N_5O_{15}^+$; calc. 734.3454); 442.1517 (32, [*M* – ring III – ring IV + 2 H]$^+$, $C_{20}H_{32}N_3O_8^+$; calc. 442.2189).

*4',6'-*O*-(2-Nitrobenzylidene)paromomycin* (**15**). According to the general acetalization protocol **B**, reaction of 91 mg of **60** for 28 h and FC gave 30 mg of **66** (29%). Yellowish solid. R_f (cyclohexane/AcOEt 1:1) 0.62. Without further characterization, deacetylation of 54 mg of **66** and FC gave 26 mg of **86** (62%). White solid. R_f ($CHCl_3$/AcOEt/MeOH 3:3.0.5) 0.51. Finally, and without further characterization, according to Staudinger protocol **A**, reaction of 20 mg of **86** and FC gave 17 mg of **15** (quant.). White solid. R_f (MeOH/25% aq. NH_3 4:1) 0.22. IR (ATR): 3179*w*, 2895*w*, 1610*w*, 1528*m*, 1452*w*, 1349*s*, 1099*s*, 1048*s*, 1028*s*. ^{1}H-NMR (500 MHz, D_2O): δ 8.02–8.00 (m, arom. H); 7.92–7.90 (m, arom. H); 7.81–7.78 (m, arom. H); 7.70–7.66 (m, arom. H); 6.26 (s, ArCH); 5.63 (d, *J* = 3.9, H–C(1')); 5.40 (d, *J* = 2.3, H–C(1")); 5.15 (d, *J* = 1.8, H–C(1"')); 4.52 (dd, *J* = 4.9, 6.7, H–C(3")); 4.38 (dd, *J* = 2.3, 4.8, H–C(2")); 4.33 (dd, *J* = 4.9, 10.2, H_a–C(6')); 4.24 (ddd, *J* = 1.5, 3.8, 7.3, H–C(5"')); 4.20–4.17 (m, H–C(4")); 4.14 (t, *J* = 3.2, H–C(3"')); 4.04 (td, *J* = 10.1, 4.9, H–C(5')); 3.96–

3.90 (m, H–C(3'), H_b–C(6')); 3.90 (dd, J = 2.8, 9.2, H_a–C(5")); 3.80 (t, J = 9.2, H–C(5)); 3.80–3.74 (m, H_b–C(5"), H–C(4"'), H–C(4')); 3.60 (t, J = 9.4, H–C(4)); 3.54 (t, J = 10.2, H–C(6)); 3.38 (dd, J = 7.4, 13.6, H_a–C(6"')); 3.32 (dd, J = 3.9, 13.6, H_b–C(6"')); 3.31–3.30 (m, H–C(2"')); 3.14 (dd, J = 3.9, 10.2, H–C(2')); 3.12–3.04 (m, H–C(3), H–C(1)); 2.17 (dt, J = 12.9, 4.1, H_{eq}–C(2)); 1.47 (q, J = 12.6, H_{ax}–C(2)). ^{13}C-NMR (126 MHz, D_2O): δ 136.48 (d); 133.51 (d); 130.28 (d); 127.32 (d); 111.79 (d, C(1")); 100.95 (d, C(1')); 100.24 (d, ArCH); 99.88 (d, C(1")); 87.02 (d, C(5)); 83.87 (d, C(4'), d, C(4"), d, C(4)); 77.87 (d, C(3")); 77.20 (d, C(6)); 76.00 (d, C(2")); 73.52 (d, C(5"')); 71.81 (d, C(3'), d, C(3"')); 70.69 (t, C(6'), d, C(4"')); 66.26 (d, C(5')); 63.16 (t, C(5")); 58.13 (d, C(2')); 54.33 (d, C(2"')); 53.16 (d, C(3)); 51.90 (d, C(1)); 43.27 (t, C(6"')); 35.23 (t, C(2)). HR-MALDI-MS (*m*/*z*): 771.2961 (31, $[M + Na]^+$, $C_{30}H_{48}N_6NaO_{16}^+$; calc. 771.3024); 750.3213 (46); 749.3186 (100, $[M + H]^+$, $C_{30}H_{49}N_6O_{16}^+$; calc. 749.3205).

*4',6'-*O*-(3,5-Dichlorobenzylidene)paromomycin* (**16**). Reaction of 80 mg of **45** at 60° for 22 h according to the general procedure for transacetalization and FC gave 22 mg of **55** (23%). White solid. R_f ($CHCl_3$/AcOEt/MeOH 3:3:0.5) 0.39. ^{1}H-NMR (300 MHz, CD_3OD): δ 7.47–7.42 (m, 3 arom. H); 5.83 (d, J = 3.8, H–C(1')); 5.59 (s, ArCH); 5.38 (d, J = 1.7, H–C(1")); 5.13 (d, J = 1.7, H–C(1"')); 4.43 (dd, J = 6.6, 4.7, H–C(3")); 4.32 (dd, J = 4.4, 1.7, H–C(2")); 4.24 (dd, J = 9.9, 4.7, H_a–C(6')); 4.17–4.08 (m, H–C(4"), H–C(5'), H–C(3')); 4.02 (ddd, J = 8.5, 4.4, 1.9, H–C(5"')); 3.94 (t, J = 3.3, H–C(3"')); 3.84 (dd, J = 12.1, 2.8, H_a–C(5")); 3.77 (t, J = 10.2, H_b–C(6')); 3.73–3.63 (m, H–C(2"'), H_b–C(5"), H–C(4), H–C(5), H_a–C(6"')); 3.58–3.49 (m, H–C(3)); 3.55 (t, J = 9.3, H–C(4')); 3.48–3.35 (m, H–C(4"'), H–C(1), H–C(6), H_b–C(6"')); 3.26 (dd, J = 10.4, 3.8, H–C(2')); 2.22 (dt, J = 12.6, 4.1, H_{eq}–C(2)); 1.41 (q, J = 12.4, H_{ax}–C(2)). ^{13}C-NMR (75 MHz, CD_3OD): δ 142.57 (s); 135.76 (2s); 129.60 (d); 126.21 (2d); 109.72 (d, C(1")); 100.76 (d, ArCH); 99.70 (d, C(1"')); 99.03 (d, C(1')); 85.21 (d, C(5)); 83.40 (d, C(4")); 82.87 (d, C(4')); 77.78 (d, C(4)); 77.24 (2d, C(3"), C(6)); 75.64 (d, C(5"')); 75.05 (d, C(2")); 71.16 (d, C(3"')); 69.80 (t, C(6')); 69.60 (2d, C(4"'), C(3')); 65.14 (d, C(2')); 64.37 (d, C(5')); 63.70 (t, C(5")); 61.89 (2d, C(1), C(2"')); 61.23 (d, C(3)); 52.51 (t, C(6"')); 33.06 (t, C(2)). Without further characterization, according to Staudinger protocol **A**, reaction of 25 mg of **55** and FC gave 19 mg of **16** (89%). White solid. R_f (MeOH/25% aq. NH_3 4:1) 0.29. IR (ATR): 3188*w*, 2900*w*, 1597*w*, 1573*w* 1526*w*, 1429*w*, 1369*w*, 1101*s*, 1029*s*. ^{1}H-NMR (500 MHz, D_2O): δ

7.57–7.56 (m, 1 arom. H); 7.53–7.52 (m, 2 arom. H); 5.73 (s, ArCH); 5.56 (d, *J* = 3.9, H–C(1’)); 5.39 (d, *J* = 2.4, H–C(1”)); 5.09 (d, *J* = 1.8, H–C(1”’)); 4.50 (dd, *J* = 6.6, 5.0, H–C(3”)); 4.35 (dd, *J* = 4.9, 2.4, H–C(2”)); 4.32 (dd, *J* = 10.3, 4.9, H_a–C(6’)); 4.21–4.16 (m, H–C(5”’), H–C(4”)); 4.10 (t, *J* = 3.2, H–C(3”’)); 4.02 (td, *J* = 10.1, 4.9, H–C(5’)); 3.94–3.87 (m, H–C(3’), H_b–C(6’), H_a–C(5”)); 3.79–3.69 (m, H_b–C(5”), H–C(4’), H–C(5), H–C(4”’)); 3.57 (t, *J* = 9.4, H–C(4)); 3.48 (t, *J* = 10.1, H–C(6)); 3.35 (dd, *J* = 13.5, 7.5, H_a–C(6”’)); 3.28 (dd, *J* = 13.6, 4.0, H_b–C(6”’)); 3.22–3.21 (m, H–C(2”’)); 3.05 (dd, *J* = 10.1, 3.9, H–C(2’)); 3.05–3.00 (m, H–C(1), H–C(3)); 2.12 (dt, *J* = 12.9, 4.1, H_{eq}–C(2)); 1.41 (q, *J* = 12.5, H_{ax}–C(2)). ^{13}C-NMR (126 MHz, D_2O): δ 132.17 (d); 127.66 (2d); 111.53 (d, C(1”)); 102.52 (d, ArCH); 101.54 (d, C(1’)); 100.57 (d, C(1”’)); 86.90 (d, C(5)); 83.92 (d, C(4), d, C(4”’)); 83.34 (d, C(4’)); 77.97 (d, C(6)); 76.04 (d, C(2”)); 73.90 (d, C(5”’)); 72.29 (d, C(3”’), d, C(3’)); 70.95 (t, C(6’), d, C(4”’)); 66.25 (d, C(5’)); 63.31 (t, C(5”)); 58.39 (d, C(2’)); 54.62 (d, C(2”’)); 53.11 (d, C(1)); 52.08 (d, C(3)); 43.35 (t, C(6”’)); 35.92 (t, C(2)). HR-MALDI-MS (*m*/z): 772.2556 (100, $[M + H]^+$, $C_{30}H_{49}Cl_2N_5O_{14}^+$; calc. 772.2575).

4’,6’-O-*(3,5-Dimethoxybenzylidene)paromomycin* (**17**). According to the general acetalization protocol **B**, reaction of 70 mg of **60** for 22 h and FC gave 58 mg of **67** (72%). White solid. R_f (hexane/AcOEt 1:1) 0.34. HR-MALDI-MS (*m/z*): 1184.3294 (43, $[M + K]^+$, $C_{44}H_{55}KN_{15}O_{22}^+$; calc. 1184.3283); 1169.3562 (54); 1168.3568 (100, $[M + Na]^+$, $C_{44}H_{55}N_{15}NaO_{22}^+$; calc. 1168.3544). Without further characterization, deacetylation of 19 mg of **67** and FC gave 26 mg of **87** (98%). White solid. R_f ($CHCl_3$/AcOEt/MeOH 3:3.0.5) 0.21. Fin ally, according to Staudinger protocol **A**, reaction of 24 mg of **87** and FC gave 19 mg of **17** (92%). White solid. R_f (MeOH/25% aq. NH_3 4:1) 0.34. IR (ATR): 3179*w*, 2896*w*, 1597*m*, 1463*w*, 1431*w*, 1384*w*, 1338*w*, 1304*w*, 1200*w*, 1150*s*, 1087*s*, 1047*s*. ^{1}H-NMR (500 MHz, D_2O): δ 6.79–6.78 (m, 2 arom. H) 6.66–6.64 (m, 1 arom. H); 5.70 (s, ArCH); 5.56 (d, *J* = 3.9, H–C(1’)); 5.39 (d, *J* = 2.4, H–C(1”)); 5.09 (d, *J* = 1.8, H–C(1”’)); 4.50 (dd, *J* = 6.7, 4.9, H–C(3”)); 4.36 (dd, *J* = 4.9, 2.4, H–C(2”)); 4.31 (dd, *J* = 10.3, 4.9, H_a–C(6’)); 4.21–4.15 (m, H–C(4”), H–C(5”’)); 4.10 (t, *J* = 3.2, H–C(3”’)); 4.04 (td, *J* = 10.1, 4.8, H–C(5’)); 3.93–3.85 (m, H_b–C(6’), H_a–C(5”)); 3.91 (t, *J* = 9.6, H–C(3’)); 3.84 (s, 2 MeO); 3.77–3.73 (m, H–C(4”’), H_b–C(5”)); 3.77 (t, *J* = 9.2, H–C(5)); 3.71 (t, *J* = 9.5, H–C(4’)); 3.57 (t, *J* = 9.4, H–C(4)); 3.48 (t, *J* = 9.4, H–C(6)); 3.34 (dd, *J* = 13.6, 7.6, H_a–C(6”’)); 3.28 (dd, *J* = 13.6, 3.9, H_b–C(6”’)); 3.22–3.20 (m, H–C(2”’));

3.07–2.99 (m, H–C(1), H–C(3)); 3.06 (dd, J =10.1, 3.9, H–C(2')); 2.13 (dt, J = 12.9, 4.2, H_{eq}–C(2)); 1.41 (q, J = 12.5, H_{ax}–C(2)). ^{13}C-NMR (126 MHz, D_2O, data from a HSQC spectrum): δ 111.60 (d, C(1")); 107.40 (d, 1 C); 104.40 (d, 2 C, ArCH); 101.50 (d, C(1')); 100.60 (d, C(1"')); 86.96 (d, C(5)); 83.91 (d, C(4), C(4")); 83.42 (d, C(4')); 77.98 (d, C(6), C(3")), 76.06 (d, C(2")); 73.90 (d, C(5"')); 72.29 (d, C(3'), C(3"')); 70.94 (d, C(4"'), t, C(6')), 66.31 (d, C(5')); 63.29 (t, C(5")); 58.29 (d, C(2'), q, 2 Me); 54.62 (d, H–C(2"')); 53.11 (d, C(3)); 51.11 (d, C(1)); 43.30 (t, C(6"')); 35.90 (t, C(2)). HR-MALDI-MS (m/z): 765.3554 (36); 764.3546 (100, $[M + H]^+$, $C_{32}H_{54}N_5O_{16}^+$; calc. 764.3560).

*4',6'-*O*-(2,5-Methoxybenzylidene)paromomycin* (**18**). According to Staudinger protocol **A**, reaction of 25 mg of **56** and FC gave 21 mg of **18** (98%). White solid. R_f (MeOH/25 % aq. NH_3 4:1) 0.29. IR (ATR): 3287*w*, 2913*w*, 1593*w*, 1503*m*, 1464*m*, 1425*w*, 1386*w*, 1280*w*, 1221*w*, 1104*s*, 1019*s*. ^{1}H-NMR (500 MHz, D_2O): δ 7.19–7.18 (m, arom. H); 7.11–7.06 (m, 2 arom. H); 5.99 (s, ArCH); 5.54 (d, J = 3.9, H–C(1')); 5.39 (d, J = 2.3, H–C(1")); 5.05 (d, J = 1.8, H–C(1"')); 4.50 (dd, J = 6.7, 4.9, H–C(3")); 4.35 (dd, J = 4.9, 2.3, H–C(2")); 4.28 (dd, J = 10.3, 5.0, H_a–C(6')); 4.18–4.13 (m, H–C(5"'), H–C(4")); 4.07 (t, J = 3.3, H–C(3"')); 4.05 (td, J = 10.0, 4.9, H–C(5')); 3.92–3.86 (m, H–C(3'), H_b–C(6'), H_a–C(5")); 3.85, 3.82 (2s, 2 MeO); 3.78–3.68 (m, H_b–C(5"), H–C(5), H–C(4"'), H–C(4')); 3.55 (t, J = 9.4, H–C(4)); 3.43 (t, J = 9.9, H–C(6)); 3.30 (dd, J = 13.5, 7.8, H_a–C(6"')); 3.23 (dd, J = 13.5, 3.9, H_b–C(6"')); 3.15–3.14 (m, H–C(2"')); 3.04–2.99 (m, H–C(3)); 3.00 (dd, J = 10.4, 3.9, H–C(2')); 2.95 (ddd, J = 12.1, 10.1, 4.1, H–C(1)); 2.09 (dt, J = 13.0, 4.1, H_{eq}–C(2)); 1.36 (q, J = 12.4, H_{ax}–C(2)). ^{13}C-NMR (126 MHz, D_2O): δ 119.21 (d); 116.72 (d); 115.15 (d); 111.37 (d, C(1")); 101.67 (d, C(1')); 100.95 (d, C(1"')); 99.89 (d, ArCH); 86.93 (d, C(5)); 84.36 (d, C(4)); 83.87 (d, C(4"), d, C(4')); 78.52 (d, C(6)); 78.01 (d, C(3")); 76.05 (d, C(2")); 74.35 (d, C(5"')); 72.59 (d, C(3'), d, C(3"')); 71.07 (t, C(6'), d, C(4"')); 66.32 (d, C(5')); 63.36 (t, C(5")); 59.47, 58.64 (2q, 2MeO); 54.80 (d, C(2"')); 53.07 (d, C(1)); 52.14 (d, C(3)); 43.41 (t, C(6"')); 36.51 (t, C(2)). HR-MALDI-MS (m/z): 786.3382 (26, $[M + Na]^+$, $C_{32}H_{53}N_5NaO_{16}^+$; calc. 786.3385); 765.3571 (37); 764.3545 (100, $[M + H]^+$, $C_{32}H_{54}N_5O_{16}^+$; calc. 764.3560).

*4',6'-*O*-(3-Bromo-4-hydroxybenzylidene)paromomycin* (**19**). According to Staudinger protocol **B**, reaction of **88** (60 mg, 0.065 mmol) for 3.5 d, followed by FC (MeOH/25% aq. NH_3 1:0 to 9:1) and

lyophilisation gave **19** (37 mg, 60%). Light brown solid. *R*f (MeOH/25% aq. NH3 4:1) 0.20. IR (ATR): 3152*w* (br.), 2896*w*, 1595*w*, 1515*w*, 1368*w*, 1293*w*, 1003*s* (br.), 835*w,* 687*w*. ^{1}H-NMR (400 MHz, D_2O): δ 7.63 (*br. s*, 1 arom. H); 7.27 (br. *d*, *J* = 8.5, 1 arom. H); 6.86 (*br. d*, *J* = 8.4, 1 arom. H); 5.56 (br. *s*, ArC*H*). 5.56 (H–C(1')); 5.34 (H–C(1'')); 5.17 (H–C(1''')); 4.49 (dd, *J* = 5.6, 5.6, H–C(3'')); 4.37…4.35 (H–C(2'')); 4.28–4.19 (m. H–C(4''), H_aC(6')); 4.16–4.13 (m, H–C(3'''), H–C(5''')); 3.98–3.48 (m, H–C(3'), H–C(4'), H–C(5'), H_b–C(6'), H–C(4), H–C(5), H–C(6), H_a–C(5'') H_b–C(5''), H–C(4''')); 3.42–3.26(m, H–C(2'''), H_a–C(6'''), H_bC(6''')); 3.22–3.04 (m, H–C(3), H–C(2')); 2.95–2.89 (d, *J* = 12.7, H–C(1)), 2,16–2.10 (m, H_{eq}–C2)); 1.45 (m, H_{ax}–C(2)). ^{13}C-NMR (100 MHz, D_2O): δ 158.88 (*s*); 131.87, 128.04 (2 *d*); 118.54 (*d*); 112.10 (*s*); 102.11 (*d*, ArCH). 110.38 (C(1'')); 98.78 (C(1')); 97.38 (C(1''')); 85.37 (C(5)); 82.13 (C(4'')); 82.09 (C(4)); 81.28 (C(4')); 76.13 (C(3'')); 74.79 (C(6)); 74.24 (C(2'')); 71.54 (C(5''')); 69.51 (C(3''')); 69.73 (C(6')); 69.24 (C(3')); 68.65 (C(4''')); 64.64 (C(5')); 61.39 (C(5'')); 56.18 (C(3)); 53.02 (C(2''')); 52.31 (C(2')); 51.42 (C(1)); 41.45 (C(6''')); 32.89 (C(2)). HR-MALDI-MS (*m*/*z*): 780.2388 (100, [*M* + H]$^{+}$, $C_{30}H_{49}{}^{81}BrN_5O_{15}{}^{+}$; calc. 780.2409), 798.2403 (96, [*M* + H]$^{+}$, $C_{30}H_{49}{}^{81}BrN_5O_{15}{}^{+}$; calc. 798.2409.

*4',6'-*O*-(3-Formyl-4-hydroxybenzylidene)paromomycin* (**20**). According to Staudinger protocol **B**, reaction of **104** (20 mg, 22.8 µmol) for 3.5 d and lyophilisation gave **20** (12 mg, 71%). Yellow fluffy solid.^{1}H-NMR (600 MHz, D_2O): δ 8.52 (*s*, C*H*O); 7.34 (*d*, *J* = 1.8, 1 arom. H); 7.21 (*dd*, *J* = 9.0, 1.8, 1 arom. H); 6.91 (*d*, *J* = 9.0, 1 arom. H); 5.76 (d, *J* = 3.6, H–C(1')); 5.45 (*s*, ArC*H*). 5.42 (d, *J* = 2.2, H–C(1'')); 5.06 (d, *J* = 1.2, H–C(1''')); 4.54 (dd, 7.8, 4.2, H–C(3'')); 4.45 (dd, *J* = 4.8, 2.2, H–C(2'')); 4.21–4.15, m, H–C(4''), H–C(5''')); 4.09 (dd, *J* = 3.2, 3.2, H–C(3''')); 4.07–4.00 (m, H–C(5'), H–C(6')); 3.96–3.84 (m, H_b–C(6'), H–C(5''), H_a–C(5'')); 3.79-3.56 (m, H–C(3'), H–C(4'), H–C(5), Hb–C(5''), H–C(4''')); 3.51 (dd, *J* = 9.6, 9.6, H–C(4)); 3.42 (dd, *J* = 10.0, 10.0, H–C(6)); 3.37–3.19 (m, H–C(3), H_a–C(6'''), H_b–C(6''')); 3.13 (dd, *J* = 3.2, 3.2, H–C(3''')); 2.18 (m, J_{gem} = 12.6, H_{eq}–C(2),); 1.80 (dd, *J* = 12.6, 12.6, H_{ax}–C(2)). ^{13}C-NMR (150 MHz, D_2O): δ 181.49 (*s*, CHO); 167.28 (*s*); 132.62, 129.78 (2 *d*); 126.20 (*s*); 118.30 (*d*); 116.13 (*s*); 109.63 (C(1''); 99.18 (*d*, ArCH); 98.45 (C(1'); 98.24 (C(2''')); 86.13 (C(5)); 80.93 (C(4'')); 80.15 (C(4')); 76.97 (C(4)); 76.37 (C(6)); 75.29 (C(2'')); 73.61 (C(3''));72.20 (C(5''')); 70.17 (C(3''')); 69.27 (C(3')); 68.51 (C(4''')); 68.50 (C(6')); 67.48 (C(2')); 65.77 (C(5')); 63.35 (C(5'')); 55.67 (C(3)); 52.33 (C(2''')); 50.08 (C(1)); 40.77

(C(6’’’)); 34.77 (C(2)). HR-MALDI-MS (*m/z*): 748.3247 (100, $[M + H]^+$, $C_{31}H_{50}N_5O_{16}^+$; calc. 748.3253).

4’,6’-O-*(3-Carboxylato-4-hydroxybenzylidene)paromomycin* ˉ 0.5 NEt_3 (**21**). According to Staudinger protocol **B**, reaction of **105** (20 mg, 22.38 µmol) for 3.5 d, evaporation, FC (MeOH/25% aq. NH3 1:0 → 9:1), and lyophilisation gave **21** (11.5 mg, 68%). White powder. *R*f (MeOH/25% aq. NH_3 85:15) 0.32. IR (ATR): 3061*w* (br.), 2884*w*, 1672*w* (sh.), 1623*w*, 1598*w*, 1524*w*, 1446*w*, 1383*w*, 1048*s,* 836*w*, 709*w*. ^{1}H-NMR (600 MHz, D_2O): δ 7.97 (*d*, *J* = 2.1, 1 arom. H); 7.57 (*dd*, *J* = 8.5, 2.1, 1 arom. H); 6.99 (*d*, *J* = 8.5, 1 arom. H); 6.04 (d, *J* = 4.2 H–C(1’)); 5.75 (*s*, ArC*H*); 5.48 (d, *J* = 1.8, H–C(1’’)); 5.35 (d, *J* = 1.2, H–C(1’’’)); 4.73–43.43 (m, H_a–C(6’), H–C(4’’)); 4.59 (dd, *J* = 6.6, 4.8, H–C(3’’)); 4.50 (dd, ¡ = 1.8, 4.8)); 4.30–4.26 (m, H–C(5’), H–C(3’’’), H–C(5’’’)); 4.19 (H_b–C(6’)); 4.05–3.89 (m, H–C(3’), H–C(5), H_a–C(5’’), H_b–C(5’’)); 3.87–3.78 (m, H–C(4’), H–C(4), H–C(4’’’)); 3.68–3.39 (m, H–C(2’), H–C(1), H–C(3), H–C(6), H–C(2’’’), H_a–C(6’’’), H_b–C(6’’’)); 3.22 (q, *J* = 7.2, 1 H, N$CH_2$$CH_3$); 2.57 (ddd, *J* = 12.6, 4.2, 4.2) H_{eq}–C(2)); 1.98 (ddd, *J* = 12.6, 12.6, 12.6, H_{ax}–C(2)); 1.30 (t, *J* = 7.2, N$CH_2$$CH_3$). ^{13}C-NMR (150 MHz, D_2O): δ 174.84 (*s*, C=O); 160.89 (*s*); 131.74, 128.54 (2 *d*); 127.05 (s); 116.69 (*d*); 110.32 (C(1’’)); 101.48 (*d*, Ar*C*H); 96.02 (C(1’)); 95.14 (C(1’’’)); 84.69 (C(5)); 81.29 (C(4’’)); 79.75 (C4’)); 75.68 (C(4)); 74.84 C(3’’)); 73.24 (C(6)); 72.45 (C(2’’)); 70.36 (C5’’’)); 67.68 (C(3’’’)); 67.61 (C(3’)); 67.17 (C6’)); 66.19 (C(4’’’)); 64.03 (C(5’)); 60.20 C(5’’)); 54.32 (C(3)); 50.86 (C(2’’’)); 49.87 (C(2’)); 48.48 (C(1)); 40.38 (C(6’’’)); 28.06 (C(2)); –(46.67 (*t*, N*C*H_2Me); 8.2 (*q*, NCH_2*Me*). HR-MALDI-MS (*m/z*): 764.3196 (100, $[M + H]^+$, $C_{31}H_{50}N_5O_{17}^+$; calc. 764.3202).

4’,6’-O-*(3,4,5-Trimethoxybenzylidene)paromomycin* (**22**). According to the standard deacetylation protocol reaction of 47 mg of **69** and FC gave 30 mg of **89** (81%). White solid. R_f ($CHCl_3$/AcOEt/MeOH 3:3.0.5) 0.22. Without characterization and according to Staudinger protocol **A**, reaction of 30 mg of **89** and FC gave 21 mg of **22** (82%). White solid. R_f (MeOH/25% aq. NH_3 4:1) 0.19. IR (ATR): 3352*w*, 3289*w*, 3182*w*, 2918*w*, 1593*w*, 1506*w*, 1462*w*, 1421*w*, 1380*w*, 1330*w*, 1236*w*, 1122*s*, 1026*s*, 994*s*. ^{1}H-NMR (500 MHz, D_2O): δ 6.93 (s, 2 arom. H); 5.70 (s, ArCH); 5.51 (d,

J = 3.9, H–C(1')); 5.38 (d, J = 2.4, H–C(1")); 5.02 (d, J = 1.2, H–C(1"')); 4.48 (dd, J = 6.6, 5.0, H–C(3")); 4.33 (dd, J = 5.0, 2.4, H–C(2")); 4.31 (dd, J = 10.4, 4.9, H_a–C(6')); 4.17–4.14 (m, H–C(4")); 4.11–4.06 (m, H–C(5"')); 4.05 (t, J = 3.3, H–C(3"')); 4.06–4.01 (m, H–C(5')); 3.91–3.89 (m, H_b–C(6'), H_a–C(5")); 3.89 (s, 2 MeO); 3.87 (t, J = 9.7, H–C(3')); 3.80 (s, MeO); 3.77–3.67 (m, H–C(4), H–C(5), H–C(4'), H–C(4"')); 3.52 (t, J = 9.3, H–C(4)); 3.36 (t, J = 9.8, H–C(6)); 3.23 (dd, J = 13.5, 8.0, H_a–C(6"')); 3.15 (dd, J = 13.6, 4.0, H_b–C(6"')); 3.10–3.08 (m, H–C(2"')); 2.99–2.94 (m, H–C(3)); 2.96 (dd, J = 10.0, 3.8, H–C(2')); 2.86 (ddd, J = 12.1, 10.0, 4.1, H–C(1)); 2.04 (dt, J = 13.0, 4.1, H_{eq}–C(2)); 1.29 (q, J = 12.8, H_{ax}–C(2)). ^{13}C-NMR (126 MHz, D_2O): δ 111.31 (d, C(1")); 106.65 (2d); 104.07 (d, ArCH)); 102.04 (d, C(1')); 101.41 (d, C(1"')); 87.12 (d, C(5)); 84.85 (d, C(4)); 84.04 (d, C(4'), C(4")); 79.35 (d, C(6)); 78.22 (d, C(3")); 76.11 (d, C(2")); 75.14 (d, C(5"')); 72.89 (d, C(3'), C(3"')); 71.18 (t, C(6'), d, C(4"')); 66.24 (d, C(5')); 63.59 (t, C(5"), q, MeO); 58.84 (d, C(2'), 2q, 2 MeO); 55.00 (d, C(2"')); 52.81 (d, C(3)); 52.33 (d, C(1)); 43.43 (t, C(6"')); 37.28 (t, C(2)). HR-MALDI-MS (*m*/*z*): 795.3686 (40); 794.3651 (100, [M + H]$^+$, $C_{33}H_{56}N_5O_{17}^+$; calc. 794.3651).

*4',6'-*O*-Furfurylideneparomomycin* (**23**). Application of the general deacetylation protocol to 73 mg of **70** and FC gave 48 mg of **90** (86%). White solid. R_f ($CHCl_3$/AcOEt/MeOH 3:3.0.5) 0.38. Without characterization, according to Staudinger protocol **A**, reaction of 49 mg of **90** and FC gave 39 mg of **23** (94%). White solid. R_f (MeOH/25% aq. NH_3 4:1) 0.21. IR (ATR): 3356*w* (br.), 2877*w*, 1664*w*, 1589*w*, 1506*w*, 1460*w*, 1460*w*, 1397*w*, 1359*w*, 1341*w*, 1135*s*, 1106*s*, 1026*s*, 994*s*, 918*s*. ^{1}H-NMR (500 MHz, D_2O): δ 7.58–7.57 (m, 1 arom. H); 6.63 (d, J = 3.4, 1 arom. H); 6.52–6.51 (m, 1 arom. H); 5.84 (s, ArCH); 5.52 (d, J = 3.8, H–C(1')); 5.38 (d, J = 2.4, H–C(1")); 4.98 (d, J = 1.9, H–C(1"')); 4.46 (dd, J = 6.6, 5.0, H–C(3")); 4.32 (dd, J = 5.0, 2.4, H–C(2")); 4.30 (dd, J = 10.4, 5.5, H_a–C(6')); 4.17–4.13 (m, H–C(4")); 4.06–3.98 (m, H–C(5'), H–C(5"')); 4.04 (t, J = 3.2, H–C(3"')); 3.92–3.85 (m, H_b–C(6'), H_a–C(5")); 3.82 (t, J = 9.7, H–C(3')); 3.75–3.66 (m, H–C(5), H–C(4'), H_b–C(5"), H–C(4"')); 3.49 (t, J = 9.3, H–C(4)); 3.29 (t, J = 9.6, H–C(6)); 3.11 (dd, J = 13.5, 8.3, H_a–C(6"')); 3.06–3.04 (m, H–C(2"')); 3.01 (dd, J = 13.5, 4.2, H_b–C(6"')); 2.98–2.93 (m, H–C(3)); 2.91 (dd, J = 10.0, 3.8, H–C(2')); 2.77 (ddd, J = 12.0, 9.9, 4.0, H–C(1)); 1.99 (dt, J = 12.4, 4.1, H_{eq}–C(2)); 1.24 (q, J = 12.7, H_{ax}–C(2)). ^{13}C-NMR (126 MHz, D_2O): δ 146.51 (d); 113.29 (d); 112.06 (d); 111.18 (d, C(1")); 101.78 (d, C(1'), C(1"')); 98.39 (d, ArCH)); 87.09 (d, C(5)); 84.83 (d, C(4)); 84.02 (d, C(4'), C(4"));

80.01 (d, C(6)); 78.41 (d, C(3”)); 76.88 (d, C(5”’)); 76.11 (d, C(2”)); 73.17 (d, C(3’), C(3”’)); 71.20 (t, C(6’), d, C(4”’)); 66.03 (d, C(5’)); 63.80 (t, C(5”)); 58.75 (d, C(2’)); 55.22 (d, C(2”’)); 52.97 (d, C(1)); 52.45 (d, C(3)); 43.65 (t, C(6”’)); 37.91 (t, C(2)). HR-MALDI-MS (*m*/*z*): 716.2997 (31, $[M + Na]^+$, $C_{28}H_{47}N_5NaO_{15}^+$; calc. 716.2966); 695.3146 (34, $[M + 2 H]^+$, $C_{28}H_{49}N_5O_{15}^+$; calc. 695.3225); 694.3128 (100, $[M + H]^+$, $C_{28}H_{48}N_5O_{15}^+$; calc. 694.3141).

4’,6’-O-*(2-Thienylidene)paromomycin* (**24**). According to the general acetalization protocol **A**, reaction of 80 mg of **60** for 22 h and FC gave 37 mg of **71** (42%). White solid. HR-MALDI-MS (*m*/*z*): 1130.2660 (52, $[M + K]^+$, $C_{40}H_{49}KN_{15}O_{20}S^+$; calc. 1130.2636); 1115.2904 (50); 1114.2864 (100, $[M + Na]^+$, $C_{40}H_{49}N_{15}NaO_{20}S^+$; calc. 1114.2897). Anal. calc. for $C_{40}H_{49}N_{15}O_{20}S$ (1091.98): C 44.00, H 4.52, N 19.24; found: C 43.86, H 4.61, N 19.23. Without further characterization, application of the general deacetylation protocol to 60 mg of **71** and FC gave 37 mg of **91** (80%). White solid. R_f ($CHCl_3$/AcOEt/MeOH 3:3.0.5) 0.40. Finally, without further characterization, according to Staudinger protocol **A**, reaction of 42 mg of **91** and FC gave 29 mg of **24** (82%). White solid. R_f (MeOH/25% aq. NH_3 4:1) 0.37. IR (ATR): 3353*w*, 3287*w*, 3174*w*, 2909*w*, 1595*w*, 1543*w*, 1445*w*, 1377*w*, 1332*w*, 1243*w*, 1100*s*, 1053*s*, 1023*s*, 974*s*, 926*s*. ^{1}H-NMR (500 MHz, D_2O): δ 7.54–7.52 (m, 1 arom. H); 7.30–7.29 (m, 1 arom. H); 7.12–7.10 (m, 1 arom. H); 6.05 (s, ArCH); 5.53 (d, *J* = 3.9, H–C(1’)); 5.39 (d, *J* = 2.4, H–C(1”)); 5.04 (d, *J* = 1.9, H–C(1”’)); 4.49 (dd, *J* = 6.6, 4.9, H–C(3”)); 4.34 (dd, *J* = 4.9, 2.4, H–C(2”)); 4.29 (dd, *J* = 10.4, 4.9, H_a–C(6’)); 4.18–4.12 (m, H–C(4”), H–C(5”’)); 4.07 (t, *J* = 3.3, H–C(3”’)); 4.04 (td, *J* = 9.9, 4.9, H–C(5’)); 3.93–3.87 (m, H_b–C(6’), H_a–C(5”)); 3.86 (t, *J* = 9.8, H–C(3’)); 3.77–3.70 (m, H–C(5), H–C(4’), H_b–C(5”), H–C(4”’)); 3.53 (t, *J* = 9.4, H–C(4)); 3.40 (t, *J* = 9.6, H–C(6)); 3.28 (dd, *J* = 13.6, 7.9, H_a–C(6”’)); 3.20 (dd, *J* = 13.5, 3.9, H_b–C(6”’)); 3.13–3.11 (m, H–C(2”’)); 3.00 (ddd, *J* = 12.2, 9.5, 4.2, H–C(3)); 2.97 (dd, *J* = 10.0, 3.9, H–C(2’)); 2.91 (ddd, *J* = 12.1, 10.0, 4.1, H–C(1)); 2.06 (dt, *J* = 12.9, 4.3, H_{eq}–C(2)); 1.33 (q, *J* = 12.7, H_{ax}–C(2)). ^{13}C-NMR (126 MHz, D_2O): δ 129.59 (3d); 111.34 (d, C(1”)); 101.81 (d, C(1’)); 101.19 (d, C(1”’), ArCH); 86.95 (d, C(5)); 84.46 (d, C(4)); 83.90 (d, C(4”)); 83.57 (d, C(4’)); 78.87 (d, C(6)); 78.10 (d, C(3”)); 76.08 (d, C(2”)); 74.67 (d, C(5”’)); 72.75 (d, C(3’), C(3”’)); 71.13 (t, C(6’), d, C(4”’)); 66.13 (d, C(5’)); 63.45 (t, C(5”)); 58.54 (d, C(2’)); 54.90 (d, C(2”’)); 53.04 (d,

C(1)); 52.22 (d, C(3)); 43.44 (t, C(6''')); 36.83 (t, C(2)). HR-MALDI-MS (*m*/*z*): 732.2730 (100, $[M + Na]^+$, $C_{28}H_{47}N_5NaO_{14}S^+$; calc. 732.2738).

*Monoacetate of 4',6'-*O*-(Cyclohexylmethylidene)paromomycin* (**25**). According to the general acetalization protocol **A**, reaction of 157 mg of **60** for 5 h and FC gave 83 mg of **72** (49%). White solid. ^{1}H-NMR (300 MHz, $CDCl_3$): δ 5.80 (d, *J* = 3.9, H–C(1')); 5.45 (t, *J* = 10.2, H–C(3')); 5.33 (d, *J* = 2.3, H–C(1'')); 5.02 (t , *J* = 2.8, H–C(3''')); 4.91 (t, *J* = 9.9, H–C(6)); 4.89–4.86 (m, H–C(2''), H–C(1''')); 4.70–4.68 (m, H–C(4'''));4.43–4.37 (m, H–C(3''), H_a–C(5'')); 4..29 (td, *J* = 4.9, 1.9, H–C(4'')); 4.23–4.07 (m, H–C(5'), H_a–C(6'), H_b–C(5''), H–C(5''')); 3.88 (t, *J* = 8.8, H–C(5)); 3.68–3.23 (m, H–C(1), H–C(3), H–C(4), H_b–C(6'), H_a–C(6''')); 3.32–3.30 (m, H–C(2''')); 3.24 (dd, *J* = 13.0, 4.2, H_b–C(6''')); 3.02 (dd, *J* = 10.4, 3.9, H–C(2')); 2.40–2.28 (m, H_{eq}–C(2), 1 H); 2.165, 2.156, 2.15, 2.12, 2.11, 2.08 (6s, 6 AcO); 1.96–1.85 (m, 1 H); 1.80–0.9 (m, H_{ax}–C(2), 5 CH_2). ^{13}C-NMR (75 MHz, $CDCl_3$): δ 170.71, 170.12, 170.02, 169.70, 169.65, 168.51 (6s, 6 C=O); 107.06 (d, C(1'')); 105.42 (d, $C_6H_{11}CH$); 99.10 (d, C(1''')); 97.50 (d, C(1')); 82.16 (d, C(5)); 79.25 (d, C(4'')); 78.64 (d, C(4')); 76.40 (d, C(4)); 75.81 (d, C(3'')); 75.35 (d, C(6)); 74.76 (d, C(2'')); 73.54 (d, C(5''')); 69.11 (d, C(3')); 68.70 (d, C(3''')); 68.26 (t, C(6')); 65.71 (d, C(4''')); 63.59 (t, C(5'')); 63.52 (d, C(5')); 61.42 (d, C(2')); 59.04 (d, C(3)); 58.04 (d, C(1)); 56.47 (d, C(2''')); 50.63 (t, C(6''')); 31.42 (t, C(2)); 20.88, 20.77, 20.67, 20.55 (4q, 6 Me). Without further characterization, application of the general deacetylation protocol to 46 mg of **72** and FC gave 26 mg of **92** (74%). White solid. R_f ($CHCl_3$/AcOEt/MeOH 3:3.0.5) 0.37. Without characterization, according to Staudinger protocol **A**, reaction of 26 mg of **92** gave 16 mg of **25** (73%) of which 14 mg were stirred in 20% aq. AcOH and taken to dryness. White solid. R_f (MeOH/25% aq. NH_3 4:1) 0.25. IR (ATR): 3121*m*, 3034*m*, 2929*m*, 2855*m*, 1753*w*, 1712*w*, 1613*w*, 1514*w*, 1441*w*, 1399*m*, 1101*s*, 996*s*. ^{1}H-NMR (300 MHz, D_2O): δ 5.99 (d, *J* = 4.0, H–C(1')); 5.45 (s, H–C(1'')); 5.33 (s, H–C(1''')); 4.59–4.47 (m, 2 H); 4.39–4.36 (m, 1 H); 4.27–4.26 (m, 2 H); 4.20–3.92 (m, 3 H); 3.86–3.35 (m, 9 H); 2.59–2.50 (m, H_{eq}–C(2)); 2.07 (s, CH_3CO_2H); 1.95 (q, *J* = 11.8, H_{ax}–C(2)); 1.80–1.57 (m, 4 H); 1.30–1.05 (m, 4 H). ^{13}C-NMR (75 MHz, D_2O): δ 112.97; 108.63; 98.61; 97.78; 87.36; 83.94; 82.16; 78.15; 77.50; 75.95; 75.12; 72.95; 70.34; 69.87; 68.88; 66.95; 62.88; 57.01; 53.52; 52.53; 51.15; 43.78; 43.08; 30.70; 29.75; 29.58; 27.79; signals for CH_3CO_2H not seen. HR-MALDI-MS (*m*/*z*): 711.3859 (34); 710.3822 (100, $[M + H]^+$,

$C_{30}H_{56}N_5O_{14}^+$; calc. 710.3824).

4',6'-O-*[(1-Naphthyl)methylidene]paromomycin* (**26**). Application of the standard deacetylation protocol to 107 mg of **73** and FC gave 75 mg of **93** (90%). White solid. R_f ($CHCl_3$/AcOEt/MeOH 3:3.0.5) 0.52. ^{1}H-NMR (300 MHz, CD_3OD): δ 8.40–8.37 (m, 1 arom. H); 7.90–7.83 (m, 2 arom. H); 7.74–7.69 (m, 1 arom. H); 7.51–7.42 (m, 3 arom. H); 6.21 (s, ArCH); 5.90 (d, *J* = 3.7, H–C(1')); 5.41 (d, *J* = 1.9, H–C(1'')); 5.15 (d, *J* = 1.6, H–C(1''')); 4.44 (dd, *J* = 6.5, 4.7, H–C(3'')); 4.38–4.27 (m, H–C(2''), H_a–C(6'), H–C(5')); 4.22–4.13 (m, H–C(4''), H–C(3')); 4.02 (ddd, *J* = 8.4, 4.7, 1.9, H–C(5''')); 3.94 (t, *J* = 3.4, H–C(3''')); 3.93–3.82 (m,, H_a–C(5''), H_b–C(6')); 3.77–3.62 (m, H–C(2'''), H_b–C(5''), H–C(4), H–C(5), H_a–C(6''') , H–C(4')); 3.62–3.51 (m, H–C(3)); 3.50–3.38 (m, H–C(4'''), H–C(1), H–C(6)); 3.39 (dd, *J* = 13.1, 4.4, H_b–C(6''')); 3.34–3.27 (H–C(2')); 2.22 (dt, *J* = 12.8, 4.0, H_{eq}–C(2)); 1.41 (q, *J* = 12.1, H_{ax}–C(2)). ^{13}C-NMR (75 MHz, CD_3OD): δ 135.40, 134.37, 132.02, 130.80, 129.41, 127.03, 126.76, 126.27, 126.24, 125.94 (3s, 7d, 10 arom. C); 109.89 (d, C(1'')); 103.46 (d, ArCH); 99.83 (d, C(1''')); 99.09 (d, C(1')); 85.31 (d, C(5)); 83.51 (d, C(4'')); 83.39 (d, C(4')); 77.62 (d, C(4)); 77.36, 77.33 (2d, C(3''), C(6)); 75.68 (d, C(5''')); 75.15 (d, C(2'')); 71.18 (d, C(3''')); 70.10 (t, C(6')); 69.65 (2d, C(4'''), C(3')); 65.22 (d, C(2')); 64.65 (d, C(5')); 63.76 (t, C(5'')); 61.93 (d, C(1)); 61.83 (d, C(2''')); 61.54 (d, C(3)); 52.52 (t, C(6''')); 33.14 (t, C(2)). Without further characterization, according to Staudinger protocol **A**, reaction of 49 mg of **93** and FC gave 39 mg of **26** (93%). White solid. R_f (MeOH/25% aq. NH_3 4:1) 0.32. IR (ATR): 3345*w*, 2888*w*, 1599*w*, 1532*w*, 1509*w*, 1460*w*, 1392*w*, 1339*w*, 1272*w*, 1246*w*, 1104*s*, 1054*s*, 1028*s*, 996*s*, 972*s*, 920*s*. ^{1}H-NMR (500 MHz, D_2O):, δ 8.32 (d, *J* = 8.3, 1 arom. H); 8.05–8.01 (m, 2 arom. H); 7.83–7.81 (m, 1 arom. H); 7.68–7.57 (m, 3 arom. H); 6.33 (s, ArCH); 5.55 (d, *J* = 3.9, H–C(1')); 5.40 (d, *J* = 2.4, H–C(1'')); 5.06 (d, *J* = 1.8, H–C(1''')); 4.50 (dd, *J* = 6.6, 5.0, H–C(3'')); 4.38 (dd, *J* = 10.3, 4.9, H_a–C(6')); 4.35 (dd, *J* = 4.9, 2.4, H–C(2'')); 4.22–4.15 (m, H–C(5'), H–C(4'')); 4.13 (ddd, *J* = 7.5, 3.8, 1.5, H–C(5''')); 4.08 (t, *J* = 3.2, H–C(3''')); 4.03 (t, *J* = 10.4, H_b–C(6')); 3.95 (t, *J* = 9.6, H–C(3')); 3.90 (dd, *J* = 12.5, 3.2, H_a–C(5'')); 3.85 (*t*, J = 9.5, H–C(4')); 3.78–3.71 (m, H–C(5), H_b–C(5'')); 3.69–3.68 (m, H–C(4''')); 3.58 (t, *J* = 9.4, H–C(4)); 3.48 (t, *J* = 9.7, H–C(6)); 3.30 (dd, *J* = 13.6, 7.6, H_a–C(6''')); 3.22 (dd, *J* = 13.6, 3.9, H_b–C(6''')); 3.19–3.17 (m, H–C(2''')); 3.07 (dd, *J* = 10.0, 3.8, H–C(2')); 2.98 (qd, *J* = 12.0, 4.1, H–C(1), H–C(3)); 2.11 (dt, *J* = 13.0, 4.2, H_{eq}–C(2)); 1.40 (q, *J* = 12.6, H_{ax}–C(2)).

^{13}C-NMR (126 MHz, D_2O): δ 133.10 (d); 131.48 (d); 129.47 (2d); 128.08 (2d); 126.62 (d); 111.24 (d, C(1”)); 103.76 (d, ArCH); 101.57 (d, C(1’)); 100.62 (d, C(1”’)); 86.67 (d, C(5)); 84.37 (d, C(4)); 83.92 (d, C(4’), C(4”)); 77.97 (d, C(6), C(3”)); 76.06 (d, C(2”)); 73.90 (d, C(5”’)); 72.32 (d, C(3’), C(3”’)); 70.97 (t, C(6’), d, C(4”’)); 66.47 (d, C(5’)); 63.27 (t, C(5”)); 58.47 (d, C(2’)); 54.62 (d, C(2”’)); 53.08 (d, C(3)); 51.99 (d, C(1)); 43.34 (t, C(6”’)); 36.02 (t, C(2)). HR-MALDI-MS (*m*/*z*): 755.3519 (40); 754.3495 (100, $[M + H]^+$, $C_{34}H_{52}N_5O_{14}^+$; calc. 754.3511).

4’,6’-O-*[(2-Naphthyl)idene]paromomycin* (**27**). According to the general acetalization protocol **B**, reaction of 80 mg of **60** for 23 h and FC gave 60 mg of **74** (66%). R_f (hexane/AcOEt 1:1) 0.64. Without further characterization, application of the standard deacetylation protocol to 60 mg of **74** and FC gave 30 mg of **94** (65%). White solid. R_f ($CHCl_3$/AcOEt/MeOH 3:3.0.5) 0.50. ESI-MS: 906.0 (100, $[M + Na]^+$, $C_{34}H_{41}N_{15}NaO_{14}^+$; calc. 906.3). Anal. calc. for $C_{34}H_{41}N_{15}O_{14}$ (883.79): C 46.21, H 4.68, N 23.77; found: C 46.44, H 4.54, N 23.37. Finally, without further characterization, according to Staudinger protocol **A**, reaction of 19 mg of **94** and FC gave 12 mg of **27** (74%). White solid. R_f (MeOH/25% aq. NH_3 4:1) 0.36. IR (ATR): 3180*w*, 2895*w*, 1600*w*, 1530*w*, 1467*w*, 1374*w*, 1346*w*, 1048*s*, 1026*s*. ^1H-NMR (500 MHz, D_2O):, δ 8.10 (s, 1 arom. H); 8.03–7.98 (m, 3 arom. H); 7.69–7.61 (m, 3 arom. H); 5.93 (s, ArCH); 5.55 (d, *J* = 3.9, H–C(1’)); 5.38 (d, *J* = 2.4, H–C(1”)); 5.08 (d, *J* = 1.8, H–C(1”’)); 4.50 (dd, *J* = 6.6, 5.0, H–C(3”)); 4.36 (dd, *J* = 10.4, 4.9, H_a–C(6’)); 4.35 (dd, *J* = 4.8, 2.5, H–C(2”)); 4.19–4.15 (m, H–C(4”), H–C(5”’)); 4.12–4.06 (m, H–C(5)); 4.09 (t, *J* = 3.3, H–C(3”’)); 3.96 (t, *J* = 10.4, H_b–C(6’)); 3.93 (t, *J* = 9.7, H–C(3’)); 3.90 (dd, *J* = 12.5, 3.1, H_a–C(5”)); 3.77 (t, *J* = 9.4, H–C(4’)); 3.77–3.69 (m, H–C(5), H_b–C(5”), H–C(4”’)); 3.55 (t, *J* = 9.4, H–C(4)); 3.45 (t, *J* = 10.0, H–C(6)); 3.33 (dd, *J* = 13.5, 7.6, H_a–C(6”’)); 3.26 (dd, *J* = 13.6, 4.0, H_b–C(6”’)); 3.19–3.18 (m, H–C(2”’)); 3.04 (dd, *J* = 10.1, 3.9, H–C(2’)); 3.00–2.94 (m, H–C(1), H–C(3)); 2.10 (dt, *J* = 13.0, 4.3, H_{eq}–C(2)); 1.38 (q, *J* = 12.6, H_{ax}–C(2)). ^{13}C-NMR (126 MHz, D_2O): δ 131.18 (3d); 129.68 (2d); 128.67 (d); 126.25 (d); 111.48 (d, C(1”)); 104.55 (d, ArCH)); 101.66 (d, C(1’)); 100.74 (d, C(1”’)); 86.88 (d, C(5)); 83.93 (d, C(4), C(4”)); 83.53 (d, C(4’)); 78.04 (d, C(6), C(3”)); 76.08 (d, C(2”)); 74.00 (d, C(5”’)); 72.40 (d, C(3’), C(3”’)); 71.00 (t, C(6’), d, C(4”’)); 66.42 (d, C(5’)); 63.32 (t, C(5”)); 58.46 (d, C(2’)); 54.69 (d, C(2”’)); 53.08, 52.12 (2d, C(1), C(3)); 43.38 (t, C(6”’)); 36.11 (t, C(2)). HR-MALDI-MS (*m*/*z*): 776.3309 (56, $[M + Na]^+$, $C_{34}H_{51}N_5NaO_{14}^+$; calc. 776.3330); 755.3538 (36);

754.3509 (100, $[M + H]^+$, $C_{34}H_{52}N_5O_{14}^+$; calc. 754.3511); 594.2633 (100, $[M$ – ring IV + 2 H$]^+$, $C_{28}H_{40}N_3O_{11}^+$; calc. 594.2663); 462.2238 (63, $[M$ – ring III – ring IV + 2 H$]^+$, $C_{23}H_{32}N_3O_7^+$; calc. 462.2240).

*4',6'-*O*-(4-Phenylbenzylidene)paromomycin* (**28**). Application of the Staudinger protocol to 20 mg of **57** and FC gave 16 mg of **28** (93%). White solid. R_f (MeOH/25% aq. NH_3 4:1) 0.31. $[\alpha]_D^{25}$ = +42.3 (*c* = 0.14, H_2O). IR (ATR): 3356*w*, 3284*w*, 2903*w*, 1599*w*, 1532*w*, 1489*w*, 1451*w*, 1375*w*, 1110*s*, 1074*s*, 1026*s*. ^{1}H-NMR (500 MHz, D_2O): δ 7.58–7.27 (m, 9 arom. H); 5.62 (s, *J* = ArCH); 5.39 (br. s, H–C(1')); 5.26 (br. s, H–C(1")); 5.00 (d, *J* = 1.5, H–C(1"')); 4.41–4.38 (m, H–C(3")); 4.25 (dd, *J* = 4.7, 2.2, H–C(2")); 4.12–4.10 (m, H–C(5"')); 4.07–4.04 (m, H–C(4")); 4.01 (t, *J* = 3.1, H–C(3"')); 3.92–3.91 (m, H–C(5')); 3.80–3.75 (m, H_a–C(5"), Ha–C(6'), H–C(3')); 3.67–3.60 (m, H_b–C(5"), H–C(4"')); 3.59–3.55 (m, H–C(4'), H–C(5)); 3.42 (t, *J* = 9.1, H–C(4)); 3.36 (t, *J* = 9.7, H–C(6)); 3.24 (dd, *J* = 13.6, 7.7, H_a–C(6"')); 3.17 (dd, *J* = 13.5, 3.9, H_b–C(6"')); 3.11 (br. s, H–C(2"')); 2.91 (dd, *J* = 10.1, 3.3, H–C(2')); 2.83–2.73 (m, H–C(3), H–C(1)); 1.93–1.91 (m, H_{eq}–C(2)); 1.28–1.23 (m, H_{ax}–C(2)). ^{13}C-NMR (126 MHz, D_2O): δ 131.83, 130.66, 129.65 (9 arom. C); 111.61 (d, C(1")); 104.22 (d, ArCH); 101.95 (d, C(1')); 100.69 (d, C(1"')); 86.87 (d, C(5)); 84.90 (d, C(4)); 83.94 (2d, C(4'), C(4")); 78.01 (2d, C(6), C(3")); 76.03 (d, C(2")); 74.03 (d, C(5"')); 72.37 (2d, C(3'), C(3"')); 70.92 (d, C(4"'), t, C(6')); 66.37 (d, C(5')); 63.37 (t, C(5")); 58.47 (d, C(2')); 54.63 (d, C(2"')); 53.06, 52.21 (2d, C(1), C(3)); 43.34 (t, C(6"')); 36.22 (t, C(2)). HR-MALDI-MS (*m/z*): 803.3565 (41); 802.3462 (100, $[M + Na]^+$, $C_{36}H_{53}N_5NaO_{14}^+$; calc. 802.3487); 780.3616 (48, $[M + H]^+$, $C_{36}H_{54}N_5O_{14}^+$; calc. 802.3481).

*4',6'-*O*-(2-Phenylethylidene)paromomycin* (**29**). Application of the standard deacetylation protocol to 79 mg of **75** and FC gave 55 mg of **95** (90%). White solid. R_f ($CHCl_3$/AcOEt/MeOH 3:3.0.5) 0.46. Without further characterization, according to Staudinger protocol **A**, reaction of 52 mg of **95** and FC gave 39 mg of **29** (89%). White solid. R_f (MeOH/25% aq. NH_3 4:1) 0.33. IR (ATR): 3286*w*, 2888*w*, 1664*w*, 1571*w*, 1472*w*, 1392*w*, 1341*w*, 1102*s*, 1017*s*, 995*s*, 937*w*, 907*w*. ^{1}H-NMR (500 MHz, D_2O):δ 7.42–7.39 (m, 2 arom. H); 7.36–7.32 (m, 3 arom. H); 5.50 (d, *J* = 3.9, H–C(1')); 5.37 (d, *J* = 2.4, H–C(1")); 5.05 (d, *J* = 1.8, H–C(1"')); 5.03 (dd, *J* = 5.9, 4.6,

PhCH$_2$CH); 4.48 (dd, *J* = 6.7, 5.0, H–C(3”)); 4.32 (dd, *J* = 4.9, 2.4, H–C(2”)); 4.18–4.14 (m, H–C(4”), H–C(5”’)); 4.12 (dd, *J* = 10.5, 5.0, H$_a$–C(6’)); 4.08 (t, *J* = 3.3, H–C(3”’)); 3.90–3.85 (m, H–C(5’)); 3.88 (dd, *J* = 12.3, 3.2, H$_a$–C(5”)); 3.81 (t, *J* = 9.7, H–C(3’)); 3.75–3.71 (m, H–C(5), H$_b$–C(5”), H–C(4”’)); 3.62 (t, *J* = 10.5, H$_b$–C(6’)); 3.502 (t, *J* = 9.3, H–C(4)); 3.497 (t, *J* = 9.5, H–C(4’)); 3.41 (t, *J* = 9.9, H–C(6)); 3.30 (dd, *J* = 13.6, 7.8, H$_a$–C(6”’)); 3.23 (dd, *J* = 13.5, 3.9, H$_b$–C(6”’)); 3.16–3.15 (m, H–C(2”’)); 3.08 (dd, *J* = 14.4, 4.6, PhCH$_a$H$_b$CH); 3.01–2.91 (m, PhCH$_a$H$_b$CH, H–C(1), H–C(3)); 2.94 (dd, *J* = 10.0, 3.8, H–C(2’)); 2.07 (dt, *J* = 12.9, 4.1, H$_{eq}$–C(2)); 1.34 (q, *J* = 12.7, H$_{ax}$–C(2)). ^{13}C-NMR (126 MHz, D$_2$O): δ 132.26 (2d); 131.43 (2d); 129.77 (d); 111.49 (d, C(1”)); 105.26 (d, PhCH$_2$CH); 101.62 (d, C(1’)); 100.97 (d, C(1”’)); 86.99 (d, C(5)); 84.20 (d, C(4)); 83.89 (d, C(4”)); 83.18 (d, C(4’)); 78.50 (d, C(6)); 78.04 (d, C(3”)); 76.06 (d, C(2”)); 74.38 (d, C(5”’)); 72.22 (2d, C(3’), C(3”’)); 71.09 (d, C(4”’)); 70.32 (t, C(6’)); 66.26 (d, C(5’)); 63.07 (t, C(5”)); 58.43 (d, C(2’)); 54.79 (d, C(2”’)); 53.05 (d, C(1)); 52.19 (d, C(3)); 43.40 (t, C(6”’)); 42.29 (t, PhCH$_2$); 36.43 (t, C(2)). HR-MALDI-MS (*m/z*): 741.3349 (37); 740.3312 (100, [*M* + Na]$^+$, $C_{31}H_{51}N_5NaO_{14}^+$; calc. 740.3330).

*4’,6’-*O*-(3-Phenylpropylidene)paromomycin* (**30**). Application of the standard deacetylation protocol to of 127 mg of **76** and FC gave 75 mg of **96** (76%). White solid. *R*$_f$ (CHCl$_3$/AcOEt/MeOH 3:3.0.5) 0.45. Without further characterization, according to Staudinger protocol **A**, reaction of 52 mg of **96** and FC gave 35 mg of **30** (79%). White solid. *R*$_f$ (MeOH/25% aq. NH$_3$ 4:1) 0.27. IR (ATR): 3356*w*, 2870*w*, 1740*w*, 1594*w*, 1495*w*, 1454*w*, 1386*w*, 1119*s*, 1016*s*, 931*s*. ^{1}H-NMR (500 MHz, D$_2$O):, δ 7.40–7.37 (m, 2 arom. H); 7.33–7.27 (m, 3 arom. H); 5.47 (d, *J* = 3.8, H–C(1’)); 5.37 (d, *J* = 2.5, H–C(1”)); 5.00 (d, *J* = 1.8, H–C(1”’)); 4.68 (t, *J* = 5.3, Ph(CH$_2$)$_2$CH); 4.46 (dd, *J* = 6.6, 5.0, H–C(3”)); 4.31 (dd, *J* = 4.9, 2.5, H–C(2”)); 4.18–4.13 (m, H$_a$–C(6’), H–C(4”)); 4.07–4.04 (m, H–C(5”’), H–C(3”’)); 3.90–3.85 (m, H$_a$–C(5”), H–C(5’)); 3.78–3.65 (m, H–C(3’), H–C(5), H$_b$–C(5”), H–C(4”’)); 3.62 (t, *J* = 10.5, H$_b$–C(6’)); 3.47 (t, *J* = 9.3, H–C(4)); 3.42 (t, *J* = 9.6, H–C(4’)); 3.40 (t, *J* = 9.7, H–C(6); 3.17 (dd, *J* = 13.5, 8.5, H$_a$–C(6”’)); 3.09–3.06 (m, H$_b$–C(6”’), H–C(2”’)); 2.93 (ddd, *J* = 12.2, 9.5, 4.1, H–C(3)); 2.86 (dd, *J* = 10.0, 3.8, H–C(2’)); 2.82–2.75 (m, H–C(1), PhCH$_2$CH$_2$); 2.03–1.95 (m, H$_{eq}$–C(2), PhCH$_2$CH$_2$); 1.25 (q, *J* = 12.4, H$_{ax}$–C(2)). ^{13}C-NMR (126 MHz, D$_2$O): δ 131.33 (4d); 128.93 (d); 111.23 (d, C(1”)); 104.67 (d, Ph(CH2)2CH); 101.65 (2d, C(1’), C(1”’)); 87.05 (d, C(5)); 84.84 (d, C(4)); 83.98 (d, C(4”)); 83.26 (d, C(4’)); 79.74 (d, C(6)); 78.34 (d, C(3”)); 76.11 (2d, C(2”),

C(5’’’)); 73.07 (2d, C(3’), C(3’’’)); 71.21 (d, C(4’’’)); 70.33 (t, C(6’)); 66.37 (d, C(5’)); 63.67 (t, C(5’’)); 58.66 (d, C(2’)); 55.12 (d, C(2’’’)); 52.98 (d, C(1)); 43.59 (t, C(6’’’)); 37.52 (t, CH2, C(2)); 32.25 (t, CH_2). HR-MALDI-MS (*m*/*z*): 755.3505 (38); 754.3467 (100, $[M+Na]^+$, $C_{32}H_{53}N_5NaO_{14}^+$; calc. 754.3481); 732.3642 (38, $[M+H]^+$, $C_{32}H_{54}N_5O_{14}^+$; calc. 732.3667).

4’,6’-O-*[(E)-Cinnamylidene)]paromomycin* (**31**). According to the general acetalization protocol **A**, reaction of 90 mg of **60** for 2 h and FC gave 57 mg of **77** (57%). Without further characterization, application of the standard deacetylation protocol to 41 mg of **77** and FC gave 24 mg of **97** (76%). White solid. R_f ($CHCl_3$/AcOEt/MeOH 3:3.0.5) 0.48. Finally, without further characterization,according to Staudinger protocol **A**, reaction of 24 mg of **97** and FC gave 19 mg of **31** (92%). White solid. R_f (MeOH/25% aq. NH_3 4:1) 0.34. IR (ATR): 3353*w*, 3288*w*, 2915*w*, 1593*w*, 1493*w*, 1451*w*, 1377*w*, 1336*w*, 1135*s*, 1115*s*, 1054*s*, 1022*s*, 994*s*, 970*s*. ^{1}H-NMR (500 MHz, D_2O): δ 7.56–7.54 (m, 2 arom. H); 7.46–7.38 (m, 3 arom. H); 6.95 (d, *J* = 16.1, PhCH=CH); 6.28 (dd, *J* = 16.2, 5.2, PhCH=CH); 5.52 (d, *J* = 3.8, H–C(1’)); 5.40 (dd, *J* = 5.2, 0.9, PhCH=CHCH); 5.38 (d, *J* = 2.4, H–C(1’’)); 5.05 (d, *J* = 1.8, H–C(1’’’)); 4.48 (dd, *J* = 6.6, 5.0, H–C(3’’)); 4.33 (dd, *J* = 4.9, 2.4, H–C(2’’)); 4.26 (dd, *J* = 10.3, 4.9, H_a–C(6’)); 4.18–4.14 (m, H–C(4’’), H–C(5’’’)); 4.07 (t, *J* = 3.3, H–C(3’’’)); 3.96 (td, *J* = 10.1, 5.0, H–C(5’)); 3.89 (dd, *J* = 12.5, 3.3, H_a–C(5’’)); 3.84 (t, *J* = 9.8, H–C(3’)); 3.84–3.79 (m, H_b–C(6’)); 3.77–3.70 (m, H–C(5), H_b–C(5’’), H–C(4’’’)); 3.63 (t, *J* = 9.5, H–C(4’)); 3.52 (t, *J* = 9.4, H–C(4)); 3.40 (t, *J* = 9.9, H–C(6)); 3.29 (dd, *J* = 13.5, 7.8, H_a–C(6’’’)); 3.21 (dd, *J* = 13.5, 4.0, H_b–C(6’’’)); 3.14–3.13 (m, H–C(2’’’)); 3.01–2.95 (m, H–C(3)); 2.96 (dd, *J* = 10.1, 3.9, H–C(2’)); 2.91 (ddd, *J* = 12.1, 10.0, 4.1, H–C(1)); 2.06 (dt, *J* = 13.0, 4.2, H_{eq}–C(2)); 1.33 (q, *J* = 12.5, H_{ax}–C(2)). ^{13}C-NMR (126 MHz, D_2O): δ 137.97 (d, PhCH); 131.66 (3d); 129.65 (2d); 125.86 (d, PhCH=CH); 111.41 (d, C(1’’)); 103.91 (d, PhCHCHCH); 101.84 (d, C(1’)); 101.13 (d, C(1’’’)); 86.96 (d, C(5)); 84.46 (d, C(4)); 83.92 (d, C(4’’)); 83.19 (d, C(4’)); 78.74 (d, C(6)); 78.12 (d, C(3’’)); 76.07 (d, C(2’’)); 74.59 (d, C(5’’’)); 72.70 (d, C(3’), C(3’’’)); 71.10 (d, C(4’’’)); 70.43 (t, C(6’)); 66.28 (d, C(5’)); 63.46 (t, C(5’’)); 58.58 (d, C(2’)); 54.87 (d, C(2’’’)); 53.05 (d, C(1)); 52.27 (d, C(3)); 43.43 (t, C(6’’’)); 36.71 (t, C(2)). HR-MALDI-MS (*m*/*z*): 731.3519 (38); 730.3492 (100, $[M+H]^+$, $C_{32}H_{52}N_5O_{14}^+$; calc. 730.3511).

4’,6’-O-*[3-(4-Methoxy)phenyl]propylideneparomomycin* (**32**). According to the

general acetalization protocol **B**, reaction of 121 mg of **60** for 3 h 40 and FC (cyclohexane/AcOEt 7:3) gave 108 mg of **78** (78%). White solid. R_f (hexane/AcOEt 1:1) 0.74. ^{1}H-NMR (300 MHz, $CDCl_3$): δ 7.08 (d, J = 8.5, 2 arom. H); 6.82 (d, J = 8.4, 2 arom. H); 5.80 (d, J = 3.8, H–C(1')); 5.48 (t, J = 9.9, H–C(3')); 5.33 (d, J = 2.3, H–C(1")); 5.02 (t, J = 2.7, H–C(3"')); 4.92 (t, J = 9.6, H–C(6)); 4.91–4.86 (m, H–C(2"), H–C(1"')); 4.69 (br. s, H–C(4"')); 4.45–4.37 (m, H–C(3"), H_a–C(5"), $Ar(CH_2)_2CH$); 4.29 (td, J = 4.7, 1.9, H–C(4")); 4.15 (dd, J = 12.1, 1.9, H_b–C(5")); 4.15–4.07 (m, H–C(5'), H_a–C(6'), H–C(5"')); 3.88 (t, J = 8.9, H–C(5)); 3.78 (s, OMe); 3.64 (t, J = 9.4, H–C(4)); 3.58 (dd, J = 13.0, 8.2, H_a–C(6"')); 3.52–3.25 (m, H–C(1), H–C(3), H–C(4'), H_b–C(6'), H–C(2"')); 3.24 (dd, J = 13.0, 4.1, H_b–C(6"')); 3.01 (dd, J = 10.4, 3.8, H–C(2')), 2.62 (t, J = 7.7, $PhCH_2$); 2.38 (dt, J = 13.3, 4.5, H_{eq}–C(2)); 2.163, 2.158, 2.15, 2.11, 2.07 (5s, 6 AcO); 1.94–1.86 (m, $ArCH_2CH_2$); 1.59 (q, J = 12.8, H_{ax}–C(2)). HR-MALDI-MS (*m/z*): 1167. 3804 (52); 1166.3763 (100, $[M + Na]^+$, $C_{45}H_{57}N_{15}NaO_{21}^+$; calc. 1166.3751). Anal. calc. for $C_{45}H_{57}N_{15}O_{21}$ (1144.03): C 47.24, H 5.02, N 18.36; found: C 47.23, H 5.06, N 18.11. Application of the standard deacetylation protocol to 82 mg of **78** for 6 h and FC ($CHCl_3$/AcOEt/MeOH 1:1:0 → 3:3:0.1) gave 45 mg of **98** (67%). White solid. R_f ($CHCl_3$/AcOEt/MeOH 3:3.0.5) 0.41. Finally, without further characterization, according to Staudinger protocol **A**, reaction of 45 mg of **98** and FC gave 38 mg of **32** (99%). White solid. R_f (MeOH/25% aq. NH_3 4:1) 0.30. IR (ATR): 3289*w* (br.), 2907*w*, 1582*w*, 1512*m*, 1460*w*, 1385*w*, 1337*w*, 1300*w*, 1244*w*, 1119*s*, 1106*s*, 1018*s*, 937*s*. ^{1}H-NMR (500 MHz, D_2O): δ 7.24 (d, J = 8.6, 2 arom. H); 6.97 (d, J = 8.6, 2 arom. H); 5.49 (d, J = 3.9, H–C(1')); 5.37 (d, J = 2.4, H–C(1")); 5.06 (d, J = 1.8, H–C(1"')); 4.66 (t, J = 5.1, $Ar(CH_2)_2CH$); 4.48 (dd, J = 6.5, 5.0, H–C(3")); 4.33 (dd, J = 4.9, 2.5, H–C(2")); 4.18–4.14 (m, H_a–C(6'), H–C(4"), H–C(5"')); 4.08 (t, J = 3.3, H–C(3"')); 3.88 (dd, J = 12.0, 3.1, H_a–C(5")); 3.89–3.84 (m, H–C(5')); 3.83 (s, MeO); 3.79 (t, J = 9.8, H–C(3')); 3.77–3.71 (m, H–C(5), H_b–C(5"), H–C(4"')); 3.61 (t, J = 10.7, H_b–C(6')); 3.50 (t, J = 9.3, H–C(4)); 3.42 (t, J = 9.5, H–C(4')); 3.41 (t, J = 9.7, H–C(6)); 3.30 (dd, J = 13.6, 7.8, H_a–C(6"')); 3.23 (dd, J = 13.6, 4.0, H_b–C(6"')); 3.15–3.14 (m, H–C(2"')); 3.00–2.90 (m, H–C(3), H–C(1)); 2.92 (dd, J = 10.0, 3.7, H–C(2')); 2.70 (t, J = 7.6, $ArCH_2$); 2.07 (dt, J = 13.0, 4.1, H_{eq}–C(2)); 1.98–1.90 (m, $ArCH_2CH_2$); 1.34 (q, J = 12.6, H_{ax}–C(2)). ^{13}C-NMR (126 MHz, D_2O): δ 159.81 (s); 136.68 (s); 132.39 (2d); 116.80 (2d); 111.41 (d, C(1")); 104.65 (d, $ArCH_2CH_2CH$); 101.72 (d, C(1')); 101.04 (d, C(1"')); 86.93 (d, C(5)); 84.41 (d,

C(4)); 83.90 (d, C(4”)); 83.11 (d, C(4’)); 78.56 (d, C(6)); 78.10 (d, C(3”)); 76.04 (d, C(2”)); 74.41 (d, C(5’”)); 72.60 (d, C(3’”)); 72.47 (d, C(3’)); 71.06 (d, C(4’”)); 70.24 (t, C(6’)); 66.36 (d, C(5’)); 63.40 (t, C(5”)); 58.45 (d, C(2’)); 58.09 (q, MeO); 54.79 (d, C(2’”)); 53.02 (d, C(1)); 52.24 (d, C(3)); 43.38 (t, C(6’”)); 37.57 (t, CH_2); 36.54 (d, C(2)); 31.27 (t, CH_2) HR-MALDI-MS (*m/z*): 762.3754 (100, [*M* + H]$^+$, $C_{33}H_{56}N_5O_{15}^+$; calc. 762.3773).

4’,6’-O-*(4-Phenylbutylidene)paromomycin* (**33**). Application of the standard deacetylation protocol to 77 mg of **79** and FC gave 57 mg of **99** (95%). White solid. R_f ($CHCl_3$/AcOEt/MeOH 3:3.0.5) 0.51. Without further characterization, according to Staudinger protocol **A**, reaction of 32 mg of **99** and FC gave 22 mg of **33** (81%). White solid. R_f (MeOH/25% aq. NH_3 4:1) 0.25. IR (ATR): 3172*w*, 3024*w*, 2864*w*, 1622*w*, 1524*w*, 1434*w*, 1045*s*, 974*s*. ^{1}H-NMR (500 MHz, D_2O): δ 7.39–7.36 (m, 2 arom. H); 7.31–7.26 (m, 3 arom. H); 5.99 (d, *J* = 4.2, H–C(1’)); 5.44 (d, *J* = 2.2, H–C(1”)); 5.32 (d, *J* = 1.7, H–C(1’”)); 4.80–4.69 (m, Ph(CH_2)$_3$CH); 4.55 (dd, *J* = 6.8, 4.8, H–C(3”)); 4.47 (dd, *J* = 4.7, 2.2, H–C(2”)); 4.36 (ddd, *J* = 8.5, 3.9, 1.4, H–C(5’”)); 4.27 (t, *J* = 3.2, H–C(3’”)); 4.28–4.23 (m, H–C(4”)); 4.24 (dd, *J* = 10.3, 4.7, H_a–C(6’)); 4.16 (t, *J* = 9.9, H–C(3’)); 4.06 (t, *J* = 9.2, H–C(4’)); 4.01 (t, *J* = 9.0, H–C(5)); 3.93 (dd, *J* = 12.5, 3.0, H_a–C(5”)); 3.86–3.84 (m, H–C(4’”)); 3.79–3.73 (m, H–C(6), H–C(5’), H_b–C(5”)); 3.67 (t, *J* = 10.3, H_b–C(6’)); 3.64–3.60 (m, H–C(3), H–C(2’”)); 3.58 (t, *J* = 9.5, H–C(4)); 3.53 (dd, *J* = 10.5, 4.3, H–C(2’)); 3.45 (dd, *J* = 13.5, 7.0, H_a–C(6’”)); 3.41–3.36 (m, H–C(1), H_b–C(6’”)); 2.67 (t, *J* = 7.0, CH_2CH); 2.54 (dt, *J* = 12.6, 4.2, H_{eq}–C(2)); 1.94 (q, *J* = 12.6, H_{ax}–C(2)); 1.77–1.58 (m, 2CH_2). ^{13}C-NMR (126 MHz, D_2O): δ 131.29 (4d); 128.71 (d); 112.99 (d, C(1”)); 105.53 (d, Ph(CH_2)$_3$CH); 98.58 (d, C(1’)); 97.83 (d, C(1’”)); 87.36 (d, C(5)); 84.01 (d, C(4”)); 82.09 (d, C(4)); 78.28 (d, C(4’)); 77.55 (d, C(3”)); 75.90 (d, C(2”)); 75.13 (d, C(6)); 73.03 (d, C(5’”)); 70.34 (d, C(3’”)); 69.85 (d, C(4’”), t, C(6’)); 68.86 (d, C(3’)); 66.80 (d, C(5’)); 62.93 (d, C(5”)); 57.01 (d, C(2’)); 53.53 (d, C(2’”)); 52.55 (d, C(1)); 51.12 (d, C(3)); 43.05 (t, C(6’”)); 37.21 (t, CH_2); 35.35 (t, CH_2); 30.71 (t, C(2)); 27.90 (t, CH_2). HR-MALDI-MS (*m/z*): 747.3848 (39); 746.3814 (100, [*M* + H]$^+$, $C_{33}H_{56}N_5O_{14}^+$; calc. 746.3824).

4’,6’-O-*(5-Phenylpentylidene)paromomycin* (**34**). Application of the standard deacetylation protocol to 75 mg of **80** and FC gave 53 mg of **100** (91%). White solid. R_f ($CHCl_3$/AcOEt/MeOH

3:3.0.5) 0.51. Without further characterization, according to Staudinger protocol **A**, reaction of 30 mg of **100** and FC gave 24 mg of **34** (94%). White solid. R_f (MeOH/25% aq. NH_3 4:1) 0.25. IR (ATR): 3141*w*, 2864*w*, 1612*w*, 1527*w*, 1497*w*, 1452*w*, 1382*w*, 1043*s*. ^{1}H-NMR (500 MHz, D_2O): δ 7.38–7.35 (m, 2 arom. H); 7.31–7.24 (m, 3 arom. H); 5.80 (d, *J* = 4.1, H–C(1')); 5.30 (d, *J* = 2.2, H–C(1")); 5.30 (d, *J* = 1.8, H–C(1"')); 4.54 (dd, *J* = 6.9, 4.8, H–C(3")); 4.44 (dd, *J* = 4.8, 2.2, H–C(2")); 4.34 (ddd, *J* = 7.0, 3.9, 1.5, H–C(5"')); 4.25 (t, *J* = 3.1, H–C(3"')); 4.24–4.22 (m, H–C(4")); 4.18 (dd, *J* = 10.5, 4.9, H_a–C(6')); 4.04 (t, *J* = 9.9, H–C(3')); 3.92 (dd, *J* = 12.5, 3.0, H_a–C(5")); 3.89 (t, *J* = 9.1, H–C(5)); 3.86–3.81 (m, H–C(5'), H–C(4"')); 3.77–3.73 (m, H–C(4), H_b–C(5")); 3.67 (t, *J* = 10.5, H_b–C(6')); 3.66 (t, *J* = 9.2, H–C(6)); 3.59–3.57 (m, H–C(2"')); 3.55 (t, *J* = 9.5, H–C(4')); 3.46–3.40 (m, H–C(2'), H_a–C(6"')); 3.37 (dd, *J* = 13.6, 3.9, H_b–C(6"')); 3.32–3.23 (m, H–C(1), H–C(3)); 2.65 (t, *J* = 7.5, $PhCH_2$); 2.33 (dt, *J* = 12.7, 4.2, H_{eq}–C(2)); 1.73–1.57 (m, H–C(2), 2 CH_2) 1.45–1.38 (m, CH_2). $Ph(CH_2)_4CH$ hidden below solvent peak. ^{13}C-NMR (126 MHz, D_2O): δ 131.26 (4d); 128.55 (d); 112.75 (d, C(1")); 105.67 (d, CH_2CH); 99.14 (d, C(1')); 98.09 (d, C(1"')); 87.62 (d, C(5)); 83.86 (d, C(4")); 82.28 (d, C(4')); 81.68 (d, C(4)); 77.63 (d, C(3")); 75.95 (d, C(6), C(2")); 73.06 (d, C(5"')); 70.54 (d, C(3"')); 69.96 (2d, C(3'), C(4"'), t, C(6')); 66.59 (d, C(5')); 62.92 (t, C(5")); 57.36 (d, C(2')); 53.63 (d, C(2"')); 53.00 (d, C(3)); 51.41 (d, C(1)); 43.09 (t, C(6"')); 37.47 (t, CH_2); 35.70 (t, CH_2); 33.03 (2t, C(2), CH_2); 25.55 (t, *C*H_2). HR-MALDI-MS (*m/z*): 782.3774 (47, $[M + Na]^+$, $C_{34}H_{57}N_5NaO_{14}^+$; calc. 782.3800); 761.3984 (39); 760.3957 (100, $[M + H]^+$, $C_{34}H_{58}N_5O_{14}^+$; calc. 760.3975).

*(2S,3S,4R,5R,6R)-5-amino-2-(aminomethyl)-6-((2R,3S,4R,5S)-5-((1R,2R,3S,5R,6S)-3,5-diamino-2-((1R,2S)-2-amino-3-hydroxy-1-(trans-2-phenyl-1,3-dioxan-5-yloxy)propoxy)-6-hydroxycyclohexyloxy)-4-hydroxy-2-(hydroxymethyl)tetrahydrofuran-3-yloxy)tetrahydro-2H-pyran-3,4-diol (**36**).* Reduction of **119** according to Staudinger protocol **C**, followed by FC (MeOH/ aq NH_3 (4:1)) gave **36** (50 mg, 60%) R_f 0.23. IR (ATR): 3358*w*, 2918*w*, 1573*w*, 1463*w*, 1407*w*, 1131*s*, 1094*s,* 1027*w*, 923*m*. ^{1}H-NMR (400 MHz, D_2O): δ 7.53–7.46 (*m,* H_5–C_6)); 5.64 (s*,* PhC*H*); 5.35 (d*, J* = 1.9, H–C(1'')); 5.28 (d*, J* = 3.1, H–C(1')); 4.98 (d*, J* = 1.9, H–C(1''')); 4.51, 4.38 (m, H_a–C(4'), H_a–C(6')); 4.47 (dd, *J* = 7.0, 4.9, H–C(3'')); 4.47 (dd, *J* = 4.9, 1.9, H–C(2'')); 4.17–4.11 (m, H–C(4'')); 4.07 (dddd, *J* = 10.0, 10.0, 5.0, 5.0, H–C(5')); 4.02 (dd, *J* = 3.3, 3.3, H–C(3''')); 3.97–3.74 (m, H_a–

C(3'), H_b–C(3'), H_b–C(4'), H_b–C(6'), H_a–C(5''), H–C(5''')); 3.66–3.60 (m, H–C(5), H_b–C(5''), H–C(4''')); 3.36 (dd, *J* = 9.4, 9.4, H–C(4)); 3.28 (dd, *J* = 9.6, 9.6, H–C(6)); 3.08–3.03 (m, H–C(2'''), H_a–C(6''')); 3.08–3.03 (m, H–C(2')); 2.93 (dd, *J* = 13.5, 4.2, H_b–C(6''')); 2.80 (ddd, *J* = 12.4, 9.4, 4.2, H–C(3)); 2.75 (dd, *J* = 12.4, 4.2, H–C(1)); 2.03 (ddd, *J* = 13.0, 4.2, 4.2, H_{eq}–C(2)); 1.24 (ddd, J = 13.0, 12.4, 12.45, H_{ax}–C(2)). ^{13}C-NMR (100 MHz, D_2O): δ 139.30 (*s,* C(1) of Ph); 129.91 (*d,* C(4) of Ph); 129.12 (*d,* C(3)/C(5) of Ph); 127.40 (*d,* C(2)/C(6) of Ph); 111.72 (*d,* C(1'')); 105.32 (C(1')); 102.48 (*d,* Ph*C*H); 101.75 (*d,* C(1''')); 85.36 (C(5)); 83.80 (C(4'')); 82.55 (C(4)); 80.03 (C(6)); 78.27 (C(3'')); 76.78 (C(5''')); 76.18 C(2'')); 55.20 (C(2''')); 73.15 (C(3''')); 72.27, 71.86 (C(4'), C(6')); 71.17 (C(4''')); 70.01 (C(5')); 63.95(C(5'')); 63.30 (C(3')); 58.05 (C(2')); 53.03 (C(1)); 52.13 (C(3)); 43.60 (C(6''')); 37.64 (C(2)). HR-MALDI-MS (*m/z*): 706.35 [*M*+H]$^+$, 728.33 [*M*+Na]$^+$.

*(2S,3S,4R,5R,6R)-5-amino-2-(aminomethyl)-6-((2R,3S,4R,5S)-5-((1R,2R,3S,5R,6S)-3,5-diamino-2-((1R,2S)-2-amino-3-hydroxy-1-(cis-2-phenyl-1,3-dioxan-5-yloxy)propoxy)-6-hydroxycyclohexyloxy)-4-hydroxy-2-(hydroxymethyl)tetrahydrofuran-3-yloxy)tetrahydro-2H-pyran-3,4-diol (35)***.** Reduction of **120** according to Staudinger protocol **C**, followed by FC (MeOH/ aq NH_3 (4:1)) gave **35** (50 mg, 60%). R_f 0.25. IR (ATR): 3354*w*, 3287*w*, 2918*s*, 1591*w*, 1345*w*, 1125*w*, 1023*s*, 974*s,* 859*m*. ^{1}H-NMR (500 MHz, CD_3OD): δ 7.52–7.48 (m*,* 5 H–Ph); 5.30 (d, *J* = 1.8, H–C(1'')); 5.77 (s, PhC*H*); 5.21 (d*, J* = 3.3, H–C(1')); 4.92 (d, *J* = 1.8, H–C(1''')); 4.44–4.39, 4.25 (m, H_a–C(4'), H–C(5'), H_aC(6'), H_bC(6')); 4.31 (dd, *J* = 5.0, 5.0, 2.1, H–C(2'')); 4.10 (ddd, *J* = 5.0, 5.0, 3.0, H–C(4'')); 3.99 (dd, *J* = 5.0, 5.0, H–C(3''')); 3.92–3.80 (m, H_a–C(3'), H–C(3''), H_a–C(5''), H–C(5''')); 3.72–3.64 (m, H_b–C(3')); 3.61 (dd, *J* = 3.3, 1.8, H–C(4''')); 3.56 (dd, *J* = 9.2, 9.2, H–C(5)); 3.45 (dd, *J* = 9.3, 9.2, H–C(4)); 3.15 (dd, *J* = 9.6, 9.1, H–C(6)); 3.09 (ddd, *J* = 7.0, 5.2, 3.1, H–C(2')); 2.98 (dd, *J* = 13.1, 8.5, H_a–C(6''')); 2.89 (dd, *J* = 13.1, 4.3, H_b–C(6''')); 2.74–2.65 (dd, *J* = 12.4, 9.3, H–C(3)); 2.70–2.61 (ddd, *J* = 12.4, 9.1, 4.2, H–C(1)); 1.93 (ddd*, J* = 12.9, 4.2, 4.2, H_{eq}–C(2)); 1.01 (ddd, *J* = 12.9, 12.4, 12.4, H_{ax}–C(2)). ^{13}C-NMR (100 MHz, D_3O): δ 132.57, 131.48, 128.77 (C_6H_5), 111.52 (d*,* C(1'')); 102.88 (d, Ph*C*H); δ 111.52 (C(1'')); 104.21 (C(1')); 101.87 (C(1''')); 87.09 (C(5)); 83.96 (C(4'')); 83.36 (C(4)); 80.17 (C(6)); 78.48 (C(5')); 77.75 (C(3'')); 76.08 (C(2'')); 72.05 (C(5''')); 73.25 (C(3''')); 72.47, 71.53 (C(4'), C(6')); 71.16 (C(4''')); 64.30 (C(5'')); 63.30 (C(3')); 57.93 (*t,* C(2')); 51.12 (C(1)); 52.35 (C(3)); 60.96 (*d,* C(3)); 43.70 (C(6''')); 38.00 (C(2)). HR_MALDI_MS:

706.35 $[M+H]^+$, 728.33 $[M+Na]^+$. HR-ES-MS (*m/z*): 706.3485 (100, $[M + H]^+$, $C_{30}H_{52}N_5O_{14}^+$, calc. 706.3511).

4',6'-O-*Benzylideneparomamine* (**44**). Under N_2, a soln. of **45** (21 mg, 0.028 mmol), benzaldehyde dimethylacetal (17 µl, 0.011 mmol), and $TsOH.H_2O$ (5 mg, 0.026 mmol) in DMF (0.5ml) was kept at 25°. After 24 h, TLC showed that about half of the starting material had reacted. The mixture was heated to 65°. No change was observed after 1 h. Benzaldehyde dimethylacetal (17 µl, 0.011 mmol) and $TsOH.H_2O$ (5 mg, 0.026 mmol) were added. After 24 h, the starting material had disappeared (TLC). The soln. (pH 5–6) was neutralized with 1M NaOH, and extracted with AcOEt (3 x 8 ml) and H_2O (5 ml). The combined org. layers were washed with brine (15 ml), dried (MgSO4), filtered, and evaporated. FC (Hexane, then Hexane/AcOEt 9:1, then $CHCl_3$/AcOEt 1:1 → $CHCl_3$/AcOEt/MeOH 5:5:0.1) gave **58** (8 mg, 58%, white solid) and **45** (3 mg, 13%). **58:** R_f ($CHCl_3$/AcOEt/MeOH 3:3:0.5) 0.80. ^{1}H-NMR (300 MHz, CD_3OD): δ 7.52–7.47 (*m*, 2 arom. H); 7.37–7.33 (*m*, 3 arom. H); 5.65 (*d*, *J* = 3.8, H–C(1')); 5.60 (*s*, PhC*H*); 4.25–4.07 (*m*, 3 H); 3.77 (*t*, *J* = 9.8, 1 H); 3.59–3.23 (*m*, 6 H); 2.25 (*dt*, *J* = 13.0, 4.1, H_{eq}–C(2)); 1.42 (*q*, *J* = 12.3, H_{ax}–C(2)). Without further characteriztion, according to Staudinger protocol A, reaction of 8 mg **58** for 2 h. followed by FC (THF, THF/MeOH, MeOH, MeOH/25% aq. NH_3 49:1 → 47:3) gave **44** (6 mg, 89%). White solid. R_f (MeOH/25% aq. NH_3 9:1) 0.26. IR (ATR): 3033*w*, 1608*w*, 1528*w*, 1452*w*, 1382*w*, 1089*s*, 1050*s*, 996*s*. ^{1}H-NMR (300 MHz, D_2O): δ 7.41–7.37 (m, 2 arom. H); 7.34–7.31 (m, 3 arom. H); 5.72 (d, *J* = 4.1, H–C(1')); 5.64 (s, PhCH); 4.22 (dd, *J* = 9.9, 4.4, H_a–C(6')); 4.08 (t, *J* = 10.0, 1 H); 3.88 (dd, *J* = 9.7, 4.4, 1 H); 3.82–3.75 (m, 2 H); 3.67 (t, *J* = 9.4, 1 H); 3.58 (t, *J* = 9.2, 1 H); 3.49–3.39 (m, 3 H); 3.25–3.16 (m, 1 H); 2.40 (dt, *J* = 12.5, 4.3, H_{eq}–C(2)); 1.75 (q, *J* = 12.5, H_{ax}–C(2)). ^{13}C-NMR (175 MHz, D_2O): δ 129.93, 128.80 (2 Cs), 126.26, 101.77, 97.03, 80.03, 78.55, 75.27, 72.59, 67.73, 66.34, 63.86, 54.34, 49.88, 48.49, 28.37. HR-MALDI-MS (*m/z*): 413.2104 (18); 412.2072 (82, $[M + 2\,H]^+$, $C_{19}H_{30}N_3O_7^+$; calc. 412.2084).

2. Synthesis of 4'- and 6'-O-alkyl Derivatives.

2.1. Synthesis of Ethers 37 and 38.

*6,3',2'',5'',3''',4'''-Hexa-*O*-acetyl-1,3,2',2''',6'''-pentadeamino-1,3,2',2''',6'''-pentaazido-4'-*O*-benzyl paromomycin* (**106**). Under N_2, a soln. of **59** (1.8 g, 1.66 mmol) in dry CH_2Cl_2 (25 ml, dried over CaH_2) was cooled to –5 °C, treated with 2M $BH_3·Me_2S$ in THF (8.3 ml, 16.6 mmol) and 1M Bu_2BOTf in CH_2Cl_2 (0.83 ml, 0.83 mmol), and stirred for 5 h. Saturated aq. $NaHCO_3$ soln. was added, and the layers were separated. The org. layer was washed with brine, dried ($MgSO_4$), filtered, and evaporated. FC (AcOEt/cyclohexane 9:11) gave **106** (1.1 g, 61%). R_f (AcOEt/cyclohexane 1:1) 0.41. M. p. 82–86 °C. $[\alpha]_D^{25}$ = +98.2 (*c* = 0.13, MeOH). IR (ATR): 2940*w*, 2872*w*, 2100*s*, 1740*s*, 1492*w*, 1453*w*, 1430*w*, 1371*m*, 1215*s*, 1027*s*. ^{1}H-NMR (CD_3OD, 400 MHz): δ 7.52–7.26 (m, 5 arom. H); 4.69, 4.65 (2d, *J* = 11.5, PhCH_2); 5.81 (*d*, *J* = 3.9, H–C(1')); 5.39 (d, *J* = 1.8; H–C(1'')); 5.13 (d, *J* = 1.9, H–C(1''')); 3.94 (dd, *J* = 3.3, 3.3, H–C(3''')); 4.16–4.13 (m, H–C(4'')); 4.45 (dd, J = 6.6, 4.7, H–C(3'')); 4.29 (dd, *J* = 4.7, 1.8, H–C(2'')); 4.04–3.99 (m, H–C(5''')); 4.01–3.96 (ddd, *J* = 9.6, 2.6, 2.6, H–C(5')); 3.83 (m, H_a–C(6')); 3.73 (m, H_a–C(5'')); 3.72–3.62 (m, H–C(3'), H_b–C(6'), H–C(5), H–C(5''), H–C(4''')); 3.53–3.34 (m, H–C(1), H–C(3), H–C(4')); 3.49–3.44 (m, H_a–C(6''')); 3.46 (dd, *J* = 9.0, 9.0, H–C(4)); 3.46–3.43 (dd, *J* = 3.3, 1.5)); 3.45 (dd, *J* = 9.0, 9.0, H–C(6)); 3.39 (dd, J = 12.9, 4.8, H_b–C(6''')); 3.14 (dd, *J* = 10.4, 3.9, H–C(2')); 2.17, 2.16, 2.157, 2.11, 2.10, 2.00 (6s, 6*Me*C=O); 2.17–2.00 (hidden OH); 2.16 (ddd, *J* = 12.6, 4.5, 4.5, H_{eq}–C(2)); 1.35 (ddd, *J* = 12.6, 12.6, 12.3, H_{ax}–C(2)). ^{13}C-NMR (CD_3OD, 100 MHz):δ 170.97, 170.13, 170.00, 169.77, 169.73, 168.53 (6s, 6MeC=O); 137.86 (s); 128.51 (d, 2 C); 127.82 (d, 3 C); 108.57 (C(1'')); 99.34 (C(1')); 97.47 (C(1''')); 84.87 (C(5)); 83.09 (C(4'')); 79.16 (C(4')); 76.59, 76.03, 75.52, 75.15, 74.78 (C(3''), C(4), C(6), C(5''') C(2'')); 74.66 (t, PhCH_2); 72.92, 72.28 (C(3'), C(5')); 70.74 (C(3''')); 69.19 (C(4''')); 64.57 (C(6')); 62.90 (C(5'')); 61.43 (2C, C(1), C(3)); 61.21 (2C, C(2'); C(2''')); 52.07 (C(6''')); 32.76 (C(2)); 20.88 (q, 6 *Me*C=O)., HR-ESI-MS (*m/z*): 1110.3489 (100, $[M + Na]^+$, $C_{42}H_{53}N_{15}NaO_{20}^+$; calc. 1110.3469). Anal. calc. for $C_{42}H_{53}N_{15}O_{20}$ (1087.97): C 46.37, H 4.91, N 19.31, O 29.41; found: C 46.27, H 4.82, N 19.02, O 29.38.

1,3,2',2''',6'''-Pentadeamino-1,3,2',2''',6'''-pentaazido-4'-O-benzyl paromomycin (**107**). Under N_2, a soln. of **106** (100 mg, 0.09 mmol) in 0.02N MeONa in MeOH (2 ml) was stirred at 26 °C for 12 h, and neutralized with *Amberlite–IR 120* (H^+ form). Filtration, evaporation and FC ($CHCl_3$/AcOEt/MeOH 10:17.5:2) gave **107** (65 mg, 86%). White solid. R_f ($CHCl_3$/AcOEt/MeOH

4:9:1) 0.45. M.p. 98 °C. $[\alpha]_D^{25}$ = +106.1 (*c* = 0.14, MeOH). IR (KBr): 3418*s*, 2928*m*, 2107*s*, 1633*m*, 1497*w*, 1454*m*, 1384*m*, 1332*m*, 1261*s*, 1114*m*, 1068*m*, 1029*s*, 939*w*, 749*w*. ^{1}H-NMR ($CDCl_3$, 300 MHz): δ 7.39–7.22 (*m*, 5 arom. H); 4.97 (*d*, *J* = 10.8), 4.68 (*d*, *J* = 11.1) (PhC*H*$_2$); 6.13 (*d*, *J* = 3.9, H–C(1')); 5.65 (d, *J* = 5.7; H–C(1'')); 4.89 (d, *J* = 1.5, H–C(1''')); 4.09 (dd, *J* = 9.8, 9.3, H–C(3')); 4.35–4.26 (m, H_a–C(6')); 4.35–4.23 (m, H–C(3''), H–C(4'')); 4.13–4.03 (m, H–C(5')); 3.95–3.90 (m, H–C(2'')); 3.83–3.53 (m, Hb–C(6'), H–C(5), H–C(6), H_a–C(5''), H_b–C(5''), H–C(3'''), H–C(5''')); 3.50–3.35 (m, H–C(1), H–C(3), H_a–C(6''')); 3.45 (dd, J = 9.3, 9.3, H–C(4')); 3.40 (m, H–C(2''')); 3.27 (dd, *J* = 9.3, 9.3, H–C(4)); 3.07 (m, H–C(4''')); 3.02 (dd, *J* = 9.8, 3.9, H–C(2')), 2.23 (ddd, *J* = 13.5, 4.2, 4.2, H_{eq}–C(2)); 1.39 (ddd, *J* = 13.5, 12.9, 12.9, H_{ax}–C(2)). ^{13}C-NMR ($CDCl_3$, 75 MHz): δ 139.50 (s); 128.82 (d, 2 C); 128.55 (d, 2 C); 128.18 (d); 105.98 (C(1'')); 98.54 (C(1''')); 96.30 (C(1')); 83.93, 82.49, 82.25, 82.17, 81.70 (C(5), C(4''), (C4'), (C(4), (C(6)); 76.70 (t, Ph*C*H$_2$); 75.57 (2C), 75.18 (C(3'), C(3'') C(4)); 74.43 (C(5''')); 71.98 (C(3''')); 71.05 (C(4''')); 70.09 (C(5'')); 69.02 (C(6')); 62.95, 62.76 (C(2'), C(5')); 60.32 (C(3)); 59.90 (C(1)); 57.38 (C(2''')); 51.18 (C(6''')); 32.54 (C(2)). HR-ESI-MS (*m/z*): 858.2835 (100, $[M + Na]^+$, $C_{30}H_{41}N_{15}NaO_{14}^+$; calc. 858.2855). Anal. calc. for $C_{30}H_{41}N_{15}O_{14}$.0.3AcOEt (862.17): C 43.46, H 5.07, N 24.37; found: C 43.13, H 5.03, N 24.47.

4'-O-Benzyl paromomycin (**37**). A soln. of **107** (30 mg, 0.04 mmol) in THF (3 ml) was treated with 0.1M aq. NaOH (1 ml) and 1M PMe_3 in THF (0.22 ml, 0.22 mmol) and stirred at 50 °C for 2 h. Evaporation and FC (MeOH/25% aq. NH_3 4:3) gave **37** (20 mg, 79%). White solid. R_f ($CHCl_3$/MeOH/25% aq. NH_3 1:3:4) 0.55. $[\alpha]_D^{25}$ = +36.3 (*c* = 0.11, H_2O). IR (KBr): 3418*s*, 2925*m*, 2852*w*, 1631*m*, 1537*w*, 1452*w*, 1397*w*, 1384*m*, 1298*w*, 1123*s*, 1051*s*, 947*w*. ^{1}H-NMR (D_2O, 400 MHz): δ 7.43–7.37 (*m*, 5 arom. H); 7.43–7.37 (*m*, 5 arom. H); 4.85 (*d*, *J* = 10.7), 5.48 (d, *J* = 3.8, H–C(1')); 5.34 (d, *J* = 2.5; H–C(1'')); 5.11 (d, *J* = 1.6, H–C(1''')); 4.85 (d, *J* = 10.7), 4.70 (d, *J* = 10.8) (PhC*H*$_2$)); 4.47 (dd, J = 6.5, 5.0, H–C(3'')); 4.31 (dd, *J* = 5.0, 2.5, H–C(2'')); 4.14 (m, H–C(4'')); 4.23–4.20 (m, H–C(5''')); 4.01 (dd, *J* = 3.2, 3.2, H–C(3''')); 3.86–3.80 (m, H–C(5'), H_a–C(6')); 3.87 (dd, *J* = 12.4, 3.2, H_a–C(5'')); 3.84 (dd, *J* = 10.5, 8.8, H–C(3')); 3.76–3.67 (m, H_b–C(5'')); 3.75–3.73 (m, H–C(4''')); 3.72 (dd, *J* = 9.3, 9.3, H–C(5)); 3.54 (dd, *J* = 9.3, 9.3, H–C(4)); 3.48 (dd, *J* = 9.3, 9.3,

H–C(6)); 3.48 (dd, 8.8, 9.2, H–C(4’)); 3.34 (dd, J = 13.6, 7.3; H_a–C(6’’’)); 3.28 (dd, J = 13.6, 4.0, H_b–C(6’’’)); 3.26 (dd, J = 3.2, 1.6, H–C(2’’’)); 3.07–3.00 (ddd, J = 12.1, 9.3, 4.1, H–C(1)); 3.04 (dd, J = 10.5, 3.8, H–C(2’)); 2.99 (ddd, J = 12.1, 9.3, 4.1, H–C(3)); 2.11 (ddd, J = 12.9, 4.5, 4.5, H_{eq}–C(2)); 1.41 (ddd, J = 12.9, 12.1, 12.1, H_{ax}–C(2)). ^{13}C-NMR ($CDCl_3$, 100 MHz): δ 139.69 (s); 131.56 (d, 2 C); 131.48 (d, 2 C); 131.31 (d); 111.86 (C(1’’)); 105.92 (C(1’’)); 100.52 (C(1’)); 100.12 (C(1’’’)); 86.92 (C(5)); 84.53 (C(4)); 83.93 (C(4’’)); 80.31 (C(4’)); 77.97 (C(3’’)); 77.19 (C(6)); 76.70 (t, Ph*C*H_2)); 76.00 (C(2’’)); 75.01 (C(3’)); 74.44 (C(5’)); 73.57 (C(5’’’)); 71.88 (C(3’’’)); 70.74 (C(4’’’)); 63.14 (C(5’’)); 62.93 (C(6’)); 57.67 (C(2’)); 54.38 (C(2’’’)); 53.05 (C(1)); 52.25 (C(3)); 43.27 (C(6’’’)); 35.23 (C(2)). HR-ESI-MS (*m/z*): δ 728.3269 (100, $[M + Na]^+$, $C_{30}H_{51}N_5NaO_{14}^+$; calc. 728.3330); 706.3493 (100, $[M + H]^+$, $C_{30}H_{52}N_5O_{14}^+$; calc. 706.3511).

*1,3,2’,2’’’,6’’’-Pentadeamino-1,3,2’,2’’’,6’’’-pentaazido-4’,6’-*O*-benzylidene-6,3’,2’’,5’’,3’’’,4’’’-hexakis-*O*-(4-methoxybenzyl) paromomycin* (**108**). Under Ar, a soln. of **46** (810 mg, 0.97 mmol) in THF (20 ml) was treated with NaH (637mg, 50–60% suspension in oil, *ca* 13.3 mmol), *p*-MeOBnCl (0.90 ml, 6.66 mmol), and tetrabutylammonium iodide (110 mg), stirred at 0 °C for 4 h and at 26 °C for 20 h, cooled to 0 °C, and diluted portionwise with H_2O. After evaporation, the aq. layer was extracted with AcOEt (3 x 20 ml). The combined org. layers were washed with brine, dried ($MgSO_4$), filtered, and evaporated. FC (AcOEt/cyclohexane 1:4) gave **108** (722 mg, 48%). White solid. R_f (AcOEt/cyclohexane 1:1) 0.46. M.p. 54.6–56.4 °C (softens at 46 °C). $[\alpha]_D^{25}$ = +75.8 (*c* = 0.40, $CHCl_3$). IR ($CHCl_3$): 3021*w*, 3006*w*, 2937*w*, 2839*w*, 2867*w*, 2106*s*, 1612*m*, 1586*w*, 1514*s*, 1465*w*, 1442*w*, 1368*w*, 1302*w*, 1250*s*, 1174*m*, 1091*m*, 1035*m*, 847*w*, 823*w*. ^{1}H-NMR ($CDCl_3$, 300 MHz): δ 7.51–7.48 (m), 7.40–7.37 (m), 7.32–7.20 (m), 7.15–7.06 (m), 6.88–6.82 (m), 6.66 (d, J = 8.4) (29 arom. H); 6.12 (d, J = 3.9, H–C(1’)); 5.63 (d, J = 5.7; H–C(1’’)); 5.52 (s, PhC*H*); 4.90 (*d*, J = 9.3), 4.82 (d, J = 10.8), 4.70 (d, J = 10.8), 4.63 (d, J = 10.2), 4.56 (d, J = 12.0), 4.50 (d, J = 11.7), 4.44 (d, J = 11.4), 4.37 (d, J = 11.4), 4.30 (d, J = 12.3), 4.29 (d, J = 11.7), 4.19 (d, J = 12.0, 2 H) (6ArCH_2); 4.87 (d, J = 1.5, H–C(1’’’)); 3.94–3.86 (m, H–C(3’), H–C(3’’)); 4.24 (dd, J = 5.7, 2.1, H–C(2’’)); 4.17 (dd, J = 3.9, 3.9, H–C(3’’’)); 4.09 (ddd, J = 9.9, 9.9, 4.8, H–C(4’’)); 3.81, 3.80, 3.786, 3.78, 3.77, 3.71 (6*s,* 6 MeO); 3.66–3.35 (m, H–C(4’), H–C(5’), H_a–C(6’), H_b–C(6’), H–C(1), H–C(3), H–C(5), H–C(6), H_a–C(5’’), H_b–C(5’’), H–C(5’’’)); 3.60 (dd, J = 12.9, 4.2, H_a–C(6’’’)); 3.39 (dd, J = 3.9, 3.9, H–

C(2’’’)); 3.22 (dd, J = 9.3, 9.3, H–C(4)); 3.07 (m, H–C(4’’’)); 2.85 ((dd, J = 12.9, 3.9, H_b–C(6’’’)); 2.20 (ddd, J = 12.9, 4.8, 4.8, H_{eq}–C(2)); 1.31 (ddd, J = 12.9, 12.9, 12.9, H_{ax}–C(2)). ^{13}C-NMR ($CDCl_3$, 75 MHz): δ 159.46, 159.34, 159.07 (2 C), 158.93, 158.84 (5*s*, 6 arom. C); 137.31 (*s*, 1 arom. C); 130.35–128.13 (several *s* and *d*, 6 arom. C and 15 arom. CH); 113.92, 113.74, 113.65, 113.59 (4*d*, 12 arom. CH); 105.92 (C(1’’)); 101.32 (Ph*C*H); 98.63 (C(1’’’)); 95.90 (C(1’)); 83.91, 82.11, 81.78 (C(5), (C(4’’), (C(6); 82.23 (C(2’’)); 79.47 (C(3’)); 75.62, 74.77 (C(3’’), (C(4)); 74.75, 74.57, 72.93 (2 C), 72.43, 71.22 (5t, 6ArCH_2); 74.321 (C(5’’’)); 72.50 (C(5’)); 71.99 (C(3’’’)); 71.04 (C(4’’’)); 70.32 (C(4’)); 70.23 (C(5’’)); 69.88 (C(6’)); 62.59 (C(2’)); 60.40 (C(3)); 60.24 (C(1)); 57.42 (C(2’’’)); 55.26 (q, 6MeO); 51.11 (C(6’’’)); 32.69 (C(2)). HR-ESI-MS (*m/z*): (100, $[M + Na]^+$, $C_{78}H_{87}N_{15}NaO_{20}^+$; calc. 1576.6149). Anal. calc. for $C_{78}H_{87}N_{15}O_{20}$ (1554.61): C 60.26, H 5.64, N 13.51; found: C 60.23, H 5.81, N 13.31.

*1,3,2’,2’’’,6’’’-Pentadeamino-1,3,2’,2’’’,6’’’-pentaazido-6’-*O*-benzyl-6,3’,2’’,5’’,3’’’,4’’’-hexakis-*O*-(4-methoxybenzyl) paromomycin* (**109**). Under N_2, a soln. of **108** (677 mg, 0.44 mmol) in THF (15 ml) was treated with 4 Å molecular sieves, stirred for 1 h, cooled to 0 °C, treated with $NaCNBH_3$ (443 mg, 7.05 mmol) at 0 °C, and then dropwise within 1 h with 0.7M HCl in Et_2O (14 ml, 9.80 mmol) (methyl orange was added as indicator) and stirred for 4 h. After neutralization with sat. $NaHCO_3$ soln. and evaporation, the aq. layer was extracted with AcOEt (3 x 25 ml). The combined org. layers were washed with brine, dried ($MgSO_4$), filtered, and evaporated. FC (AcOEt/cyclohexane 3/7) gave **109** (219 mg, 33%). White solid. R_f (AcOEt/cyclohexane 1:1) 0.42. M.p. 52–55 °C. $[\alpha]_D^{25}$ = +66.9 (c = 0.37, $CHCl_3$). IR ($CHCl_3$): 3673*w*, 3020*s*, 2937*w*, 2839*w*, 2106*s*, 1612*m*, 1583*w*, 1514*m*, 1465*w*, 1438*w*, 1366*w*, 1302*w*, 1250*m*, 1174*w*, 1118*w*, 1072*w*, 1035*m*, 901*w*. ^{1}H-NMR ($CDCl_3$, 300 MHz): δ 7.35–7.26 (m), 7.19 (d, J = 8.7), 7.14 (d, J = 6.6), 7.11 (d, J = 6.6), 7.08 (d, J = 9.0), 6.90–6.83 (m), 6.66 (d, J = 8.7) (29 arom. H); 5.83 (d, J = 3.6, H–C(1’)); 5.38 (d, J = 2.1; H–C(1’’)); 5.12 (d, J = 1.8, H–C(1’’’)); 4.90 (d, J = 9.9), 4.84 (d, J = 11.4), 4.67 (d, J = 11.4), 4.65 (d, J = 12.0), 4.58 (d, J = 9.9), 4.57 (d, J = 12.0, 2 H), 4.51 (d, J = 11.4), 4.44 (d, J = 11.4, 2 H), 4.41 (d, J = 10.2), 4.34 (d, J = 11.7), 4.29 (d, J = 11.7), 4.19 (d, J = 12.3) (6ArCH_2, PhCH_2); 4.43 (dd J = 6.6, 4.4, H–C(3’’)); 4.30 (dd, J = 4.4, 2.1, H–C(2’’)); 4.13–4.05 (ddd, J = 6.6, 5.4, 2.4, H–C(4’’)); 4.00 (ddd, J = 8.4, 4.8, 1.8, H–C(5’’’)); 3.93 (dd, J = 6.4, 4.4, H–C(3’’’)); 3.91 (m, H–C(3’)); 3.83 (dd, J = 11.7, 3.0, H_a–

C(6’)); 3.81, 3.80, 3.796, 3.78, 3.72, 3.70 (6s, 6 MeO); 3.79 (dd, J = 11.6, 2.4, H_a–C(5’’)); 3.73 (dd, J = 11.6, 5.4, H_b–C(5’’)); 3.68–3.50 (m, H_b–C(6’)); 3.68–3.36 (m, H–C(5’), H–C(5), H–C(6), H–C(4’’’)); 3.63 (dd, J = 8.8, 8.7, H–C(4)); 3.52–3.47 (ddd, J = 8.8, 8.7, 4.2, H–C(3)); 3.50–3.47 (m, H–C(1)); 3.45–3.41 (m, H–C(4’), H–C(2’’’)); 3.45–3.41 (dd, J = 13.1, 8.4, H_a–C(6’’’)); 3.37 (dd, J = 13.1, 4.8, H_b–C(6’’’)); 3.09 (dd, J = 10.5, 3.6, H–C(2’)); 2.40 (d, J = 2.7, OH); 2.15 (ddd, J = 12.6, 4.2, 4.2, H_{eq}–C(2)); 1.27 (ddd, J = 12.6, 12.2, 12.2, H_{ax}–C(2)). ^{13}C-NMR ($CDCl_3$, 75 MHz): δ 159.47, 159.35, 159.27, 159.08, 158.92, 158.89 (6*s*, 6 arom. C); 137.96 (*s*); 130.27–127.44 (several *s* and *d*, 6 arom. C and 15 arom. CH); 113.93, 113.75, 113.65, 113.62, 113.57 (5*d*, 12 arom. C); 159.47, 159.35, 159.27, 159.08, 158.92, 158.89 (6s, 6 arom. C); 137.96 (s); 130.27–127.44 (several s and d, 6 arom. C and 15 arom. CH); 113.93, 113.75, 113.65, 113.62, 113.57 (5d, 12 arom. C); 109.00 (C(1’’)); 97.63 (C(1’)); 99.36 (C(1’’’)); 84.91 (C(5)); 75.57 (C(6) or C(4)); 76.18 (C(4) or C(6)); 83.08 (C(4’’)); 76.85 (C(2’) or C(3’’) or C(3) or C(2’’’)); 74.70 (2 C), 73.35, 73.03, 72.91, 71.99, 71.26 (6t, Ar*C*H_2, Ph*C*H_2); 72.62, 71.98, 71.71, (C(3’), C(4’), C(5’)); 76.18, 75.18, 74.75, 74.03, 61.46 (C1), C(4), C(6), C(2’’), C(5’’’)), 10.77 C(3’’’)); 70.36 C(6’)); 69.20 (C4’’’)); 63.60 (C(5’’)); 61.46 (C(2’) or C(2’’’) or C(3), or C(3’’)); 61.22 (C(2’) or C(2’’’) or C(3) or C(3’’)); 55.30 (q, 3 MeO), 55.26 (q, 2 MeO), 55.21 (q, MeO)); 52.06 (C(6’’’)); 32.76 (C2)). HR-ESI-MS (*m/z*): 1578.6274 (100, $[M + Na]^+$, $C_{78}H_{89}N_{15}NaO_{20}^+$; calc. 1578.6306); 1529.6556 (84, $[M - N_2 + 2H]^+$, $C_{78}H_{91}N_{13}NaO_{20}^+$; calc. 1529.6503); 1528.6536 (94, $[M - N_2 + H]^+$, $C_{78}H_{90}N_{13}NaO_{20}^+$; calc. 1528.6425). Anal. calc. for $C_{78}H_{89}N_{15}O_{20}$ (1556.65): C 60.18, H 5.76, N 13.50; found: C 60.05, H 5.64, N 13.47.

*1,3,2’,2’’’,6’’’-Pentadeamino-1,3,2’,2’’’,6’’’-pentaazido-6’-*O*-benzyl paromomycin* (**110**). Under N_2, a soln. of **109** (107 mg, 0.07 mmol) in CH_2Cl_2/H_2O/i-PrOH 20:1:1 (7 ml) was treated with DDQ (103 mg, 0.45 mmol) at 0 °C and stirred at 0° for 2 h and at 26 °C for 24 h. The color of the mixture changed from colorless to green and then to light orange. After neutralization with sat. $NaHCO_3$ soln., the aq. layer was extracted with AcOEt (3 x 20 ml). The combined org. layers were washed with brine, dried ($MgSO_4$), filtered, and evaporated. FC ($CHCl_3$/AcOEt/MeOH 10:17.5:2)) gave **110** (34 mg, 59%). White solid. R_f ($CHCl_3$/AcOEt/MeOH 4:9:1) 0.68. M.p. 89.1–92.9 °C. $[\alpha]_D^{25}$ = +97.2 (*c* = 0.12, MeOH). IR (KBr): 3434*s*, 2929*w*, 2107*s*, 1635*w*, 1500*w*, 1401*w*, 1384*m*, 1331*w*, 1262*m*, 1140*m*, 1077*m*, 1036*m*, 743*w*. ^{1}H-NMR ($CDCl_3$, 300 MHz): δ 7.39–7.26 (*m*, 5 arom. H); 6.13 (d, J = 3.6, H–

C(1')); 5.57 (d, J = 2.6), H–C(1'')); 4.92 (J = 1.9, H–C(1''')); 4.25 (dd, J = 5.1, 2.3, H–C(3'')); 3.94 (dd, J = 6.0, 5.1, H–C(2'')); 4.28 (ddd, J = 4.9, 3.3, 2.3, H–C(4'')); 3.94 (dd, J = 8.9, 8.9, H–C(5)); 3.88–3.85 (m, H–C(5')); 3.86 (dd, J = 10.3, 8.7, H–C(3')); 3.81–3.75 (m, H_a–C(6'), H_a–C(5'), H–C(5''')); 3.74 (dd, J = 2.9, 2.9, H–C(3''')); 3.66–3.64 (m, H_b–C(6')); 3.64 (dd, J = 13.0, 8.6, H_a–C(6''')); 3.61 (dd, J = 9.7, 8.9, H–C(6)); 3.54 (dd, J = 10.4, 3.3, H_b–C(5'')); 3.49–3.38 (ddd, J = 12.7, 10.3, 4.6, H–C(3), m, H–C(4'), m, H–C(2''')); 3.49–3.38 (ddd, J = 12.7, 9.7, 4.6, H–C(1)); 3.27 (dd, J = 10.3, 8.9, H–C(4)); 3.08–3.07 (m, H–C(4''')); 2.86 (dd, 6.0, 5.1, H–C(2')); 2.84 (dd, J = 12.9, 4.5, H_b–C(6''')); 2.22 (ddd, J = 12.9, 4.6, 4.6, H_{eq}–C(2)); 1.37 (ddd, J = 12.9, 12.7, 12.7, H_{ax}–C(2)). ^{13}C-NMR ($CDCl_3$, 75 MHz): δ 139.25 (*s*); 128.92 (*d*, 2 C), 128.32 (*d*, 2 C), 128.21 (*d*); 106.05 (C(1'')); 98.73 (C(1''')); 95-96 (C(1')); 84.05 (C(4)); 82.30 (C(5) *or* C(4'')); 82.28 (C(4'') *or* C(5)); 81.87 (C(2'')); 79.49 (C(3')); 76.85 (*t*, Ph*C*H_2); 75.64 (C(3'')); 74.94 (C(6) or C(5''')); 74.47 (C(5''') *or* C(6)); 72.59 (C(3''')); 71.65 (C(5')); 71.21 (C(4''')); 70.51 (C(4')); 70.11 (C(5'')); 62.69 (C(2')); 62.24 (C(6')); 60.42 (C(1) *or* C(3)); 60.32 (C(3) or C(1)); 57.41 (C(2''')); 51.23 (C(6''')); 32.61 (C(2)). HR-ESI-MS (*m/z*): 858.2836 (100, $[M + Na]^+$, $C_{30}H_{41}N_{15}NaO_{14}^+$; calc. 858.2855). Anal. calc. for $C_{30}H_{41}N_{15}O_{14}.CH_3OH$ (867.78): C 42.91, H 5.23, N 24.21; found: C 42.73, H 4.96, N 24.25.

6'-O-Benzyl paromomycin (**38**) [3]). A soln. of **110** (25 mg, 0.03 mmol) in THF (3 ml) was treated with 0.1M aq. NaOH (1 ml) and 1M PMe_3 in THF (0.22 ml, 0.22 mmol) and heated to 50 °C for 2 h. Evaporation and FC (MeOH/25% aq. NH_3 4:3) gave **38** (18 mg, 85%). White solid. R_f ($CHCl_3$/MeOH/25% aq. NH_3 1:4:3) 0.56. M.p. 164.7 °C (dec.). $[\alpha]_D^{25}$ = +28.1 (*c* = 0.08, H_2O). IR (KBr): 3420*s*, 2925*m*, 1633*m*, 1595*m*, 1491*w*, 1454*w*, 1397*w*, 1384*m*, 1252*w*, 1151*m*, 1092*m*, 1026*s*, 1051*s*, 741*w*. ^{1}H-NMR (D_2O, 400 MHz): δ 7.46–7.37 (*m*, 5 arom. H); 5.33 (d, J = 2.7, H–C(1'')); 5.30 (d, J = 3.5; H–C(1')); 5.13 (d, J = 2.7, H–C(1''')); 4.60 (*s*, PhC*H_2*); 4.42 (dd, J = 6.2, 5.0, H–C(3'')); 4.26 (dd, J = 5.0, 2.7, H–C(2'')); 4.14 (ddd, J = 6.2, 5.1, 1.8, H–C(4'')); 4.05–4.01 (m, H–C(5''')); 3.94–3.88 (ddd, J = 9.8, 5.1, 3.2, H–C(5')); 4.01 (dd, J = 3.3, 3.3, H–C(3''')); 3.85 (dd, J = 12.4, 3.2, H_a–C(6')); 3.84 (dd, J = 11.1, 1.8, H_a–C(5'')); 3.71 (dd, J = 11.1, 5.1, H_b–C(5'')); 3.71 (dd, J = 12.4, 5.1, H_b–C(6')); 3.65 (m, H–C(4''')); 3.63 (dd, J = 9.9, 9.2, H–C(5)); 3.58 (dd, J = 10.3, 9.2, H–C(3'));

[3]) The free base was converted to the acetate by dissolving it in 50% aq. AcOH followed by evaporation (rotavap) and lyophilization.

3.40 (dd, J = 9.2, 9.2, H–C(4)); 3.39 (dd, J = 9.9, 9.2, H–C(6)); 3.27 (dd, 9.8, 9.2, H–C(4’)); 3.14 (dd, J = 13.5, 7.9; H_a–C(6’’’)); 3.13 (dd, J = 10.5, 3.7, H–C(2’)); 3.05 (dd, J = 13.5, 3.9, H_b–C(6’’’)); 3.04 (dd, J = 3.3, 1.9, H–C(2’’’)); 2.86–2.73 (m, H–C(1), H–C(3)); 2.76 (dd, J = 10.3, 3.5, H–C(2’)); 1.91 (ddd, J = 13.1, 4.3, 4.3, H_{eq}–C(2)); 1.16 (ddd, J = 13.1, 12.4, 12.4, H_{ax}–C(2)). ^{13}C-NMR ($CDCl_3$, 100 MHz): δ 139.97 (s); 131.40 (d, 2 C), 131.12 (d, 2 C), 131.02 (d); 111.25 (C(1’’)); 101.97 (C(1’)); 101.66 (C(1’’’)); 86.65 (C(5)); 86.16 (C(4)); 84.03 (C(4’’)); 79.47 (C(6)); 78.33 (C(3’’)); 75.99 (C(2’’)); 75.94 (t, Ph*C*H_2); 75.79 (C(3’) or C(5’’’)); 75.63 (C(5’’’) or C(3)); 74.52 (C(5’)); 72.98 (C(4’)); 72.75 (C(3’’’)); 71.66 (C(6’)); 71.16 (C(4’’’)); 63.60 (C(5’’)); 57.99 (C(2’)); 55.03 (C(2’’’)); 52.91 (C(1)); 52.74 (C(3)); 43.49 (C(6’’’)); 37.51 (C(2)). HR-ESI-MS (*m/z*): 728.3269 (100, [*M* + Na]$^+$, $C_{30}H_{51}N_5NaO_{14}^+$; calc. 728.3330); 706.3493 (100, [*M* + H]$^+$, $C_{30}H_{52}N_5O_{14}^+$; calc. 706.3511). Anal. calc. for $C_{30}H_{51}N_5O_{14}$.6 AcOH.3 H_2O (1120.11)$^{\pm}$: C 45.04, H 7.29, N 6.25; found: C 44.85, H 6.72, N 6.68.

2.2. Synthesis of Ethers 39-43.

1,3,2’,2’’’,6’’’-Pentaazido-1,3,2’,2’’’,6’’’-Pentadeamino-6,3’,2’’,5’’,3’’’,4’’’-hexakis-O-(4-methoxybenzyl)paromomycin ***(111)***. Under N_2, a soln. of **108** (400 mg, 0.256 mmol) in MeOH/CH_2Cl_2 10:1 (5.5 ml) was treated with TsOH·H_2O (49 mg, 0.3 mmol), stirred at 25° for 3 h, and diluted with 1N NaOH. After separation of phases, the aq. layer was extracted with CH_2Cl_2 (3 x 5 ml). The combined org. layers were dried ($MgSO_4$) and evaporated. FC (AcOEt/cyclohexane 2:3) gave **111** (296 mg, 79%). White solid. M.p. 54–60°. R_f (AcOEt/cyclohexane 1:1) 0.37. $[\alpha]_D^{25}$ = +81.9 (*c* = 0.675, $CHCl_3$). IR (ATR): 3458*w*, 2933*w*, 2099*s*, 1733*w*, 1611*m*, 1586*w*, 1512*s*, 1463*w*, 1367*w*, 1302*m*, 1244*s*, 1173*m*, 1110*m*, 1066*m*, 1028*s*. ^{1}H-NMR (600 MHz): δ 7.32–7.29 (*m*, 4 arom. H); 7.19–7.18 (*m*, 2 arom. H); 7.14–7.09 (*m*, 6 arom. H); 6.89–6.83 (*m*, 10 arom. H); 6.69–6.68 (*m*, 2 arom. H); 6.22 (d, J = 3.7, H–C(1’)); 5.66 (d, J = 6.1), H–C(1’’)); 4.92 (d, J = 10.0), 4.87 (d, J = 11.2), 4.62 (d, J = 9.9), 4.58 (d, J = 11.2), 4.55 (d, J = 11.9), 4.51 (d, J = 11.4), 4.43 (d, J = 11.4), 4.41 (d, J = 11.8), 4.40 (s), 4.39 (d, J = 9.5), 4.30 (d, J = 11.7), 4.19 (d, J = 11.9) (12 Ar*CH*); 4.89 (J = 1.9, H–C(1’’’)); 4.28-4.26 (ddd, J = 4.9, 3.3, 2.3, H–C(4’’)); 4.20–4.14 (m, H–C(3’’)); 3.96 (dd, J = 8.9, 8.9,

H–C(5)); 3.94 (dd, J = 6.0, 5.1, H–C(2'')); 3.91 (dd, J = 10.2, 8.7, H–C(3')); 3.84 (dd, J = 9.7, 8.9, H–C(6)); 3.81–3.69 (m, H–C(5), H_a–C(5''), H–C(5''')); 3.81, 3.80, 3.79, 3.78, 3.77, 3.72 (6*s*, 6 MeO); 3.68–3.67 (m, H–C(3''')); 3.59 (dd, J = 12.9, 8.4, H_a–C(6''')); 3.54– 3.51 (m, H–C(4)); 3.50 (ddd, J = 12.6, 9.7, 4.5, H–C(1)); 3.46–3.30 (m, H–C(2'''), H–C(3), H_a–C(6'), 3.53 (dd, J = 10.4, 3.1, H_b–C(5'')); 3.27 (dd, J = 9.3, 8.7, H–C(4')); 3.07 (m, H–C(4''')); 3 .00 (dd, J = 10.3, 3.7, H_b–C(6')); 2.84 (dd, 10.2, 3.7, H–C(2')); 2.81 (dd, J = 12.9, 4.5, H_b–C(6''')); 2.25 (ddd, J = 13.0, 4.6, 4.5, H_{eq}–C(2)); 1.39 (ddd, J = 13.0, 12.9, 12.6, H_{ax}–C(2)). ^{13}C-NMR (150 MHz, $CDCl_3$): δ 159.70, 159.59, 159.58, 159.32, 159.18, 159.16 (6*s*, 6 arom. C); 130.39–128.79 (several *s* and *d*, 6 arom. C and 12 arom. CH); 114.15, 114.07, 113.89, 113.76, 113.72 (5*d*, 12 arom. CH); 106.0 (C(1'')); 98.72 (C(1''')); 95.70 (C(1')); 84.09 (C(4)); 82.63 (C(5)); 82.23 (C(4'')); 81.91 (C(2'')); 79.75 (C(3')); 75.74 (C(3'')); 74.82, 74.61, 72.93, 72.05, 71.32 (5*t*, 6 Ar*C*H_2); 74.56 (C(5''') *or* C(6)); 74.36 (C(6) *or* C(5''')); 72.61 (C(3''')); 72.37 (C(5')); 71.16 (C(4''')); 70.85 (C(4')); 70.34 (C(5'')); 64.44 (C(6')); 62.80 (C(2')); 60.51(C(1) *or* C(3)); 60.32 (C(3) *or* (C(1)); 57.39 (C(2''')); 55.29, 55.26, 55.25, 55.21 (4*q*, 6 MeO) 51.11 (C(6''')); 32.85 (C(2)). HR-MALDI-MS (*m/z*): 1504.5677 (75, $[M + K]^+$, $C_{71}H_{83}N_{15}KO_{20}^+$; calc. 1504.5576); 1488.5839 (100, $[M + Na]^+$, $C_{71}H_{83}N_{15}NaO_{20}^+$; calc. 1488.5836). Anal. calc. for $C_{71}H_{83}N_{15}O_{20}$ (1466.51): C 58.15, H 5.70, N 14.33; found: C 58.16, H 5.89, N 14.31.

Procedure for the Alkylation of 111 and 112.

Under N_2, a 0.1M soln. of the alcohol (1 eq.) in DMF was treated with NaH (1.5 eq. for each OH group, 50–60% suspension in oil), the alkyl halide (1.5 eq. for each OHG group) and Bu_4NI (0.1 equiv.) were added, the mixture was stirred at 25° for 4–24 h, and diluted with H_2O and Et_2O. The aq. layer was extracted with Et_2O (3x). The combined org. phases were dried ($MgSO_4$) and evaporated to give the crude ethers.

*1,3,2',2''',6'''-Pentaazido-1,3,2',2''',6'''-Pentadeamino-6,3',2'',5'',3''',4'''-hexakis-O-(4-methoxybenzyl)-6'-O-(monomethoxytrityl)paromomycin (**112**)*. Alkylation of **111** and FC (AcOEt/cyclohexane 2:8) gave **112** (1.319 g, 65%). White solid. M.p. 60–65°. R_f (AcOEt/cyclohexane 3:7) 0.58. $[\alpha]_D^{25}$ = +96.4 (c = 0.12, $CHCl_3$). IR (ATR): 3500*w* (br.), 2927*w*, 2103*s*, 1611*m*, 1581*w*, 1513*s*, 1460*w*, 1362*w*, 1303*w*, 1249*s*, 1173*m*, 1033*s*. ^{1}H-NMR (500 MHz,

$CDCl_3$): δ 7.50–6.58 (*m*, 38 arom. H); 6.29 (d, *J* = 3.7; H–C(1')); 5.69 (d, *J* = 6.2, H–C(1'')); 4.89 (d, *J* = 1.9, H–C(1''')); 4.80 (d, *J* = 11.0), 4.76 (d, *J* = 10.3), 4.67 (d, *J* = 10.9), 4.60 (d, *J* = 11.9), 4.56 (d, *J* = 11.7), 4.52 (d, *J* = 10.9), 4.46 (d, *J* = 11.4), 4.41 (d, *J* = 11.2), 4.40 (d, *J* = 11.6), 4.34 (d, *J* = 11.4), 4.29 (d, *J* = 11.7), 4.19 (d, *J* = 11.8) (12 ArC*H*); 4.28 (ddd, *J* = 7.2, 3.0, 2.1, H–C(4'')); 4.21 (dd, *J* = 4.9, 2.1, H–C(3'')); 4.21–4.18 (m, H–C(5')); 4.06 (dd, *J* = 10.2, 9.1, H–C(3')); 3.99 (dd, *J* = 9.6, 9.5, H–C(5)); 3.98 (dd, *J* = 6.2, 4.9, H–C(2'')); 3.83 (dd, *J* = 9.6, 9.6, H–C(6)); 3.81 (dd, *J* = 10.4, 7.2, H_a–C(5'')); 3.80, 3.79, 3.78, 3.76, 3.75, 3.65, 3.55 (7*s*, 7 MeO); 3.76–3.72 (m, H–C(3'''), H–C(5''')); 3.60 (dd, *J* = 13.0, 8.7, H_a–C(6''')); 3.56 (m, H_b–C(5'')); 3.49–3.43 (m, H–C(3); H_a–C(6')); 3.49 (dd, J = 9.5, 9.5, H–C(4)); 3.43 (ddd, *J* = 12.4, 9.6, 5.4, H–C(1)); 3.39–3.38 (m, H–C(2''')); 3.31 (dd, *J* = 10.5, 9.1, H–C(4')); 3.16 (dd, 10.2, 3.7, H–C(2')); 3 .11 (dd, *J* = 10.0, 5.2, H_b–C(6')); 3.06 (m, H–C(4''')); 2.81 (dd, *J* = 13.0, 4.0, H_b–C(6''')); 2.23 (ddd, *J* = 13.0, 5.2, 4.5, H_{eq}–C(2)); 1.37 (ddd, *J* = 13.0, 12.7, 12.4, H_{ax}–C(2)). ^{13}C-NMR (125 MHz, $CDCl_3$): δ 159.68, 159.56, 159.46, 159.24, 159.10, 159.00, 158.65 (7*s*, 7 arom. C); 144.50, 144.36, 135.54, 130.44–131.18, 129.99–126.99 (several *s* and *d*, 9 arom. C and 26 arom. CH); 113.95, 113.87, 113.70, 113.67 (4*d*, 14 arom. CH); 105.94 (C(1'')); 98.68 (C(1''')); 95.71 (C(1')); 86.63 (s, *C*Ar_3); 74.79, 73.22, 73.00, 72.01, 71.30 (5*d*, 6 Ar*C*H_2); 84.07 (C(4)); 82.82 (C(5)); 82.24 (C(4'')); 81.88 (C(2'')); 80.16 (C(3')); 78.80 (C(4')); 75.76 (C(3'')); 74.48 (C(5''') *or* C(6)); 74.41 (C(6) *or* C(5''')); 72.52 (C(3''')); 71.19 (C(5')); 71.06 (C(4''')); 70.12 (C(5'')); 63.61 (C(2')); 60.47 (C(6')); 60.40 (C(1) *or* C(3)); 60.24 (C(3) or C(1)); 57.35 (C(2''')); 55.30, 55.28, 55.27, 55.23, 55.18, 55.14, 55.02 (7*q*, 7 MeO) 51.11 (C(6''')); 32.80 (C(2)). HR-MALDI-MS (*m/z*): 1777.6820 (100, $[M + 1 + K]^+$, $[C_{91}H_{99}N_{15}KO_{21}+1]^+$; calc. 1777.6777); 1776.6813 (92, $[M + K]^+$, $C_{91}H_{99}N_{15}KO_{21}^+$; calc. 1776.6777); 1761.7045 (85, $[M + 1 + Na]^+$, $[C_{91}H_{99}N_{15}NaO_{21}+1]^+$; calc. 1761.7038), 1760.7034 (85, $[M + Na]^+$, $C_{91}H_{99}N_{15}NaO_{21}^+$; calc. 1760.7038). Anal. calc. for $C_{91}H_{99}N_{15}O_{21}$ (1738.85): C 62.86, H 5.74, N 12.08; found: C 63.25, H 6.06, N 11.55.

*1,3,2',2''',6'''-Pentaazido-1,3,2',2''',6'''-Pentadeamino-6,3',2'',5'',3''',4'''-hexakis-*O-*(4-methoxybenzyl)-4'-O-(4-chlorobenzyl)-6'-*O-*(monomethoxytrityl)paromomycin* (**113**). Alkylation of **112** and FC gave **113** (AcOEt/cyclohexane 2:8) (518 mg, 96%). White solid. M.p. 66–71°. R_f (AcOEt/cyclohexane 3:7) 0.54. $[\alpha]_D^{25}$ = +94.1 (*c* = 0.08, $CHCl_3$). IR (ATR): 2932*w*, 2099*s*, 1611*m*,

1585*w*, 1512*s*, 1462*w*, 1359*w*, 1301*w*, 1245*s*, 1173*m*, 1110*m*, 1068*m*, 1031*s*. ^{1}H-NMR (500 MHz, $CDCl_3$): δ 7.53–6.57 (*m*, 42 arom. H); 6.30 (d, *J* = 3.6; H–C(1')); 5.69 (d, *J* = 6.2, H–C(1'')); 4.92 (d, *J* = 10.1), 4.76 (d, *J* = 10.3), 4.68 (d, *J* = 10.4), 4.62 (d, *J* = 11.4), 4.60 (d, *J* = 9.9), 4.56 (d, *J* = 11.3), 4.54 (d, *J* = 11.9), 4.50 (d, *J* = 11.4), 4.45 (d, *J* = 11.4), 4.41 (d, *J* = 11.8), 4.36 (d, *J* = 11.4), 4.30 (d, *J* = 11.8), 4.18 (d, *J* = 11.8, 2 H) (14 ArC*H*); 4.89 (d, *J* = 1.9, H–C(1''')); 4.28–4.26 (m, H–C(4'')); 4.22–4.20 (m, H–C(5')); 4.21 (dd, *J* = 4.9, 2.2, H–C(3'')); 4.08 (dd, *J* = 10.3, 9.0, H–C(3')); 3.99 (dd, *J* = 9.7, 8.8, H–C(5)); 3.97 (dd, *J* = 6.2, 4.9, H–C(2'')); 3.84–3.80 (m, H_a–C(5'')); 3.82 (dd, *J* = 9.7, 9.7, H–C(6)); 3.83–3.72 (m, H–C(3'''), H–C(5''')); 3.81, 3.80, 3.78, 3.77, 3.72, 3.65, 3.34 (7*s*, 7 MeO); 3.61 (dd, *J* = 12.9, 8.6, H_a–C(6''')); 3.56 (dd, J = 9.7, 8.8, H–C(4)); 3.56 (dd, *J* = 10.4, 3.0, H_b–C(5'')); 3.50–3.48 (m, H_a–C(6')); 3.49 (ddd, *J* = 12.5, 9.7, 4.9, H–C(1)); 3.43 (ddd, *J* = 12.5, 9.7, 4.6, H–C(3)); 3.39 (dd, *J* = 1.9, 1.9, H–C(2''')); 3.31 (dd, *J* = 9.5, 9.0, H–C(4')); 3.18 (dd, 10.3, 3.6, H–C(2')); 3.06 (m, H–C(4''')); 2.81 (dd, *J* = 12.9, 3.9, H_b–C(6''')); 2.23 (ddd, *J* = 13.2, 4.9, 4.6, H_{eq}–C(2)); 1.37 (ddd, *J* = 13.2, 12.5, 12.5, H_{ax}–C(2))., ^{13}C-NMR (125 MHz, $CDCl_3$): δ 159.65, 159.53, 159.37, 159.23, 159.07, 158.90, 158.58 (7*s*, 7 arom. C); 130.51–128.28, 127.84, 127.82, 126.89, 126.86 (several *s* and *d*, 11 arom. C and 28 arom. CH), 114.03, 113.87, 113.84, 113.70, 113.54, 113.08 (6*d*, 14 arom. CH); 86.05 (*s*, Ar_3*C*); 105.92 (C(1'')); 98.69 (C(1''')); 95.64 (C(1')); 84.08 (C(4)); 82.81 (C(5)); 82.22 (C(4'')); 81.89 (C(2'')); 80.18 (C(3')); 78.58 (C(4')); 75.67 (C(3'')); 75.32, 74.83, 73.66, 73.35, 73.11, 71.98, 71.24 (7*t*, 7 Ar*C*H_2), 74.40 (C(6) and (5''')); 72.52 (C(3''')); 71.24 (C(5')); 71.06 (C(4''')); 70.11(C(5'')); 63.59 (C(2')); 62.85 (C(6')); 60.48 (C(1) *or* C(3)); 60.19 (C(3) *or* C(1)); 57.35 (C(2''')); 55.27, 55.23, 55.13, 54.75 (4*q*, 7 MeO); 51.10 (C(6''')); 32.79 (C(2)) . HR-MALDI-MS (*m*/*z*): 1901.6918 (64, [*M* + 1 + K]$^+$, [$C_{98}H_{104}ClN_{15}KO_{21}$+1]$^+$; calc. 1901.6857); 1900.6892 (57, [*M* + K]$^+$, $C_{98}H_{104}ClN_{15}KO_{21}^+$; calc. 1900.6857); 1885.7180 (100, [*M* + 1 + Na]$^+$, [$C_{98}H_{104}N_{15}ClNaO_{21}$+1]$^+$; calc. 1885.7117); 1884.7151 (84, [*M* + Na]$^+$, $C_{98}H_{104}N_{15}ClNaO_{21}^+$; calc. 1884.7117). Anal. calc. for $C_{98}H_{104}ClN_{15}O_{21}\cdot 1.5\ H_2O$ (1890.44): C 62.26, H 5.71, N 11.11; found: C 62.39, H 5.83, N 10.73.

1,3,2',2''',6'''-Pentaazido-1,3,2',2''',6'''-Pentadeamino-6,3',2'',5'',3''',4'''-hexakis-O-(4-methoxybenzyl)-6'-O-(monomethoxytrityl)-4'-O-[4-(trifluoromethyl)benzyl]paromomycin (**114**). Alkylation of **112** and FC (AcOEt/cyclohexane 1:9 to 2:8) gave **114** (441 mg, 85%). White solid. M.p.

60–76°. R_f (AcOEt/cyclohexane 4:6) 0.54. $[\alpha]_D^{25}$ = +70.3 (*c* = 0.26, $CHCl_3$). IR (ATR): 2934*w*, 2100*s*, 1611*m*, 1586*w*, 1512*s*, 1463*w*, 1362*w*, 1325*m*, 1302*m*, 1245*s*, 1173*m*, 1111*m*, 1065*s*, 1030*s*. ^{1}H-NMR (500 MHz, $CDCl_3$): δ 7.53–6.58 (*m*, 42 arom. H); 6.26 (d, *J* = 3.6; H–C(1')); 5.69 (d, *J* = 6.2, H–C(1'')); 4.92 (d, *J* = 10.1), 4.78 (d, *J* = 10.4), 4.72 (d, *J* = 12.1), 4.65 (d, *J* = 10.4), 4.61 (d, *J* = 11.1), 4.57 (d, *J* = 11.3), 4.55 (d, *J* = 11.9), 4.52 (d, *J* = 11.4), 4.44 (d, *J* = 11.3), 4.41 (d, *J* = 11.8), 4.37 (d, *J* = 11.5, 2 H), 4.29 (d, *J* = 11.7), 4.19 (d, *J* = 11.9) (14 ArC*H*); 4.88 (d, *J* = 1.8, H–C(1''')); 4.28–4.27 (m, H–C(4'')); 4.14–4.11 (m, H–C(5')); 4.20 (dd, *J* = 4.9, 2.2, H–C(3'')); 3.98 (dd, *J* = 10.4, 9.3, H–C(3')); 3.97 (dd, *J* = 9.9, 8.8, H–C(5)); 3.98–3.96 (dd, *J* = 6.2, 4.7, H–C(2'')); 3.80–3.78 (m, H_a–C(5'')); 3.84 (dd, *J* = 9.9, 9.5, H–C(6)); 3.81, 3.80, 3.77, 3.76, 3.69, 3.65, 3.32 (7s, 7 MeO); 3.76–3.72 (m, H–C(3'''), H–C(5''')); 3.58 (dd, *J* = 12.9, 8.5, H_a–C(6''')); 3.55 (dd, *J* = 10.2, 2.9, H_b–C(5'')); 3.43 (ddd, *J* = 11.6, 9.8, 4.0, H–C(3)); 3.31 (dd, *J* = 9.8, 8.8, H–C(4)); 3.48 (ddd, *J* = 12.6, 9.5, 4.4, H–C(1)); 3.47–3.43 (m, H_a–C(6')); 3.38–3.36 (dd, *J* = 1.9, 1.9, H–C(2''')); 3.28 (dd, *J* = 9.8, 9.3, H–C(4')); 3.13 (dd, 10.4, 3.6, H–C(2')); 3.07 (m, H–C(4''')); 2.83 (dd, *J* = 12.9, 4.0, H_b–C(6''')); 2.23 (ddd, *J* = 12.9, 4.4, 4.0, H_{eq}–C(2)); 1.38 (ddd, *J* = 12.9, 12.6, 11.6, H_{ax}–C(2)). ^{13}C-NMR (125 MHz, $CDCl_3$): δ 159.66, 159.54, 159.39, 159.25, 159.08, 158.91, 158.59 (7*s*, 7 arom. C); 144.75, 144.32, 130.50–127.22, 126.90, 126.87, 125.04, 125.01 (several *s* and *d*, 10 arom. C and 28 arom. CH); 126.88 (*q*, $^1J_{(C,F)}$ = 37.0, CF_3); 114.03, 113.86, 113.84, 113.71, 113.66, 113.08 (6*d*, 14 arom. CH); 105.91 (C(1'')); 98.72 (C(1''')); 95.63 (C(1')); 86.06 (*s*, Ar_3C); 75.36, 74.85, 73.57, 73.37, 73.13, 71.99, 71.24 (7*t*, 7 ArCH_2), 84.10 (C(4)); 82.84 (C(5)); 82.23 (C(4'')); 81.92 (C(2'')); 79.96 (C(3')); 78.92 (C(4')); 75.74 (C(3'')); 74.37 (C(6') and C(5''')); 72.6 (C(3''')); 71.40 (C(5')); 71.15 (C(4''')); 70.12 (C(5'')); 63.51 (C(2')); 62.95 (C(6')); 60.52 (C(1) *or* C(3)); 60.27 (C(3) *or* C(1)); 55.28, 55.27, 55.24, 55.14, 55.07, 54.73 (6*q*, 7 OMe); 51.10 (C(6''')); 32.35 (C(2)). HR-MALDI-MS (*m*/*z*): 1935.7108 (100, $[M + 1 + K]^+$, $[C_{99}H_{104}F_3KN_{15}O_{21}+1]^+$; calc. 1935.7120); 1934.7084 (84, $[M + K]^+$, $C_{99}H_{104}F_3KN_{15}O_{21}^+$; calc. 1934.7120); 1919.7357 (95, $[M + 1 + Na]^+$, $[C_{99}H_{104}F_3N_{15}NaO_{21}+1]^+$; calc. 1919.7381); 1918.7333 (80, $[M + Na]^+$, $C_{99}H_{104}F_3N_{15}NaO_{21}^+$; calc. 1918.7381). Anal. calc. for $C_{99}H_{104}F_3N_{15}O_{21}$ (1896.97): C 62.68, H 5.53, N 11.08; found: C 62.42, H 5.62, N 10.66.

*Pentaazido-6,3',2'',5'',3''',4'''-1,3,2',2''',6'''-pentadeamino-1,3,2',2''',6'''-hexakis-*O*-(4-methoxybenzyl)-6'-*O*-(monomethoxytrityl)-4'-O-(3-phenylpropyl)paromomycin* (**115**). Alkylation of

112 and FC (AcOEt/cyclohexane 1:9 to 2:8) gave **115** (459 mg, 86%). White solid. M.p. 63–78°. R_f (AcOEt/cyclohexane 3:7) 0.60. $[\alpha]_D^{25}$ = +56.9 (*c* = 0.65, $CHCl_3$). IR (ATR): 2933*w*, 2099*s*, 1672*w*, 1611*m*, 1585*w*, 1512*s*, 1454*w*, 1363*w*, 1301*w*, 1245*s*, 1174*m*, 1029*s*. ^{1}H-NMR (500 MHz, $CDCl_3$): δ 7.56–6.57 (*m*, 43 arom. H); 6.28 (d, *J* = 3.7; H–C(1')); 5.69 (d, *J* = 6.1, H–C(1'')); 4.91 (*d*, *J* = 10.0), 4.72 (*d*, *J* = 10.3), 4.69 (*d*, *J* = 10.3), 4.59 (*d*, *J* = 10.2), 4.56 (*d*, *J* = 11.3), 4.55 (*d*, *J* = 11.9), 4.52 (*d*, *J* = 10.5), 4.50 (*d*, *J* = 11.5), 4.43 (*d*, *J* = 11.3), 4.41 (*d*, *J* = 11.8), 4.33 (*d*, *J* = 11.4), 4.29 (*d*, *J* = 11.2) (12 ArC*H*); 4.89 (d, *J* = 1.9, H–C(1''')); 4.28–4.27 (m, H–C(4'')); 4.22–4.15 (ddd, *J* = 10.3, 3.3, 1.9, H–C(5'); m, H–C(3'')); 4.06 (dd, *J* = 10.2, 9.0, H–C(3')); 3.98 (dd, *J* = 9.1, 8.0, H–C(5)); 3.98 (m, H–C(2'')); 3.81, 3.80, 3.78, 3.77, 3.76, 3.65, 3.33 (7*s*, 7 MeO); 3.80–3.78 (m, H_a–C(5'')); 3.84 (dd, *J* = 9.6, 9.1, H–C(6)); 3.75–3.71 (m, H–C(3'''), H–C(5''')); 3.70–3.67, 3.25 (ddd, *J* = 2.2, 7.0, 13.9, $PhCH_2CH_2CH_2$); 3.62–3.56 (m, H_a–C(6''') (dd, *J* = 10.2, 2.9, H_b–C(5'')); 3.42 (ddd, *J* = 12.7, 9.7, 4.6, H–C(3)); 3.55 (dd, J = 9.7, 8.9, H–C(4)); 3.55 (dd, J = 10.3, 3.3, H_a–C(6')); 3.51–3.45 (ddd, *J* = 12.3, 9.6, 4.5, H–C(1)); 3.39 (m, H–C(2''')); 3.31 (dd, *J* = 9.5, 9.0, H–C(4')); 3.16 (dd, *J* = 10.2, 3.7, H–C(2')); 3.07 (m, H–C(4''')); 2.83 (dd, *J* = 12.8, 3.9, H_b–C(6''')); 2.41 (*ddd*, *J* = 6.5, 9.8, 13.7), 2.27 (*ddd*, *J* = 6.2, 9.7, 13.8, $PhCH_2CH_2CH_2$); 2.22 (ddd, *J* = 12.7, 4.6, 4.6, H_{eq}–C(2)); 1.67–1.60 (*m*, $PhCH_2CH_2CH_2$); 1.38 (ddd, *J* = 12.7, 12.7, 12-3, H_{ax}–C(2)). ^{13}C-NMR (125 MHz, $CDCl_3$): δ 159.68, 159.56, 159.35, 159.26, 159.09, 158.92, 158.59 (7*s*, 7 arom. C); 144.82, 144.52, 141.99, 135.80, 130.49–128.28, 127.80, 126.89, 125.88, 125.71 (several *s* and *d*, 10 arom. C and 29 arom. CH); 114.05, 113.88, 113.72, 113.67, 113.09 (5*d*, 14 arom. CH); 86.02 (*s*, Ar_3C); 105.95 (C(1'')); 98.71 (C(1''')); 95.66 (C(1')); 86.08 (C(4)); 84.08 (C(5)); 82.22 (C(4'')); 81.90 (C(2'')); 80.14 (C(3')); 78.76 (C(4')); 75.72 (C(3'')); 75.19, 74.81, 73.38, 73.13, 72.01, 71.29 (6*t*, 6 $ArCH_2$); 74.45 (C(6) *or* C(5''')); 74.37 (C(5'') or C(6)); 72.61 (C(3''')); 72.32 (*t*, $PhCH_2CH_2CH_2$); 55.29, 55.24, 55.14, 55.10, 54.76 (5*q*, 7 MeO); 71.36 (C(5')); 71.16 (C(4''')); 70.13 (C(5'')); 63.64 (C(2')); 63.00 (C(6')); 60.51 (C(1) *or* C(3)); 60.17 (C(3) *or* C(1)); 57.39 (C(2''')); 51.11 (C(6''')); 32.09 (*t*, $PhCH_2CH_2CH_2$); 31.88 (*t*, $PhCH_2CH_2CH_2$); 30.68 (C(2)). HR-MALDI-MS (*m/z*): 1895.7531 (86, $[M + 1 + K]^+$, $[C_{100}H_{109}KN_{15}O_{21}+1]^+$; calc. 1895.7560), 1894.7513 (72, $[M + K]^+$, $C_{100}H_{109}KN_{15}O_{21}^+$; calc. 1894.7560), 1879.7787 (100, $[M + 1 + Na]^+$, $[C_{91}H_{99}N_{15}NaO_{21}+1]^+$; calc. 1879.7820), 1878.7773 (82,

$[M + Na]^+$, $C_{91}H_{99}N_{15}NaO_{21}^+$; calc. 1878.7820). Anal. calc. for $C_{100}H_{109}N_{15}O_{21}$ (1857.02): C 64.68, H 5.92, N 11.31; found: C 65.08, H 5.71, N 10.86.

Pentaazido-6,3',2'',5'',3''',4'''-1,3,2',2''',6'''-pentadeamino-1,3,2',2''',6'''-4'-O-(benzyloxymethyl)-hexakis-O-(4-methoxybenzyl)-6'-O-(monomethoxytrityl)paromomycin (**116**). Alkylation of **112** and FC (AcOEt/cyclohexane 1:9 to 2:8) gave **116** (442 mg, 83%). White solid. M.p. 52–55°. R_f (AcOEt/cyclohexane 3:7) 0.54. $[\alpha]_D^{27}$ = +237.2 (*c* = 0.035, $CHCl_3$). IR (ATR): 2933*w*, 2001*s*, 1611*m*, 1585*w*, 1513*s*, 1463*w*, 1361*w*, 1301*m*, 1247*s*, 1174*m*, 1111*m*, 1068 *m*,1033*s*. ^{1}H-NMR (500 MHz, $CDCl_3$): δ 7.55–6.55 (*m*, 43 arom. H); 6.28 (d, *J* = 3.7; H–C(1')); 5.69 (d, *J* = 6.1, H–C(1'')); 4.91 (d, *J* = 10.3), 4.68 (d, *J* = 11.1), 4.64 (d, *J* = 11.2), 4.61 (d, *J* = 10.3), 4.56 (d, *J* = 11.0), 4.54 (d, *J* = 11.9), 4.51 (d, *J* = 11.5), 4.44 (d, *J* = 11.3), 4.41 (d, *J* = 11.3), 4.35 (d, *J* = 10.9), 4.28 (d, *J* = 12.0), 4.19 (d, *J* = 12.0) (12 ArC*H*); 4.89 (d, *J* = 1.9, H–C(1''')); 4.75, 4.74 (*2d*, *J* = 6.5, OCH_2O); 4.65–4.58 (m, PhCH_2O); 4.22–4.15 (m, H–C(3'')); 4.28–4.27 (m, H–C(4'')); 4.22–4.15 (ddd, *J* = 9.5, 3.3, 1.9, H–C(5')); 4.06 (dd, 10.2, 9.0, H–C(3')); 3.98 (dd, *J* = 6.1, 4.8, H–C(2'')); 3.81, 3.80, 3.77, 3.76, 3.72, 3.65, 3.36 (7s, 7 MeO); 3.80–3.78 (m, H_a–C(5'')); 3.62–3.56 (m, H_b–C(5'')); 3.98 (dd, *J* = 9.1, 8.9, H–C(5)); 3.84 (dd, *J* = 9.6, 9.1, H–C(6)); 3.55 (m, H_a–C(6')); 3.80–3.78 (m, H_b–C(5'')); 3.75–3.71 (m, H–C(3'''), H–C(5''')); 3.62–3.56 (m, H_a–C(6''')); 3.55 (dd, *J* = 9.7, 8.9, H–C(4)); 3.51–3.45 (ddd, *J* = 12.3, 9.6, 4.5, H–C(1)); 3.42 (ddd, *J* = 12.7, 9.7, 4.5, H–C(3)); 3.39 (m, H–C(2''')); 3.31 (dd, J = 9.5, 9.0, H–C(4')); 3.16 (dd, *J* = 10.3, 1.9, H_b–C(6')); 3.16 (dd J = 10.2, 3.7, H–C(2')); 3.07 (m, H–C(4''')); 2.83 (dd, *J* = 12.8, 3.9, H_b–C(6''')); 2.22 (ddd, *J* = 12.9, 4.4, 4.0, H_{eq}–C(2)); 1.35 (ddd, *J* = 12.7, 12.7, 12.3, H_{ax}–C(2)). ^{13}C-NMR (125 MHz, $CDCl_3$): δ 105.95 (C(1'')); 98.71 (C(1''')); 95.66 (C(1')); 86.08 (C(4)); 84.08 (C(5)); 82.22 (C(4'')); 81.90 (C(12'')); 80.14 (C(3')); 78.76 (C(4')); 75.72 (C(3'')); 74.45 (C(6) or C(5''')); 74.37 (C(5''') or C(6)); 72.61 (C(3''')); 71.36 (C(5')); 71.16 (C(4''')); 70.13 (C(5'')); 63.64 (C(2')); 63.00 (C(6')); 60.51 (C(1) or C(3)); 60.17 (C(3) or C(1)); 57.39 (C(2''')); 51.11 (C(6''')); 30.68 (C(2)). HR-MALDI-MS (*m/z*): 1897.7351 (18, $[M + 1 + K]^+$, $[C_{99}H_{107}KN_{15}O_{22}+1]^+$; calc. 1897.7352), 1896.7326 (16, $[M + K]^+$, $C_{99}H_{107}KN_{15}O_{22}^+$; calc. 1896.7352), 1881.7593 (27, $[M + 1 + Na]^+$, $[C_{99}H_{107}N_{15}NaO_{22}+1]^+$; calc. 1881.7613), 1880.7566 (22, $[M + Na]^+$, $C_{99}H_{107}N_{15}NaO_{22}^+$; calc. 1880.7613), 1867.7199 (71, $[M - CH_2{=}O + 1 + K]^+$, $[C_{98}H_{105}KN_{15}O_{21}+1]^+$; calc. 1867.7247), 1866.7181 (65, $[M - CH_2{=}O + K]^+$,

$C_{98}H_{105}KN_{15}O_{21}^+$; calc. 1866.7247), 1851.7429 (100, $[M - CH_2{=}O + 1 + Na]^+$, $[C_{98}H_{105}N_{15}NaO_{21}+1]^+$; calc. 1851.7507), 1850.7403 (89, $[M - CH_2{=}O + Na]^+$, $C_{98}H_{105}N_{15}NaO_{21}^+$; calc. 1850.7507). Anal. calc. for $C_{99}H_{107}N_{15}O_{22}$ (1858.99): C 63.96, H 5.80, N 11.30; found: C 63.74, H 5.63, N 10.78.

Procedure for the Deprotection of 113–116.

Under N_2, a 0.02M soln. of **113-116** (1 equiv.) in CH_2Cl_2/MeOH/H_2O 20:1:0.4 was treated with DDQ (1.1 equiv. per PMB and MMTr group), stirred at 25° for 12–24 h, neutralized with 1N NaOH, and diluted with AcOEt. After separation of phases, the aq. layer was extracted with AcOEt (3 x). The combined org. layers were dried ($MgSO_4$) and evaporated to yield the azido alcohols. (R_f values ($CHCl_3$/AcOEt/MeOH/ 2:2:0.5) of the intermediate azido alcohols: 0.29 (from **113**), 0.25 (from **114**), 0.41 (from **115**), and 0.31 (from **116**)). A 0.03M soln. of the azido alcohols (1 equiv.) in THF/0.1N NaOH 4:1 was treated with 1M PMe_3 in THF (1.2 equiv. per azido group), stirred for 4–8 h at 50°, and evaporated.

*4'-O-(4-chlorobenzyl)paromomycin (**39**)*. Deprotection of **113** and FC (MeOH/25% aq. NH_3 9:1→8:2) gave **39** (47 mg, 86%). White solid. M.p. 145° (dec.). R_f (MeOH/25% aq. NH_3 8:2) 0.27. $[\alpha]_D^{25}$ = +60.3 (c = 0.27, H_2O). IR (ATR): 3358*m* (br.), 3286*m* (br.), 2915*m*, 1590*w*, 1491*w*, 1454*w*, 1366*w*, 1088*s*, 1014*s*. ^{1}H-NMR (500 MHz, D_2O): δ 7.43–7.37 (*m*, 4 arom. H); 5.34 (d, J = 3.7, H–C(1')); 5.33 (d, J = 2.6), H–C(1'')); 4.97 (J = 1.9, H–C(1''')); 4.82 , 4.67 (2*d*, J = 10.9, PhCH_2). 4.43 (dd, J = 6.4, 4.9, H–C(3'')); 4.26 (dd, J = 4.9, 2.6, H–C(2'')); 4.11 (ddd, J = 8.0, 6.4, 3.2, H–C(4'')); 4.05 (ddd, J = 8.2, 3.9, 1.5, H–C(5''')); 4.03 (dd, J = 3.2, 3.1, H–C(3''')); 3.85 (dd, J = 12.4, 3.2, H_a–C(5'')); 3.84–3.79 (m, H–C(3'), H–C(4) H–C(5)); 3.74–3.70 (m, H–C(5'), H_b–C(5'), H_a–C(6')); 3.47–3.41 (m, H–C(6), H_b–C(6')); 3.29 (dd, J = 9.7, 9.7, H–C(4')); 3.17 (dd, J = 13.5, 3.9, H_a–C(6''')); 3.04 (ddd, J = 3.1, 1.9, 1.2, H–C(2''')); 3.08 (dd, J = 13.5, 8.2, H_b–C(6''')); 2.87 (ddd, J = 12.2, 9.5, 4.2, H–C(3)); 2.78 (m, H–C(2')); 2.77 (m, J(1,2ax), J(1,2eq) = 4.2, H–C(1)); 1.96 (ddd, J = 12.6, 4.2, 4.2, H_{eq}–C(2)); 1.21 (ddd, J = 12.6, 12.2, 12.1, H_{ax}–C(2)), 7.43–7.37 (*m*, 4 arom. H); 4.82 , 4.67 (2*d*, J = 10.9, PhCH_2). ^{13}C-NMR (125 MHz, D_2O): δ 138.44 (*s*); 136.31 (*s*); 133.02 (2*d*); 131.33 (2*d*); 111.18 (C(1'')); 101.74 (C(1'''); 101.65 (C(1')); 86.59 (C(5)); 85.68 (C(4'')); 84.02 (C(4'80.58 (C(4)); 79.41 (C(3'')); 78.27 (C(2'')); 76.80 (*s*, ArCH_2); 75.98 (C5')); 75.90 (C(6)); 75.69 (C(5''')); 74.69 (C(3')); 73.00 (3''')); 71.18 (C(4''')); 63.56 (C(5'')); 63.05 (C(6')); 58.21 (C(2')); 55.05

(C(2’’’)); 52.92 (C(1)); 52.71 (C(3)); 43.51 (C(6’’’)); 37.46 (C2)). HR-MALDI-MS (*m*/*z*): 762.2921 (100, $[M + Na]^+$, $C_{30}H_{50}N_5NaO_{14}^+$; calc. 762.2940), 740.3110 (86, $[M + H]^+$, $C_{30}H_{51}N_5O_{14}^+$; calc. 740.3121).

*1,3,2’,2’’’,6’’’-Pentaammonium-4’-O-[4-(trifluoromethyl)benzyl]paromomycin Pentaacetate (**41**·*5AcOH*)*. Deprotection of **114** and FC (MeOH/25% aq. NH_3 9:1 → 8:2), followed by dissolving the crude product in 10% aq. HOAc, concentration, and lyophilisation gave **41**·5AcOH (84 mg, 87%). White solid. M.p. 148° (dec.). R_f (MeOH/25 aq. NH_3 8:2) 0.30. $[\alpha]_D^{25}$ = +65.4 (*c* = 0.16, H_2O). IR (ATR): 3500-2700*m*, 1772*w*, 1551*m*, 1403*m*, 1326*s*, 1241*w*, 1107*s*, 1064*s*, 1046*s*, 1016*s*. ^{1}H-NMR (500 MHz, D_2O): δ 7.73 (*d*, *J* = 8.1, 2 arom. H); 7.57 (*d*, *J* = 8.1, 2 arom. H); 5.59 (d, *J* = 3.8, H–C(1’)); 5.35 (d, *J* = 2.2), H–C(1’’)); 5.20 (H–C(1’’’)); 4.93 , 4.78 (2*d*, *J* = 11.3, PhC*H₂*); 4.50 (dd, *J* = 6.4, 4.8, H–C(3’’)); 4.35 (dd, *J* = 4.8, 2.2, H–C(2’’)); 4.27 (m, H–C(5’’’)); 3.78–3.73 (m, H–C(5’), H_a–C(6’)); 3.76 (m, H–C(4’’)); 4.18–4.16 (m, H–C(4’’), H–C(3’’’)); 3.93 (dd, *J* = 9.6, 9.4 H–C(3’)); 3.93–3.82 (m, H–C(4), H–C(5), H_a–C(5’’)); 3.74 (dd, J = 12.3, 4.9, H_b–C(5’’)); 3.60 (m, H_b–C(6’)); 3.54 (m, H–C(4’)); 3.55 (d, *J* = 9.2, H–C(6)); 3.42 (m, H–C(2’’’)); 3.38 (dd, *J* = 13.5, 7.1, H_a–C(6’’’)); 3.32 (dd, *J* = 13.5, 3.9, H_b–C(6’’’)); 3.19 (dd, *J* = 10.6, 3.8, H–C(2’)); 3.14 (m, H–C(3)); 3.05 (ddd, *J* = 12.9, 9.2, 4.2, H–C(1)); 2.18 (ddd, *J* = 12.6, 4.1, 4.1, H_{eq}–C(2)); 1.88 (*s*, 5 AcO); 1.49 (ddd, *J* = 12.7, 12.6, 12.5, H_{ax}–C(2)). ^{13}C-NMR (125 MHz, D_2O): δ 184.14 (*s*, 5 C=O); 147.77 (*s*); 131.57 (*s* and 2*d*); 128.25 (2*d*); 126.83 (*q*, ^1J(C,F) = 271.5, CF_3); 112.27 (C(1’’)); 99.67 (C(1’)); 99.03 (C(1’’’)); 87.23 (C(5)); 83.90 (C(4’’’)); 83.83 (C(4’)); 80.343 (C(4)); 77.97 (C(3’’)); 77.05 (*t*, Ar*C*H_2); 25.93 (*q*, 5 Me) 76.42 (C(2’’)); 75.96 (C(5’)); 75.01 (C(6)); 73.47 (C(5’’’)); 73.21 (C(3’)); 71-14 (C(3’’’)); 70.86 (C(4’’’)); 62.95 (C(5’’)); 62.77 (C(6’)); 56.32 (C(2’)); 53.95 (C(2’’’)); 53.04 (C(1)); 51.98 (C(3)); 43.15 (C(6’’’)); 33.39 (C(2)). HR-MALDI-MS (*m*/*z*): 796.3201 (72, $[M + Na]^+$, $C_{31}H_{50}N_5F_3NaO_{14}^+$; calc. 796.3204),774.3366 (100, $[M + H]^+$, $C_{31}H_{51}F_3N_5O_{14}^+$; calc. 774.3385).

*4’-O-(3-Phenylpropyl)paromomycin (**42**)*. Deprotection of **115** and FC ($CHCl_3$/MeOH/25% aq. NH_3 1:3:1) gave **42** (64 mg, 92%).White solid. M.p. 179° (dec.). R_f ($CHCl_3$/MeOH/25% aq. NH_3 1:3:2) 0.47. $[\alpha]_D^{25}$ = +62.9 (*c* = 0.12, H_2O). IR (ATR): 3500-2700*m*, 2920*w*, 1572*w*, 1495*w*, 1454*w*, 1381*w*, 1339*w*, 1015*s*. ^{1}H-NMR (500 MHz, D_2O): δ 7.37–7.23 (*m*, 5 arom. H); 5.41 (d, *J* = 3.7, H–

C(1')); 5.34 (d, J = 2.6), H–C(1'')); 5.04 (d, J = 2.6, H–C(1''')); 4.45 (dd, J = 6.5, 5.0, H–C(3'')); 4.29 (dd, J = 5.0, 2.6, H–C(2'')); 4.16 (ddd, J = 7.6, 3.8, 1.6, H–C(5''')); 4.13 (m, J(3'',4'') = 6.5, J(2,''3'') = 5.0, H–C(4'')); 4.06 (dd, J = 6.5, 5.0, H–C(3''')); 3.87–3.78 (m, H–C(5'), H_a–C(6')); 3.87–4.78, 3.74–3.65 (2*m*, $PhCH_2CH_2CH_2$); 3.86 (dd, 12.5, 3.3, H_a–C(5'')); 3.74–3.65 (m, H–C(4'''), H–C(5), H–C(5''), H_b–C(6')); 3.65 (dd, J = 10.5, 9.6, H–C(3')); 3.41 (d. J = 9.7, H–C(6)); 3.30 (dd, J = 13.5, 3.8, H_a–C(6''')); 3.26 (dd, J = 9.6, 9.4, H–C(4')); 3.23 (dd, J = 13.5, 7.6, H_b–C(6''')); 3.14 (m, J(2''',3''') = 3.2, J(1''',2''') = 1.8, H–C(2''')); 2.98–2.91 (m, H–C(1), H–C(3)); 2.88 (dd, J = 10.5, 3.7, H–C(2')); 2.72– 2.65 (*m*, $PhCH_2CH_2CH_2$); 1.90 (*dt*, J = 7.2, 14.1, $CH_2CH_2CH_2$); 2.06 (ddd, J = 12.6, 14.1, 4.1, H_{eq}–C(2)); 1.33 (ddd, J = 12.7, 12.6, 12.5, H_{ax}–C(2)). ^{13}C-NMR (125 MHz, D_2O): δ 144.87 (*s*); 131.34 (2*d*); 131.19 (2*d*); 128.76 (*d*); 111.61 (C(1'')); 101.03 (C(1')); 100.86 (C(1'''));86.84 (C(5)); 84.88 (C(4)); 83.91(C(4'')); 80. 70 (C(4')); 78.04 (C(6)); 77.98 (C(3'')); 75.96 (C(2'')); 75.22 (*t*, $PhCH_2CH_2CH_2$); 34.12 (*t*, $PhCH_2CH_2CH_2$); 74.90 (C(5')); 74.80 (C(3')); 74.06 (C(5''')); 72.45 (C(3''')); 70.98 (C(4''')); 63.23 (C(5'') *or* C(6')); 62.95 (C(6') *or* C(5'')); 57.84 (C(2')); 53.01(C(1) or C(3)); 54.68 (C(2''')); 52.46 (C(3) *or* C(1)); 43.34 (C(6''')); 36.05 (C(2)); 33.67 (*t*, $PhCH_2CH_2$ CH_2). HR-MALDI-MS (*m/z*): 756.3648 (37, $[M + Na]^+$, $C_{32}H_{56}N_5NaO_{14}^+$; calc. 756.3643), 734.3805 (100, $[M + H]^+$, $C_{32}H_{56}N_5O_{14}^+$; calc. 734.3824).

*4'-O-(Benzyloxymethyl)paromomycin (**43**)*. Deprotection of **116** and FC ($CHCl_3$/MeOH/25% aq. NH_3 1:3:1) gave **43** (36 mg, 72%) White solid. M.p. 143° (dec.). R_f ($CHCl_3$/MeOH/25% aq. NH_3 1:3:2) 0.36. $[\alpha]_D^{27}$ = +48.9 (c = 0.24, H_2O). IR (ATR): 3500-2700*m*, 1622*w*, 1525*w*, 1429*m*, 1043*s*. ^{1}H-NMR (500 MHz, D_2O): δ 7.47–7.42 (*m*, 5 arom. H); 5.83 (d, J = 3.8, H–C(1')); 5.42 (d, J = 2.4, H–C(1'')); 5.33 (d, J = 1.7, H–C(1''')); 5.03, 4.96 (2*d*, J = 7.1, OCH_2O); 4.76–4.74 (*m*, $PhCH_2$). 4.58 (dd, J = 6.8, 4.7, H–C(3'')); 4.45 (dd, J = 6.8, 4.7, H–C(2'')); 4.36 (ddd, J = 7.1, 4.0, 1.5, H–C(5''')); 4.27 (m, H–C(3''')); 4.25 (dd, J = 6.8, 4.6, 2.4, H–C(4'')); 4.12–4.07 (m, H–C(4), H–C(5')); 4.11 (dd, J = 9.1, 8.8, H–C(3')); 3.97 (dd, J = 9.1, 9.1, H–C(5)); 3.81–3.75 (dd, J_{gem} = 12.6, H_a–C(6')); 3.99–3.88 (m, H_a–C(5''), H_a–C(6')); 3.81–3.75 (m, H_b–C(6'), H_b–C(5''), H–C(6)); 3.85–3.84 (m, H–C(4''')); 3.64 (dd, *J(3',4')* = 9.1, H–C(4')); 3.63–3.60 (m, H–C(2''')); 3.50 (dd, J = 10.7, 3.8, H–C(2')); 3.47–3.42 (m, $J(2_{ax},3)$ = 12.6, $J(2_{eq},3)$ = 4.1, H–C(3)); 3.44 (dd, J = 13.6, 4.0, H_a–C(6''')); 3.42–3.35 (dd, J = 12.6, 4.1, H–C(1)); 3.38 (dd, J = 13.6, 7.1, H_b–C(6''')); 2.54 (ddd, J = 12.6, 12.6, 4.1, H_{eq}–C(2)); 1. 95

(ddd, *J* = 12.6, 12.6, 12.6, H_{ax}–C(2)). ^{13}C-NMR (125 MHz, D_2O): δ 139.37 (*s*); 131.61 (*d*); 131.49 (2*d*); 131.15 (2*d*); 112.73 (C(1'')); 98.47 (*s*, O*C*H_2O); 98.34(C(1')); 97.81(C(1''')); 86.76 (C(5)); 83.88 (C(4'')); 80.19 (C(4)); 78.62 (C(3')); 77.49 (C(3'')); 75.82 (C(2'')); 75.62 (C(5')); 74.81 (C(6)); 73.48 (*t*, Ph*C*H_2); 73.02 (C(5''')); 71.22 (C(3')); 70.34 (C(3''')); 69.81 (C(4''')); 62.76 (C(5''') *or* C(6')); 62.71 (C(6') *or* C(5'')); 56.34 (C(2')); 523.50 (C(2''')); 52.45 (C(1)); 51.51 (C(3)); 43.03 (C(6''')); 30.68 (C(2)).. HR-MALDI-MS (*m*/*z*): 758.3428 (21, [*M* + Na]$^+$, $C_{31}H_{53}N_5NaO_{15}^+$; calc. 758.3436), 736.3598 (100, [*M* + H]$^+$, $C_{31}H_{54}N_5O_{15}^+$; calc. 736.3616).

2.3. Synthesis of 40.

*1,3,2',2''',6'''-Pentaazido-1,3,2',2''',6'''-Pentadeamino-4'-O-(4-methoxybenzyl)paromomycin (**117**).* Under N_2, **48** (204 mg, 0.236 mmol) was cooled to -5°, treated with 1M BH_3·THF in THF (2.36 ml) and 1M Bu_2BOTf in CH_2Cl_2 (0.236 ml), stirred at -5° to 0° for 50 min., treated with Et_3N (0.1ml) and MeOH (0.1ml), and coevaporated with MeOH three times. FC (AcOEt/$CHCl_3$/MeOH 2:2:0.1–2:2:0.2) gave **117** (120 mg, 59%). White solid. M.p. 65–68°. R_f (AcOEt/$CHCl_3$/MeOH 2:2:0.25) 0.55. $[\alpha]_D^{25}$ = +99.3 (*c* = 1.63, MeOH). IR (ATR): 3374*m* (br.), 2942*w*, 2874*w*, 2102*s*, 1770*w*, 1631*w*, 1612*w*, 1514*w*, 1453*w*, 1369*w*, 1331*w*, 1248*m*, 1143*w*, 1101*m*, 1075*m*, 1027*s*. ^{1}H-NMR (500 MHz, $CDCl_3$): δ 5.81 (d, *J* = 3.7; H–C(1')); 5.38 (d, *J* = 1.9, H–C(1'')); 5.13 (d, *J* = 1.9, H–C(1''')); 4.91 (d, *J* = 10.3), 4.68 (d, *J* = 11.1), 4.64 (d, *J* = 11.2), 4.61 (d, *J* = 10.3), 4.56 (d, *J* = 11.0), 4.54 (d, *J* = 11.9), 4.51 (d, *J* = 11.5), 4.44 (d, *J* = 11.3), 4.41 (d, *J* = 11.3), 4.35 (d, *J* = 10.9), 4.28 (d, *J* = 12.0), 4.19 (d, *J* = 12.0) (12 ArC*H*); 4.75, 4.74 (2*d*, *J* = 6.5, OC*H$_2$*O); 4.65–4.58 (*m*, PhC*H$_2$*O); 4.44 (dd, J = 6.6, 4.6, H–C(3'')); 4.14 (dd, *J* = 10.0, 2.8, H–C(4'')); 4.29 (dd, *J* = 6.6, 1.9, H–C(2'')); 4.11 (dd, *J* = 10.5, 8.9, H–C(3')); 3.96 (ddd, *J* = 10.0, 4.3, 2.1, H–C(5')); 3.83 (dd *J* = 12.0, 2.8, H_a–C(5'')); 3.81, 3.80, 3.77, 3.76, 3.72, 3.65, 3.36 (7*s*, 7 MeO); 3.73–3.63 (dd, *J* = 12.0, 10.0, H_b–C(5'')); 3.73–3.63 (m, H–C(5), H–C(6), H_b–C(6')); 4.01 (ddd, J = 8.3, 4.6, 1.9, H–C(5''')); 3.93 (m, H–C(3'''), 3.65 (dd, J = 12.3, 8.3; H_a–C(6''')); 3.39 (dd, *J* = 12.0, 10.0, H_b–C(5'')); 3.51–3.40 (m, H–C(1), H–C(3), H–C(4), H–C(4''')); 3.93 (m, H–C(3''')); 3.77 (dd, *J* = 8.5, 2.1, H_a–C(6')); 3.39 (dd, *J* = 12.3, 4.6, H_b–C(6''')); 3.13 (dd, *J* = 10.5, 3.7, H–C(2')); 2.16 (ddd, *J* = 12.7, 4.3, 4.3, H_{eq}–C(2)); 1.35 (ddd, *J* = 12.7, 12.6, 12.6, H_{ax}–C(2)). ^{13}C-NMR (125 MHz, $CDCl_3$,): δ 159.68, 159.56, 159.37, 159.25, 159.09, 158.94,

158.58 (7*s*, 7 arom. C); 144.85, 144.50, 138.12, 135.70, 130.54–127.38, 126.86 (several *s* and *d*, 10 arom. C and 29 arom. CH); 114.05, 114.00, 113.87, 113.80, 113.72, 113.67, 113.12 (7*d*, 14 arom. CH); 82.80 (*s*, Ar_3C); 109.08 (C(1'')); 99.80 (C(1''')); 97.90 (C(1')); 85.30(C(5)); 83.52 (C(4'')); 79.30 (C(4')); 77.14 (C(4)); 77.02 (C(3'')); 76.44 (C(6)); 75.56 (C(1'')); 75.19 (C(5''')); 75.29 (*t*, OCH_2O); 74.81, 76.62, 73.34, 73.13, 72.01, 71.29 (6*t*, 6 $ArCH_2$, PhCH_2O), 73.34 (C(5')); 72.71 (C(3')); 71.17 (C(3''')); 69.60 (C(4''')); 63.32 (C(5'')); 62.17 (C(6')); 61.85 (C(2''')); 61.85 (C(1) *or* C(3)); 61.54 (C(3) *or* C(1)); 55.29, 55.24, 55.13, 54.79 (4*q*, 7 MeO) 52.44 (C(6''')); 33.08 (C(2)). HR-MALDI-MS (*m*/*z*): 888.2942 (100, $[M + Na]^+$, $C_{31}H_{43}N1_5NaO_{15}^+$; calc. 888.2961); 456.0339 (91, $[M + H + 2Na]^{2+}$, $C_{15.5}H_{22}N_{7.5}NaO_{7.5}^{2+}$; calc. 456.1468).

*1,3,2',2''',6'''-Pentaammonium-4'-O-(4-methoxybenzyl)paromomycin Pentaacetate (***40** *· 5AcOH)*. Staudinger reaction and hydrolysis, as described above, of **117** and FC (MeOH/$CHCl_3$/25% aq. NH_3 1:3:1 → 1:3:2), followed by dissolving crude **40** in 10% aq. HOAc, concentration, and lyophilisation gave **40** · 5AcOH (44 mg, 74%). White solid. M.p. 190° (dec.). R_f (MeOH/$CHCl_3$/25% aq. NH_3 1:3:2) 0.33. $[\alpha]_D^{25}$ = +48.9 (*c* = 0.32, H_2O). IR (ATR): 3500-2700*m*, 2874*m*, 1609*w*, 1513*s*, 1405*m*, 1333*w*, 1248*w*, 1050*s*, 1031*s*. ^{1}H-NMR (500 MHz, D_2O): δ 7.41 (*d*, *J* = 8.7, 2 arom. H); 7.04 (*d*, *J* = 8.7, 2 arom. H); 5.76 (d, *J* = 3.9, H–C(1')); 5.40 (d, *J* = 2.4), H–C(1'')); 5.32 (d, *J* = 1.7, H–C(1''')); 4.83, 4.67 (2*d*, *J* = 10.5, ArCH_2); 4.56 (dd, *J* = 6.7, 4.8, H–C(3'')); 4.29 (ddd, *J* = 6.7, 4.7, 3.3, H–C(4''));4.42 (dd, *J* = 4.8, 2.4, H–C(2'')); 4.35 (ddd, *J* = 6.5, 3.9, 1.4, H–C(5''')); 4.26 (dd, *J* = 3.1, 3.1, H–C(3''')); 4.03 (dd, *J* = 9.9, 9.0, H–C(3')); 3.93 (dd, *J* = 12.4, 3.3, H_a–C(5'')); 3.90 –3.86 (m, H–C(5), H–C(4)); 3.87–3.84 (dd, J_{gem} = 12.0, H_a–C(6')); 3.85 (*q*, MeO); 3.84–3.80 (m, H–C(5'), H–C(4''')); 3.79 (dd, *J* = 12.0,4.7, H_b–C(5'')); 3.74 (dd, *J* = 12.0, 5.6, H_b–C(6')); 3.71 (dd, *J* = 8.9, 8.9, H–C(6)); 3.61 (ddd, *J* = 3.1, 1.7, 1.2, H–C(2''')); 3.56 (dd, *J* = 9.3, 9.0, H–C(4')); 3.46 (dd, *J* = 13.5, 6.5, H_a–C(6''')); 3.43 (dd, *J* = 9.9, 3.9, H–C(2')); 3.38 (dd, *J* = 13.5, 3.9, H_b–C(6''')); 3.31 (dd, *J* = 12.4, 4.1, H–C(1)); 2.84–2.67 (dd, *J* = 12.6, 4.3 H–C(3)); 2.39 (ddd, *J* = 12.9, 12.6, 4.3, H_{eq}–C(2)); 1.93 (*s*, 15 AcO); 1. 77 (ddd, *J* = 12.9, 12.6, 12.4, H_{ax}–C(2)). ^{13}C-NMR (125 MHz, D_2O): δ 161.75 (*s*); 133.35 (2*d* and *s*); 132.16 (*d*); 116.84 (2*d*); 112.61 (C(1'')); 98.61 (C(1')); 97.97 (C(1''')); 87.06 (C(5)); 83.86 (C(4'')); 81.62 (C(4)); 79.45 (C(4')); 77.65 (C(3'')); 77.62 (*t*, PhCH_2). 58.04 (*q*, MeO). 25.68 (*s*, 5 Me); 75.90 (C(2'')); 75.53 (C(5')); 75.25 (C(6)); 72.99 (C(5''')); 72.13 (C(3')); 70.38

(C(3’’’)); 69.86 (C(4’’’)); 62.79 (C(5’’)); 62.68 (C(6’)); 56.71 (C(2’)); 53.53(C(2’’’)); 52.66 (C(1)); 51.59 (C(3)); 43.03 (C(6’’’)); 32.04 (C(2)). HR-MALDI-MS (*m*/*z*): 736.3597 (100, $[M + H]^+$, $C_{31}H_{54}N_5O_{15}^+$; calc. 736.3616)

Supplementary References

50. Pathak, R., Böttger, E. C. & Vasella, A. Design and synthesis of aminoglycoside antibiotics to selectively target 16S ribosomal RNA position 1408. *Helv. Chim. Acta* **88**, 2967-2984 (2005).

51. Clerici, A., Pastori, O. & Porta, N. Efficient acetalisation of aldehydes catalyzed by titanium tetrachloride in a basic medium. *Tetrahedron* **54**, 15679-15690 (1998).

52. Hassner, A., Wiederkehr, E. & Kascheres, A. J. Reaction of aldehydes with N-hydroxybenzenesulfonamide. Acetal formation catalyzed by nucleophiles. *J. Org. Chem.* **35**, 1962-1964 (1970).

53. Bayley, H., Standring, D. N. & Knowles, J. R. Propane-1,3-dithiol: A selective reagent for the efficient reduction of alkyl and aryl azides to amines. *Tetrahedron Lett.* **19**, 3633 (1978).
